# Are Bayesian regularization methods a must for multilevel dynamic latent variables models?

**DOI:** 10.3758/s13428-024-02589-9

**Published:** 2025-01-22

**Authors:** Vivato V. Andriamiarana, Pascal Kilian, Holger Brandt, Augustin Kelava

**Affiliations:** Methods Center, Eberhard Karls University of Tübingen, Haußerstr. 11, 72076 Tübingen, Germany

**Keywords:** Bayesian regularization, Sparsity, Dynamic latent variable models, Markov switching, Intensive longitudinal data

## Abstract

Due to the increased availability of intensive longitudinal data, researchers have been able to specify increasingly complex dynamic latent variable models. However, these models present challenges related to overfitting, hierarchical features, non-linearity, and sample size requirements. There are further limitations to be addressed regarding the finite sample performance of priors, including bias, accuracy, and type I error inflation. Bayesian estimation provides the flexibility to treat these issues simultaneously through the use of regularizing priors. In this paper, we aim to compare several Bayesian regularizing priors (ridge, Bayesian Lasso, adaptive spike-and-slab Lasso, and regularized horseshoe). To achieve this, we introduce a multilevel dynamic latent variable model. We then conduct two simulation studies and a prior sensitivity analysis using empirical data. The results show that the ridge prior is able to provide sparse estimation while avoiding overshrinkage of relevant signals, in comparison to other Bayesian regularization priors. In addition, we find that the Lasso and heavy-tailed regularizing priors do not perform well compared to light-tailed priors for the logistic model. In the context of multilevel dynamic latent variable modeling, it is often attractive to diversify the choice of priors. However, we instead suggest prioritizing the choice of ridge priors without extreme shrinkage, which we show can handle the trade-off between informativeness and generality, compared to other priors with high concentration around zero and/or heavy tails.

**Supplementary Information:**

The online version contains supplementary material available at 10.3758/s13428-024-02589-9.

In recent years, the ease of collecting intensive longitudinal data (ILD) has significantly increased in the social and cognitive sciences, in accordance with the increase in computational resources. Such advantages have made it easier for researchers to explore complex phenomena using multilevel dynamic latent variable models (DLVMs). For example, DLVMs for ILD allow researchers to model dynamic relationships between latent measurement error-free variables, in order to investigate relationships between psychological constructs. DLVMs offer the opportunity to differentiate between inter- and intra-individual changes. In combination with Markov switching models, they even offer novel ways to address relevant practical problems such as student drop-out (e.g., Heusel et al., 2023; Kelava et al., 2022), inattention (e.g., Roman et al., 2024), or psychometric changes of measurement instruments (e.g., Flückiger et al., 2022). Naturally, these models can become very complex, incorporating features such as large numbers of predictors, a between-level structure, or Markov switching states. Therefore, it is important for this complexity to be addressed using appropriate estimation methods.

In the context of DLVMs, there are two major challenges stemming from model complexity that must be addressed: overfitting and small sample sizes. Overfitting occurs when the implementation of a large number of predictors (*P*) leads to a false discovery of active predictors. Consequently, a sparse estimation becomes necessary to reduce model complexity (e.g., McNeish, 2015; Serang et al., 2017; Williams and Rodriguez, 2022). Moreover, increased data availability in some areas often allows researchers to increase dimensionality or parameters, particularly in *P*, rather than alleviating issues of small sample sizes. If the number of individuals (*N*) and time points (*T*) for ILD is insufficient to achieve adequate statistical power, it is crucial to address sample size requirements (e.g., Andriamiarana et al., 2023; McNeish, 2019; Schultzberg and Muthén, 2018).

Bayesian methods offer several options to deal with these challenges. Such methods usually have great flexibility in handling increases in model complexity as well as small sample constraints (McNeish, 2019), but more importantly, they offer several regularizing priors to obtain sparsity in the number of selected variables (for a large overview of regularizing priors, see Van Erp et al., 2019). Regularizing priors are priors that are designed to handle the “nearly black” vector of regression coefficients (usually their shape consists of an increasing concentration of the density around zero while maintaining heavy tails; e.g., Bhadra et al., 2016; Carvalho et al., 2010; Piironen and Vehtari, 2017). As defined by Bhadra et al. (2019), a “nearly black” vector is a vector of coefficients that has a large proportion of its elements equal (or close) to zero, while a minority of elements are greater (or less) than zero. Nevertheless, each regularizing prior has its respective strengths and weaknesses. For this reason, it is important to conduct a sensitivity analysis (see Van Erp et al., 2018). The fact that the performance of each of these priors depends on the data conditions and the type of model used presents an additional issue. For example, Zwet and Gelman (2022) showed that a low signal-to-noise ratio (SNR) often leads to highly biased estimates. Meanwhile, discussions that warn about the limitations of heavy-tailed priors and Lasso priors in logistic regression models have been disseminated throughout the Bayesian literature (Gelman et al., 2008; Piironen & Vehtari, 2015, 2017; Bhadra et al., 2019; Ghosh, 2019).

In this paper, we investigate the properties of regularizing priors in providing sparse estimation in the framework of multilevel DLVM, particularly when the number of individuals (*N*), the number of time points (*T*), the number of predictors (*P*), and the proportion of zero vs. non-zero effects vary, and when the SNR is low. To further examine the behavior of the tails of prior distributions, we conduct a prior sensitivity analysis.

## Bayesian regularization for multilevel DLVM

Although many regularizing priors have been developed to account for sparsity (Van Erp et al., 2019), we will focus on a representative set of priors which are often used in the context of latent variables models: the Bayesian Lasso (B-Lasso; Casella et al., 2010; Park and Casella, 2008), which is a standard regularizing prior; the adaptive Bayesian spike-and-slab Lasso prior (aBSS-Lasso; Brandt et al., 2018), which is a two-component regularizing prior; the regularized horseshoe prior (reg. HS; Piironen and Vehtari, 2017), which is a one-group regularizing prior (for a discussion of these different types of priors see, e.g., Bhadra et al., 2017); and the ridge prior (e.g., Van Erp et al., 2019). Though the performance of these priors has been explored for other models, little is known of their behavior for multilevel DLVM with ILD.

### Bayesian regularization

The B-Lasso prior can be seen as the Bayesian counterpart to the frequentist Lasso (Park & Casella, 2008). Using this prior allows for the effective handling of parameter uncertainty, simplifying issues like variance estimation and penalty parameter selection. A recent extension to this prior is the aBSS-Lasso (Brandt et al., 2018). Initially, spike-and-slab priors use a zero-point mass and a normal distribution mixture (Ishwaran & Rao, 2005; Kuo & Mallick, 1998). Instead of using the normal distribution, the aBSS-Lasso prior uses a mixture of Laplace distributions with adaptive penalization for each parameter subject to shrinkage. Another alternative choice of prior is the regularized horseshoe (reg. HS), which is an improved version of the horseshoe prior (Carvalho et al., 2009, 2010). In contrast to the standard horseshoe prior, the reg. HS offers computational stability and flexibility by allowing adjustable shrinkage and modification of the prior’s tails.^1^ Finally, we propose a ridge prior, which is the Bayesian counterpart of the frequentist ridge estimation, and differs from the B-Lasso prior by using a normal distribution centered on zero instead of a double exponential distribution (Van Erp et al., 2019). We argue that the ridge prior offers advantages for logistic models due to their lighter Gaussian tails, which offer more appealing properties in comparison to distributions with Cauchy-like tails (e.g., Gelman et al., 2008; Ghosh, 2019; Ghosh et al., 2018).

### The case of DLVM with ILD

Researchers often prefer models with single-level data structures in order to demonstrate the strength of their newly designed regularizing prior (e.g., Brandt et al., 2018). Consequently, it is often suitable to use ILD as an empirical example of application (e.g., Nagel et al., 2024). Hence, simulation studies using multilevel DLVM are rare. As a result, it is currently unknown what the performance of Bayesian regularization methods is when they are applied to such frameworks.

## The problem of overfitting

Overfitting occurs when a model learns the noise in the training data, resulting in poor generalization to new data (Hastie et al., 2009). Regularization methods have been developed to avoid this problem and achieve sparse estimation (Fan & Li, 2001). Their Bayesian counterparts and extensions share the same expectations (Park & Casella, 2008; Bhadra et al., 2016).

In structural equation modeling (SEM) literature, for example, Bayesian regularization of latent variables has successfully addressed overfitting, with approaches like aBSS-Lasso and B-Lasso demonstrating promising results (Brandt et al., 2018; Feng et al., 2017). Similarly, in moderated nonlinear factor analysis frameworks, regularizing priors have been used to select relevant differential item functioning and to shrink less influential parameters, with aBSS-Lasso outperforming other priors (Chen et al., 2022; Brandt et al., 2020). Note that these studies used small effects, which are generally consistent with latent variables. In addition, they tended to rely on large sample sizes, which are not always plausible for empirical studies.

However, under some conditions, regularization methods may prove ineffective due to challenges in selecting appropriate priors. For example, several claims have been highlighted about the tendency of Lassos to shrink large effect sizes (Van Erp et al., 2019; Bhadra et al., 2019). In such cases, regularizing priors with heavy tails, such as the horseshoe prior or the spike-and-slab normal, is required (see also Bhadra et al., 2016; Ishwaran and Rao, 2005; Kuo and Mallick, 1998). On the other hand, these priors may not be appropriate in logistic models, for which coefficients are more difficult to estimate and interpret than for linear models. For example, as argued by Ghosh et al. (2018), data separation – which occurs when each value of the outcome variable (e.g., infected vs. not infected patients in the case of binary data) is determined by specific non-overlapping ranges of values of the covariates (e.g., age, gender, previously recorded infections, etc.) – implies challenges in sampling the posterior distribution. In such cases, the authors demonstrated that heavy-tailed priors like Cauchy priors can lead to infinite posterior means (for further discussion on regularizing priors, see also Ghosh, 2019). As an alternative, they suggested using Student-*t* priors and normal priors with lighter tails, which will result in finite posterior means even if data separation occurs. Note that using Lasso priors typically will not solve this problem due to their overshrinkage properties, which may lead to an inflation of type I error rates (e.g., Gelman et al., 2008).

There are also some claims that overfitting per se is not necessarily a problem. For example, in the context of deep neural networks, overfitting can work well, and the inclusion of additional, possibly irrelevant, predictors can even be beneficial (Bartlett et al., 2020). In Gaussian graphical models using psychological constructs, Williams and Rodriguez (2022) argued that allowing models to overfit can be beneficial. Non-regularized methods can be used to reduce type I errors and improve predictive accuracy, although they require larger sample sizes to mitigate multicollinearity. Going back to the context of Bayesian regularization, any priors that are centered to zero could arguably be considered a regularization approach, even if no amount of shrinkage is specified (for an overview see Gelman et al., 2008). That being said, the most important point would be to question the informativeness of the priors used (see also Zwet and Gelman, 2022).

## Aims of the article

In this paper, we use two-level Markov switching (vector) autoregressive models as a special case of the Nonlinear Dynamic Latent Class-SEM framework (NDLC-SEM; Kelava and Brandt, 2019; Kelava et al., 2022). Our main concern is the identification of the predictors of the latent classes through which we will compare the Bayesian regularization methods previously discussed. We conduct two simulation studies. For each prior, we examine the convergence properties of the sampling method, the average posterior means, the power rates, and type I error rates. The first simulation study focuses on time-invariant transition probabilities, varying data conditions such as sample size (*N*), number of measurement occasions (*T*) and the number of predictors (*P*), and zero-coefficient rates. More specifically, the selected numbers of individuals were $N= 50, 75, 100$; the selected number of time points were $T=10, 25, 50$; the numbers of predictors were $P = 5, 15, 25$; and the zero-coefficient rates were varied between 50% and 75%. The second study considers the case with time-varying transition probabilities. More precisely, we added an intra-individual predictor that predicts the discrete latent states (a similar procedure can be found in Kelava et al., 2022). Here, we varied the number of time points from $T = 30,40,50,60,70$ and maintained the number of individuals at $N = 100$. We further maintained the number of predictors at $P = 15$, which included: (i) seven time-invariant predictors, four of which have zero coefficients; (ii) one time-varying predictor with nonzero coefficient; and (iii) seven interaction effects, three of which were zeros. We additionally conducted a prior sensitivity analysis through an empirical application to highlight the impact of the choice of different hyperparameters in the tails’ behavior of the regularizing priors.

The remainder of this paper is structured as follows: In the next section, we will describe the general framework from which the data were drawn in the later simulation studies. Second, we will present the prior distributions used in this article, as well as the reparametrization procedure needed for our sampling method. Third, we will provide a description of the designs of the simulation studies. Fourth, we present the results of our simulation studies in the light of convergence rates, precision of the sampling method, bias and accuracy of the estimates, and sparsity of the priors describing the type I error rates and the null detection rates. Finally, we will discuss the contribution of our results to the existing literature.

## The two-level Markov switching autoregressive model

In this study, we propose a two-level Markov switching autoregressive model of order 1 (MSAR(1)) as a representative framework of multilevel DLVMs.^2^ It can be viewed as a simplified version of the NDLC-SEM model. The NDLC-SEM framework (Kelava & Brandt, 2019; Kelava et al., 2022; Andriamiarana et al., 2023) is a general model that combines features of the DSEM and the Dynamic Latent Class framework (DLCA; see Asparouhov et al., 2017). It consists of three sub-models: (i) the within-level model, which contains a measurement model and a structural model that describes the trajectories of latent continuous states as a function of latent discrete time-dependent classes; (ii) the between-level model, which contains a measurement model and a structural model for the stable inter-individual differences; (iii) a Markov switching process that predicts latent class memberships of the individuals. Empirical applications of such models can be found, for example, in Flückiger et al. (2022) or Kelava et al. (2022).

### The within-level model

For each individual $i=1,\dots ,N$ and for each time point $t=1,\dots ,T$, let $\pmb {Y}_{it}$ be a $(J_1\times 1)$-vector representing the within-level (repeated measures) observed variable, where $J_1$ is the number of within-level observed items that measure the $(K\times 1)$-vector $\pmb {\eta }_{yit}$ of within-level latent factors. Additionally, let $\pmb {Z}_{it}$ be a $(L\times 1)$-vector representing another set of within-level observed items that measure a scalar latent time-dependent covariate $\eta _{zit}$. The within-level of the two-level MSAR(1) model is written as follows:

1 $$\begin{aligned} \pmb {Y}_{it}=&\, \pmb {\Lambda }_y \pmb {\eta }_{yit} + \pmb {u}_{yit} \end{aligned}$$

2 $$\begin{aligned} \pmb {Z}_{it} =&\, \pmb {\Lambda }_z \eta _{zit} + \pmb {u}_{zit} \end{aligned}$$

3 $$\begin{aligned} \{\pmb {\eta }_{yit}|S_{it}=s\} =&\, \pmb {A}_s + \pmb {B}_s \,\pmb {\eta }_{yit-1} + \pmb {\Gamma } \eta _{zit}+ \pmb {\epsilon }_{\eta _{y}its} \end{aligned}$$

Equation (1) describes a measurement model where $\pmb {\Lambda }_y$ is the $(J_1\times K)$-matrix of the factor loadings and $\pmb {u}_{yit}\sim \mathcal {N}_{J_1}(\pmb {0}_{J_1},\pmb {\Sigma }_y)$ is the $(J_1\times 1)$-vector of measurements errors.^3^ Here, $\pmb {0}_{J_1}$ and $\pmb {\Sigma }_y$ represent the $(J_1\times 1)$-vector of zeros and the diagonal $(J_1\times J_1)$-covariance matrix of the residuals $\pmb {u}_{yit}$, respectively. Equation (2) describes the measurement model that measures the latent variable $\eta _{zit}$, where $\pmb {\Lambda }_z$ is the associated $(L \times 1)$-vector of factor loadings. We assume that $\eta _{zit}\sim \mathcal {N}(0,\sigma _{\eta _z})$ is centered and normally distributed. We also assume that the error terms $\pmb {u}_{zit}\sim \mathcal {N}_L(\pmb {0}_L,\pmb {\Sigma }_z)$, where $\pmb {0}_L$ is a $(L\times 1)$-vector of zeros and $\pmb {\Sigma }_z$ is a diagonal $(L\times L)$-covariance matrix of measurement errors. Equation (3) describes the structural model of the latent continuous state vector $\pmb {\eta }_{yit}$ given a discrete latent class membership $S_{it}=s$ of individual *i* at time *t*. $\pmb {A}_s$ is a state-specific $(K\times 1)$-vector of latent intercepts. We denote by $\pmb {B}_s$, the state-specific $(K\times K)$-matrix of autoregressive coefficients, and by $\pmb {\Gamma }=(\gamma ,0,\dots ,0)^\top$, the $(K\times 1)$-vector of the latent covariate effects. The error term $\pmb {\epsilon }_{\eta _{y}it}\sim \mathcal {N}_K(\pmb {0}_K,\pmb {\Sigma }_\epsilon )$ is centered and normally distributed, where $\pmb {\Sigma }_\epsilon$ is a time-invariant covariance matrix. As a result, $\pmb {\eta }_{yit}$ follows a vector autoregressive model of order 1 or VAR(1). $\pmb {\eta }_{yit}$ changes depending on the latent discrete states $S_{it}$. Furthermore, only the first component of the latent factor $\{\pmb {\eta }_{yit}|S_{it}=s\}$ has an additional latent covariate, which is $\eta _{zit}$.

### The between-level model

Let $\pmb {X}_{i}$ be a $(J_2\times 1)$-vector representing the between-level observed items which measure *P* between-level latent factors (with $q=1,\dots ,Q$) arranged in the $(Q \times 1)$-vector $\pmb {\eta }_{xi}$. We then define the between-level model with the following measurement equation:

4 $$\begin{aligned} \pmb {X}_i = \pmb {\Lambda }_x \pmb {\eta }_{xi} + \pmb {u}_{xi} \end{aligned}$$

where $\pmb {u}_{xi} \sim \mathcal {N}_{J_2}(\pmb {0}_{J_2},\pmb {\Sigma }_x)$ represents the $(J_2\times 1)$-vector of measurement errors. Here, $\pmb {0}_{J_2}$ and $\pmb {\Sigma }_x$ represent the $(J_2\times 1)$-vector of zeros and the $(J_2\times J_2)$ covariance matrix of $\pmb {u}_{yit}$, respectively. $\pmb {\Lambda }_x$ is the $(J_2\times P)$-matrix of factor loadings. We assume that $\pmb {\eta }_{xi}\sim \mathcal {N}_P(\pmb {0}_P,\pmb {\Phi }_x)$, where $\pmb {\Phi }_x$ is the corresponding $(P\times P)$ diagonal covariance matrix.

### The Markov switching transition probabilities

We define the discrete state transition probabilities as follows:

5 $$\begin{aligned} \textrm{Pr}[S_{it} = s|S_{it-1}= s']= &  p_{its's}(\tilde{\eta }_{it}) \quad s,s' = 1,\dots ,S \end{aligned}$$

6 $$\begin{aligned} \tilde{\eta }_{it}= &  \mu _{0i}+ \mu _{1it} + \mu _{2it-1} \end{aligned}$$

7 $$\begin{aligned} \mu _{0i}= &  \beta _0 + \pmb {\beta }_x^\top \pmb {\eta }_{xi} + \epsilon _{0i} \end{aligned}$$

8 $$\begin{aligned} \mu _{1it}= &  \beta _z \eta _{zit}+\pmb {\beta }_{xz}^\top \pmb {\eta }_{xi} \eta _{zit} \end{aligned}$$

9 $$\begin{aligned} \mu _{2it-1}= &  \pmb {\beta }_y^\top \pmb {\eta }_{yit-1} +\pmb {\beta }_{xy}^\top (\pmb {\eta }_{xi}\otimes \pmb {\eta }_{yit-1})\nonumber \\ \end{aligned}$$

Equation (5) defines the transition probabilities which depend on $\tilde{\eta }_{it}$. This dependency is illustrated by the function $p_{its's}(\tilde{\eta }_{it})$ on the right-hand side. For simplicity, we will denote these transition probabilities by $p_{its's}$. *S* is the total number of latent states. In Eq. (6), $\tilde{\eta }_{it}$ summarizes the latent predictors of $p_{its's}$, with $p(\cdot )$ being, for example, a sigmoid function. In Eq. (7), we assume a random intercept ($\mu _{0i}$), where $\beta _0$ is an intercept with a fixed effect, $\pmb {\beta }_x$ is a $(Q \times 1)$-vector of fixed slopes/coefficients, and $\epsilon _{0i}\sim \mathcal {N}(0,\sigma _{\epsilon _0}^2)$ is the random effect term. Equations (8) and (9) include the time-varying latent predictors $\eta _{zit}$ and $\pmb {\eta }_{yit-1}$ as well as their respective interaction with the time-invariant latent factors. Note that in Eq. (9), $\otimes$ represents the Kronecker product which implies that the product $(\pmb {\eta }_{xi}\otimes \pmb {\eta }_{yit-1})$ gives a $(KQ\times 1)$-vector. We denote by $\pmb {\beta }^*= (\pmb {\beta }_x,\beta _z,\pmb {\beta }_{xz},\pmb {\beta }_{y},\pmb {\beta }_{xy})$ the vector which gathers the slopes associated with the transition probabilities’ predictors, where $\beta _z$ is a scalar, $\pmb {\beta }_{xz}$ is a $(Q \times 1)$-vector, $\pmb {\beta }_{y}$ is a $(K \times 1)$-vector and $\pmb {\beta }_{xy}$ is $(KQ\times 1)$-vector. For the rest of the paper, we assume that $\pmb {\beta }^*$ is a $(P \times 1)$-vector.

**Fig. 1:**
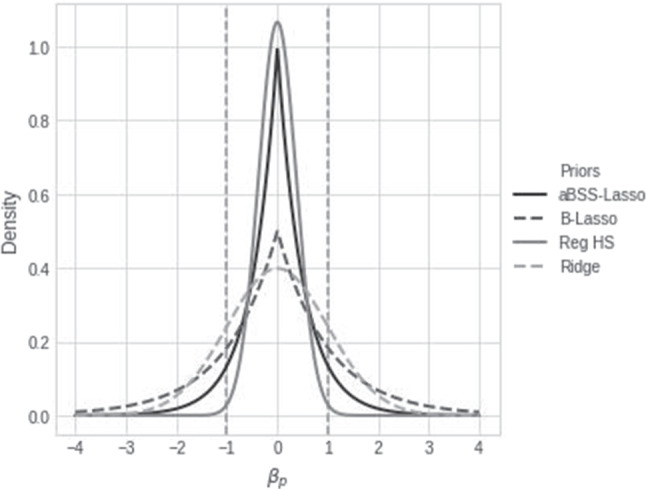
Comparison of the shapes of the density functions of the regularizing priors. These densities were drawn from Eqs. (12), (11), (13) and (14) for which we set $\sigma _eta=\lambda = 2$. For the aBSS-Lasso, we fix the probability of inclusion to $\nu = 0.5$. For the reg. HS, we fix $\tau = \frac{2}{\sqrt{50}}$ and $c^2 =4$

In our simulation studies and empirical application, we investigate the ability of regularization methods to select the relevant predictors of the latent states $S_{it}$. To this end, we assign the regularizing priors described in Fig. 1 to $\pmb {\beta }^*$ and evaluate the properties of their estimates. We suggest several scenarios that include the cases of time-varying and time-invariant transition probabilities, which can be implemented from Eqs. (7) to (9).

### Further assumptions

As a part of the data-generating process and for parameter identification, additional assumptions specific to the two-level MSAR(1) are required.

(A) As an initialization for $t=1$, we assume that each individual *i* starts the experiment with $S_{i1}=1$.^4^
(B) We assume that for each individual *i* the latent states are distributed through the so-called *Markov switching prior* so that $S_{it}\sim Multinomial(p_{is's}),\, s,s' = 1,\dots ,S$. Throughout this paper, we fix the number of latent states to $S=2$. Therefore, we assume a binomial distribution $S_{it} \sim Bin(p_{iss'}), s,s' = 1,2$ for each individual and time point (for further explanations of the Markov switching prior see Frühwirth-Schnatter, 2001).
(C) We assume that $p_{it11} = \dfrac{\exp (\tilde{\eta }_{it})}{1+\exp (\tilde{\eta }_{it})}$, which is the probability to stay in the first latent discrete state, and $p_{it12}=0.05$, which is the probability to move from state $S=2$ to state $S=1$.^5^
(D) For the loading factors, we assume that $\pmb {\Lambda }_y= {\textbf {block-Diag}}$ $(\pmb {\lambda }_{y1},\dots ,\pmb {\lambda }_{yK})$, where $\pmb {\lambda }_{yk}=(1,\lambda _{y1k},\dots ,\lambda _{yJ_{1}k})^T$ for $k=1,\dots ,K$. Moreover, we propose that $\pmb {\Lambda }_z =(1,\lambda _{z1},\dots ,\lambda _{zL})$ and $\pmb {\Lambda }_x =(1,\lambda _{x1},\dots ,\lambda _{xJ_2})$.
(E) For the variance parameters, we assume that $\pmb {\Sigma }_y$, $\pmb {\Sigma }_z$ and $\pmb {\Sigma }_x$ are diagonal matrices if they are included in the model (in the simulation studies and/or in the empirical application). Furthermore, note that $\pmb {\Sigma }_\epsilon$ is positive definite and can be decomposed following a Cholesky factorization $\pmb {\Sigma }_\epsilon = \pmb {L}_\epsilon \pmb {L}_\epsilon ^\top$ with $\pmb {L}_\epsilon = {\textbf {Diag}}(\pmb {\tau }_\epsilon ) \pmb {\Omega }_\epsilon$ being the Cholesky factor. Here, $\pmb {\tau }_\epsilon$ is the corresponding $(K\times 1)$-vector of standard deviations and $\pmb {\Omega }_\epsilon$ is a triangular matrix such that the $(K\times 1)$ correlation matrix is given by $\pmb {\Omega }_\epsilon \pmb {\Omega }_\epsilon ^\top$.

Assumptions 4.4 and 4.4 are particularly important for the simulation studies because they show how the latent states $S_{it}$ and the associated transition probabilities are constructed. We use assumptions 4.4 and 4.4 for the case of multivariate models. Assumption 4.4 mainly represents the components of the factor loadings. Assumption 4.4 introduces the Cholesky decomposition of the variance matrix, which is often recommended for computational efficiency (see Li et al., 2022). Note that these assumptions are not directly relevant in the case of univariate models.

## Bayesian estimation

As we previously presented, the two-level MSAR(1) can be considered as part of the NDLC-SEM framework. Since Bayesian estimation methods are feasible in this framework (Kelava & Brandt, 2019; Andriamiarana et al., 2023), it is necessary to consider the choice of prior distributions. In this study, we use Stan (Carpenter et al., 2017), which offers efficiency advantages for approximating the posterior distribution. In particular, Stan’s No-U-Turns-Hamiltonian Monte Carlo (NUTS-HMC; Stan Development Team, 2024) method eliminates the random walk effect of the Metropolis algorithm (see also Neal, 1992; Gelman et al., 2013; Betancourt, 2017). Recent studies by Hecht et al. (2021); Li et al. (2022) have shown that in addition to its flexibility in handling reparameterization techniques, Stan outperforms JAGS (Plummer et al., 2003) and Mplus (Muthén and Muthén, 2017) in terms of estimation efficiency and accuracy for both simple and complex models.

Let $\pmb {\Theta }=(\pmb {\Theta }_{yzx},\pmb {\Theta }_\eta )$ be the set of all the parameters in the model, where $\pmb {\Theta }_{yzx}=(\pmb {\Lambda }_y,\pmb {\Sigma }_y,\pmb {\Lambda }_z,\pmb {\Sigma }_z,\pmb {\Lambda }_x,\pmb {\Sigma }_x)$ gathers all the factor loadings and measurement error variance in each measurement model and $\pmb {\Theta }_\eta =(\pmb {A}_s,\pmb {B}_s,\pmb {\Sigma }_\epsilon ,\gamma ,\pmb {\beta }^*,\sigma _0,p_{its's})$ gathers all parameters involved in the MSAR(1) model. We also denote by $\pmb {\omega }_{it}=(\pmb {\eta }_{xi},\eta _{zit},\pmb {\eta }_{yit})$, the set of latent variables for each individual *i* and measurement occasion *t*. The posterior distribution sampling over Stan requires users to jointly consider prior specifications and build-up model likelihood so that:

10 $$\begin{aligned} &  \prod _{i=1}^{N}\prod _{t=1}^{T} \pi (\pmb {\Theta },\pmb {\omega }_{it}|\pmb {Y}_{it},\pmb {X}_{it},\pmb {Z}_{it}) \propto \pi (\pmb {\Theta })\nonumber \\ &  \prod _{i=1}^{N}\prod _{t=1}^{T} \mathcal {L}(\pmb {Y}_{it},\pmb {Z}_{it},\pmb {X}_{it},\pmb {\omega }_{it}|\pmb {\Theta }) \end{aligned}$$

where $\pi (\pmb {\Theta })$ illustrates the prior distribution over all parameters of the models and $\mathcal {L}(\pmb {Y}_{it},\pmb {Z}_{it},\pmb {X}_{it},\pmb {\omega }_{it}|\pmb {\Theta })$ denotes the conditional likelihood of the observed data and the latent variables. In the following subsections, we formally describe $\pi (\pmb {\Theta })$ including the regularizing priors, and then describe the conditional likelihood. Note that the NUTS-HMC sampling uses the gradients of the posterior distribution to propose new draws efficiently, and therefore cannot handle discrete parameters of Markov switching models (Stan Development Team, 2024).

To address this issue, we will demonstrate how to reparametrize the model using the Hamilton (forward) filter. This involves marginalizing out the latent classes $S_{it}$ in the likelihood function to allow the sampling from the gradient (Hamilton, 2010).

### Prior specification

Let us denote by $\beta _p$ the components of $\pmb {\beta }^*$, for $p=1,\dots ,P$. To ensure sparse estimation, we propose to compare the regularizing prior distributions which will be assigned to the model coefficients related to the predictors of the transition probabilities $\beta _p$ (see Eqs.  (7), (8) and  (9)). In addition, for the remaining model parameters, we propose the use of one of the most commonly recommended choices of weakly informative priors, i.e., all parameters that we did not wish to shrink. Therefore, regularizing priors are assigned only to between-level slopes $\beta _p$. For these parameters, we propose the comparison of four prior specifications, as shown in Fig. 1: the B-Lasso prior, the aBSS-Lasso prior, the reg. HS prior, and the ridge prior.

#### The B-Lasso prior

Following Casella et al. (2010), the B-Lasso prior is defined by a scale mixture of normal distributions. In our framework, this definition implies two properties. First, we assume that $\beta _p$ is drawn from a normal prior with mean 0 and variance equal to $\sigma _\eta ^2 \tau _p^2$. We denote by $\mathcal {N}(\beta _p|0,\sigma _\eta ^2 \tau _p^2)$ the prior distribution of $\beta _p$ where $\sigma _\eta ^2$ and $\tau _p^2$ are the related hyperparameters of the variance.^6^ Second, the associated scale parameter $\tau _p^2$ follows an exponential hyperprior with density $\mathcal {E}(\tau _p|\lambda )$ for $\lambda >0$. For an easier expression of the B-Lasso prior $\pi (\beta _p|\sigma _\eta ,\tau _p,\lambda )$, integrating over the scale parameter is required so that:

$$\begin{aligned} \pi (\beta _p | \sigma _\eta ,\tau _p,\lambda ) = \int \mathcal {N}(\beta _p|0,\sigma _\eta ^2\tau _p^2) \, \mathcal {E}(\tau _p|\lambda ) \, \textrm{d}\tau _p \end{aligned}$$

It can be shown that this integral results in a Laplace prior (see also Van Erp et al., 2019). Furthermore, the flexibility of the Bayesian estimation allows us to bypass cross-validation techniques by assigning a prior to the penalty term $\lambda$. Hence, we can express the B-Lasso prior as follows:

11 $$\begin{aligned} \beta _p\sim &  Laplace (0,\frac{\sigma _\eta }{\lambda }) \nonumber \\ \lambda\sim &  \mathcal {C}^+(0,a_\lambda ) \end{aligned}$$

where $a_\lambda$ is the scale hyperparameter of the half-Cauchy prior for the penalty parameter $\lambda$. Note that while inverse-Gamma priors can be assigned to $\lambda$, we will choose half-Cauchy priors, which are more suitable for scale parameters (Gelman, 2006; Brandt et al., 2018; Feng et al., 2017).

#### The aBSS-Lasso prior

The spike-and-slab prior is mainly understood as a discrete mixture of normal distributions (Kuo & Mallick, 1998; Ishwaran & Rao, 2005). Hence, we have

$$\begin{aligned} \pi (\beta _p) = \nu _p \mathcal {N}(\beta _p|0,\sigma _{\eta }^2) +(1-\nu _p) \mathcal {N}(\beta _p|0,c) \end{aligned}$$

where $\nu _p$ is the Dirac measure and *c* is a small value (here, we choose $c=0$ which implies that we have 0 in the slab instead of a normal distribution $\mathcal {N}(\beta _p|0,c)$, see Brandt et al., 2018). Here, $\nu _p$ is specific to each $\beta _p$. This implies a computational burden, as we would have *P* different mixtures of distributions. To solve this issue, we can assume that $\nu _p$ follows a Beta distribution. Furthermore, a spike-and-slab Lasso prior can be implemented by substituting the normal distribution (the spike) with a Laplace distribution $Laplace (0,\frac{\sigma _\eta }{\lambda })$, where $\lambda$ is the penalty parameter. Note that the adaptive feature is introduced by adapting a specific penalty parameter $\lambda _p$ instead of keeping a fixed penalty $\lambda$ for each coefficient $\beta _p$ (for further details, see Brandt et al., 2018). Taking all of these specifications into account, we can formulate the aBSS-Lasso prior as follows:

12 $$\begin{aligned} \beta _p= &  \bar{\beta _p} \nu _p \nonumber \\ \bar{\beta _p}\sim &  Laplace (0,\frac{\sigma _\eta }{\lambda _p})\nonumber \\ \nu _p\sim &  Beta (a, b) \\ \lambda _p\sim &  \mathcal {C}^+(0,a_\lambda )\nonumber \end{aligned}$$

As for the B-Lasso prior, we also assign a half-Cauchy prior for each penalty parameter.

#### The reg. HS prior

Let $\lambda _p$ and $\tau$ be the local and global shrinkage parameters, respectively. Piironen and Vehtari (2017) proposed that the reg. HS prior can be expressed by the following hierarchical prior:

13 $$\begin{aligned} \beta _p\sim &  \mathcal {N}(0,\tilde{\lambda }_p^2 \tau ^2), \tilde{\lambda }_p = \sqrt{\frac{c^2 \lambda _p^2}{c^2 + \tau ^2 \lambda _p^2}} \nonumber \\ \lambda _p\sim &  \mathcal {C}^+(0,1) \nonumber \\ \tau\sim &  t _{\nu _0}^+(0,\tau _0^2), \tau _0 = \frac{P_0}{P-P_0} \frac{\sigma _\eta }{\sqrt{NT}} \\ c^2\sim &  I\Gamma (\frac{\nu _c}{2},\frac{\nu _c s_c^2}{2})\nonumber \end{aligned}$$

where:

- $P_0$ is the prior guess on the effective number of non-zero coefficients. In this study, we specify a prior guess of 50% of non-zero coefficients, i.e. $P_0=P/2$.
- $\tau _0$ is the hyperparameter scale of $\tau$’s prior^7^which incorporates the information of $P_0$, and $t^+_{\nu _0}$ is the half Student distribution with $\nu _0$ degrees of freedom (for example, if $\nu _0=1$ then we obtain a half-Cauchy distribution).
- $c^2$ represents the variance of a Gaussian slab to which Eq. (13) approximates when the values of $\beta _p$ are large. Note that when $\beta _p$ is close to zero, the reg. HS approaches the original HS. We assume that $\sigma _\eta =\frac{\pi }{\sqrt{3}}$, which implies that $\tau _0=\frac{\pi }{\sqrt{3NT}}$.
- The inverse Gamma ($I\Gamma$) prior on $c^2$ implies that when the $\beta _p$s take large values, the reg. HS prior approaches a Student distribution slab $t _{\nu _c}(0,s_c^2)$, where $\nu _c$ is the degrees of freedom of the Student distribution and *s* is the corresponding scale parameter. By taking $\nu =1$ and $s^2=1$, we obtain a Cauchy slab prior.

We initially chose the values $\nu =s^2=1$ in the simulation studies and in the empirical study. In order to check the sensitivity of a change in the hyperparameter, we add the hyperparameter values $\nu =s^2=4$ into the comparison of priors.

#### The ridge prior

The ridge prior is the Bayesian counterpart of the $l_2$ norm used in the frequentist regularization paradigm (e.g., Van Erp et al., 2019). By assigning this prior to the $\beta _p$ coefficients, we can describe the ridge prior as follows:

14 $$\begin{aligned} \beta _p\sim \mathcal {N}(0,\frac{\sigma _\eta ^2}{\lambda }), \, \lambda >0 \end{aligned}$$

There are several ways to tune this prior. In our case, we tuned it in such a way that there is no extreme amount of imposed shrinkage. In particular, we set the penalty hyperparameter to $\lambda =1$ (see Fig. 1). This setting suggests a regularization approach without extreme amounts of shrinkage.

**Table 1:** Description of the different prior distributions for all the model parameters, where $\beta _p$ denotes the slope parameter for $p=1,\dots ,P$ . *g*(.) denotes the prior distribution for the slope parameters that are described in Table 2

	Prior distributions	
	Between level	Within level
Variances and Std. deviations	$\sigma _x \sim \mathcal {C}^+(0,2.5)$	$\sigma _y\sim \mathcal {C}^+(0,2.5)$
	$\phi _x \sim \mathcal {C}^+(0,2.5)$	$\tau _{\epsilon k} \sim \mathcal {C}^+(0,2.5)$
	$\sigma _{\epsilon _0} \sim \mathcal {C}^+(0,2.5)$	$\pmb {\Omega }_\epsilon \pmb {\Omega }_\epsilon ^\top \sim LKJ(4.0)$
Intercepts and slopes	$\beta _0 \sim \mathcal {N}^+(0,10)$	$$\begin{array}{lll} a_{s}= 0 & \text {if} & s=1 \\ a_{s}\sim \mathcal {N}^+(0,10) & \text {if} & s=2 \end{array}$$
	$\beta _p \sim g(.)$	$$\begin{array}{lll} b_{s}= 0 & \text {if} & s=1 \\ b_{s}\sim \mathcal {N}(0,1) & \text {if}& s=2 \\ \end{array}$$
Factor Loadings	$\lambda _x \sim \mathcal {N}^+(0,1)$	$\lambda _y \sim \mathcal {N}^+(0,1)$

Note that $\lambda _x \text { and }\lambda _y$ describe free elements (not equal to 0 or 1) of $\pmb {\Lambda }_x\text { and }\pmb {\Lambda }_y$, respectively. $\sigma _x,\phi _x\text { and }\sigma _y$ denote the diagonal elements of $\pmb {\Sigma }_x,\pmb {\Phi }_x\text { and }\pmb {\Sigma }_y$, respectively. $\tau _{\epsilon k}$ is the $k^{th}$ element of $\pmb {\tau }_\epsilon$. $\mathcal {C}^+$ is the half Cauchy distribution. $\mathcal {N}^+$ is the half normal distribution. *LKJ* is the Lewandowski–Kurowicka–Joe distribution

#### Priors for the rest of the parameters

Weakly informative priors are assigned to the within-level coefficients and the variance parameters. Prior distributions assigned to the variances and the standard deviations are presented in the first row of Table 1. We follow recommendations from Gelman (2006) and propose half-Cauchy priors $\mathcal {C}^+(0,a_\sigma )$ for standard deviations. Since $\pmb {\Sigma }_\epsilon$ can be decomposed using factor distribution (see assumptions 4.4), we can implement the Lewandowski–Kurowicka–Joe correlation (LKJCorr) prior distribution for the correlation matrix $\pmb {\Omega }_\epsilon \pmb {\Omega }_\epsilon ^\top$ and half-Cauchy priors for the elements of $\pmb {\tau }_\epsilon$ (Stan Development Team, 2024, The implementation in Stan is fully detailed in the Stan’s User guide on Chapter 1, section 1.13 and 1.15). As the corresponding hyperparameter (which is greater than or equal to 1) approaches 1, the density becomes more concentrated around $\pmb {I}_K$; the greater it is compared to 1, the higher the correlations. The second row of Table 1 shows the prior distributions that were assigned for the intercepts and slopes. On the within-level, when the latent state was $S=2$, a half normal prior $\mathcal {N}^+(0,10)$ was specified for the intercept, while a $Beta(a_b,b_b)$ was given to the slope if the model is univariate. For the consideration of multivariate models, we use standard normal priors. When the latent state is $S=1$, the autoregressive coefficient and the intercept will be set to zero. The third row of the table shows that standard normal priors are assigned to the factor loadings.

### Likelihood function

We write the model likelihood as follows:

15 $$\begin{aligned} &  \prod _{i=1}^{N}\prod _{t=1}^{T} \mathcal {L}(\pmb {Y}_{it},\pmb {Z}_{it},\pmb {X}_{it},\pmb {\omega }_{it}|\pmb {\Theta }_\eta ) \nonumber \\ &  = \mathcal {L}_\eta \prod _{i=1}^{N} \prod _{t=1}^{T} f(\pmb {\eta }_{xi},\eta _{zit}|\pmb {\Theta }_\eta ) \mathcal {L}_{yzx}(\pmb {Y}_{it},\pmb {Z}_{it},\pmb {X}_{it}|\pmb {\Theta }_{yzx}) \end{aligned}$$

where we define $\mathcal {L}_{\pmb {\eta }} = \prod _{i=1}^{N} \prod _{t=1}^{T} f(\pmb {\eta }_{yit}|\pmb {\Theta }_\eta )$ as the likelihood contribution of the MSAR(1) model with time-varying (or time-invariant) transition probabilities.^8^ Here, we denote by $\mathcal {L}_{yzx}(.)$, the likelihood contribution of the measurement models on both the intra-individual and the inter-individual level.

Note that HMC sampling uses the information provided by the gradient of the posterior distribution. As a consequence, in order to sample over discrete parameters, we must compute derivatives with respect to discrete variables, which is not feasible. An appropriate reparametrization is required to avoid sampling from the discrete latent class $S_{it}$ in Stan. For the implementation of the MSAR(1), the likelihood contribution of $\pmb {\eta }_{yit}$ must be obtained by marginalizing over the states $S_{it}$. This can be achieved by using the so-called Hamilton filter (Rabiner, 1989; Hamilton, 2010; Kohlschein, 2006; Manual, 2017; Hamilton, 2020; Savage, 2018).

**Table 2:** Prior specifications for the slopes parameters of the transition probabilities

	Hierarchies			
Priors	(1)	(2)	(3)	(4)
Ridge	$\beta _{p} \sim \mathcal {N}(0,1)$	$\lambda =1$		
B-Lasso	$\beta _p\sim Laplace (0,\frac{\sigma _0}{\lambda })$	$\lambda \sim \mathcal {C}^+(0,2.5)$		
ABSS-Lasso	$\beta _p = \beta _p^{*} \nu _p$	$\beta _p^{*}\sim Laplace (0,\frac{\sigma _0}{\lambda _p})$	$\nu _p \sim Beta (\frac{1}{2},\frac{1}{2})$	$\lambda _p \sim \mathcal {C}^+(0,2.5)$
reg. HS	$\beta _p \sim \mathcal {N}(0,\tilde{\lambda }_p^2 \tau ^2)$	$\lambda _p \sim \mathcal {C}^+(0,1)$	$\tau \sim \mathcal {C}^+(0,\tau _0)$	$c^2 \sim I\Gamma (\frac{1}{2},\frac{1}{2})$
	$\tilde{\lambda }_p = \sqrt{\frac{c^2 \lambda _p^2}{c^2 + \tau ^2 \lambda _p^2}}$		$\tau _0 = \frac{\pi }{\sqrt{N T}}$	

For a given individual *i*, let us denote by $\pmb {W}_{it} = \{\pmb {\eta }_{yi1},\dots ,\pmb {\eta }_{yit}\}$, the trajectory of the latent factor $\pmb {\eta }_{yit}$ up to a given measurement occasion *t*. In order to perform an inference over $S_{it}$’s values which are not observed, we must rely on the following probability distribution:

16 $$\begin{aligned} \xi _{sit} =P(S_{it}=s|\pmb {W}_{it};\pmb {\Theta }_\eta ). \end{aligned}$$

Note that for $s=1,2$ we must have $\sum _{s=1}^{2}\xi _{sit} = 1$. Then, by computing this basic conditional probability, we can deduce the following expressions:

17 $$\begin{aligned} \xi _{sit}&= \dfrac{f(\pmb {\eta }_{yit}|S_{it}=s,\pmb {W}_{it-1};\pmb {\Theta }_\eta ) P(S_{it}=s|\pmb {W}_{it-1};\pmb {\Theta }_\eta )}{f(\pmb {\eta }_{yit}|\pmb {W}_{it-1};\pmb {\Theta }_\eta )} \nonumber \\&= \dfrac{f(\pmb {\eta }_{yit}|S_{it}=s,\pmb {W}_{it-1};\pmb {\Theta }_\eta ) \sum _{s'=1}^{2} P(S_{it}=s,S_{it-1}=s'|\pmb {W}_{it-1};\pmb {\Theta }_\eta )}{f(\pmb {\eta }_{yit}|\pmb {W}_{it-1};\pmb {\Theta }_\eta )} \nonumber \\&=\dfrac{f(\pmb {\eta }_{yit}|S_{it}=s,\pmb {W}_{it-1};\pmb {\Theta }_\eta ) \sum _{s'=1}^{2} P(S_{it}=s|S_{it-1}=s') P(S_{it-1}=s'|\pmb {W}_{it-1};\pmb {\Theta }_\eta )}{f(\pmb {\eta }_{yit}|\pmb {W}_{it-1};\pmb {\Theta }_\eta )} \nonumber \\&=\dfrac{f(\pmb {\eta }_{yit}|S_{it}=s,\pmb {W}_{it-1};\pmb {\Theta }_\eta ) \sum _{s'=1}^{2} p_{its's} \ \xi _{s'i(t-1)}}{f(\pmb {\eta }_{yit}|\pmb {W}_{it-1};\pmb {\Theta }_\eta )} \end{aligned}$$

Since $\xi _{sit}$ is a probability, we must have $\sum _{s=1}^2\xi _{sit} =1$. Consequently, we can deduce that the conditional density of $\pmb {\eta }_{yit}$, which is the denominator in Eq. (17), is given by:

18 $$\begin{aligned} f(\pmb {\eta }_{yit}|\pmb {W}_{it-1};\pmb {\Theta }_\eta )= &  \sum _{s=1}^{2} f(\pmb {\eta }_{yit}|S_{it}=s,\pmb {W}_{it-1};\pmb {\Theta }_\eta )\nonumber \\ &  \times \sum _{s'=1}^{2}p_{its's} \ \xi _{s'i(t-1)} \end{aligned}$$

Equation (18) represents the likelihood contribution across all individuals *N* and time points *T*. Hence, the conditional log-likelihood of the observed data is written as follows:

19 $$\begin{aligned} \log \mathcal {L}_\eta = \sum _{i=1}^{N} \sum _{t=1}^{T} \log f(\pmb {\eta }_{yit}|\pmb {W}_{it-1};\pmb {\Theta }_\eta ) \end{aligned}$$

where $\xi _{s'i1}$ for $s'=1,2$ corresponds to the initial probabilities which must be specified to start the forward algorithm. In this study, we assumed that $\xi _{1i1}=1$ and $\xi _{2i1}=0$. This matches with the population values, since we set $S_{i1}=1$ for all individuals.

## Simulation design

In this section, we aim to compare the ridge prior, the B-Lasso prior, the aBSS-Lasso prior, and the reg. HS prior in simulation studies (see Table 2). We first describe the conditions used to generate the data. Globally, we simulated a univariate two-level MSAR(1) where $J_1=1$ (one endogenous latent variable) and $K=1$ (one latent covariate) and $J_2=P$, which implies one latent variable per observed item. We also assumed that all factor loadings are equal to 1 and that the measurement errors are equal to 0 (i.e., $\pmb {u}_{yit}=\pmb {0}_2$ and $\pmb {u}_{xi}=\pmb {0}_P$). In the following sections, we will describe how Simulation Studies 1 and 2, with time-invariant and time-varying transition probabilities, respectively, will address the two-level MSAR(1). Then, we will describe our expectations concerning these simulation studies.

**Fig. 2:**
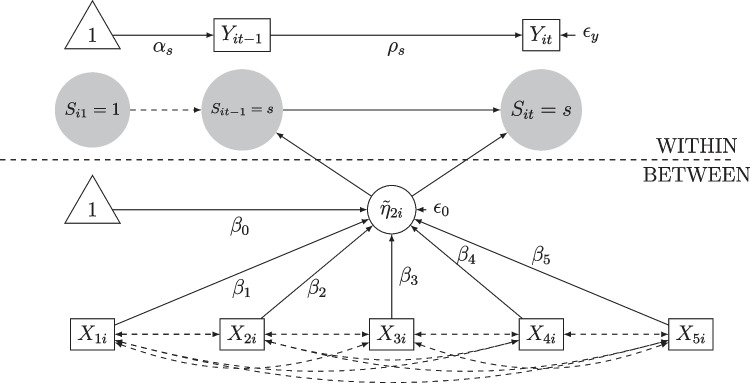
Path diagram when the number of predictors of the between-level is $p=5$

### Simulation 1: Time-invariant transition probabilities

In Simulation Study 1, we used the two-level MSAR(1) with time-invariant transition probabilities, as depicted in Fig. 2. Hence, we had $\pmb {\beta }^*=\pmb {\beta }_{x}$, which is a vector of dimension *P* and suggested that $\pmb {\mu }_{1it}$ and $\pmb {\mu }_{2it-1}$ are excluded from the model.

**Data conditions** At the between-level, we set the intercept to $\beta _0 = 3$. We set the non-zero slopes by alternating their population values between 0.5 and -0.5 (i.e., $\pmb {\beta }^* = (0.5, -0.5, \dots , 0, \dots , 0)$). We chose these values because they reflect the relatively small effect sizes that are typically found in psychological research (e.g., Flückiger et al., 2022; Heusel et al., 2023; Kelava et al., 2022). We argue that the identification of such values can be challenging.^9^ This is, for example, the case for Lasso priors, which suffer from overshrinkage issues (e.g., Bhadra et al., 2019). As part of our simulation studies, a comparison of regularizing priors in the context of multilevel DLVM with these effects would be interesting. We maintained the value of the covariate correlation coefficients at $\phi _{kl} = 0.3$ and their variances at $\phi _{kk} = 1$. At the within-level, the true parameter values, which we now express as scalars by omitting the bold notation (see Eq. (3)), are given by $A_1 = 0$, $A_2 = 3$, $B_1 = 0$, $B_2 = 0.8$, $\gamma = 0.5$, and $\sigma _\epsilon = 0.25$.

**Table 3:** Description of the data conditions in Simulation Study 1

Cases	Design conditions	Number of
		conditions
Number of individuals	$N=50,75,100$	3
Lengths of time points	$T=10,25,50$	3
No. of predictors	$P=5,15,25$	3
Proportion of zero-coefficients	$50\%,75\%$	2
Total		54

**Fig. 3:**
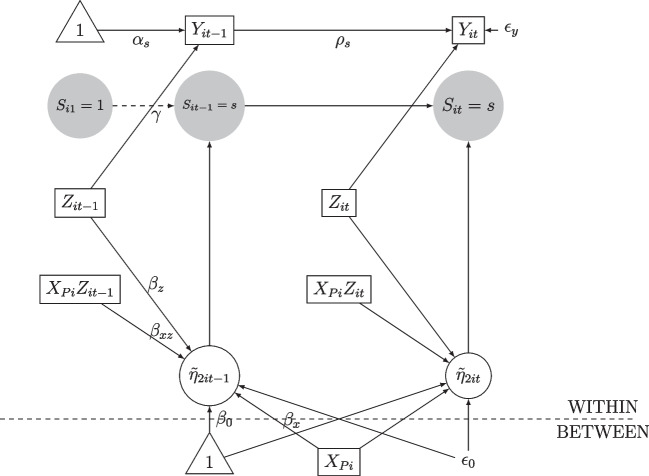
Path diagram for the case with time-varying transition probabilities with cross-level interaction effects

Table 3 outlines the data conditions under which the four prior distributions are compared. We varied the number of individuals ($N = 50, 75, 100$) and the number of time points ($T = 10, 25, 50$) over a range of values that include sample sizes (*N* and *T*) typically collected in empirical research. For example, Flückiger et al. (2022) investigated how the working alliance between patients and therapists develops over successive sessions using the NDLC-SEM framework. An initial empirical study involved $N_1=57$ patients and $T=14$ sessions. A second empirical study involved $N_1=80$ patients and $T=16$ sessions. Consequently, it would be reasonable to fix, for example, $T=10$ as a minimal condition for the length of time points (see also Andriamiarana et al., 2023; Schultzberg and Muthén, 2018). Furthermore, Kelava et al. (2022) used the NDLC-SEM framework to predict university students’ affective states. They collected ILD data that included $N=122$ students and a large number of time points ($T=50$) in daily online surveys. Therefore, it is reasonable to fix the maximum length of time points to $T=50$. We also varied the dimension of the between-level latent predictors ($P = 5, 15, 25$). Then, we implemented two schemes of “nearly black” vector of parameters (e.g., Bhadra et al., 2019), which we evaluated by varying the proportion of zeros components in $\pmb {\beta }^*$ from 50% to 75%. These are common practices for the evaluation of regularizing priors through simulation studies (e.g., Brandt et al., 2018; Piironen and Vehtari, 2017; Zhang et al., 2022).

#### Sampling

We used Stan’s HMC, which benefits from the NUTS correction (Manual, 2017; Betancourt, 2017). Four chains were used, and each chain generated 2000 iterations and 1000 warm-ups. For 200 replications and 54 conditions, the elapsed times were respectively: $\sim 15.8$ days in CPU times for the ridge prior; $\sim 23.5$ days in CPU times for the reg. HS prior; $\sim 36.8$ days for the aBSS-Lasso prior and $\sim 53$ days for the B-Lasso prior. In total, Simulation Study 1 required around 4 months and 9 days of CPU time. All the computational processes in this paper were parallelized within the bwHPC Cluster.

### Simulation 2: Time-varying transition probabilities

We extended the analysis by comparing the four prior distributions over the two-level MSAR(1) with time-varying transition probabilities. We also included between-level and within-level interaction effects. Figure 3 shows the path diagram of the model from which we generated the data. Table 4 shows the simulation conditions used to generate the data. The parameters of the transition probability slopes were given by $\pmb {\beta }_x=(0.5,-0.5,0.5,0,0,0,0)$, $\pmb {\beta }_{xz}=(0,0,0,0,0,0.5,-0.5,0.5)$, and $\beta _z=0.5$. Here, $\mu _{2it-1}$ previously described in Eq. (9) was not included in the model. The variance of the random effect was $\sigma _{\epsilon 0} = 1$, and in the autoregressive model, the effect of the covariates was $\gamma =0.5$. We used the same sampling setting as in Simulation Study 1. In total, Simulation Study 2 required around 1 month and 12 days in CPU times, with 6.2 days for the ridge prior, 6.9 days for the B-Lasso prior, 9.6 days for the reg. HS prior, and 19.2 days for the aBSS-Lasso prior.

**Table 4:** Description of the data conditions in Simulation Study 2

Cases	Design conditions	Number of
		conditions
Number of individuals	$N=100$	1
Lengths of time points	$T=30,40,50,60$	5
No. of predictors	$P=15$ including $\pmb {\eta }_{xi}\eta _{zit}$	1
Correlation of covariates	$\phi _{kl}=0.3 \ \ \ \ k\ne l$	1
Total		5

### Our expectations

In the previous subsections, we assumed that the population values for the slope parameters $\beta _p$ in the transition probabilities are within the interval $(-1,1)$. This allowed us to investigate scenarios where regularizing priors might have difficulties identifying the non-zero coefficients. We therefore formulated the following expectations for the results of our simulation studies:

1. We expect the ridge prior to outperform the other regularizing priors due to its normal distribution tails. Following the arguments of Ghosh et al. (2018), distributions with light tails are more suitable for the logistic model. As shown in Fig. 1, the regularizing priors have a very high concentration around zero compared to the normal distribution. This implies that their resulting posterior means will tend to approach zero, particularly for values within the interval $(-1,1)$.
2. We also expect the reg. HS prior, with its Cauchy-like tails, to be better suited to logistic models than the Lassos (aBSS-Lasso and the B-Lasso). This is because reg. HS priors are designed for generalized linear models (Piironen & Vehtari, 2017). We expect that the Lassos will lead to overshrinkage and inflated type I error rates, and can be outperformed by priors with Cauchy-like tails (for further explanation, see Chen et al., 2022; Gelman et al., 2008; Ghosh, 2019).
3. Let us define the signal-to-noise ratio (SNR) by the following quantity $SNR=\frac{\pmb {\beta }^{*T}\pmb {\Phi }_x \pmb {\beta }^*}{\sigma ^2_0 + \sigma ^2_s}$. This quantity computes the ratio between the contribution of the relevant variables (when $\beta _p \ne 0$) in terms of variance and the error variance. Note that when we increase *P*, the SNR increases as well. For example, for the model with time-invariant transition probabilities, $P=5$ gives $SNR\approx 0.08$; $P=15$ gives $SNR\approx 0.21$; and $P=25$ gives $SNR\approx 0.31$. For the model with time-varying transition probabilities, we have $SNR \approx 0.26$. Computing this quantity allows us to have an understanding of the quality of the estimates. Since a low SNR is associated with biased estimates (e.g., Zwet and Gelman, 2022), we expect the estimates of $\beta _p$ to be biased for each prior.

## Results of the simulation studies

### Simulation 1 results

#### Convergence and sampling precision

Convergence rates were assessed using the Gelman–Rubin *R*-*hat* statistic. If all parameters of the model had an *R*-$hat<1.1$, then we concluded that the model had converged. We computed the convergence rates over the 200 replicated data sets. We found high convergence rates for all prior distributions: The ridge prior had convergence rates greater than 99% over all data conditions, followed by the reg. HS prior with an average rate across data conditions of 99.9% and a minimum rate of 98.5%. For the aBSS-Lasso, the lowest rate across conditions was 83.5%, with an average rate of 97.3%. For the B-Lasso, the lowest rate across conditions was 70%, with a mean rate of 90.7%. The figures describing the behavior of the convergence rates across conditions (i.e., with respect to *N*, *T*, *P* and the proportion of zeros) are provided in the Supplementary materials. We only considered models that converged to compute all quantities used to analyze the properties of the estimates (in particular, the average posterior mean, bias, accuracy, coverage rates, and type I error rates).

As argued by Zitzmann and Hecht (2019), the sampling precision of Monte Carlo Markov chain (MCMC) should not be underestimated. Sampling precision rates were calculated based on a cutoff value *x* over the Effective Sample Size (ESS). The ESS represents the number of independent samples amongst all generated iterations. We counted the number of times that the ESS exceeded a chosen cutoff across replications and data conditions (i.e., when $ESS>x$). We set the cutoffs at $x=100,400,1000$. The $x=400$ threshold is the default in Mplus (Muthén and Muthén, 2017), while $x=1000$ is suggested by Zitzmann and Hecht (2019). If all parameters satisfied $ESS>x$, then we selected the replicated model and considered it to have good precision with respect to *x*. This was the basis for the calculation of the precision rates across replications. Overall, we found that the ridge prior and the reg. HS had the best sampling precision. They were followed by the aBSS-Lasso prior, which had less stable precision rates. The B-Lasso prior had the lowest precision rates. The details of the precision rates are provided in the Supplementary materials.

Note that we rarely encountered divergence transition issues throughout the simulation studies. In fact, divergence transitions, when they occurred, were associated with large *R*-*hat* and low *ESS*, which as we showed were very rare and were not accounted for in the evaluation of the finite sample properties of the estimates.

#### Posterior mean and bias

**Fig. 4:**
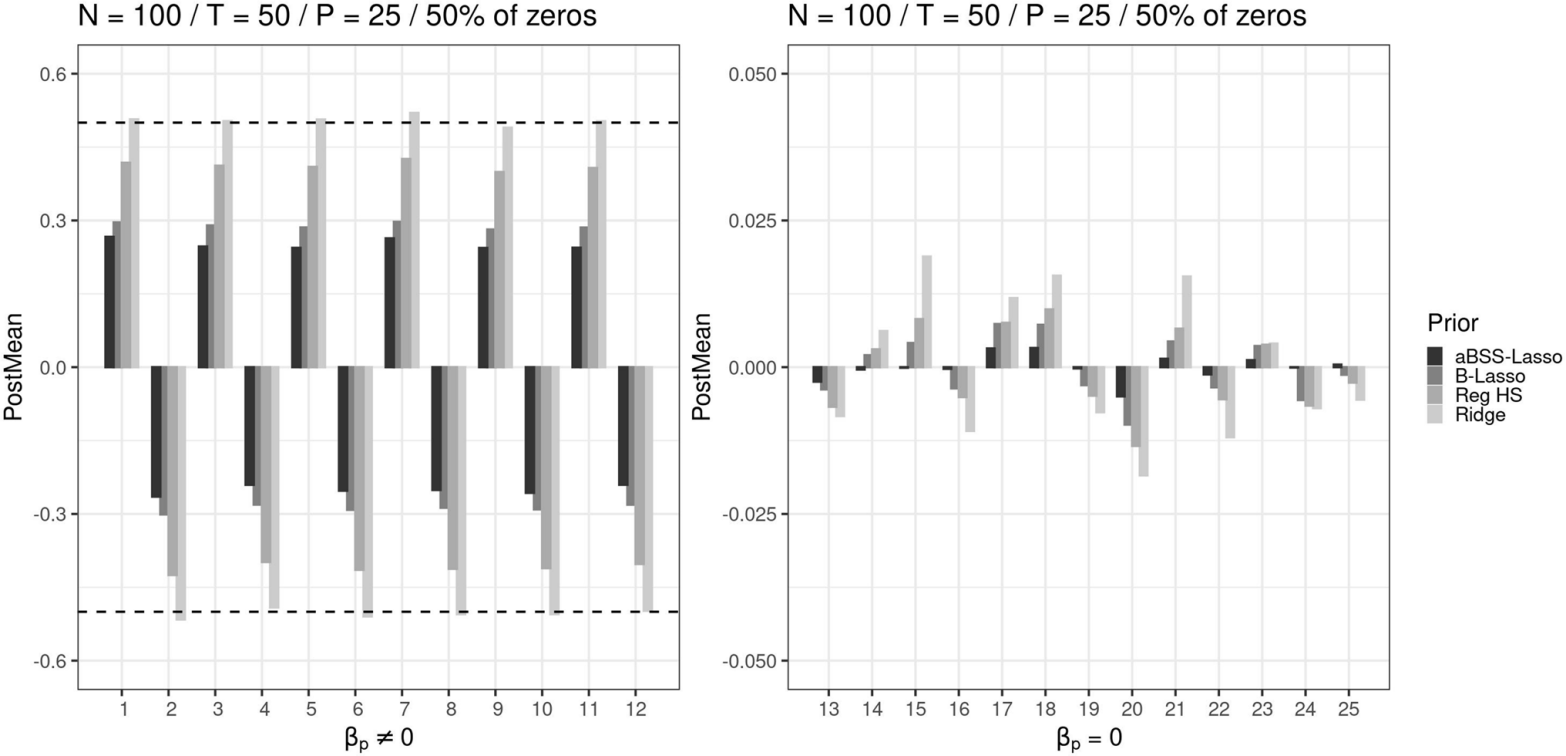
Comparison of the average posterior means for each prior distribution when $N=100, T=50, P=25$. The *left side* shows the posterior means for the 50% of the elements of the $\pmb {\beta }^*$ parameter vector which are non-zero (i.e., 12 components out of 25 of the parameter vector $\pmb {\beta }^*$ are not equal to zero). The *x*-axis corresponds to the numbered elements of the $\pmb {\beta }^*$ parameter vector. The *red dashed lines* represent the population values of the non-zero slopes (0.5 and -0.5, respectively)

The results showed that the aBSS-Lasso, the B-Lasso, and the reg. HS priors provided highly biased estimates of the non-zero coefficients compared to the ridge prior. This was not surprising, since we expected that low SNR would lead to such results. Figure 4 compares the average posterior means given by the four priors when half of $\pmb {\beta }^*$’s components are equal to 0. The left side of the figure shows the case of non-zero coefficients and the right side shows the case of the zero coefficients. We observed that the B-Lasso and aBSS-Lasso priors overshrunk the non-zero slopes. As shown in the Supplementary materials, this could even be worse with smaller *N* and *T*. The reg. HS outperformed the Lasso priors in determining the non-zero coefficients. In all conditions, the ridge prior provided the least biased estimates of the non-zero coefficients. Furthermore, we found that all priors were able to identify the zero coefficients. Although it was almost negligible (in particular, when *N* and *T* increased), we also noticed small improvements in the results when the number of zeros increased, i.e., from 50% zeros to 75% in $\pmb {\beta }^*$ (We refer the reader to the Supplementary materials for further figures on the posterior means, relative bias, absolute bias, and accuracy).

In fact, these results can easily be explained by the shape of each prior density, as shown in Fig. 1. In particular, overshrinkage issues happen because of the high concentration around zero, particularly for the aBSS-Lasso and the B-Lasso. In contrast, this issue was less problematic for the ridge and reg. HS priors because the Lasso priors are not appropriate for logistic models (Gelman et al., 2008). In particular, distributions with lighter tails, such as those of the ridge priors, are highly recommended for such models (Ghosh et al., 2018; Ghosh, 2019).

#### Power and type I error rates

We investigated the power and the type I error rate of the estimates. The power is the probability of finding a non-zero coefficient when it is truly non-zero. Type I error is the probability of identifying a non-zero coefficient when it is truly zero. To evaluate these probabilities, we used the 95% credible interval (CI) rule and thresholding rules similar to those studied by Zhang et al. (2021). Specifically, we choose the following two thresholding rules: $|\hat{\beta _p}| > 0.1$ and $|\hat{\beta _p}| > 0.15$. If $\hat{\beta _p}$ corresponds to the estimate of the non-zero coefficient $\beta _p$, then the obtained quantity corresponds to the power rates. If it instead corresponds to the estimate of the zero coefficient, then this quantity corresponds to the type I error rates. Therefore, the power rates are given by the following quantities:

$$\begin{aligned} \text {Power}(CI_{95\%})&= \frac{1}{M}\sum _{m=1}^{M} \pmb {1}\{0 \notin (l_m, u_m) | \beta _p \ne 0\}\\ \text {Power}(|\hat{\beta _p}|> 0.1)&= \frac{1}{M}\sum _{m=1}^{M} \pmb {1}\{|\hat{\beta _p}|> 0.1 | \beta _p \ne 0\}\\ \text {Power}(|\hat{\beta _p}|> 0.1)&= \frac{1}{M}\sum _{m=1}^{M} \pmb {1}\{|\hat{\beta _p}| > 0.15 | \beta _p \ne 0\} \end{aligned}$$

while the type I error rate rates are:

$$\begin{aligned} \text {Type I error}(CI_{95\%})&= \frac{1}{M}\sum _{m=1}^{M} \pmb {1}\{0 \notin (l_m, u_m) | \beta _p = 0\} \\ \text {Type I error}(|\hat{\beta _p}|> 0.1)&= \frac{1}{M}\sum _{m=1}^{M} \pmb {1}\{|\hat{\beta _p}|> 0.1 | \beta _p = 0\}\\ \text {Type I error}(|\hat{\beta _p}|> 0.15)&= \frac{1}{M}\sum _{m=1}^{M} \pmb {1}\{|\hat{\beta _p}| > 0.15 | \beta _p = 0\} \end{aligned}$$

where $l_m$ and $u_m$ are the lower and upper bounds of the $m^{th}$ replicate of 95% CI, and *M* is the number of replicates. Note that unlike the 95% CI rule, thresholding rules impose fixed intervals and therefore do not take into account the variability of estimates.

**Fig. 5:**
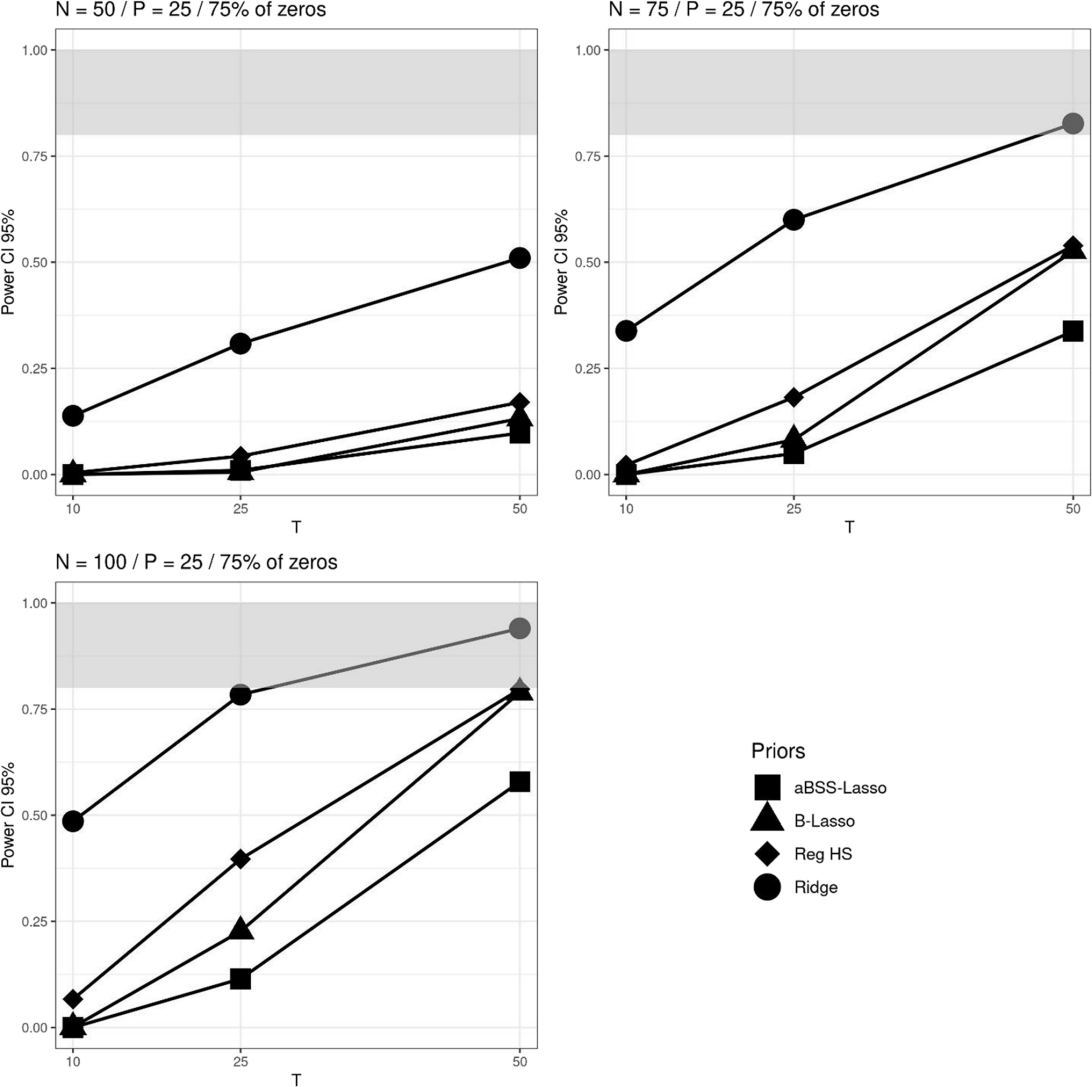
Power based on 95% credible interval (CI) across *N*, *T* and *P* for the average non-zero and zero slopes when 75% of the elements of the $\beta$ parameter vector are zeros. The *grey bands* correspond to the interval [0.8, 1]

**Fig. 6:**
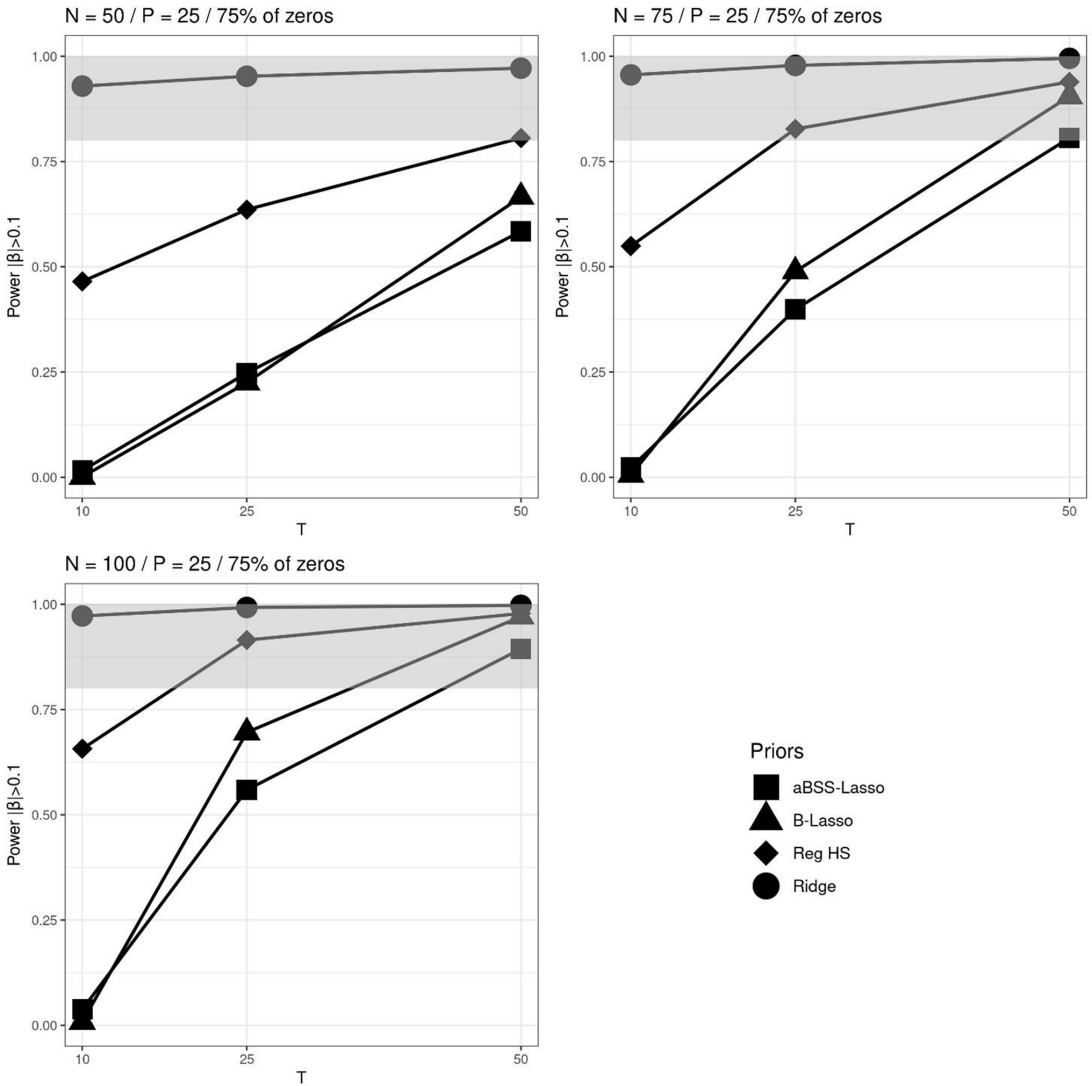
Power based on $|\beta _p|>0.1$’s thresholding rule across *N*, *T* and *P* for the average non-zero and zero slopes when 75% of the elements of the $\beta$ parameter vector are zeros. The *gray bands* correspond to the interval [0.8, 1]

**Fig. 7:**
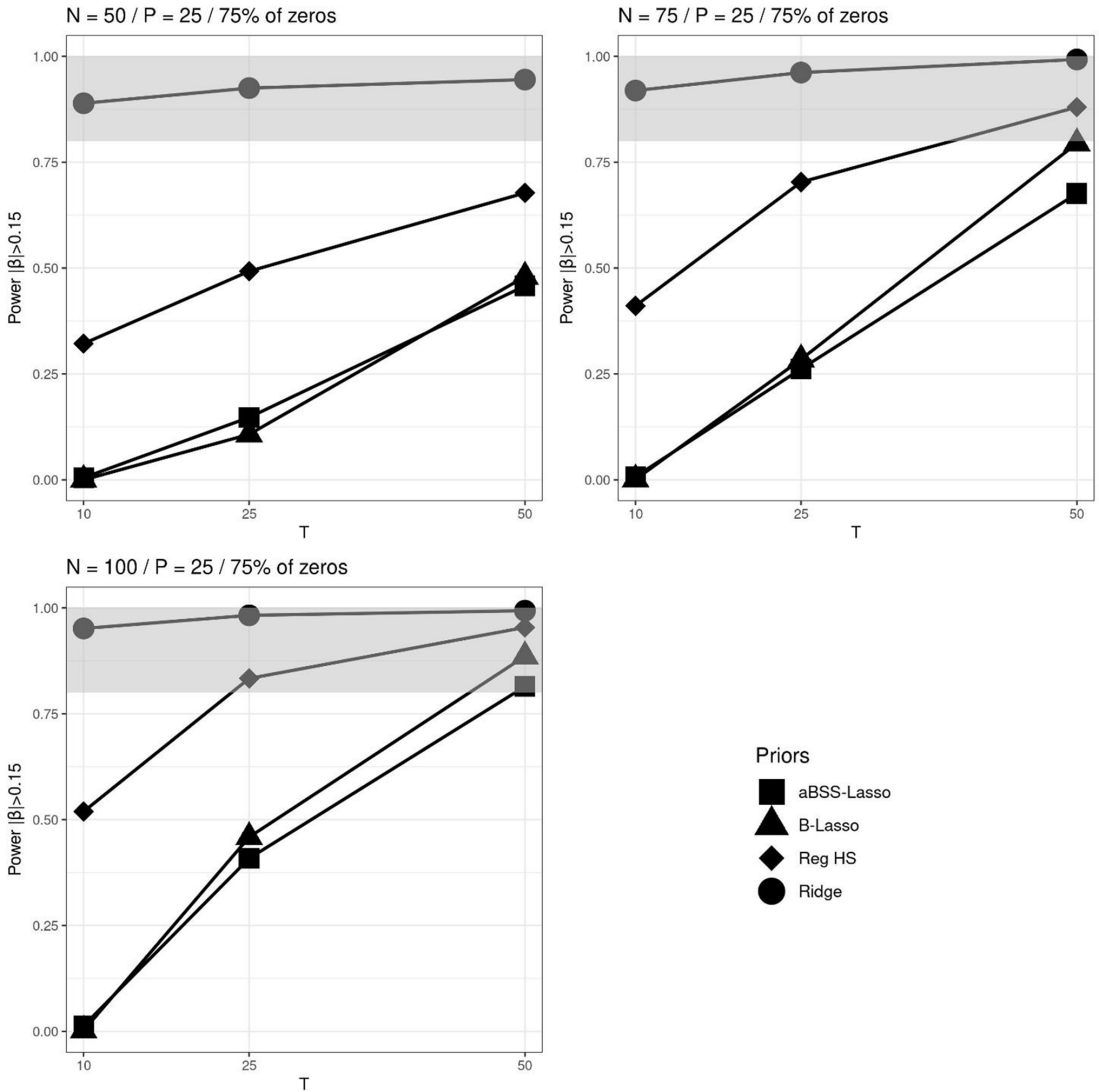
Power based on $|\beta _p|>0.15$’s thresholding rule across *N*, *T* and *P* for the average non-zero and zero slopes when 75% of the elements of the $\beta$ parameter vector are zeros. The *gray bands* correspond to the interval [0.8, 1]

Overall, the different approaches of computing the power arguably provided rather stable ranking of the regularizing priors. We particularly found that the ridge prior outperformed the other regularizing priors, and that the reg. HS prior outperformed the Lasso priors. Figures 5, 6 and 7 illustrate the comparison of the regularizing priors across the three criteria of evaluation of the power previously described. These figures focus on the cases where 75% of $\pmb {\beta }^*$’s components are zeros and $P=25$. For the power based on the 95% CI, we found that with sufficiently large *N* and *T*, the ridge prior could provide power rates greater than 0.8. In contrast, the reg. HS hardly reached this level, and the Lasso priors never achieved it. The results were slightly different for the power rates based on thresholding rules. Notably, we observed more instances in which the reg. HS had a higher power. The Lasso priors also reached the admissible power range, but only when $N=100$ and $T=50$.

**Fig. 8:**
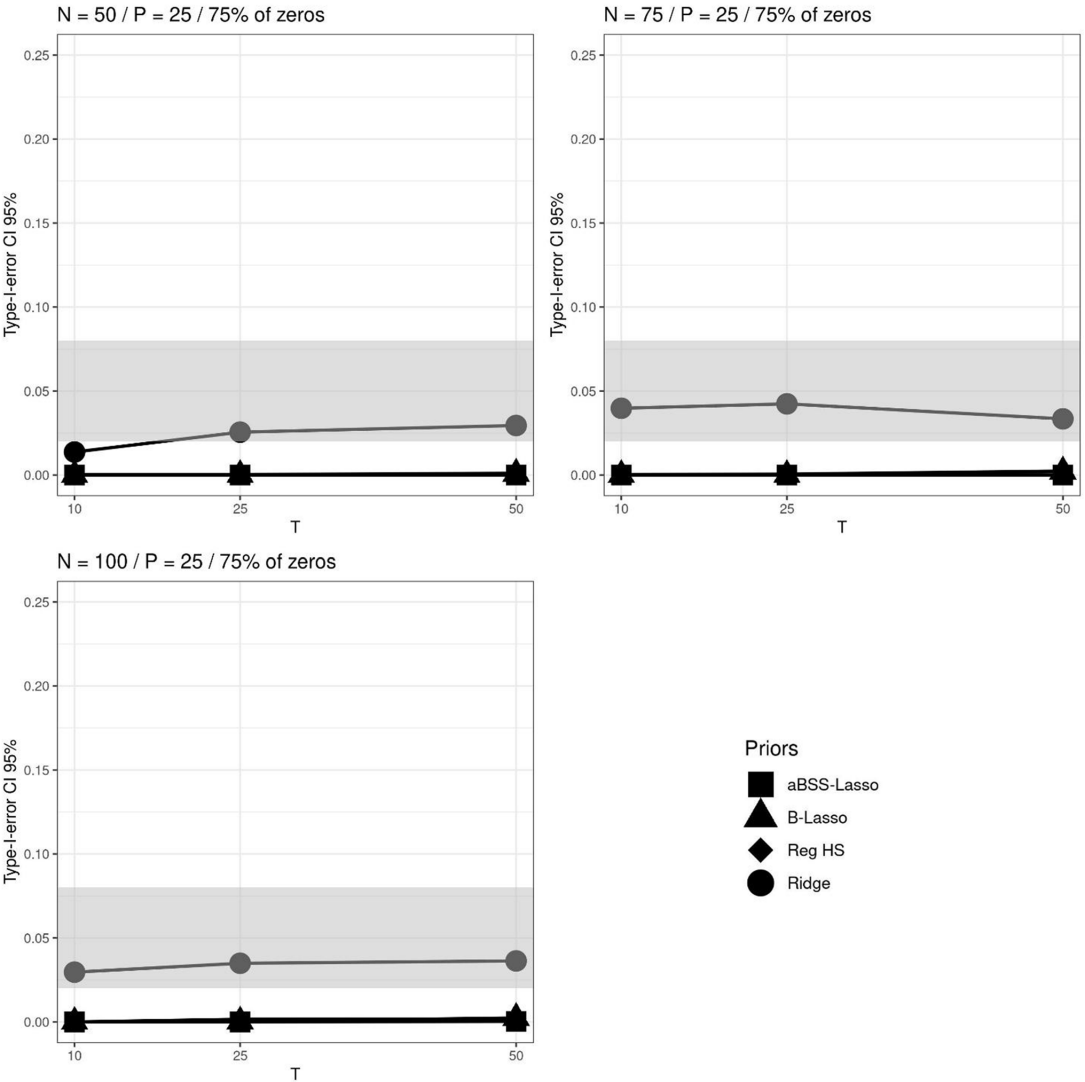
Type I error rates based on 95% credible interval (CI) across *N*, *T* and *P* for the average non-zero and zero slopes when 75% of the elements of the $\beta$ parameter vector are zeros. The *gray bands* correspond to the interval [0.02, 0.08] (which is approximately equal to the interval $[0.05\pm 1.96 \sqrt{0.05*0.95/M}]$, where *M* is the number of replications)

**Fig. 9:**
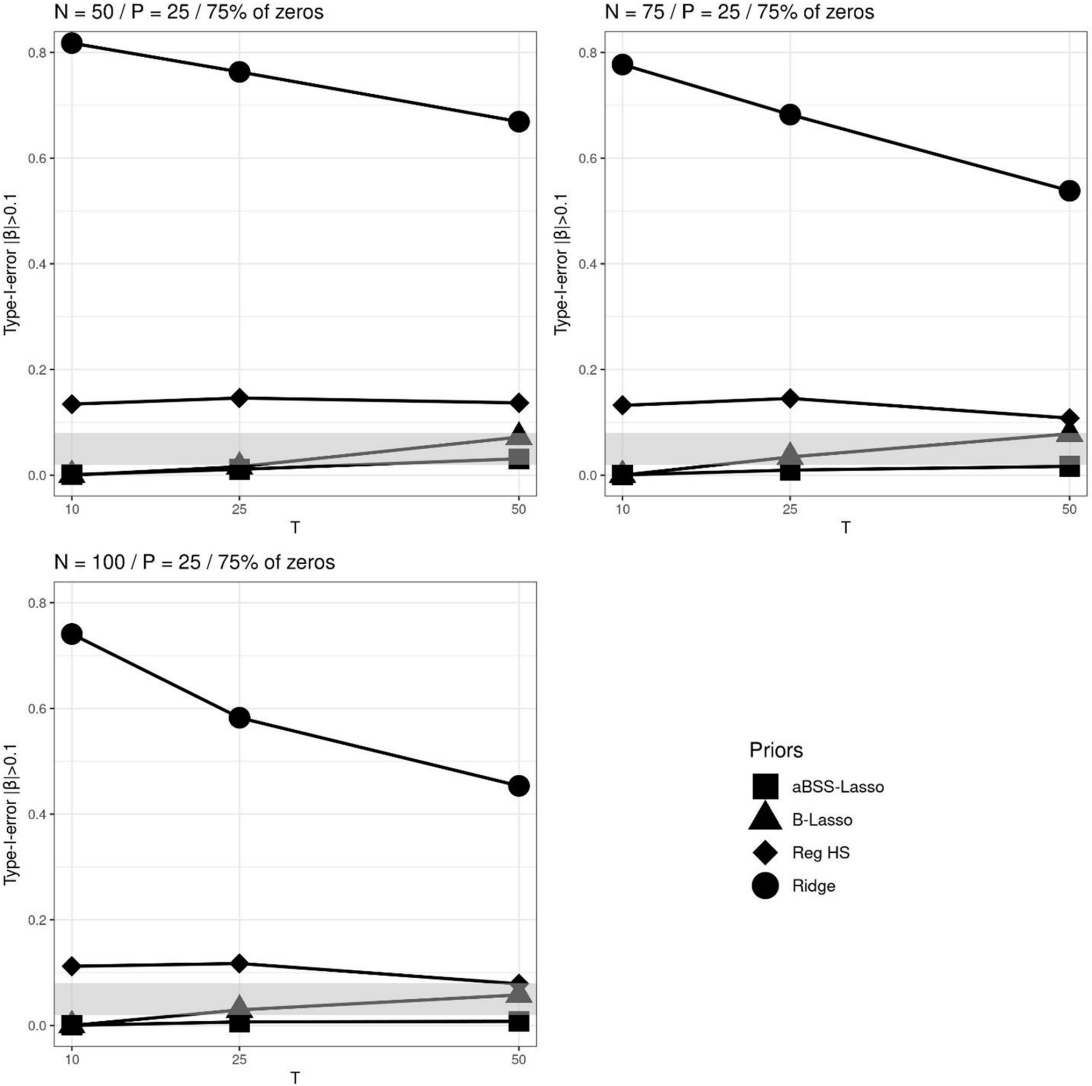
Type I error rates based on $|\beta _p|>0.1$’s thresholding rule across *N*, *T* and *P* for the average non-zero and zero slopes when 25% of the elements of the $\beta$ parameter vector are zeros. The *gray bands* correspond to the interval [0.02, 0.08] (which is approximately equal to the interval $[0.05\pm 1.96 \sqrt{0.05*0.95/M}]$, where *M* is the number of replications)

**Fig. 10:**
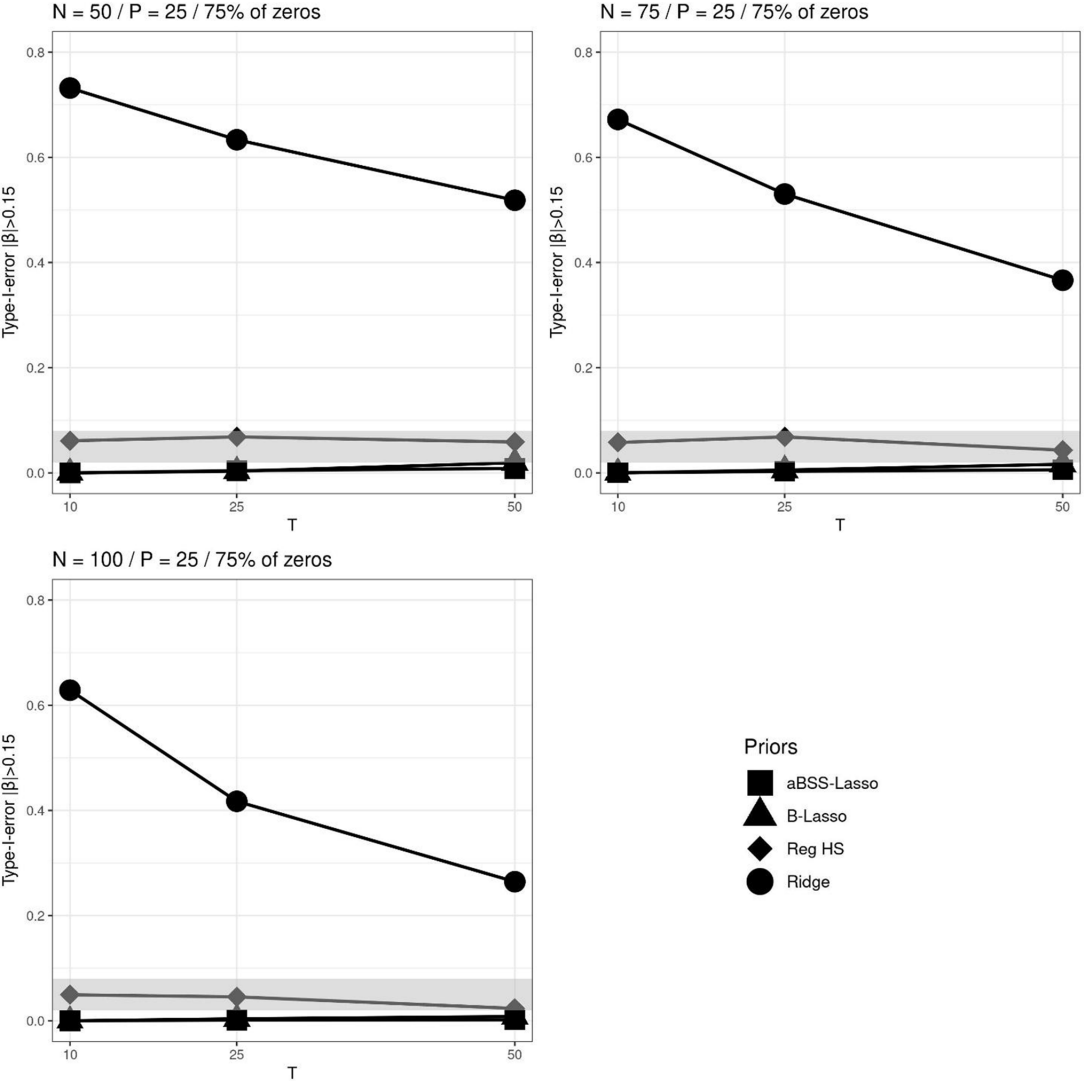
Type I error rates based on $|\beta _p|>0.15$’s thresholding rule across *N*, *T* and *P* for the average non-zero and zero slopes when 25% of the elements of the $\beta$ parameter vector are zeros. The *gray bands* correspond to the interval [0.02, 0.08] (which is approximately equal to the interval $[0.05\pm 1.96 \sqrt{0.05*0.95/M}]$, where *M* is the number of replications)

In contrast, we observed that the criteria used to evaluate the type I error provided rather contradictory results. More specifically, the type I error rates did not offer a better ranking of the regularizing priors. Figures 8, 9 and 10 illustrate the comparison of the regularizing priors when 75% of $\pmb {\beta }^*$’s components were zeros and $P=25$. The type I error rates based on the 95% CI clearly favored the ridge prior, as it was the only one that satisfied the admissible range. In contrast, the ridge prior seemed to be far away from the admissible range when computing the type I error rates based on the thresholding rules of 0.1 and 0.15. This was mainly due to the variability of the estimates which were not taken into account by the thresholding rules. In Fig. 4 (right), we observed that the parameter estimates were less than 0.025, while Figs. 9 and 10 (thresholding rules) suggest that between 40% and 80% of these estimates were greater than 0.1. However, this is not surprising because, in contrast to the 95% CI rule (see Fig. 8), the thresholding rules ignore the variability of the estimates by suggesting tight and deterministic intervals across replications. From the Markov–Chebyshev inequality, it can be intuitively seen that tight intervals imply inflated type I error rates as the probability boundary increases. Furthermore, deterministic intervals eventually imply difficulties in capturing the variability of the estimates. It is additionally shown in the Appendix that the ridge prior exhibits the largest RMSE (root mean square error) amongs the priors.

### Simulation 2 results

#### Convergence rates

Figures related to the convergence rates of each Bayesian estimation method across conditions are shown in the Supplementary materials. For the ridge prior, we observed convergence rates of 100% for all conditions. For the B-Lasso prior, the lowest convergence rate across conditions was 99%. For the reg. HS prior, the lowest convergence rate was 97%, and the highest was 99.5%. For the aBSS-Lasso prior, the lowest convergence rate was 89%, and the highest was 95.5%. We noted that replications which failed to converge were associated with divergent state transitions. In contrast, when the models converged, warnings associated with divergent transitions did not occur. For the remainder of this study, we mainly selected the replications that converged according to the $R\text {-hat}<1.1$ criterion. Again, the remaining quantities (mean estimates, type I error rates, etc.) were computed based on replications that converged. Note that the sampling precision was described in the Supplementary materials.

#### Sparsity

**Fig. 11:**
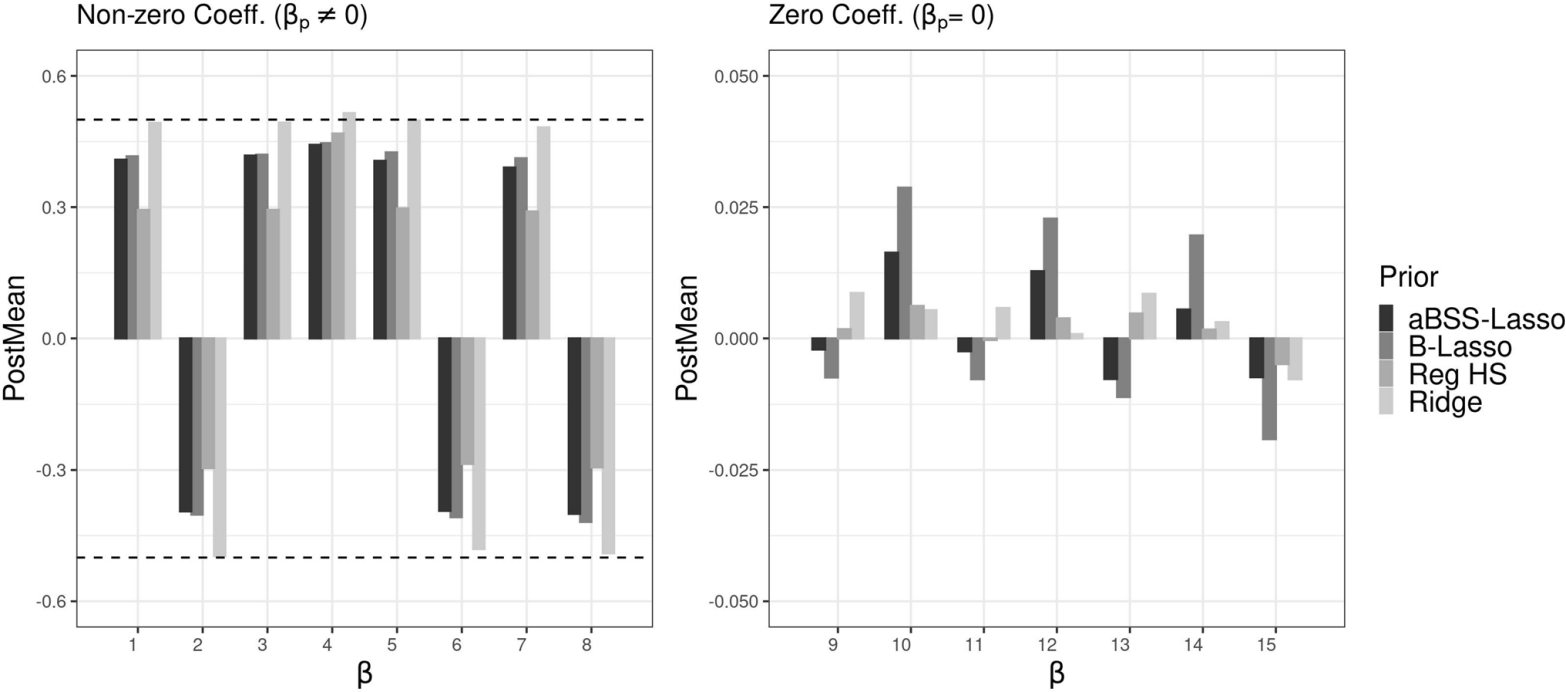
Comparison of posterior means of the slopes for each prior distribution for the case of $N=100$ and $T=50$. The *left panel* shows the posterior means of the non-zero coefficients. Positions 1 to 7 on the *x*-axis correspond to the slopes of the time-invariant covariates ($\beta _x$). Position 8 on the *x*-axis corresponds to the slope of the time-varying covariate ($\beta _z$). Positions 9 to 14 on the *x*-axis correspond to the interaction effects between the time-varying and time-invariant covariates ($\beta _{xz}$). The *red dashed lines* describe the population values of the nonzero slopes (0.5 and -0.5)

Figure 11 compares the posterior mean estimates of $\pmb {\beta }^*$ for each regularizing prior for the case of $N=100$ and $T=50$. The results show that, once again, the ridge prior had unbiased and accurate estimates of the non-zero coefficients and outperformed the other regularizing priors. In general, all priors were able to identify the zero coefficients (the largest values were given by the B-Lasso).

**Fig. 12:**
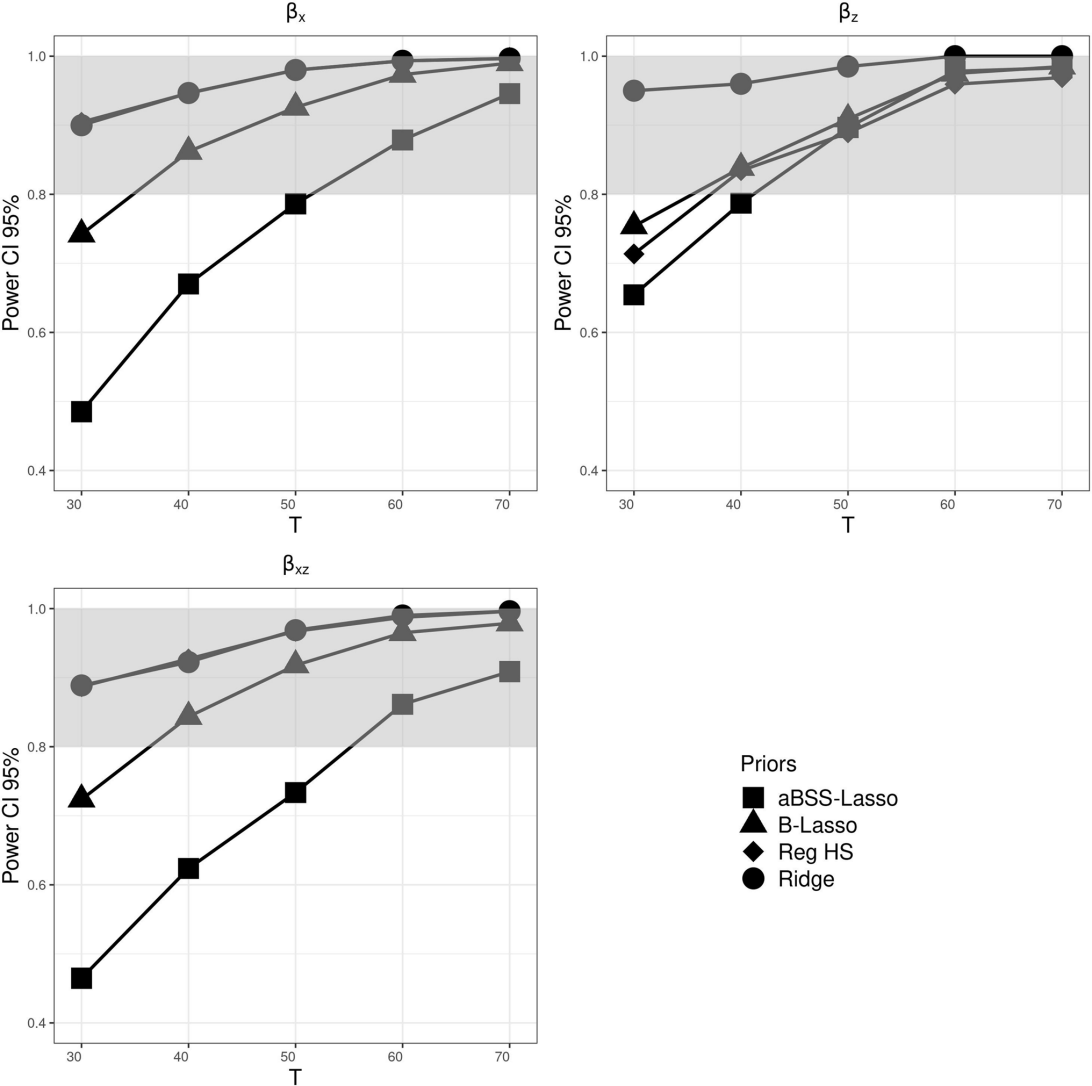
Power based on 95% credible interval (CI) for each prior distribution across different numbers of time points *T*, where the *gray bands* correspond to the interval [0.8, 1]. $\beta _x$ corresponds to the time-invariant covariates slopes. $\beta _z$ corresponds to the time-varying covariates slopes. $\beta _z$ corresponds to the interaction effect between the time-invariant and the time-varying covariates

The results related to the evaluation of the power of the estimates followed the same pattern as in Simulation Study 1. Figure 12 shows the behavior of the power based on the 95% CI across *T*. As shown in the Supplementary materials, the power based on the thresholding rules showed comparable results. Overall, we observed that the ridge prior had the best performance, followed by the reg. HS prior, which in turn outperformed the Lasso priors. Note that the Lasso priors, despite having the lowest power, still maintained a reasonably high power.

**Fig. 13:**
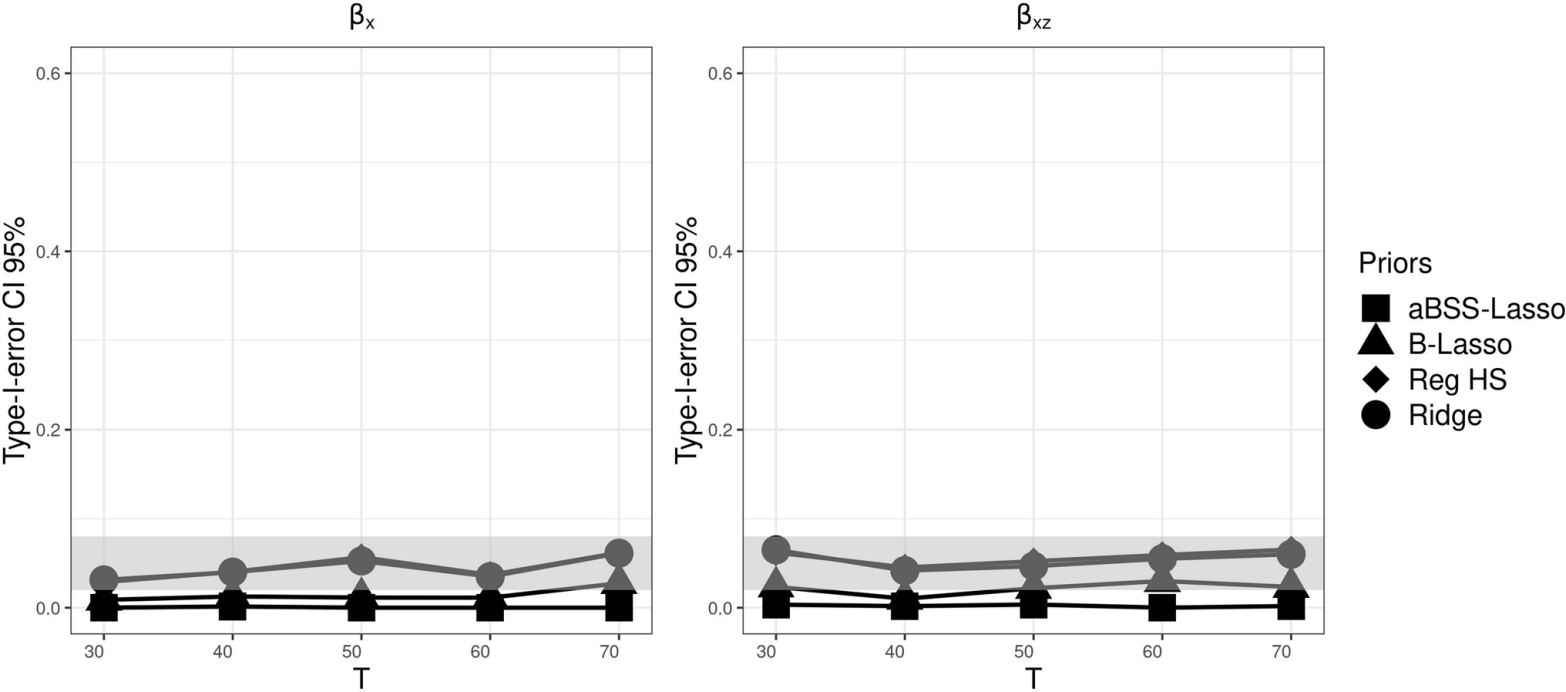
Comparison of the type I error rates based on 95% credible interval (CI) for each prior distribution across different numbers of time points *T*, where the *gray bands* correspond to the interval [0.02, 0.08] (which is approximately equal to the interval $[0.05\pm 1.96 \sqrt{0.05*0.95/M}]$, where *M* is the number of replications). $\beta _x$ corresponds to the time-invariant covariates slopes. $\beta _z$ corresponds to the time-varying covariates slopes. $\beta _z$ corresponds to the interaction effect between the time-invariant and the time-varying covariates

**Fig. 14:**
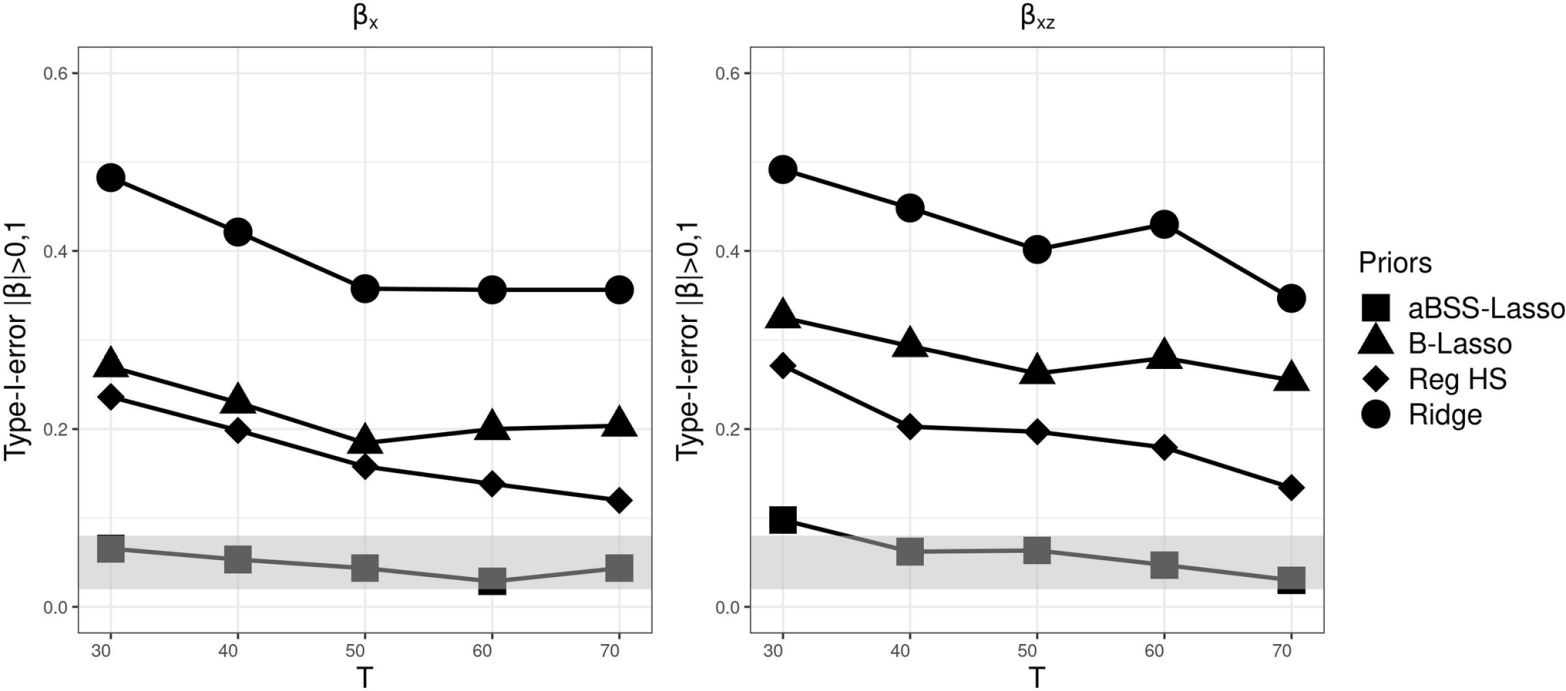
Comparison of the type I error rates based on $|\beta _p|>0.1$’s thresholding rule for each prior distribution across different numbers of time points *T*, where the *gray bands* correspond to the interval to the interval [0.02, 0.08] (which is approximately equal to the interval $[0.05\pm 1.96 \sqrt{0.05*0.95/M}]$, where *M* is the number of replications). $\beta _x$ corresponds to the time-invariant covariates slopes. $\beta _z$ corresponds to the time-varying covariates slopes. $\beta _z$ corresponds to the interaction effect between the time-invariant and the time-varying covariates

**Fig. 15:**
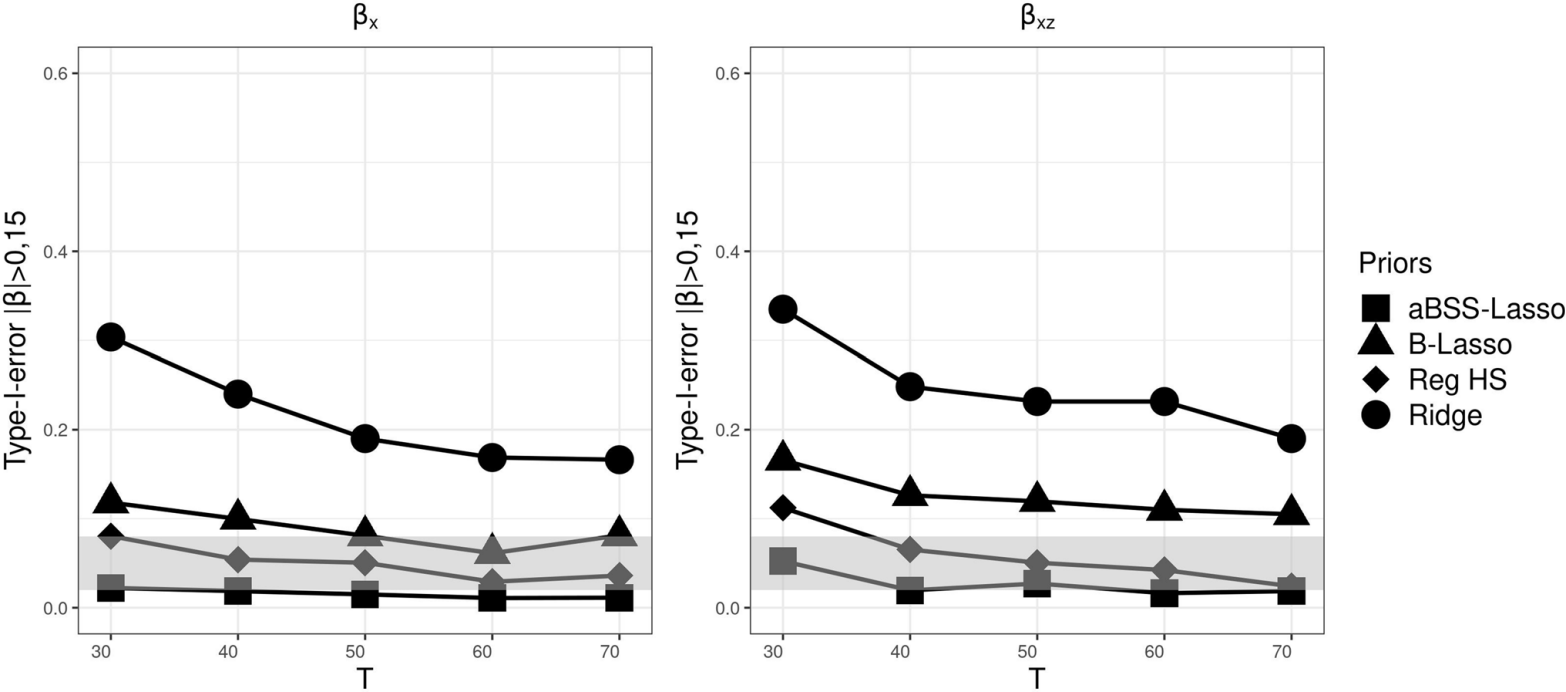
Comparison of the type I error rates based on $|\beta _p|>0.15$’s thresholding rule for each prior distribution across different numbers of time points *T*, where the *gray bands* correspond to the interval to the interval [0.02, 0.08] (which is approximately equal to the interval $[0.05\pm 1.96 \sqrt{0.05*0.95/M}]$, where *M* is the number of replications). $\beta _x$ corresponds to the time-invariant covariates slopes. $\beta _z$ corresponds to the time-varying covariates slopes. $\beta _z$ corresponds to the interaction effect between the time-invariant and the time-varying covariates

As in Simulation Study 1, we found that the type I error rate based on the 95% CI was favorable for the ridge prior, while being disfavored by the thresholding rules. Figures 13, 14, and 15 describe the behavior of the type I error rates across *T*. We observed that the B-Lasso rarely reached the admissible range. The aBSS-Lasso prior and the reg. HS prior performed best when using thresholding rules of 0.1 and 0.15, respectively. For the case of the 95% CI, the reg. HS performed as well as the ridge prior. These results again reflect the inability of the thresholding rules to deal with the variability in the estimates.

## Prior sensitivity analysis

In this section, we compare the regularizing priors using real data. We specifically conduct a sensitivity analysis for the case of the reg HS. prior to verify if a setting with a lighter tails would reach comparable outcomes to that of the ridge prior. We consider working on a multivariate model which is a simplified version of the NDLC-SEM used by Kelava et al. (2022) to forecast within-level latent factors in the context of university student drop-out.

### Data description

The data used in this study comes from the SAM (Studienabbruch in der Mathematik: German acronym for university dropout in mathematics, see Kelava et al., 2022) study, and was collected from a cohort of mathematics students starting their first semester at a German university. The measurements were carried out during the 2017-2018 winter semester. In this empirical application, we selected the same sample, which consisted of $N=122$ students and $T=50$ measurement occasions over 131 days. In total, we considered $J_1=9$ intra-individual observed items ($\pmb {Y}_{it}$) and $J_2=8$ inter-individual observed items ($\pmb {X}_i$). Overall, we had around 59% missing data.

### Implementation in Stan

Overall, the model was built from Eqs. (1), (3), (4), (5), (6), (7), and (9). At the within-level, we divided $\pmb {Y}_{it}$’s components into three categories of three items each, measuring one component of $\pmb {\eta }_{yit}$. This meant that we had $K=3$ intra-individual vector latent factors. The first category contained the items: *I liked the content, it is important for me to know a lot about the content*, and *This content will be useful for my future job*. It measured the latent factor *subjective importance of the content*. The second category measured *positive affect* through the items describing the tendency to feel *attentive, active*, and *stimulated*. The last category measured *negative affect* through the tendency to feel *nervous, anxious*, and *annoyed*. Note that the items were written originally in German and we provide here their translation into English. At the between level, $\pmb {X}_i$ contained $J_2 = 8$ items. The first group of items were students’ *math grades*, their *total score over 20 TIMSS (Trends in International Mathematics and Science Study;* Mullis, 2005*)* items, and their *sum of scores on the basic IQ (Intelligence Quotient)* that measure students’ *global cognitive abilities*. The second group included students *attainment value*, their *intrinsic value*, their *utility value*, their *costs* and their *mathematical self-concept*. These items were elaborated through the so-called *expected value theory* (for an overview, see Heusel et al., 2023). For this second group, we assumed no measurement errors, which allowed us to include them as between-level latent predictors. Therefore, $\pmb {\eta }_{xi}$ became a $(6\times 1)$-vector. Furthermore, we considered that $\pmb {\Gamma }=\pmb {0}_K$ and $\beta _z=0$, implying that Eq. (8) was removed from the model. By taking into account the interaction effects, we had in total 27 predictors of the latent states $S_{it}$.

Given that Stan cannot handle missing values directly (see Chapter 3 of Stan’s User Guide Stan Development Team, 2024), we adopted the same procedure as used by Li et al. (2022), where missing values are treated as parameters of the model. In particular, we replaced the missing data with placeholders and merged them with the observed data. Since we used factor models where the latent variables are also considered parameters to be estimated, we could deduce that the prior distribution for these placeholders would be similar to the individual likelihood of each observed data point.

Table 5 describes the designs of priors through which we conducted the prior sensitivity analysis. We particularly varied the scaling parameter $\sigma _\eta$, which took different values such as 1, $\sigma _{\epsilon 0}$ and 2 (or $\pi /\sqrt{3} \approx 2$). In addition, we considered several settings for the reg. HS priors, including cases with heavy (Cauchy) and lighter tails (with $c^2 \sim I\Gamma (2,8)$ as recommended by Piironen and Vehtari (2017) for logistic regression models). We also computed the posterior mode for all regularizing priors which are equivalent to the estimates of their frequentist counterparts.

To perform the sampling, we used *cmdstanpy* (Stan Development Team, 2023), which is a Stan interface in Python. We implemented four chains, each generating 2000 iterations and 2000 warm-ups. As a preliminary step, we ran the model with Stan’s ADVI (Kucukelbir et al., 2017, Automatic Differential Variational Inference) and used it to initialize the NUTS-HMC sampler. This practice has been proven to improve sampling efficiency as well as model estimates (Zhang et al., 2022, see also).

**Table 5:** Prior sensitivity analysis for the slope parameters of the transition probabilities with varying hyperparameters

	Hierarchies			
Priors	(1)	(2)	(3)	(4)
Ridge-0	$\beta _{p} \sim \mathcal {N}(0,1)$	$\lambda =1$		
Ridge-1	$\beta _{p} \sim \mathcal {N}(0,2^2)$	$\lambda =1$		
B-Lasso	$\beta _p\sim Laplace (0,\frac{1}{\lambda })$	$\lambda \sim \mathcal {C}^+(0,2.5)$		
ABSS-Lasso-0	$\beta _p = \beta _p^{*} \nu _p$	$\beta _p^{*}\sim Laplace (0,\frac{\sigma _{\epsilon 0}}{\lambda _p})$	$\nu _p \sim Beta (\frac{1}{2},\frac{1}{2})$	$\lambda _p \sim \mathcal {C}^+(0,2.5)$
ABSS-Lasso-1	−	$\beta _p^{*}\sim Laplace (0,\frac{1}{\lambda _p})$	−	−
ABSS-Lasso-2	−	$\beta _p^{*}\sim Laplace (0,\frac{2}{\lambda _p})$	−	−
reg. HS-0	$\beta _p \sim \mathcal {N}(0,\tilde{\lambda }_p^2 \tau ^2)$	$\lambda _p \sim \mathcal {C}^+(0,1)$	$\tau \sim \mathcal {C}^+(0,\tau _0)$	$c^2 \sim I\Gamma (\frac{1}{2},\frac{1}{2})$
	$\tilde{\lambda }_p = \sqrt{\frac{c^2 \lambda _p^2}{c^2 + \tau ^2 \lambda _p^2}}$		$\tau _0 = \frac{\pi }{\sqrt{3 N T}}$	
reg. HS-1	−	−	−	$c^2 \sim I\Gamma (2,8)$
	−		−	
reg. HS-2	−	−	$\tau \sim t _3^+(0,\tau _0)$	$c^2 \sim I\Gamma (2,8)$
	−		$\tau _0 = \frac{2}{\sqrt{N T}}$	

Note that we vary the scale $\sigma _\eta$ through different values, including 1, $\sigma _{\epsilon 0}$, and either 2 or $\pi /\sqrt{3} \approx 2$. Note that we did not include the B-Lasso with scaling parameters other than 1, as they had an *R*-hat>1.1 (related to very low ESS)

**Fig. 16:**
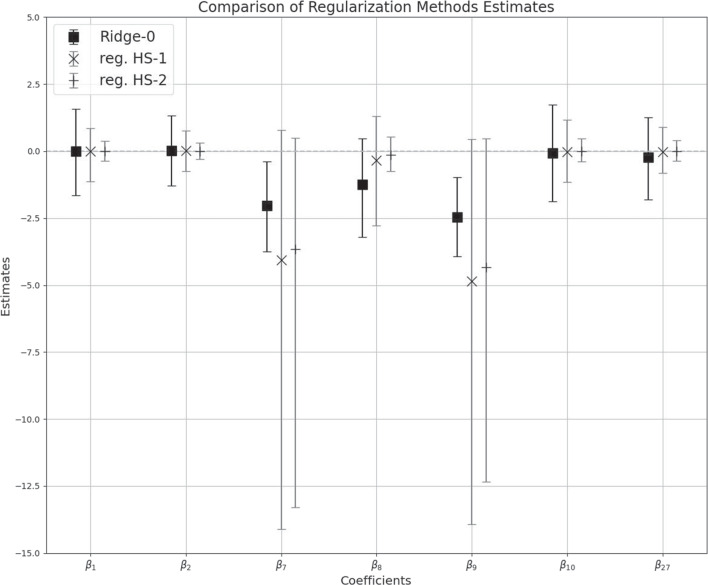
Comparison of the ridge priors and the reg. HS priors for some selected coefficients $\beta _p$ i.e., for $p=1,2,7,8,9,10,27$. The rest of the $\beta _p$s were ignored, as they were all close to zero (and were similar to $\beta _1,\beta _2.\beta _{10}$ and $\beta _{27}$). The bar plots correspond to the 95% highest posterior density interval, whereas the symbols correspond to the posterior means

### Results

Regarding sampling convergence and precision, we observed that all aBSS-Lasso priors and the reg. HS-0 prior produced divergent transition warnings. Specifically, for the aBSS-Lasso priors, these warnings undermined the sampling quality, resulting in $R\text {-hat} > 1.1$. Researchers often address these issues by applying the so-called brute force approach, which consists of increasing the Metropolis–Hastings acceptance ratio to 0.999 and the initial step size to 0.001. With these adjustments, divergent transitions decreased for the aBSS-Lasso prior, so we present results using this tuning. However, the brute force approach is not always a good solution. When applying brute force tuning to the reg. HS-0 prior, which initially had $R\text {-hat} < 1.1$, the divergent transitions were eliminated, but at the expense of maximum tree depth, resulting in $R\text {-hat} > 1.1$ and low *ESS*. Instead of using brute force, a simple solution would be to vary the hyperparameters. Notably, switching to the reg. HS with lighter tails (reg. HS-1 and reg. HS-2) removed divergent transitions while maintaining satisfactory $R\text {-hat}$ and *ESS*. This suggests that adjusting the prior is often preferable to tweaking the acceptance ratio, which can decrease computational efficiency.^10^ We excluded results that did not have $R\text {-hat} < 1.1$, i.e., the aBSS-Lasso priors without brute force tuning and the reg. HS-0 with brute force.

Overall, we observed that distributions with lighter tails (reg. HS-1, reg. HS-2, and Ridge-0) were more appropriate for the logistic model to define the transition probabilities. In contrast, the reg. HS-0 with heavy tails (the same as used in the simulation studies), the aBSS-Lasso, and the B-Lasso priors all exhibited a high standard deviation and a wide credible interval compared to the other priors. For the results related to the reg. HS-0, the aBSS-Lasso, and the B-Lasso priors, the reader is directed to the Supplementary materials. Figure 16 compares the estimates from the ridge priors and the reg. HS priors. The general tendency of the estimates suggests that $\beta _7,\beta _8$, and $\beta _9$ had the largest effects and that $\beta _1, \beta _2, \beta _{10}$, and $\beta _{27}$ tended to be very close to zero. As we changed the hyperparameters to obtain a reg. HS with lighter tails (Student-like tails), the standard deviation diminished, and the credible interval became tighter. We observed that the reg. HS-1 and reg. HS-2 were more or less comparable to the Ridge-1 prior, particularly for the larger effects. However, the Ridge-1 prior involved more uncertainty when the estimates were close to zero. This was not a problem for the reg. HS-1 and reg. HS-2 priors, which are characterized by a high concentration around zero, as initially described in Fig. 1. Furthermore, we found that the Ridge-0 had the tightest credible interval and the lowest standard deviation for all estimates. As shown in Fig. 16, it provided the best identification for large effects, in particular for $\beta _7 \, (OR=7.64)$ and $\beta _9 \, (OR=11.8)$. We observed some patterns where the credible interval was tighter than that of the reg. HS priors when the estimates were close to zero. These results are particularly consistent with our simulation studies, suggesting that the informativeness offered by the design of the ridge prior with its normal tails is advantageous. We also observed that for the ridge priors, the difference between the posterior means and the posterior modes was almost negligible. However, for the reg. HS-1 and reg. HS-2 priors, we observed that the posterior means tended to overestimate large effects, while the posterior modes tended to shrink the model. For the B-Lasso and the aBSS-Lasso in general, we observed that the posterior modes tended to reduce the overestimation problem implied by the posterior means without completely solving it (we refer the readers to the Supplementary materials for further visualization).

Finally, the DLVM used to analyze the student drop-out data revealed several important aspects: first, the *subjective importance of the content* and *the negative affect* predicted students’ intentions to drop out. The *positive affect* of the students also contributed to this tendency, but the effects were less obvious than those of the other two latent constructs. In contrast, the inter-individual differences in the baseline measures of *cognitive ability* and *expected values* only had a negligible effect on the intention to drop out. These baseline measures did not interact with either the *subjective importance of the content* or *the positive or negative affect scores*; that is, they did not modify the impact of current ratings during the ILD collection on the intention to drop out. Taken together, this implies that future studies aimed at preventing student drop-out could focus on changes in students’ perception of the relevance of the content, as well as their reported negative affect.

## Discussion

In this paper, we investigated the ability of Bayesian regularization methods to identify the non-zero and zero coefficients in the context of multilevel DLVM. As a representative framework, we proposed two-level Markov switching (vector) autoregressive models, which can be considered simplified versions of the NDLC-SEM framework (e.g., Andriamiarana et al., 2023; Kelava and Brandt, 2019; Kelava et al., 2022). This framework was employed to conduct a comparative analysis of the B-Lasso, aBSS-Lasso, and reg. HS priors, as well as a specific design of the ridge prior, when they were assigned to the coefficients of the latent state predictors (e.g., Brandt et al., 2018; Park and Casella, 2008; Piironen and Vehtari, 2017; Van Erp et al., 2019).

In a first simulation study, we considered a time-invariant transition probability and an increasing number of latent states predictors. In a second simulation study, we focused on time-varying covariates among the predictors, implying time dependence in the transition probability. Then, we conducted a prior sensitivity analysis using empirical data. We particularly wanted to investigate the behavior of the reg. HS prior tails compared to those of the ridge prior. Taken together, we found that the ridge prior had a favorable performance compared to other shrinkage priors in the modeling of complex Markov switching models in DLVMs.

Through simulation studies, we evaluated the properties of the sampling methods (convergence and precision) and retained replicated results that converged. Then, we computed the finite sample properties, specifically the average posterior means, bias measures, accuracy measures, as well as power and type I error rates. In line with our expectations, the results showed a large bias in the estimates, except for the ridge prior. In particular, it is known that a low SNR usually leads to biased estimates (Zwet & Gelman, 2022). We observed that when identifying the non-zero coefficients, the ridge prior outperformed the other regularizing priors. We also found that the reg. HS was able to identify the non-zero coefficients and could even perform as well as the ridge prior. Similar to that discussed in the literature, we found that the Lasso priors hardly identified the non-zero effects and tended to overshrink the model (Gelman et al., 2008; Van Erp et al., 2019; Bhadra et al., 2019). However, when it came to identifying the zero coefficients, it was not straightforward to decide which one of these priors provided the best performance. In particular, the type I error rates based on the 95% CI and the type I error rates based on the thresholding rules could not give a clear consensus. Nevertheless, regarding the level of bias and accuracy, it seemed clear that each prior was able to provide sparse estimation. In particular, when considering only the power and the type I error based on 95% CI, the results tend to favor the ridge prior in handling “nearly black” vectors of coefficients in the context of transition probabilities using logistic models.

To validate these findings, we conducted a prior sensitivity analysis using real data by varying the hyperparameters. We mainly focused on the case of the reg. HS priors that, for some patterns (particularly in terms of power and bias), could perform as well as the ridge prior. We then considered designs of the reg. HS with Student-tails, which are lighter than those of a Cauchy. As a result, we found that the ridge prior (Ridge-0) provided more accurate results (in terms of credible intervals and standard deviation). This is particularly consistent with the simulation studies. Broadly speaking, normal tails are known to perform better than Cauchy tails for logistic model coefficients (Ghosh et al., 2018; Ghosh, 2019). We also observed that the posterior means and the posterior modes as point estimates were similar for the ridge priors. However, for the other regularizing priors, the posterior means tended to overestimate large effects.

### Are Bayesian regularization methods a must?

The answer to this question is clearly “yes”. Throughout this paper, we used priors that are specifically centered around zero. Following Zwet and Gelman (2022)’s discussions, even weakly informative priors that are centered around zero have regularization properties of their own. One can therefore conclude that Bayesian regularization methods are mandatory to achieve sparse estimation for multilevel DLVM. However, the question related to the choice of regularizing priors needs to be addressed with more nuance. As described in the Results section, each prior regularizes in a different way due to its respective informativeness. Zwet and Gelman (2022) highlighted the importance of this informativeness and the generality of the prior–that is, its performance regardless of model complexity. The findings of our study concur with this argument and provide a relevant application of this general discussion to multilevel DLVM. In particular, for the case of the logistic model that was used to determine the latent states transition in our multilevel DLVM, the ridge prior with a simplified design was able to ensure both informativeness and generality, and could even outperform priors with sophisticated tuning.

As a general recommendation, we encourage applied researchers to diversify their choice of regularizing priors in order to cope with model uncertainty. In the context of multilevel dynamic latent class models (with logistic models) involving small effect sizes, we particularly recommend including ridge priors, particularly using specifications without an extreme amount of shrinkage (see Ghosh et al., 2018). We also suggest that when including reg. HS priors in the choice of regularizing prior, designs with Student-tails should be considered. We argue that specific designs of the reg. HS with lighter tails may be relevant for logistic models (for an overview, see Piironen and Vehtari, 2017). Furthermore, when using such models, researchers must beware of Lasso priors and instead rely on the previously suggested priors (see also Gelman et al., 2008). This is not to say that Lasso priors are irrelevant; rather, we suggest that they should not be prioritized at the expense of the ridge or the reg. HS priors.

Despite the ability of Bayesian methods to provide flexibility in handling parameter uncertainty in the context of complex models and small sample sizes (Andriamiarana et al., 2023; McNeish, 2019), we must point out that the computational time of the MCMC methods is always challenging. This in particular prevented us from providing answers to further research questions. As demonstrated in this paper, the implementation of regularization priors for two-state multilevel Markov switching models can be computationally challenging. In addition, most variants of NDLC-SEM, such as multilevel Markov switching models, use two states (e.g., Flückiger et al., 2022; Kelava et al., 2022; Vogelsmeier et al., 2019). We believe that exploring models with more than two states requires a completely separate study, as they are often much more complicated to handle in terms of creating realistic state-specific models.

From another perspective, implementing variational inference can also be considered as a faster alternative to MCMC sampling (Stan, for instance, has its specific version of variational inference; Kucukelbir et al., 2017). However, we must deal with a trade-off between computational speed and the quality of the estimates (Yao et al., 2018; Blei et al., 2017). An examination of the sample size requirements in comparison to the MCMC approach would be interesting in this respect.

## Supplementary Information

Below is the link to the electronic supplementary material.

Supplementary file 1 (pdf 10223 KB)

## Data Availability

Software codes used to generate the data and evaluate the models are available at https://github.com/Roots-awv/BReg_MSAR.git, and preregistration is not applicable.

## Footnotes

1 Note that in addition to its clarity on handling the amount of shrinkage, the original horseshoe prior often suffers from computational stability and convergence issues (Van Erp et al., 2019). For these reasons, the reg. HS prior was proposed by Piironen and Vehtari (2017), which is capable of handling sparse estimation in complex frameworks.

2 Note that most studies using dynamic models often have (vector) autoregressive models with lag 1 (see, for example, Chow and Zhang, 2013; McNeish, 2019; Schultzberg and Muthén, 2018). We also follow this approach in our framework.

3 Note that for reasons of identification, we assume that either a loading is fixed to one, or the latent variables’ variances are set to be equal to one.

4 For $S_{i1} = 1$, we assume that all individuals are in state $s=1$ at time $t=1$. Of course, $S_{i1}$ can be initialized in many ways depending on the context of the substantive research questions. In our case, we are interested in specific cases such as the inferences over students’ intentions to drop out from college or university. In this example, it would be reasonable to assume that initially, students do not have any intention to quit on the first day of the semester (see also Andriamiarana et al., 2023; Kelava and Brandt, 2019; Kelava et al., 2022).

5 For example, in the case of the student drop-out model, a small value of 0.05 is assumed because it is unlikely that an intention to drop out would change immediately after one day (see Andriamiarana et al., 2023; Kelava et al., 2022).

6 Note that the notation “|” represents the dependence of the parameter (here, $\beta _p$) on the hyperparameters (here, $\sigma _\eta ^2$ and $\tau _p^2$).

7 For generalized linear models, it is particularly difficult to quantify the value of the residual variance $\sigma _\eta ^2$. As we are using a logistic model for the transition probabilities, we assign a half-Cauchy prior to $\tau$, with scale parameter $\tau _0$ which depends on $\sigma _\eta$, where $\sigma _\eta ^2$ is equal to the theoretical variance $\pi ^2/3$. Note that this is not very far from the pseudo-variance of 4.

8 For each individual *i* at a measurement occasion *t*, we assume that $\pmb {\eta }_{xi}, \pmb {\eta }_{yit}$ and $\pmb {\eta }_{zit}$ are independently drawn. Hence, we can write that $f(\pmb {\omega }_{it}|\pmb {\Theta }_\eta )= f(\pmb {\eta }_{xi}|\pmb {\Theta }_\eta ) f(\eta _{zit}|\pmb {\Theta }_\eta ) f(\pmb {\eta }_{yit}|\pmb {\Theta }_\eta )$.

9 In the context of logistic models, Chen et al. (2010) wrote the following: “Our calculations indicate that $OR = 1.68, 3.47,$ and 6.71 are equivalent to Cohen’s $d = 0.2$ (small), 0.5 (medium), and 0.8 (large), respectively, when disease rate is 1% in the non-exposed group; Cohen’s $d < 0.2$ when $OR <1.5$, and Cohen’s $d > 0.8$ when $OR >5$”. Later, they wrote that: “We converted *OR* to 1/*OR* when $OR < 1$ in order to place all effects in a common frame for this discussion”. Hence, in our case, we had $OR(0.5) = exp(0.5)=1.64$ and $OR(-0.5) = 1/exp(-0.5)=1.67$ meaning that we have small effects.

10 We observed that sampling with brute force led to a saturation of maximum tree depth. Note that for sampling from NUTS-HMC, Stan uses trees for approximating sampling path iterations. A maximum tree depth equal to 10 (the maximum tree depth is fixed at 10 by default) means that all trees should have a maximal depth of 10, corresponding to a total of $2^{10} = 1024$ nodes.

## Declarations

### Open Practices Statement

Due to data protection rules, the data cannot be made available, but documented code for the analyses in this article is also available at https://github.com/Roots-awv/BReg_MSAR.git and is illustrated with simulated data.

### Copyrighted Material

The authors acknowledge that if the manuscript is accepted for publication, the authors are responsible for obtaining written permission to use the material.

### Copyright Transfer

The authors understand that for publication in Behavior Research Methods, the copyright to the manuscript must be transferred to Psychonomic Society (refer to http://www.psychonomic.org/psp/access.html for specific details). Open-access articles are published under a different license (see Open Access Publishing for more information).
