# Supplementary material for: Are Bayesian regularization methods a must for multilevel dynamic latent variables models?
Source: Behav Res Methods. 2025 Jan 22;57(2):71. doi: 10.3758/s13428-024-02589-9 (PMC11754388; doi:10.3758/s13428-024-02589-9)
Supplement: Supplementary file 1 — (pdf 10223 KB) [file 13428_2024_2589_MOESM1_ESM.pdf]

Supplementary materials: Are Bayesian Regularization Methods a Must for Multilevel Dynamic Latent Variables Models?

Vivato Andriamiarana, Pascal Kilian, Holger Brandt, and Augustin Kelava

University of Tübingen

We provide here supplementary materials containing results that are not shown in the manuscript for simulation study 1 and simulation study 2. We present here several graphics showing the small sample properties of the estimates. In particular, we illustrated the convergence rates and the sampling precision rates across data conditions. We also computed the absolute bias, the relative bias as well as the root mean squared error (RMSE). Their behavior across the data conditions are shown here. Furthermore, in the manuscript we only presented the type-I-error rates and the NDR when 50% of the components of the vector of slopes $\boldsymbol{\beta}$ are zeros. In this document, we presented these metrics when 75% of $\boldsymbol{\beta}$'s components are zeros.

This document is organized as follow: in the first section, we show the results for simulation study 1 and in the second section, we described the sampling precision rates of simulation study 2.

Simulation study 1

Convergence rates and sampling precision rates

Convergence Here, we describes the convergence rates across replications and the data conditions. We say that a model converge if we have R-hat > 1.1 for each parameter of the model. The results are presented in Figures (1) to (6). As explained in the manuscript, R-hat > 1.1 were always associated to divergent transitions warnings. However, as shown in the figures, we rarely observed these case and excluded them when computing the finite sample properties metrics (Average estimates, Bias, RMSE, . . .).

Sampling Precision We also calculated the sampling precision based on the threshold over the effective sample size (ESS). These thresholds are $x = 100, 400, 1000$. The precision rates are calculated across replications, so that for a given replication, if all parameters had $ESS > x$, then we say that the HMC sampling applied to that replication provided the expected sampling precision.

First, Figures (7) to (12) show the precision rates when model parameters have $ESS > 100$. The weakly informative prior provided a precision rate above 99%, with an average precision rate of 99.8% across all data conditions. For the Reg HS, the lowest precision rate was 97.5% and the average precision rate across data conditions

was 99.6%. For the aBSS-lasso prior, the lowest precision rate was 76% with an average rate of 94.7% across data conditions. For the B-Lasso prior, the lowest precision rate was 51.5% and the average rate across data conditions was 82.7%. Note that for the B-Lasso prior, precision rates were particularly low when the length of time points T was small. As shown in the figures, larger T were needed to increase these precision rates. Second, Figures (13) to (18) show the precision rates when model parameters have $ESS > 400$. The weakly informative priors (with an average precision rate of 98.5% and a minimum precision rate of 92%) and the Reg HS (with an average precision rate of 98.1% and a minimum precision rate of 95%) had the best precision rate. The aBSS-Lasso prior provided lower precision with respect to $x = 400$ (with an average rate of 86.4% and a minimum rate of 86.4%) The B-Lasso prior performed the worst at this threshold. As shown in figures, larger N and T were required to cope with the increase in P. Third, in Figures (19) to (24), we also provided the precision rates when all model parameters have $ESS > 1000$.

Finite sample properties

To evaluate the properties of the estimates, we computed several quantities that provide information relative to the average posterior means, the biases, the accuracy, the power and the Type I error of the estimates.

Posterior means We compute the average posterior means across replications. The assocaited results are given by Figures (25) to (36)

Absolute Bias We calculate the absolute bias which, for an estimator $\hat{\theta}_m$ of a parameter θ, is computed by the following quantity:

$$AB = \frac{1}{M} \sum_{m=1}^{M} |\theta - \hat{\theta}_m|$$

Figures (37) to (42) show the related results.

Relative Bias The relative bias of an estimator $\hat{\theta}_m$ of a parameter θ is computed by the following quantity:

$$RB = \frac{1}{M} \sum_{m=1}^{M} \frac{\hat{\theta}_m - \theta}{\theta}$$

Figures (43) to (48) show the related results.

RMSE The RMSE of an estimator $\hat{\theta}_m$ of a parameter θ is computed by the following quantity:

$$RMSE = \sqrt{\frac{1}{M} \sum_{m=1}^{M} (\theta - \hat{\theta}_m)^2}$$

Figures (49) to (60) show the related results.

Power rates The following figures show the power rates and Type-I-error rates.

Type-I-error rates

Simulation study 2

In this section, we present the sampling precision over the data conditions of Simulation Study 2. To compute this quantity we applied the same thresholds to ESS as (simulation study 1), i.e. $x = 100, 400, 1000$. Figure (**??**) shows the corresponding results.

When the threshold was $x = 100$, the results showed that the weakly informative prior reached precision rate of 100%. In other words, all parameters produced 100 ESS across all replications and all data conditions. For the other priors, the lowest sampling precision rates were respectively: 98.5% for the B-Lasso, 94% for the Reg. HS and 81% for the aBSS-Lasso. When the threshold was $x = 400$, the weakly informative prior still provided a sampling precision rate of 100%. For the the B-lasso, the lowest rate was 96.5% and the highest was 100%. For the Reg. HS, the lowest rate was 86% and the highest was 92%. For the aBSS-Lasso, the lowest rate was 68% and the highest was 79%. When the threshold was $x = 1000$, the sampling rates for all priors decreased further. The B-Lasso prior had the best sampling rates with an average rate of 87.4%. This is followed by the weakly informative prior with a sampling rate of 57.9%. For the aBSS-Lasso and the Reg. HS priors, the average sampling rates were 51.8% and 44.3% respectively.

Prior sensitvity Analysis

In this section, we show the results related to prior sensitivity analysis. We used real data through wich we compared the regularzing priors. We initially proposed at least 2 or 3 different of setting hyperparameters for each prior. However, due to convergence monitoring issues (that brute force couldn't solve), we only present one version of the B-Lasso prior.

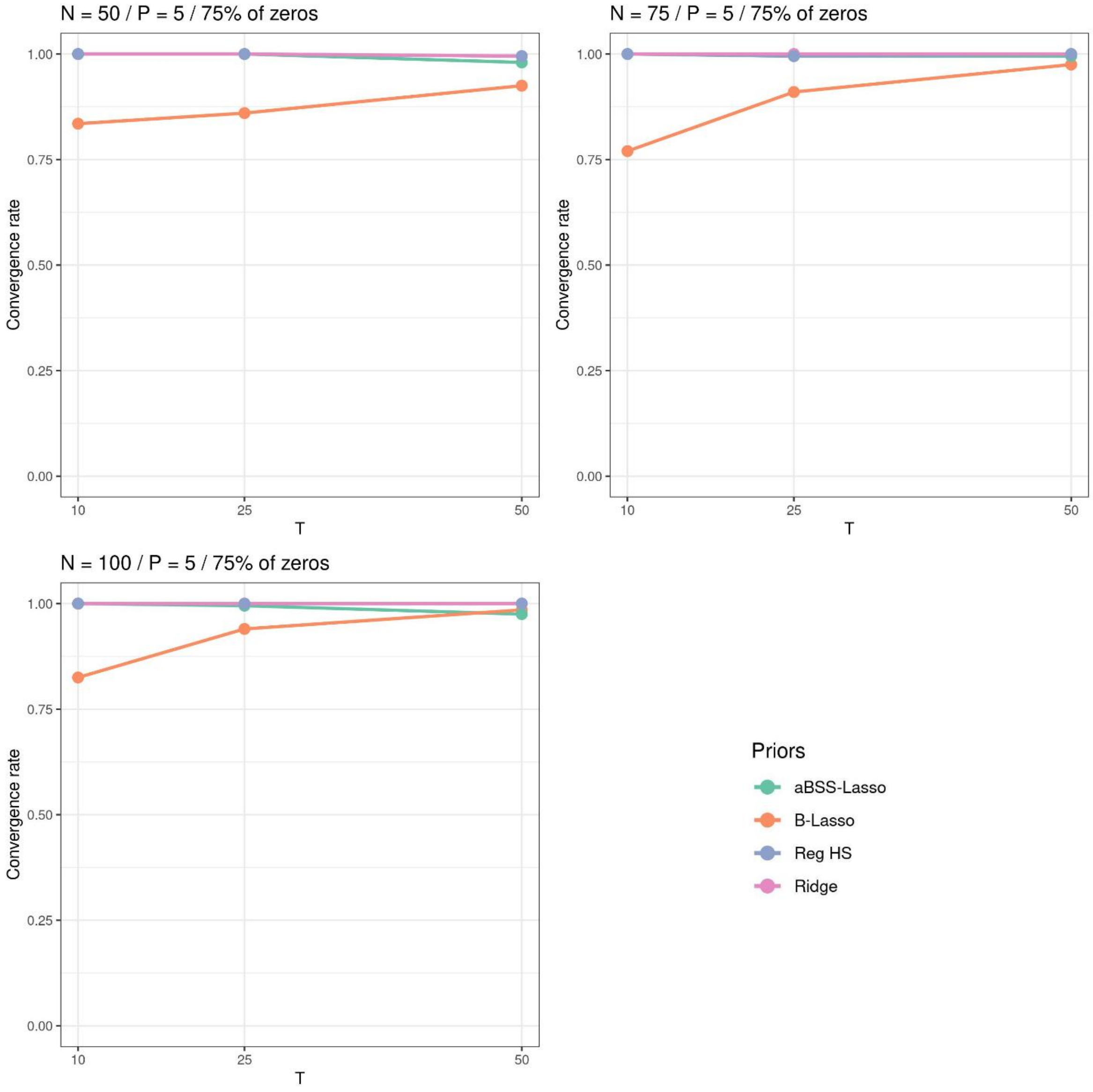

Figure 1: Convergence rate of the sampling across N and T when $P = 5$ and 75% of the elements of the $\boldsymbol{\beta}^*$ are zeros (simulation study 1).

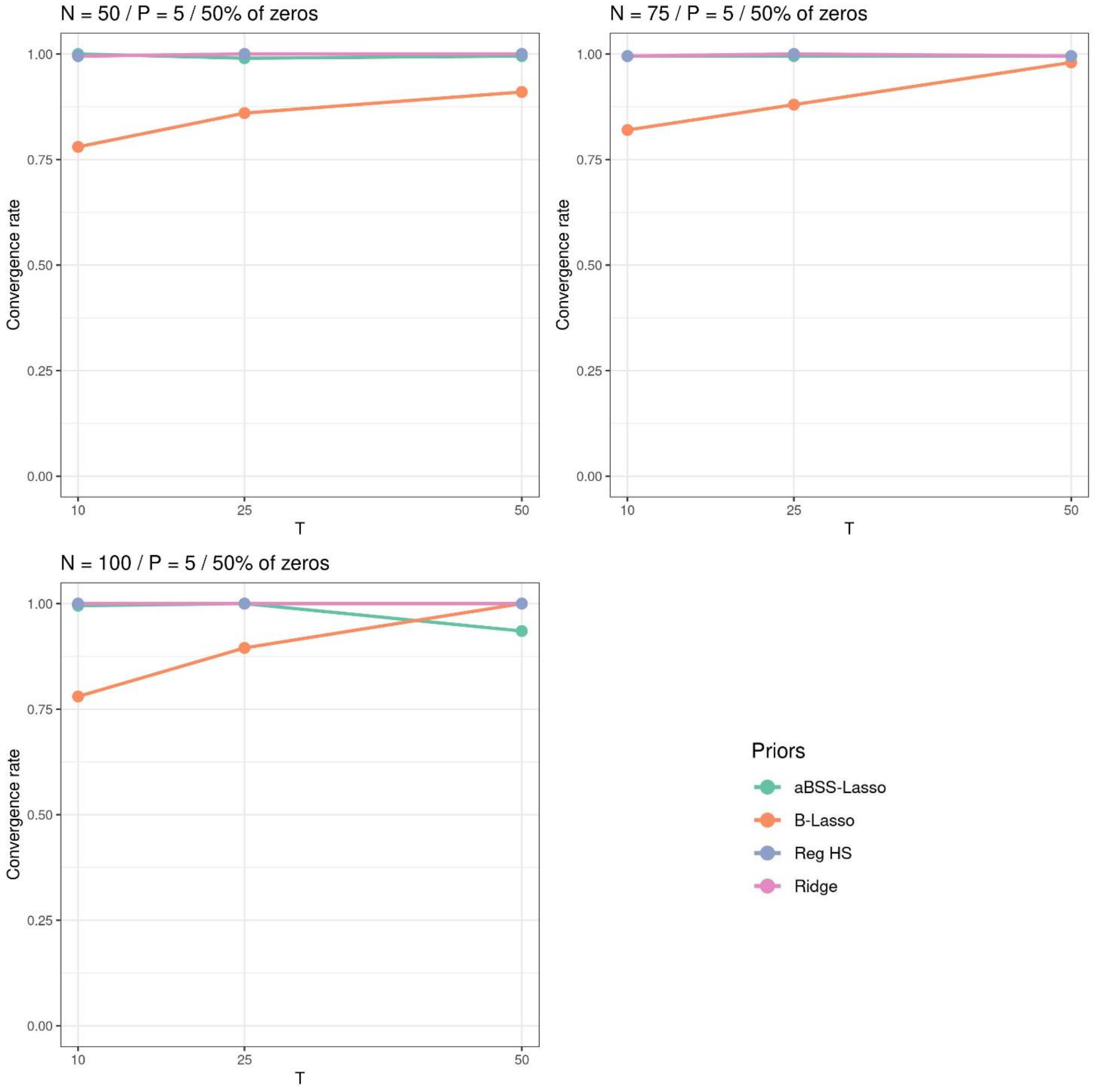

Figure 2: Convergence rate of the sampling across N and T when $P = 5$ and 50% of the elements of the $\boldsymbol{\beta}^*$ are zeros (simulation study 1).

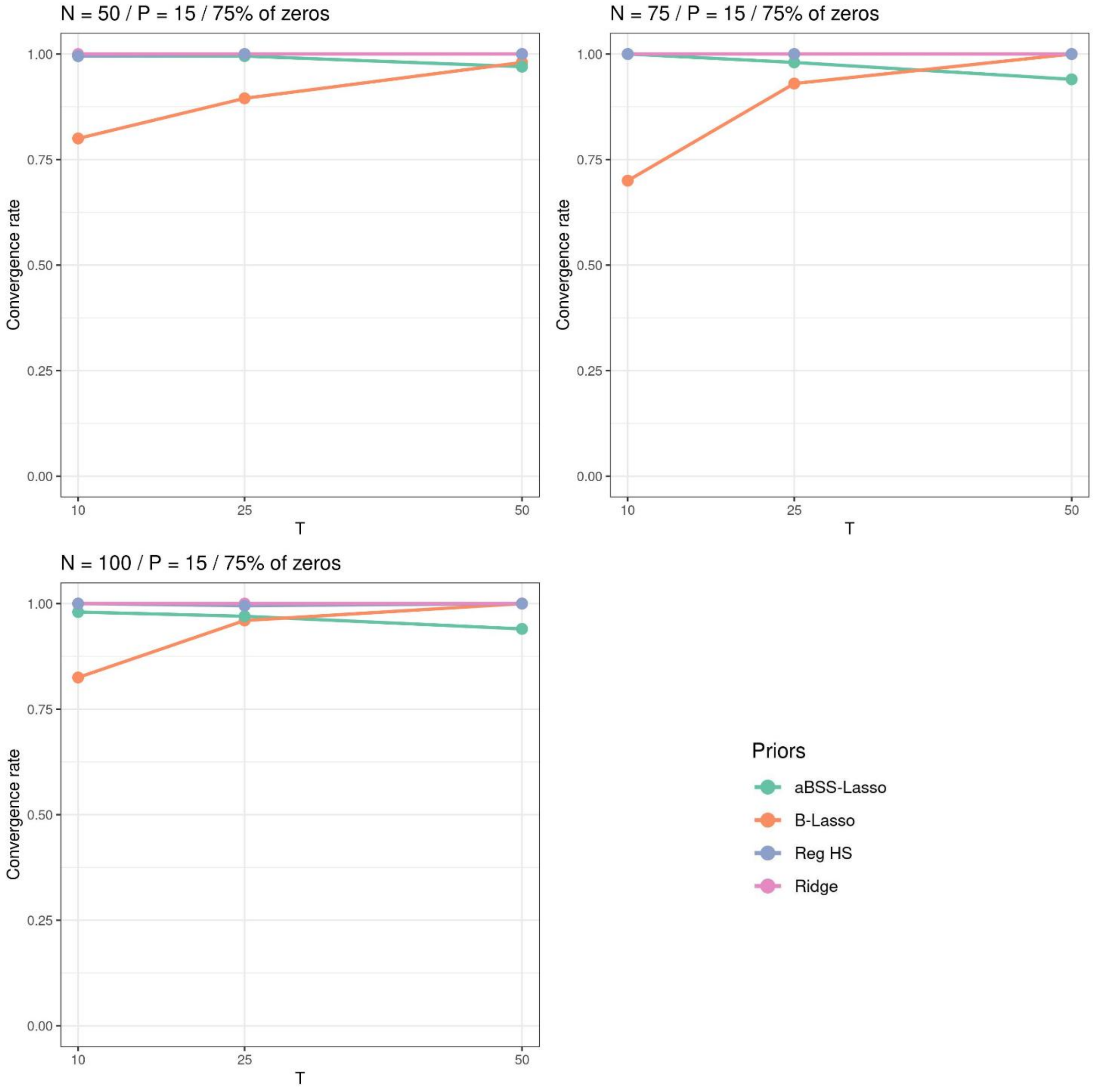

Figure 3: Convergence rate of the sampling across N and T when $P = 15$ and 75% of the elements of the $\boldsymbol{\beta}^*$ are zeros (simulation study 1).

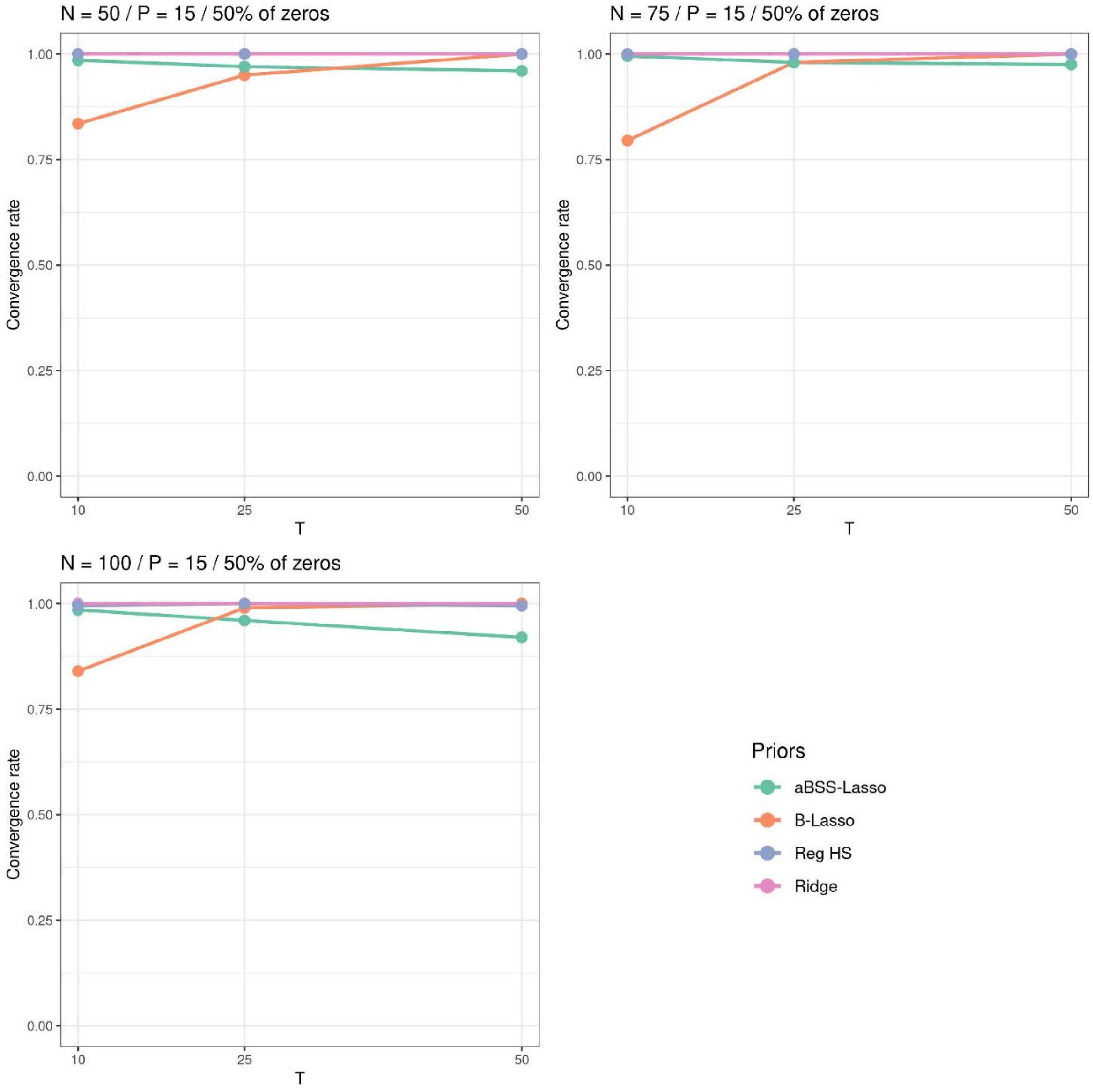

Figure 4: Convergence rate of the sampling across N and T when $P = 15$ and 50% of the elements of the $\boldsymbol{\beta}^*$ are zeros (simulation study 1).

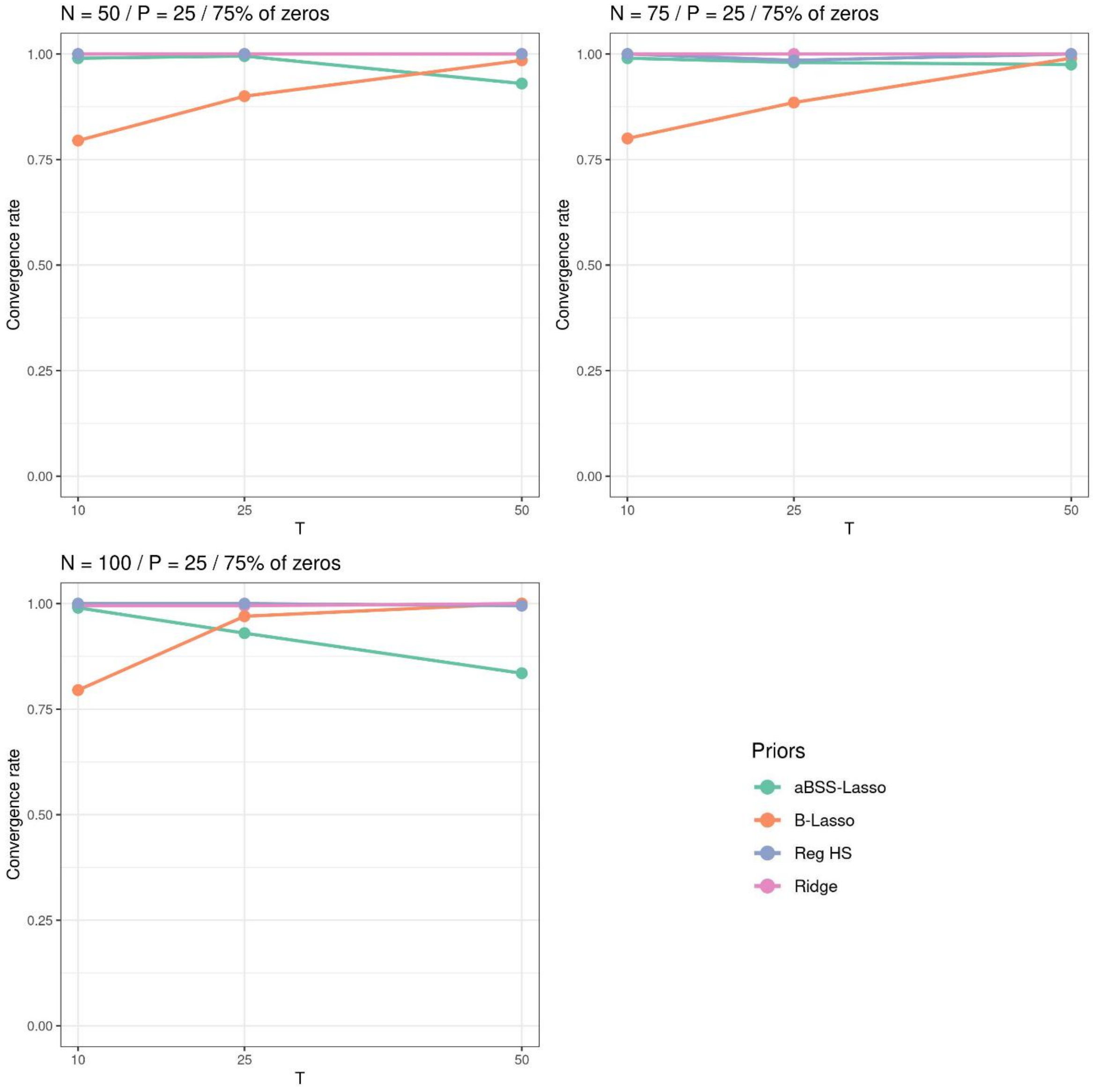

Figure 5: Convergence rate of the sampling across N and T when $P = 25$ and 75% of the elements of the $\boldsymbol{\beta}^*$ are zeros (simulation study 1).

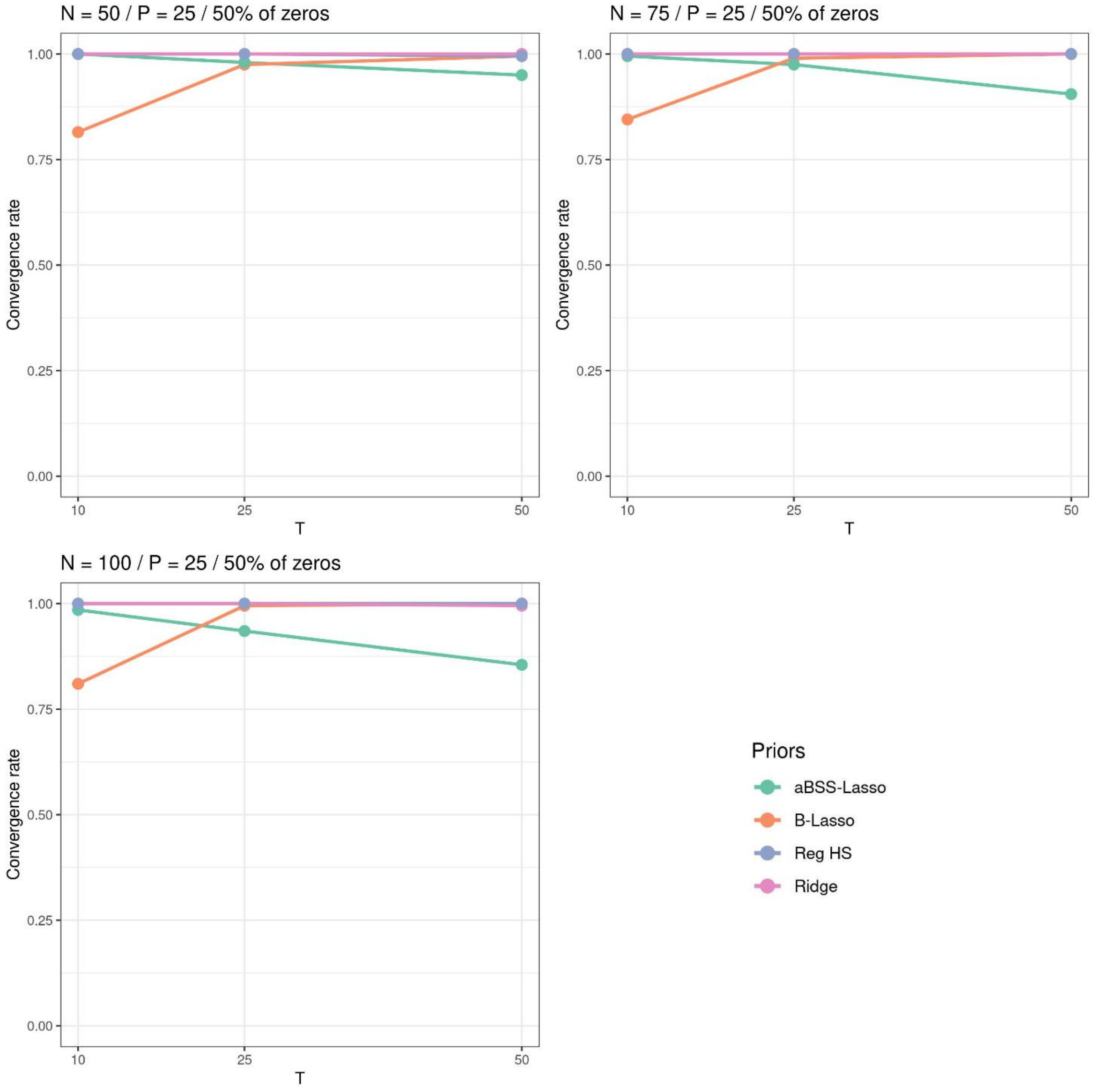

Figure 6: Convergence rate of the sampling across N and T when $P = 25$ and 50% of the elements of the $\boldsymbol{\beta}^*$ are zeros (simulation study 1).

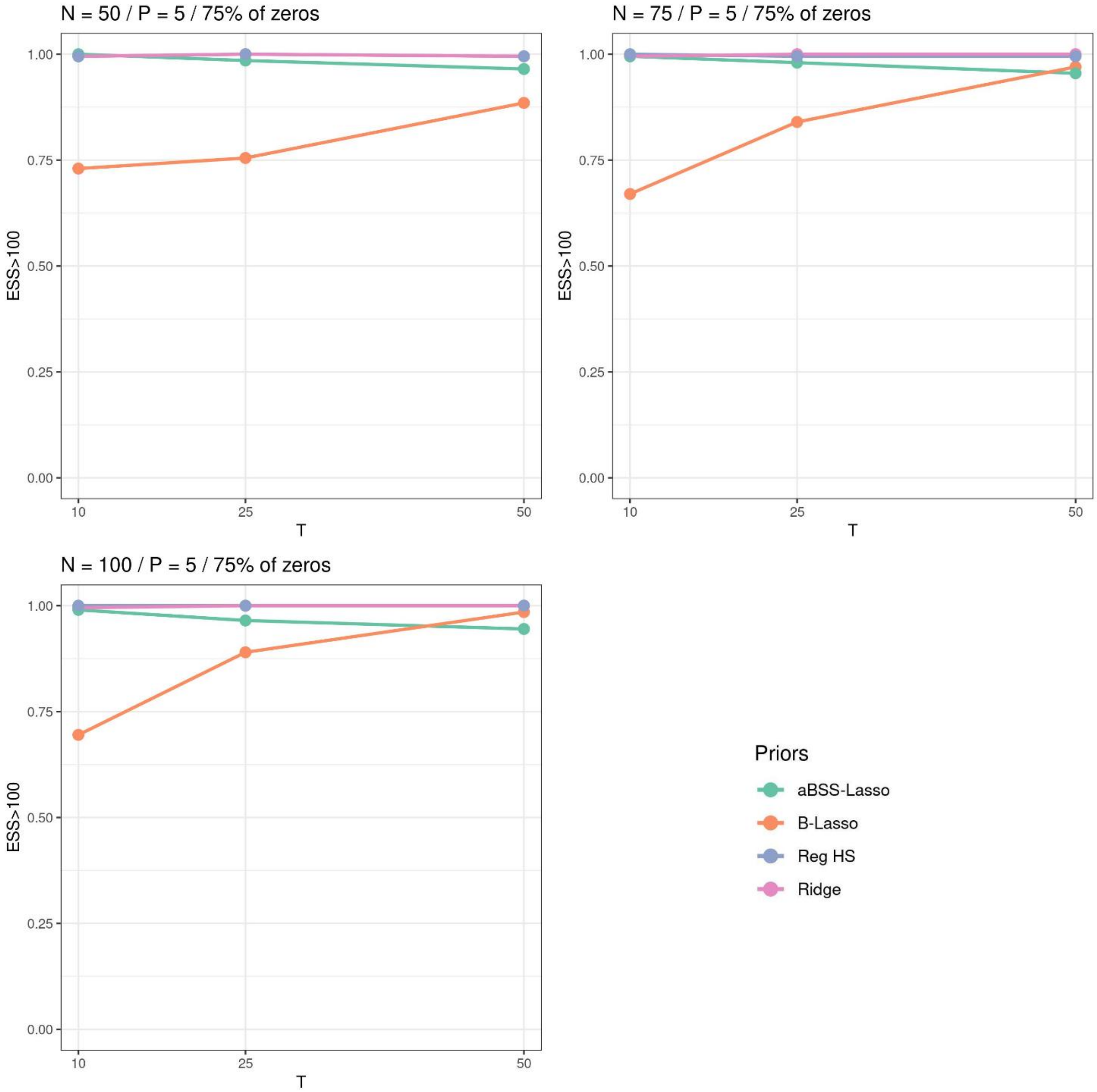

Figure 7: Precision rate of the sampling based on $ESS > 100$ threshold across N and T when $P = 5$ and 75% of the elements of the $\boldsymbol{\beta}^*$ are zeros (simulation study 1).

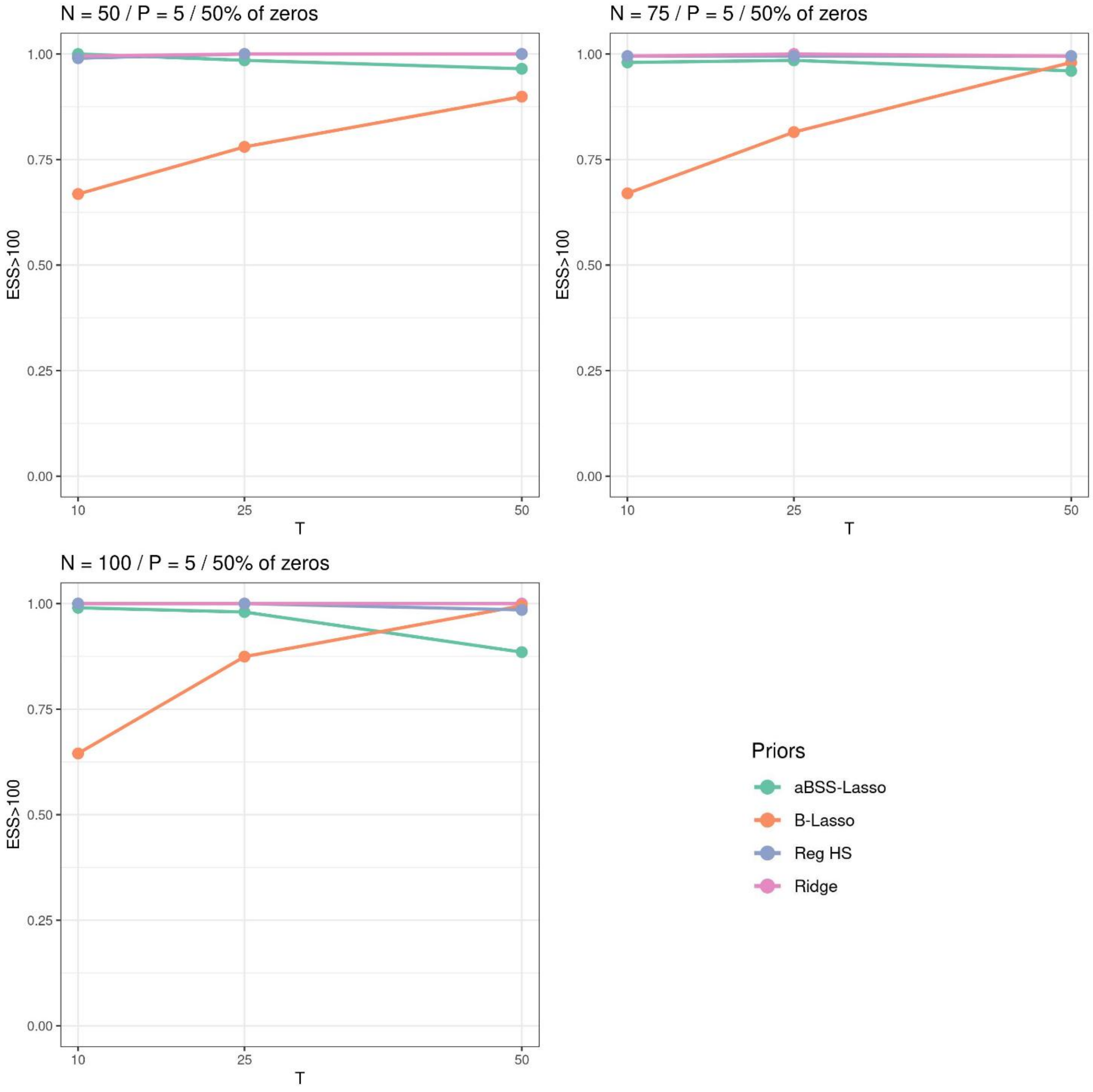

Figure 8: Precision rate of the sampling based on $ESS > 100$ threshold across N and T when $P = 5$ and 50% of the elements of the $\boldsymbol{\beta}^*$ are zeros (simulation study 1).

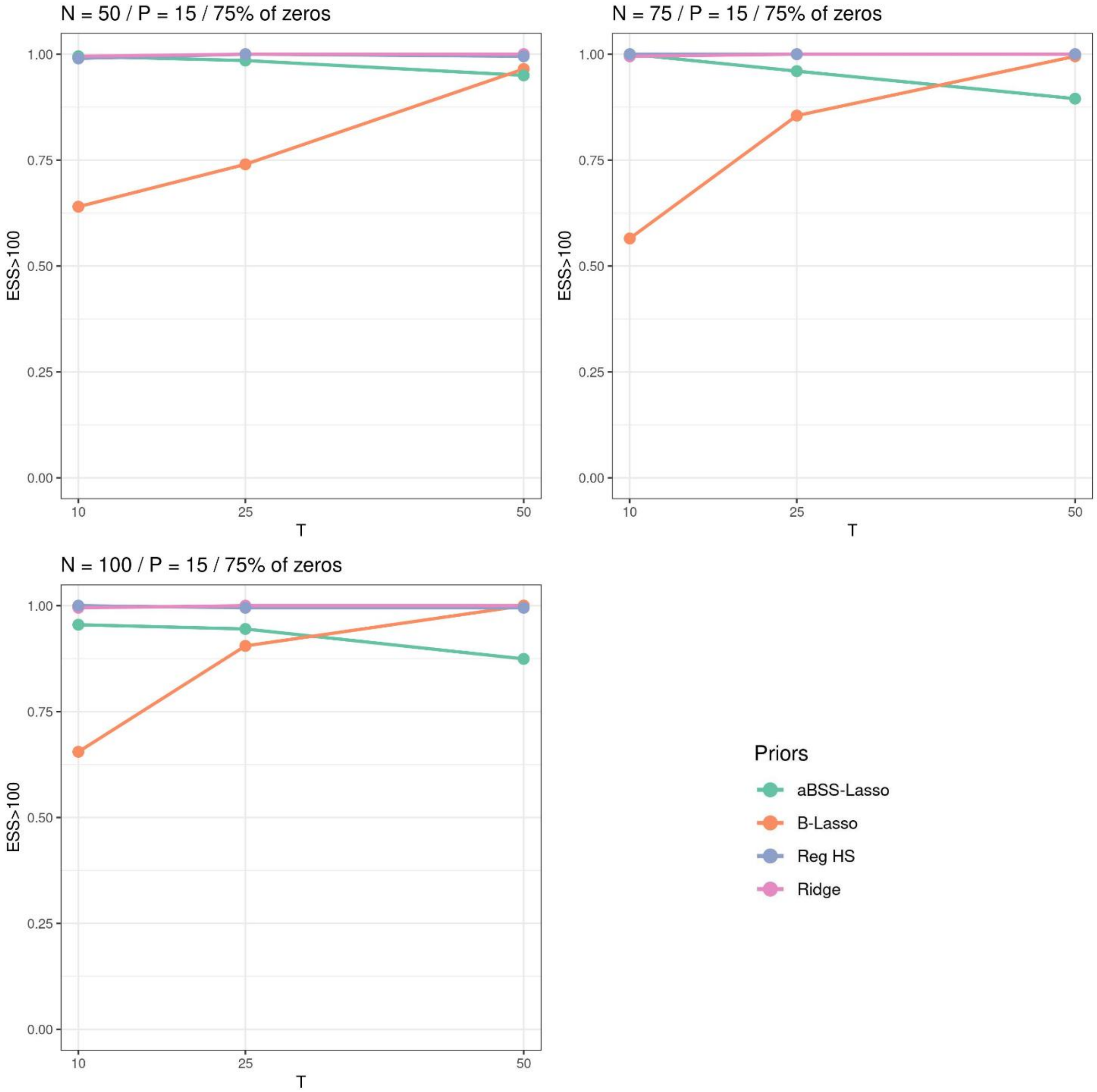

Figure 9: Precision rate of the sampling based on $ESS > 100$ threshold across N and T when $P = 15$ and 75% of the elements of the $\boldsymbol{\beta}^*$ are zeros (simulation study 1).

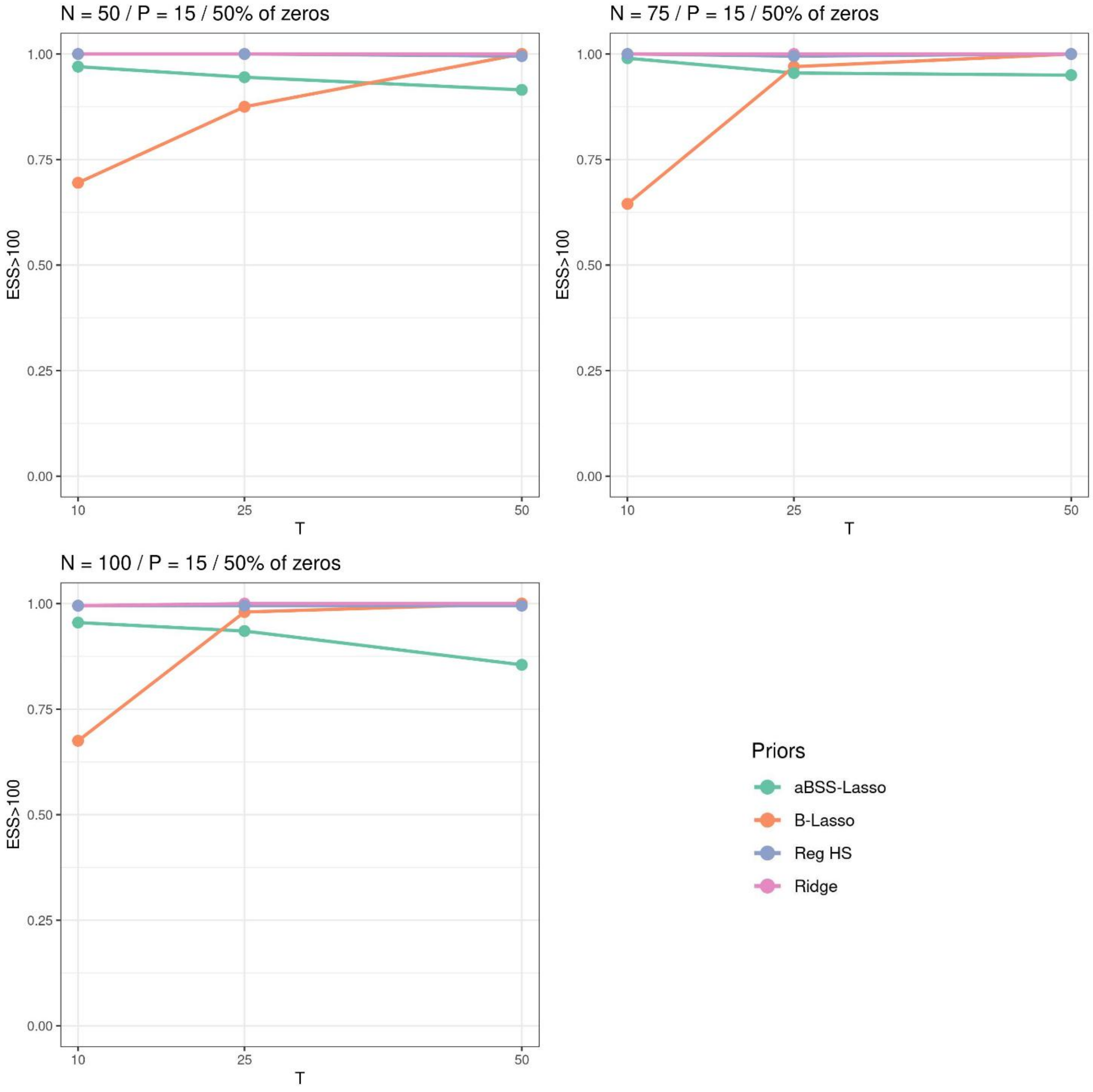

Figure 10: Precision rate of the sampling based on $ESS > 100$ threshold across N and T when $P = 15$ and 50% of the elements of the $\boldsymbol{\beta}^*$ are zeros (simulation study 1).

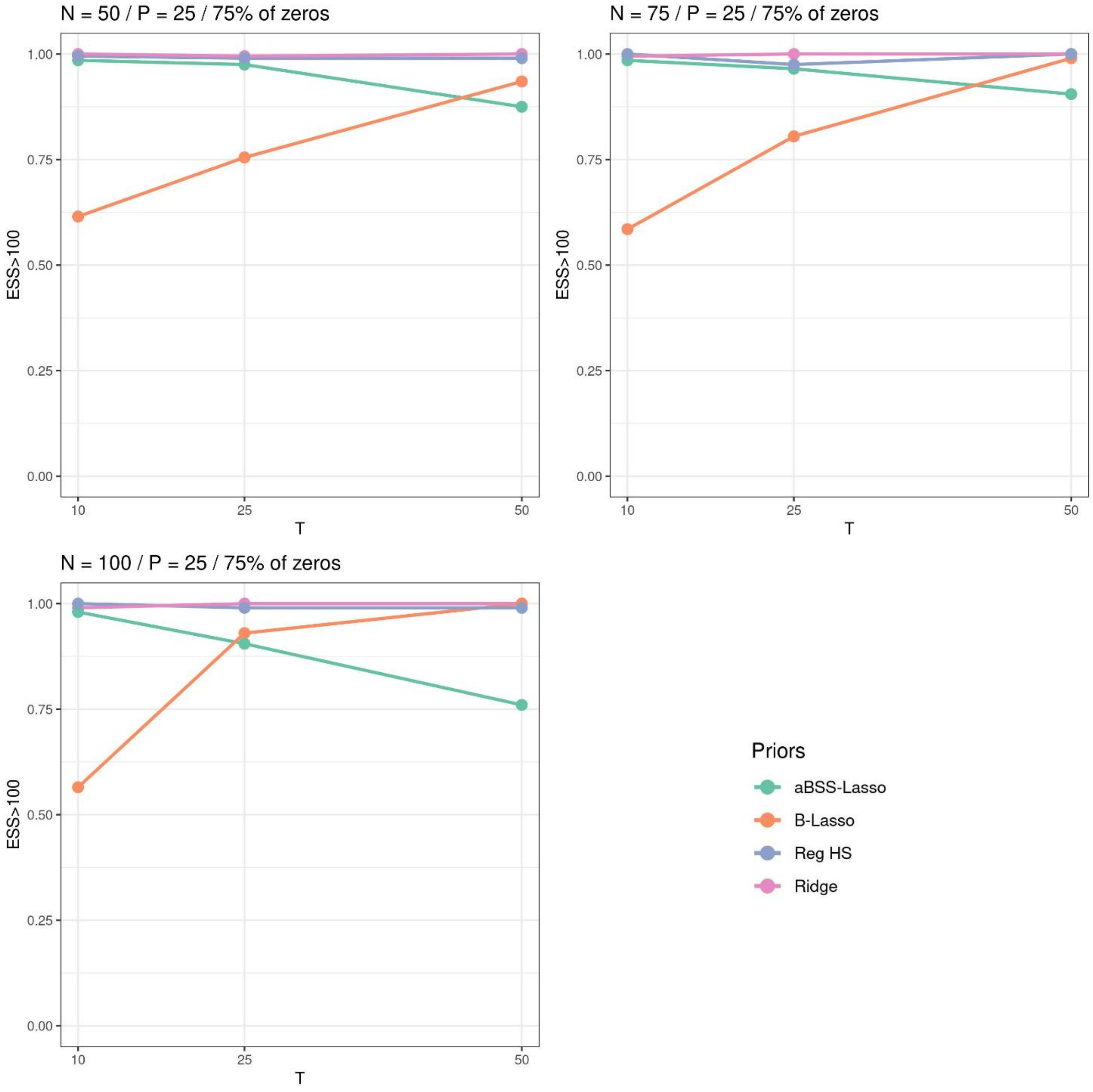

Figure 11: Precision rate of the sampling based on $ESS > 100$ threshold across N and T when $P = 25$ and 75% of the elements of the $\boldsymbol{\beta}^*$ are zeros (simulation study 1).

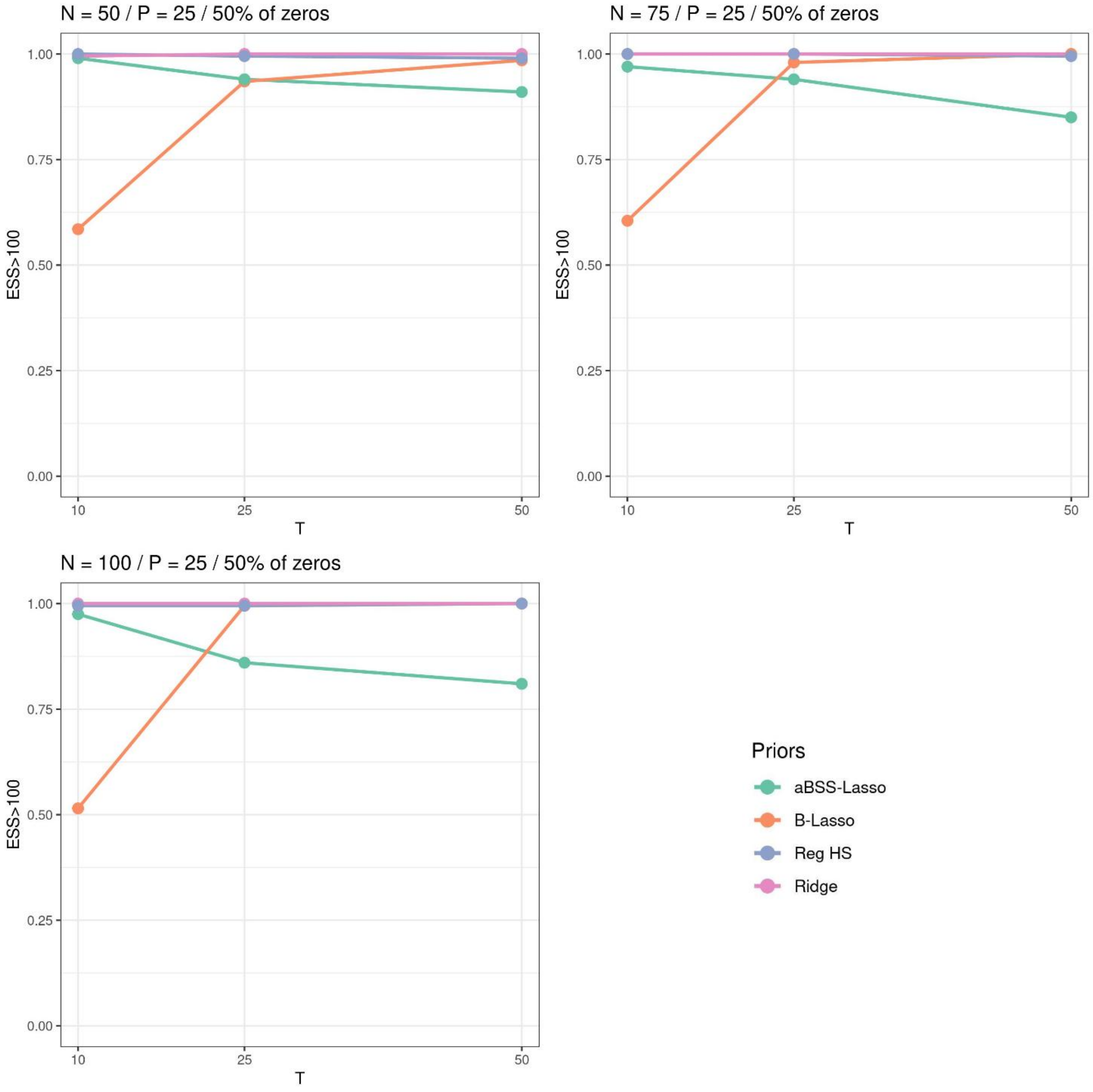

Figure 12: Precision rate of the sampling based on $ESS > 100$ threshold across N and T when $P = 25$ and 50% of the elements of the $\boldsymbol{\beta}^*$ are zeros (simulation study 1).

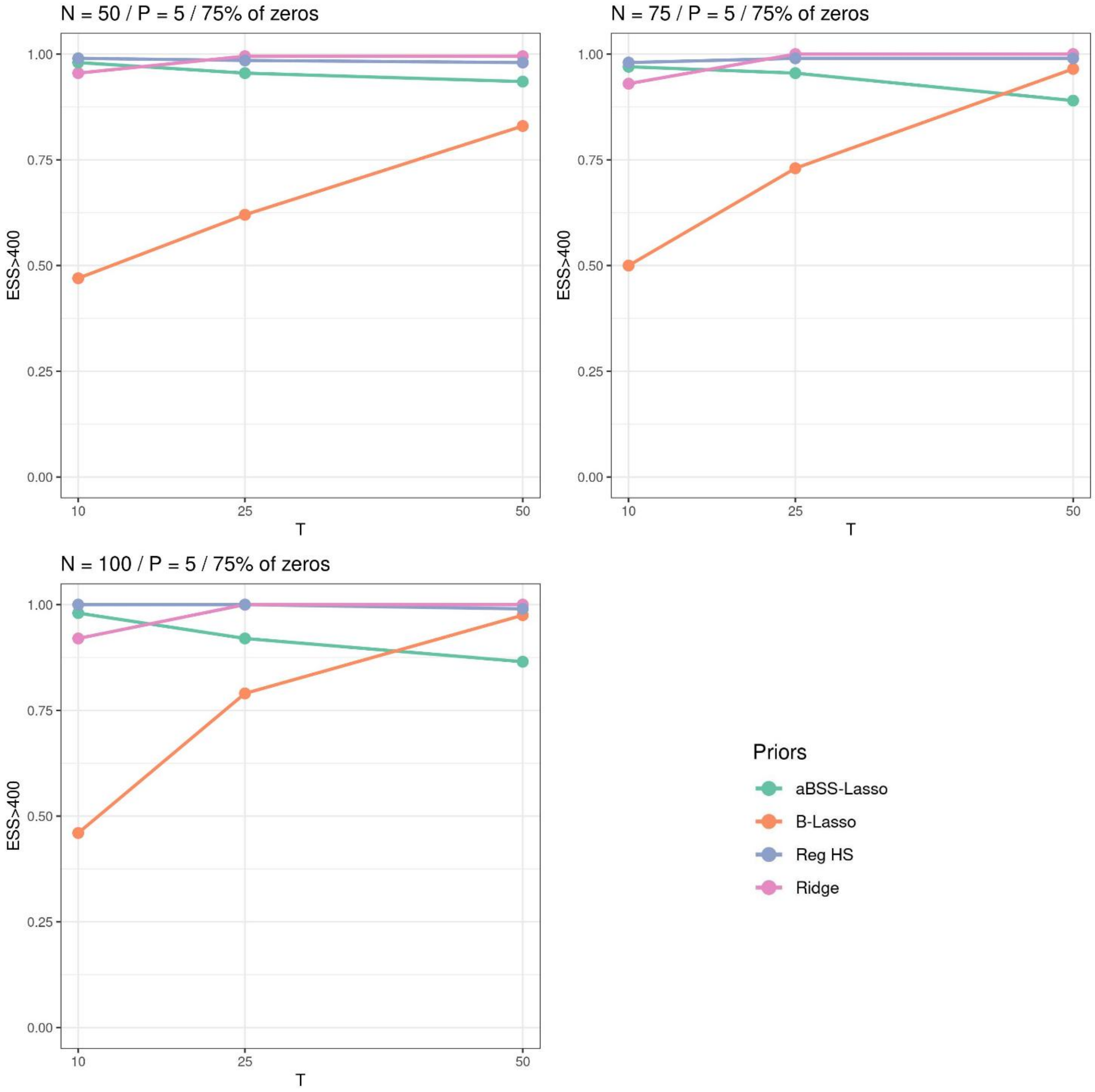

Figure 13: Precision rate of the sampling based on $ESS > 400$ threshold across N and T when $P = 5$ and 75% of the elements of the $\boldsymbol{\beta}^*$ are zeros (simulation study 1).

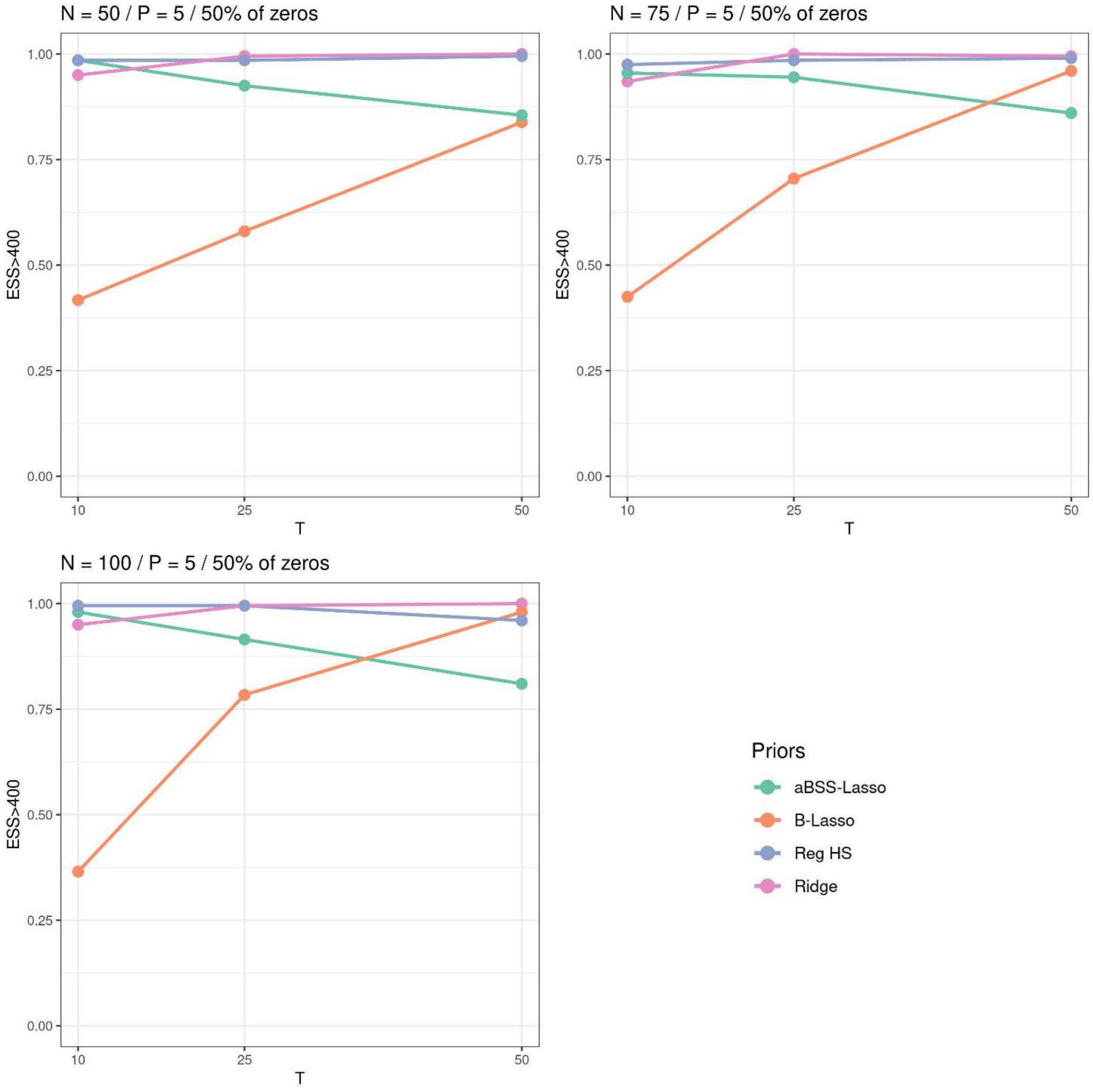

Figure 14: Precision rate of the sampling based on $ESS > 400$ threshold across N and T when $P = 5$ and 50% of the elements of the $\boldsymbol{\beta}^*$ are zeros (simulation study 1).

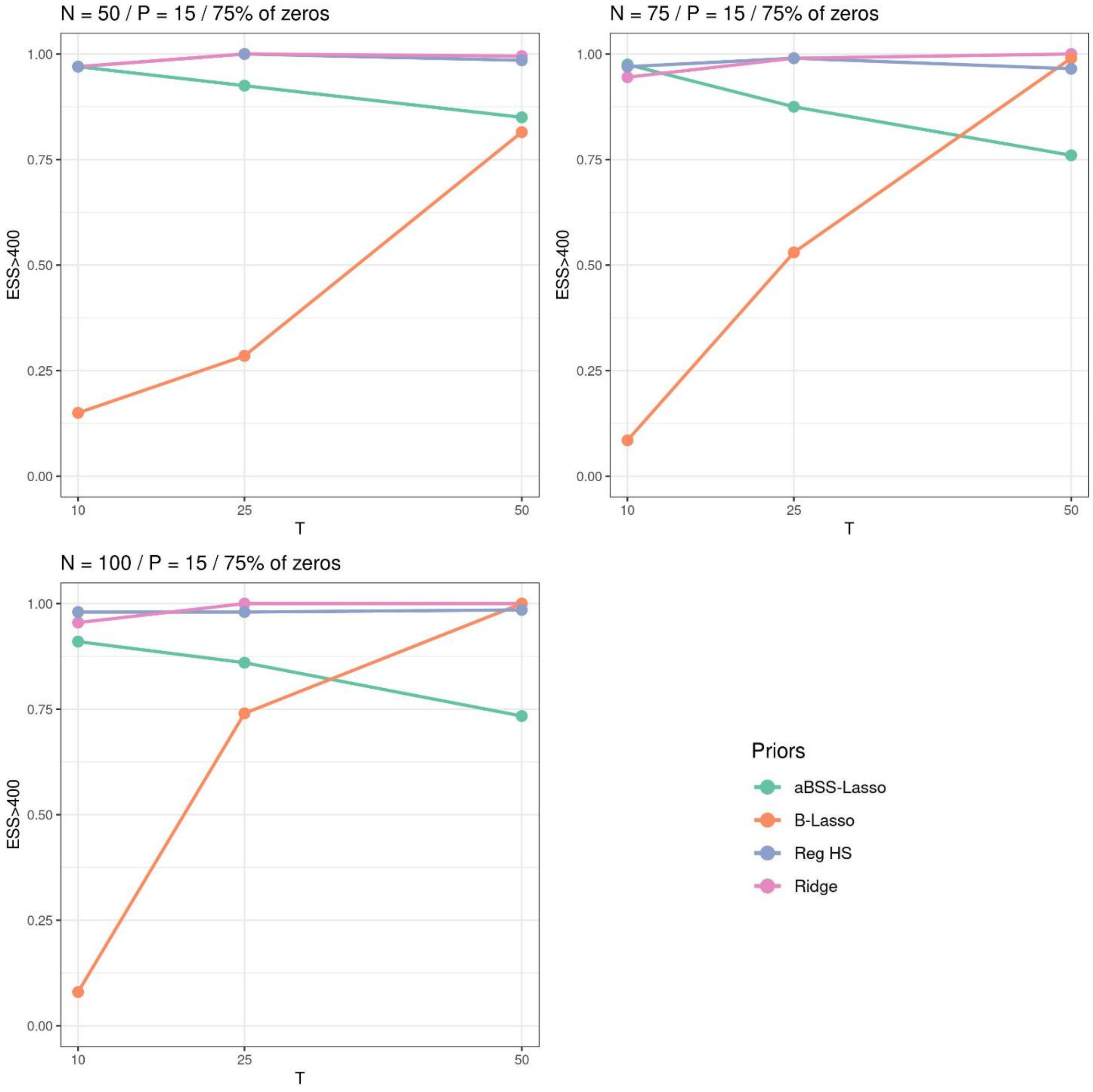

Figure 15: Precision rate of the sampling based on $ESS > 400$ threshold across N and T when $P = 15$ and 75% of the elements of the $\boldsymbol{\beta}^*$ are zeros (simulation study 1).

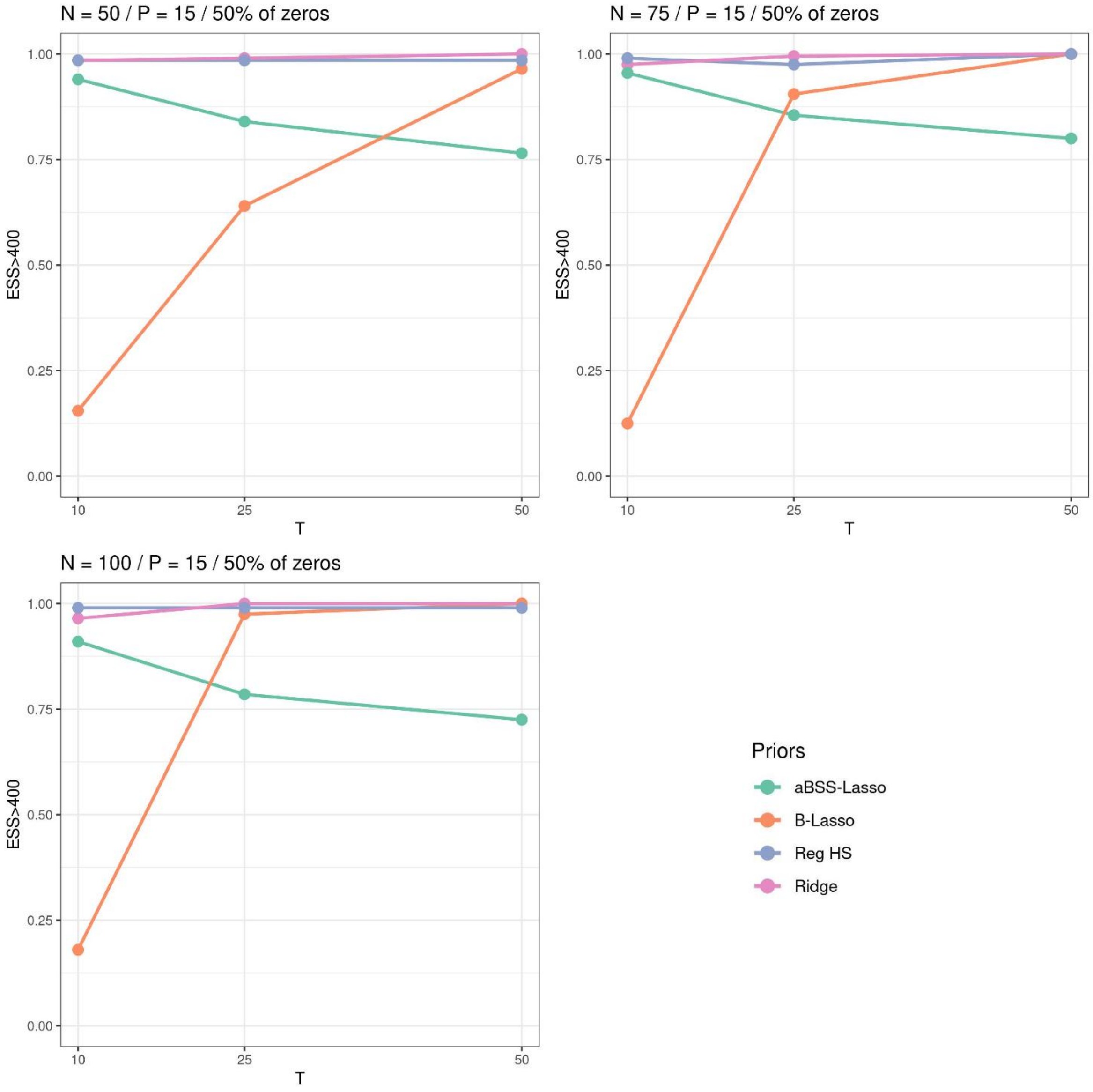

Figure 16: Precision rate of the sampling based on $ESS > 400$ threshold across N and T when $P = 15$ and 50% of the elements of the $\boldsymbol{\beta}^*$ are zeros (simulation study 1).

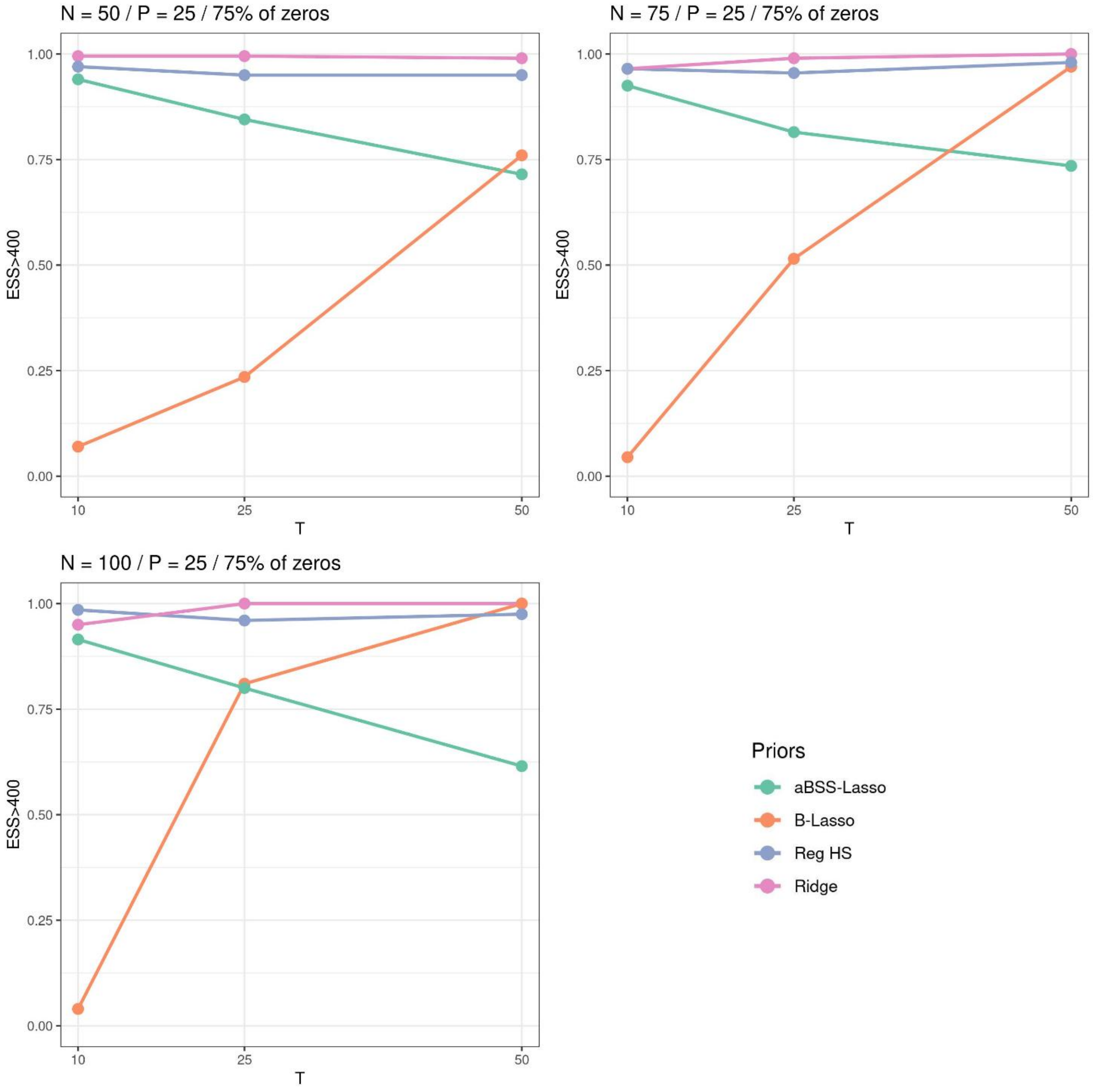

Figure 17: Precision rate of the sampling based on $ESS > 400$ threshold across N and T when $P = 25$ and 75% of the elements of the $\boldsymbol{\beta}^*$ are zeros (simulation study 1).

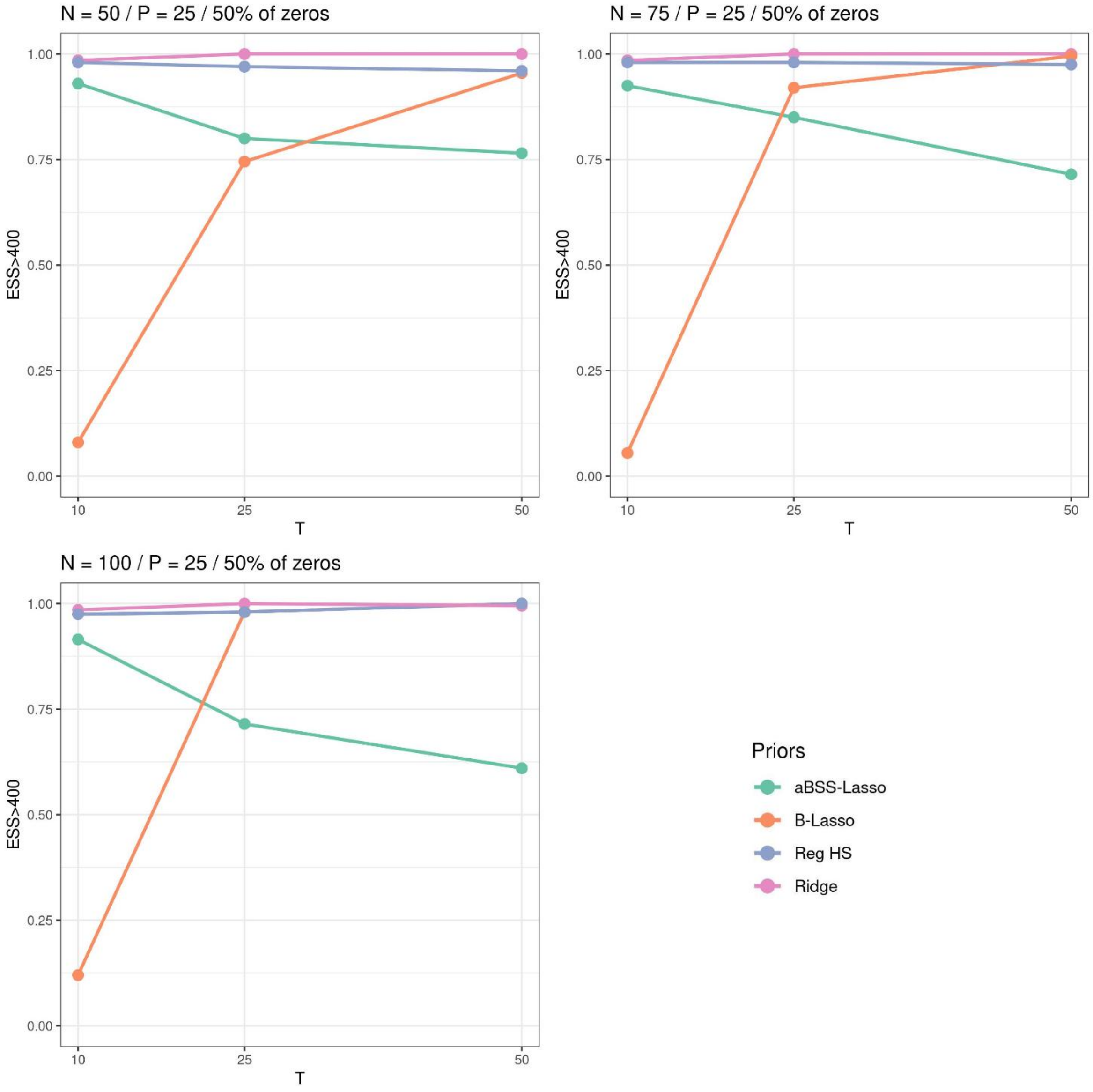

Figure 18: Precision rate of the sampling based on $ESS > 400$ threshold across N and T when $P = 25$ and 50% of the elements of the $\boldsymbol{\beta}^*$ are zeros (simulation study 1).

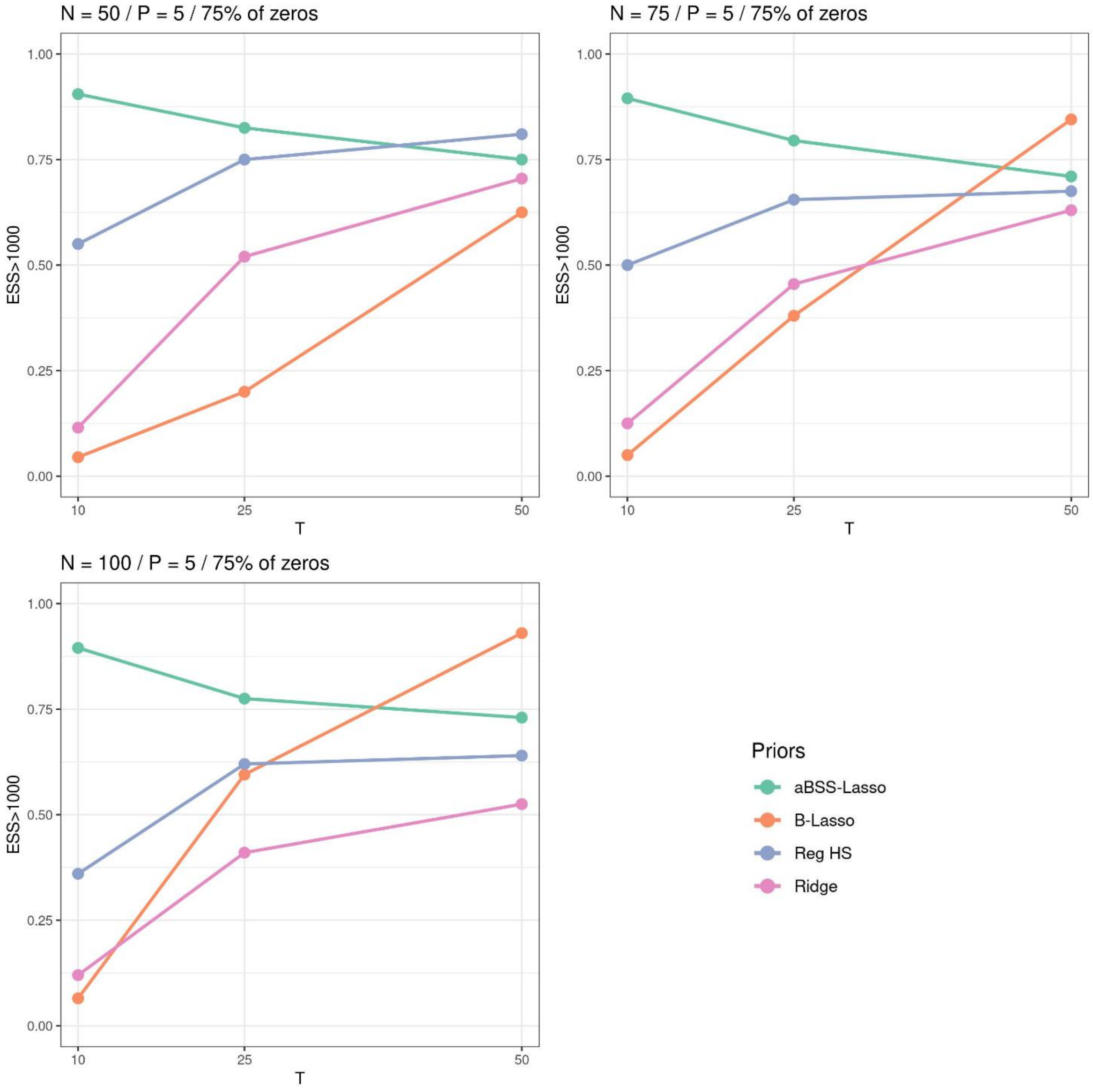

Figure 19: Precision rate of the sampling based on $ESS > 1000$ threshold across N and T when $P = 5$ and 75% of the elements of the $\boldsymbol{\beta}^*$ are zeros (simulation study 1).

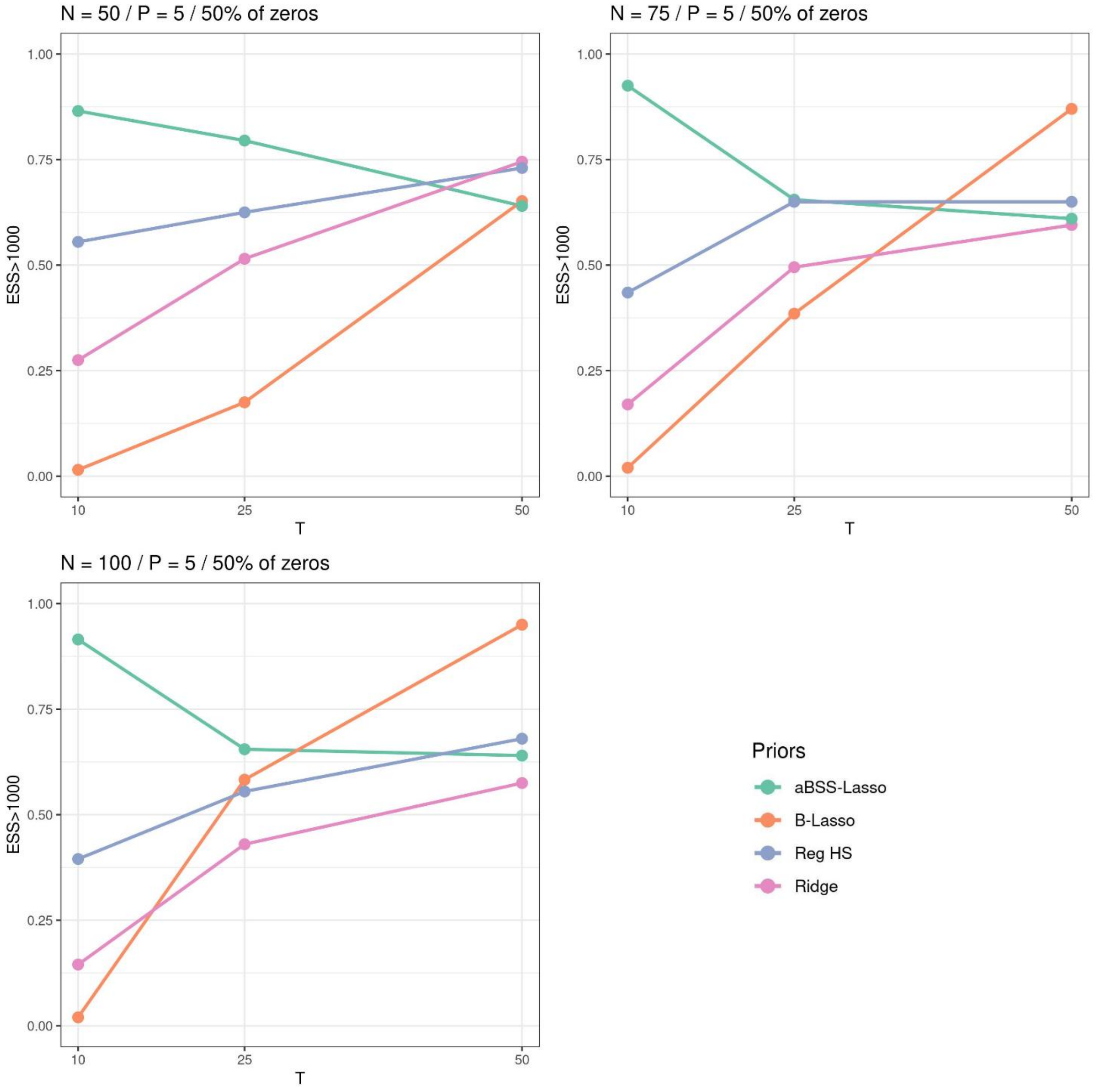

Figure 20: Precision rate of the sampling based on $ESS > 1000$ threshold across N and T when $P = 5$ and 50% of the elements of the $\boldsymbol{\beta}^*$ are zeros (simulation study 1).

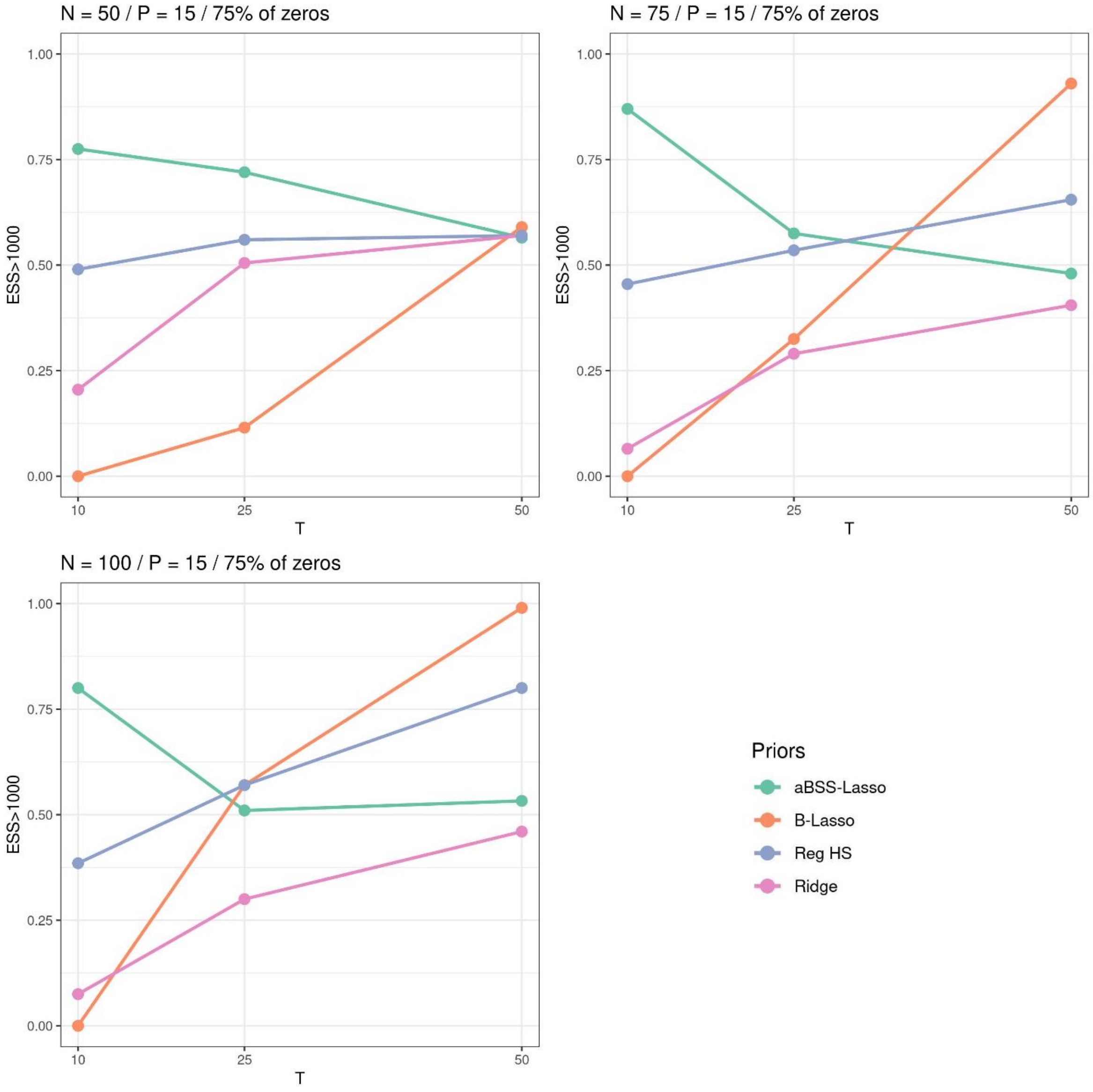

Figure 21: Precision rate of the sampling based on $ESS > 1000$ threshold across N and T when $P = 15$ and 75% of the elements of the $\boldsymbol{\beta}^*$ are zeros (simulation study 1).

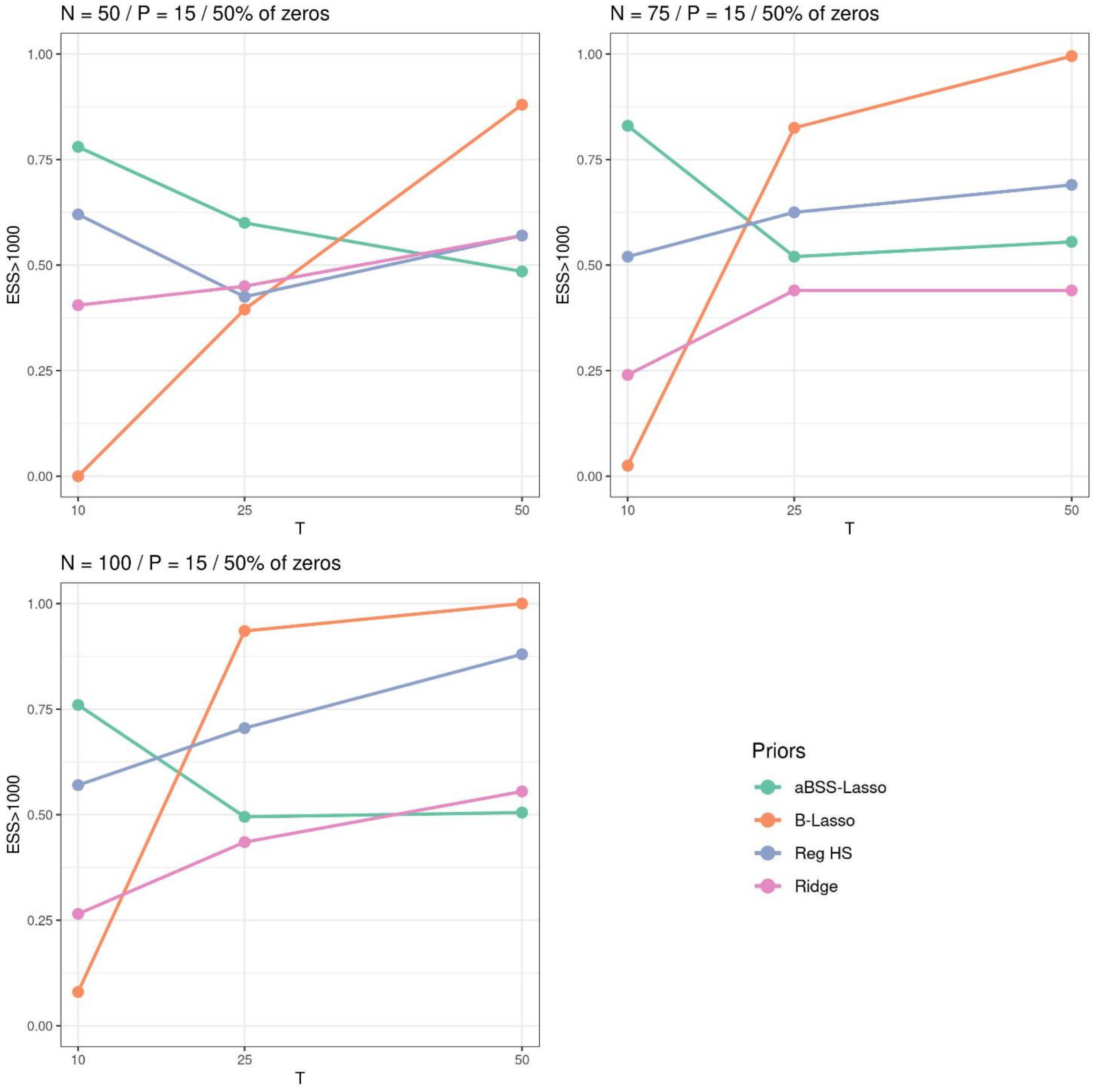

Figure 22: Precision rate of the sampling based on $ESS > 1000$ threshold across N and T when $P = 15$ and 50% of the elements of the $\boldsymbol{\beta}^*$ are zeros (simulation study 1).

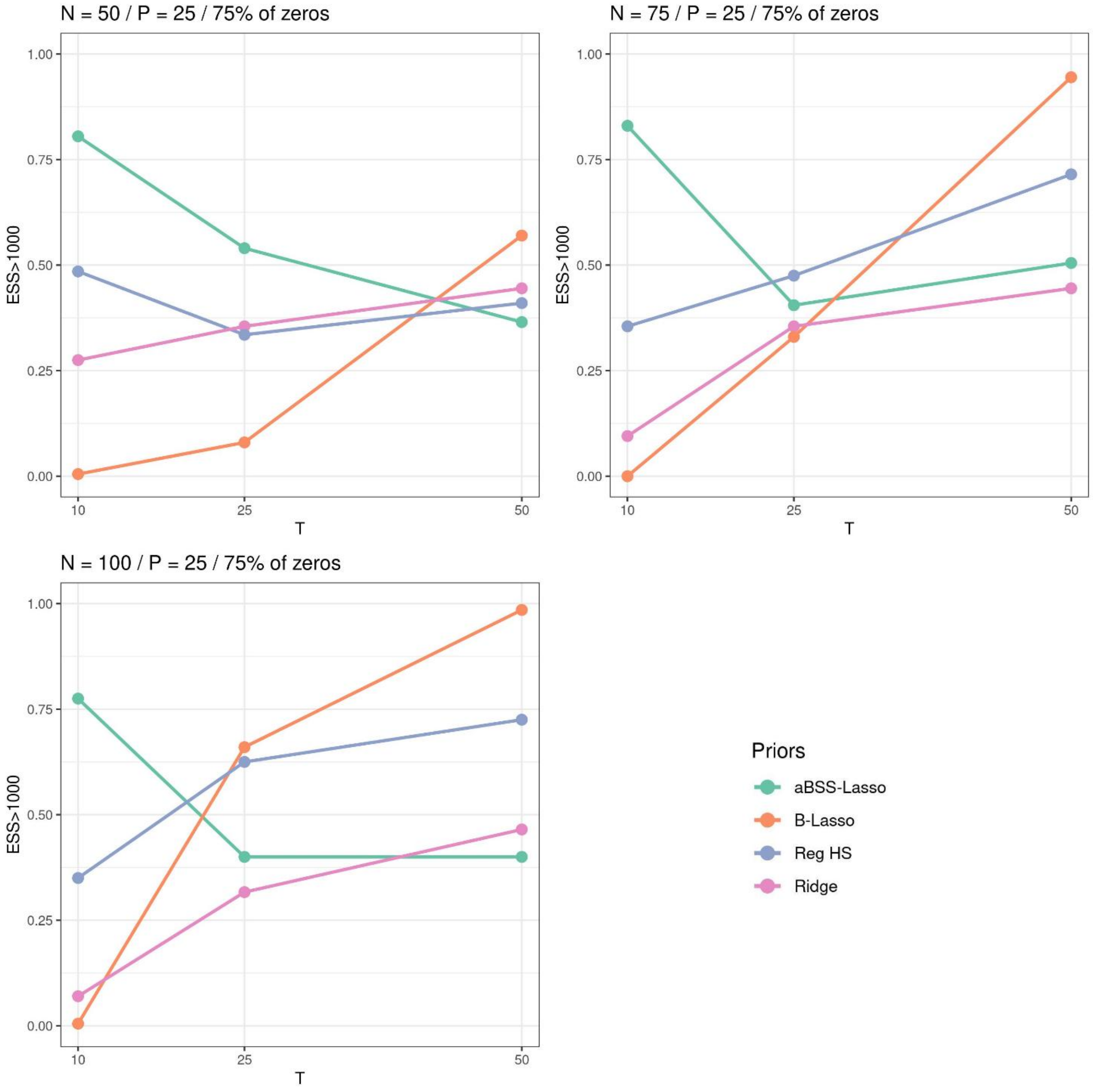

Figure 23: Precision rate of the sampling based on $ESS > 1000$ threshold across N and T when $P = 25$ and 75% of the elements of the $\boldsymbol{\beta}^*$ are zeros (simulation study 1).

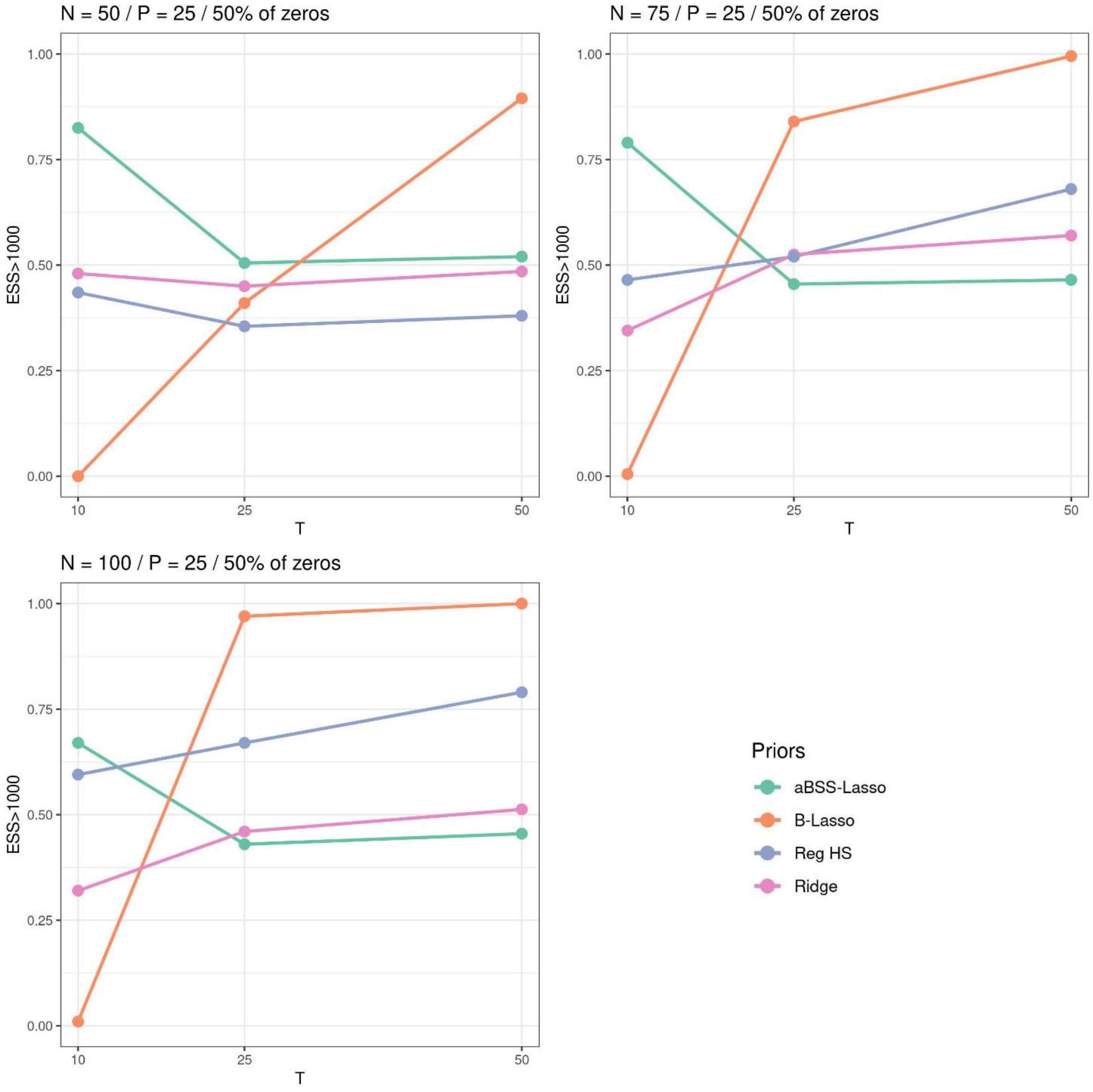

Figure 24: Precision rate of the sampling based on $ESS > 1000$ threshold across N and T when $P = 25$ and 50% of the elements of the $\boldsymbol{\beta}^*$ are zeros (simulation study 1).

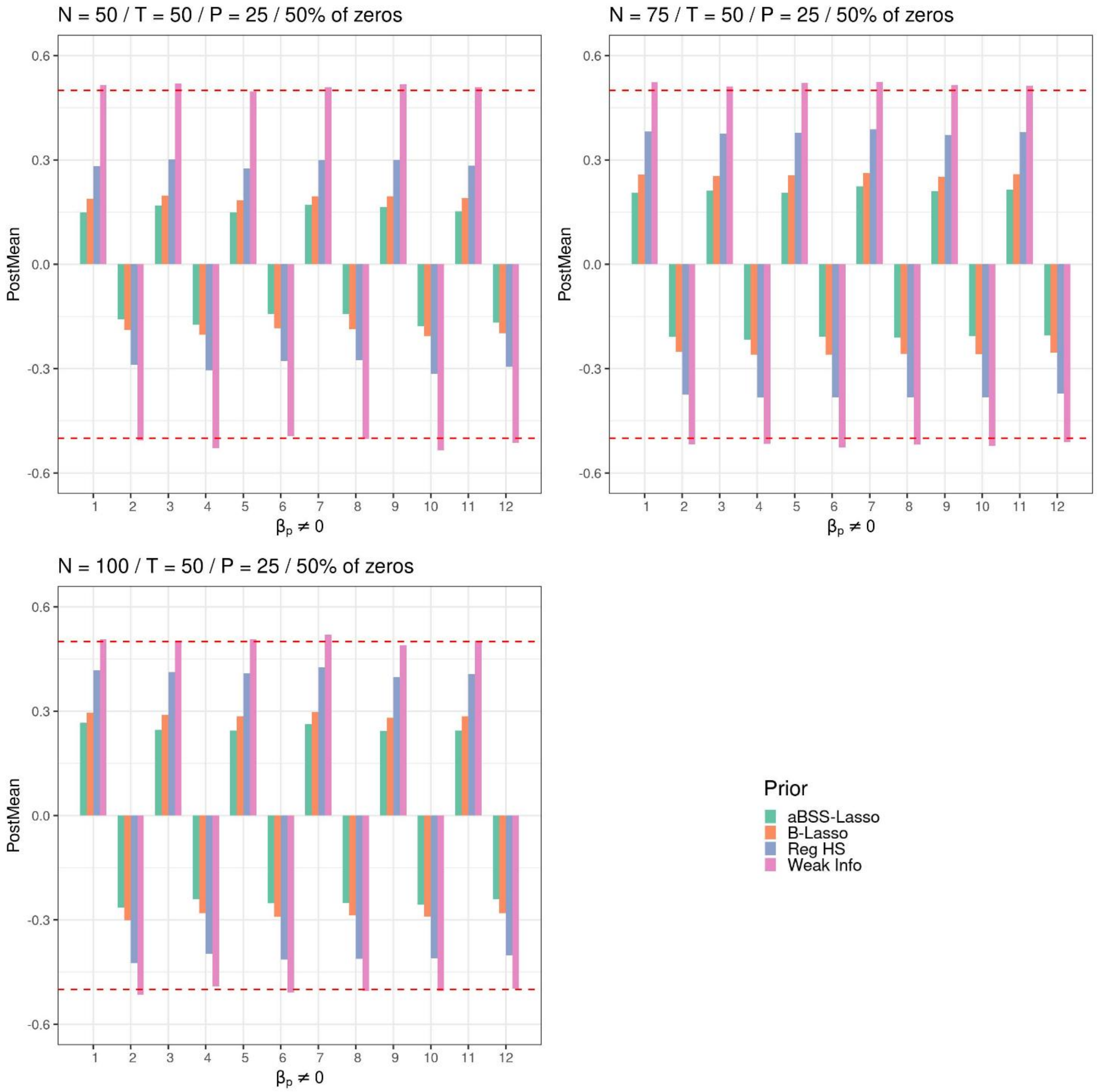

Figure 25: Average Posterior means (for the non- zero coeffcients) across N when $T = 50$, $P = 25$ and 50% of the elements of the $\boldsymbol{\beta}^*$ are zeros (simulation study 1).

1

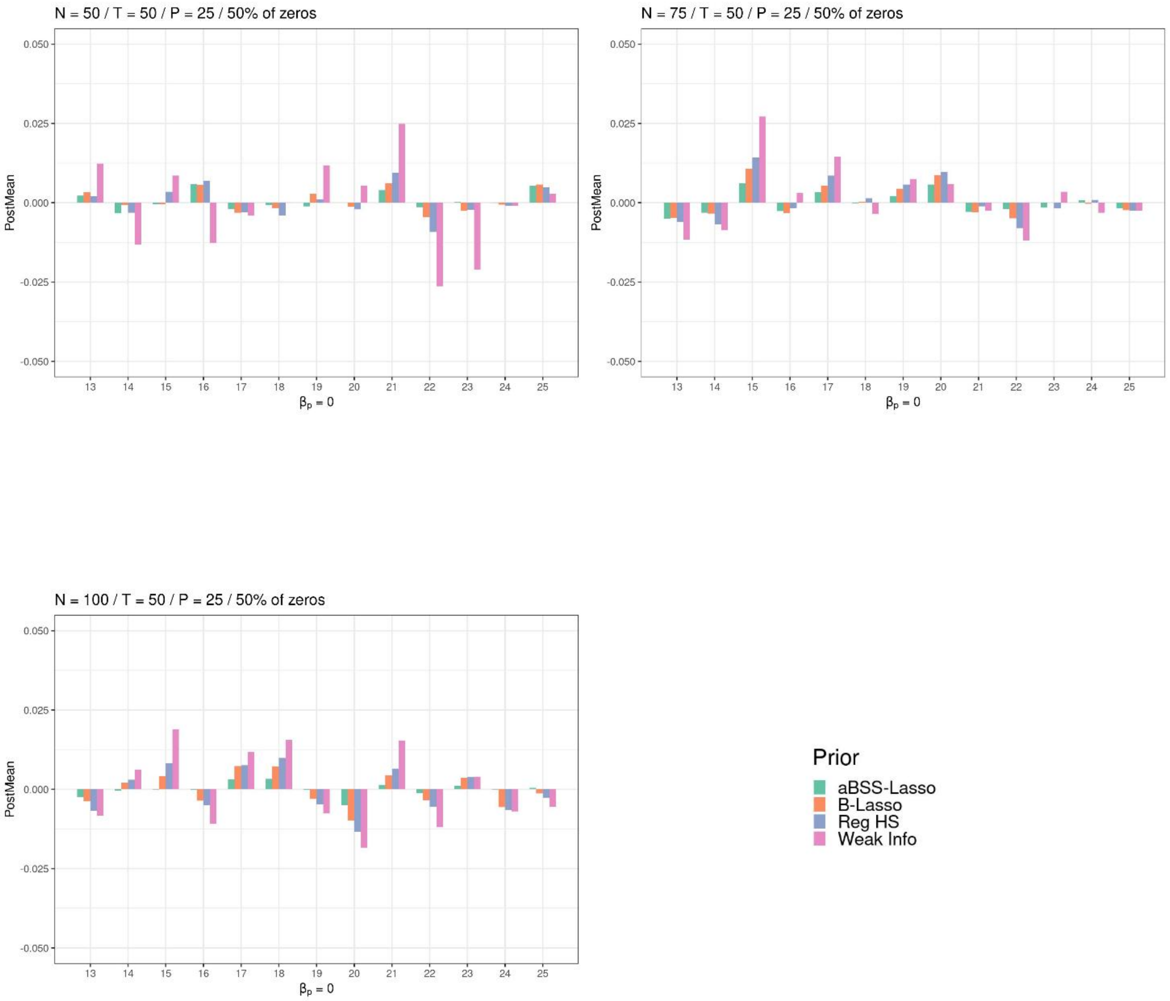

Figure 26: Average Posterior means (for the zero coeffcients) across N when $T = 50$, $P = 25$ and 50% of the elements of the $\boldsymbol{\beta}^*$ are zeros (simulation study 1).

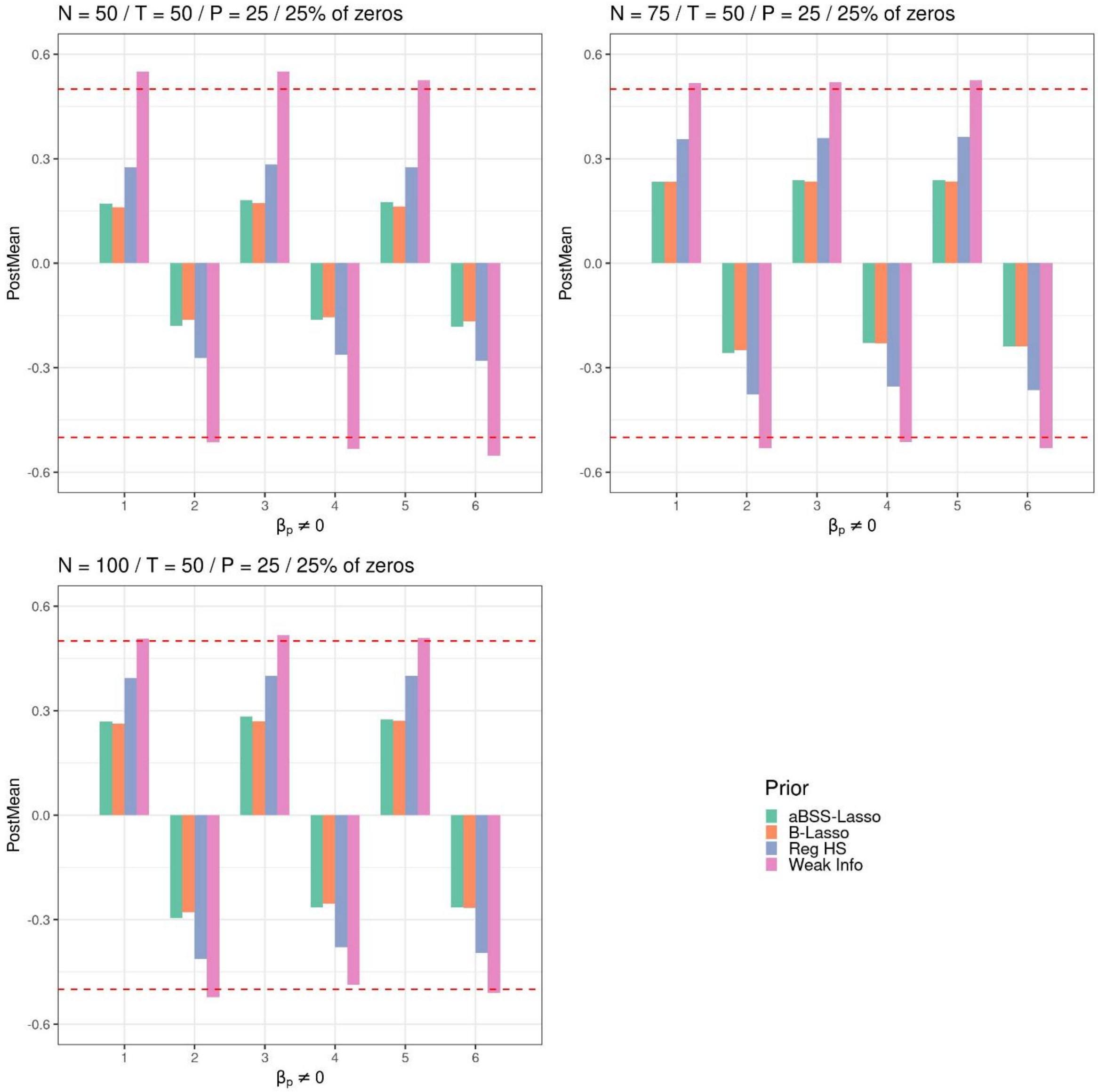

Figure 27: Average Posterior means (for the non- zero coeffcients) across N when $T = 50$, $P = 25$ and 75% of the elements of the $\boldsymbol{\beta}^*$ are zeros (simulation study 1).

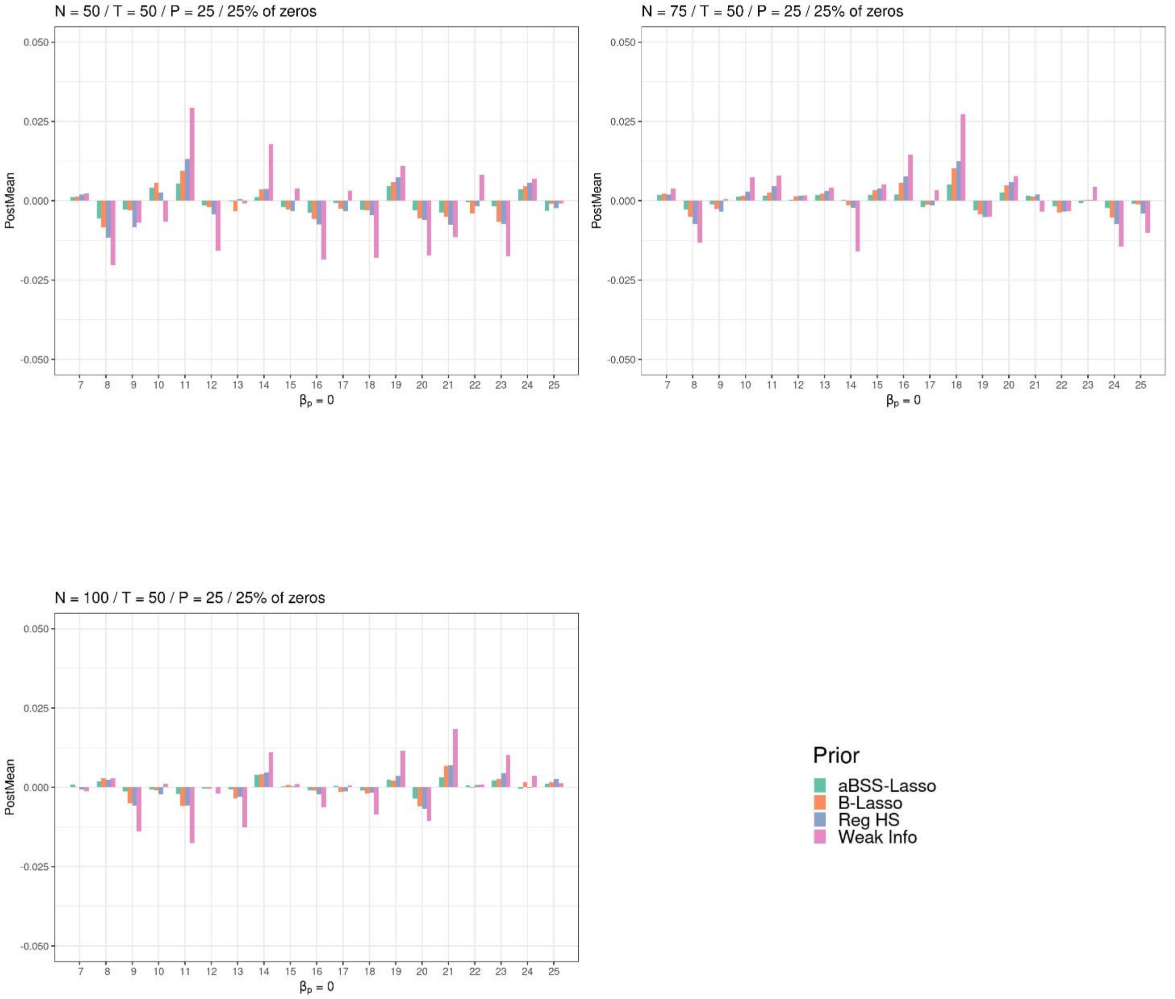

Figure 28: Average Posterior means (for the zero coeffcients) across N when $T = 50$, $P = 25$ and 75% of the elements of the $\boldsymbol{\beta}^*$ are zeros (simulation study 1).

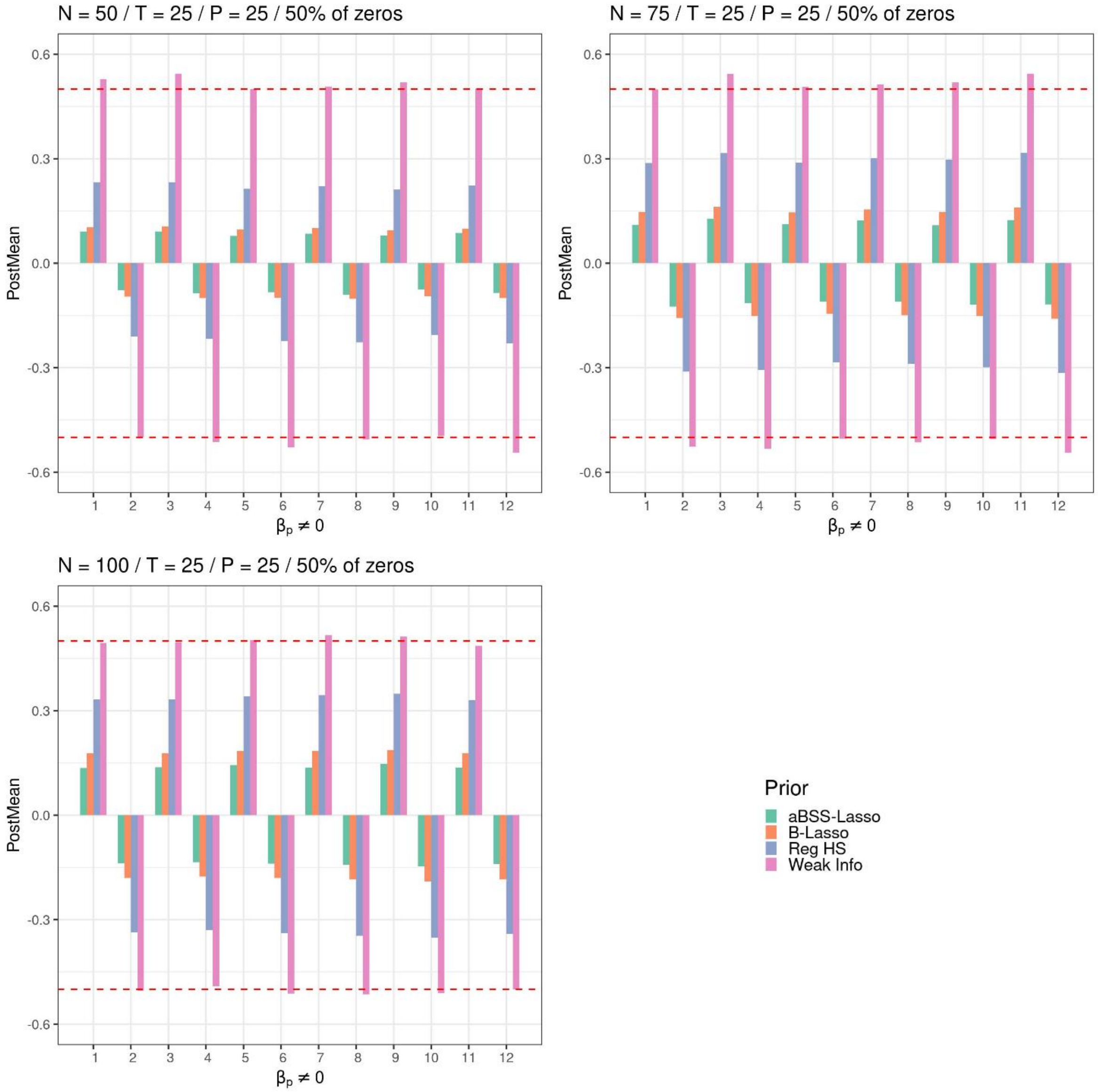

Figure 29: Average Posterior means (for the non- zero coeffcients) across N when $T = 25$, $P = 25$ and 50% of the elements of the $\boldsymbol{\beta}^*$ are zeros (simulation study 1).

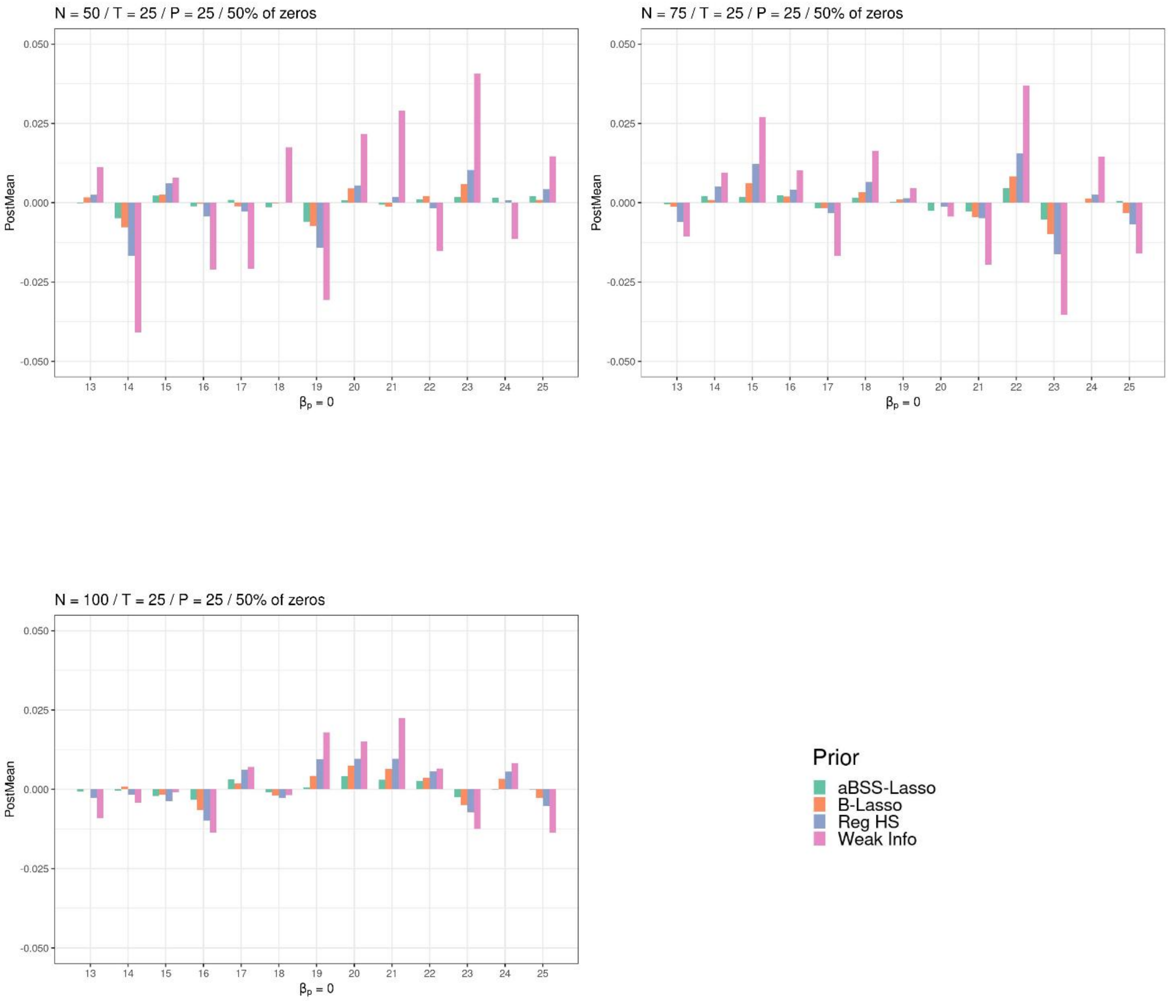

Figure 30: Average Posterior means (for the zero coeffcients) across N when $T = 25$, $P = 25$ and 50% of the elements of the $\boldsymbol{\beta}^*$ are zeros (simulation study 1).

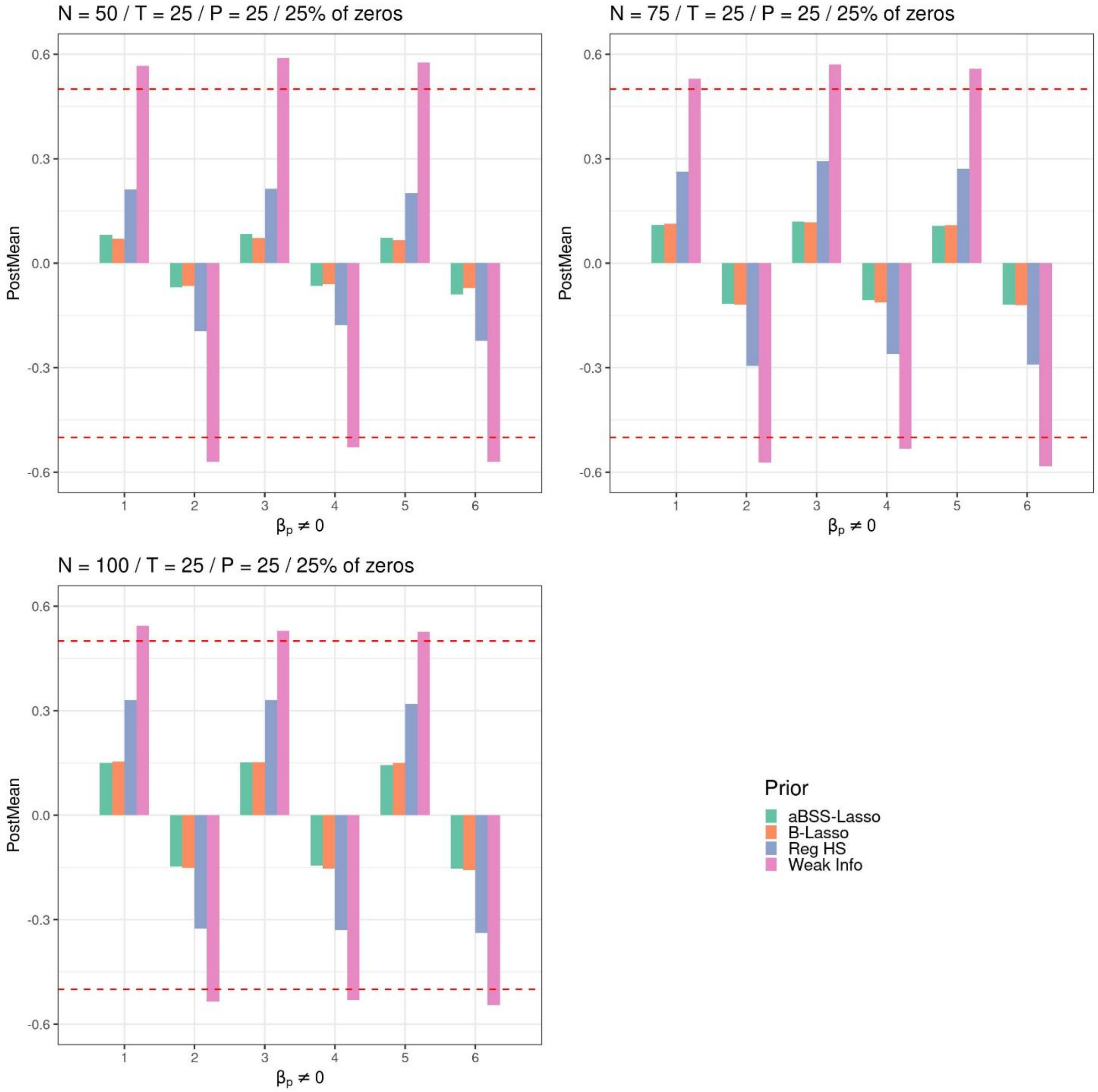

Figure 31: Average Posterior means (for the non- zero coeffcients) across N when $T = 25$, $P = 25$ and 75% of the elements of the $\boldsymbol{\beta}^*$ are zeros (simulation study 1).

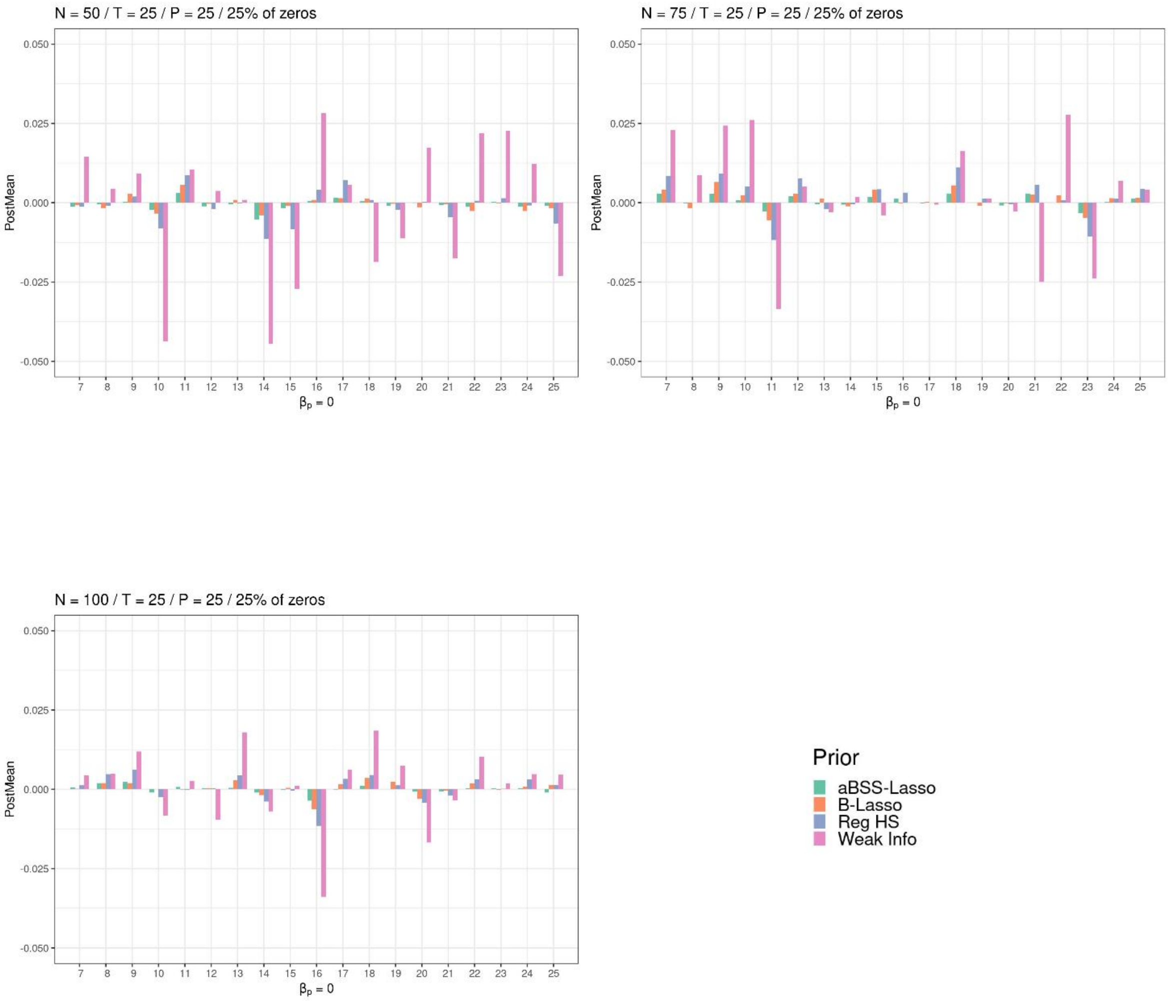

Figure 32: Average Posterior means (for the zero coeffcients) across N when $T = 25$, $P = 25$ and 75% of the elements of the $\boldsymbol{\beta}^*$ are zeros (simulation study 1).

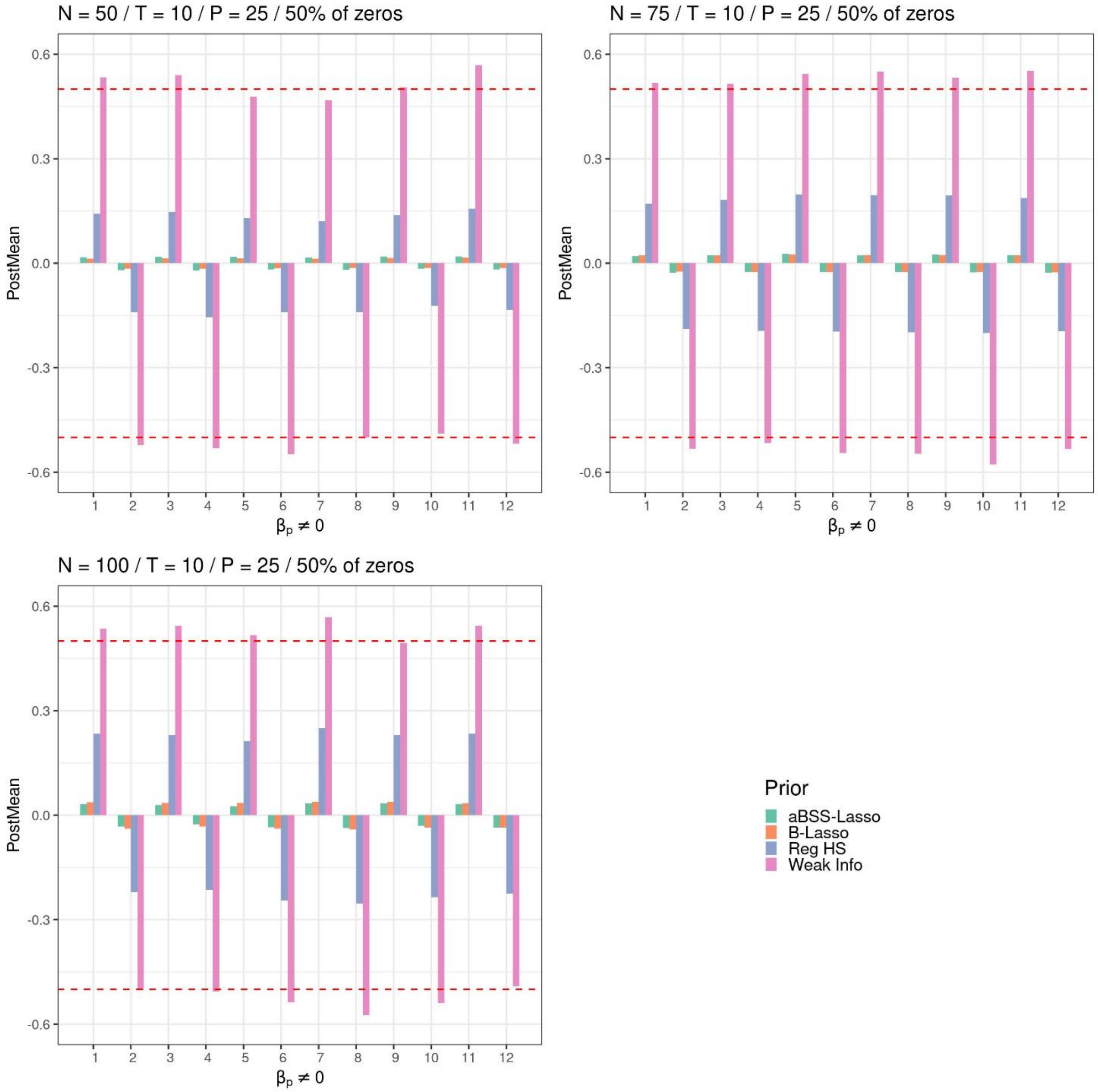

Figure 33: Average Posterior means (for the non- zero coeffcients) across N when $T = 10$, $P = 25$ and 50% of the elements of the $\boldsymbol{\beta}^*$ are zeros (simulation study 1).

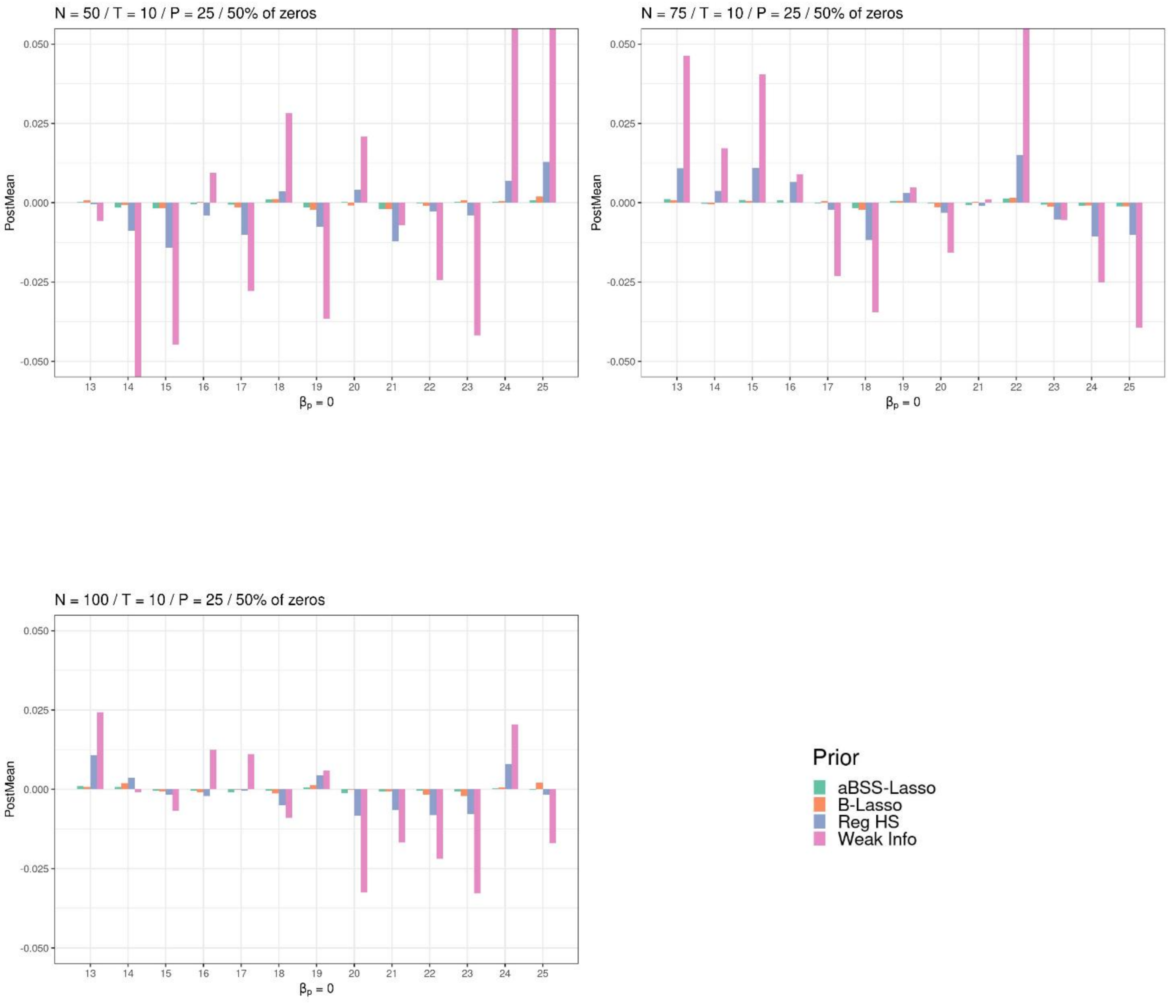

Figure 34: Average Posterior means (for the zero coeffcients) across N when $T = 10$, $P = 25$ and 50% of the elements of the $\boldsymbol{\beta}^*$ are zeros (simulation study 1).

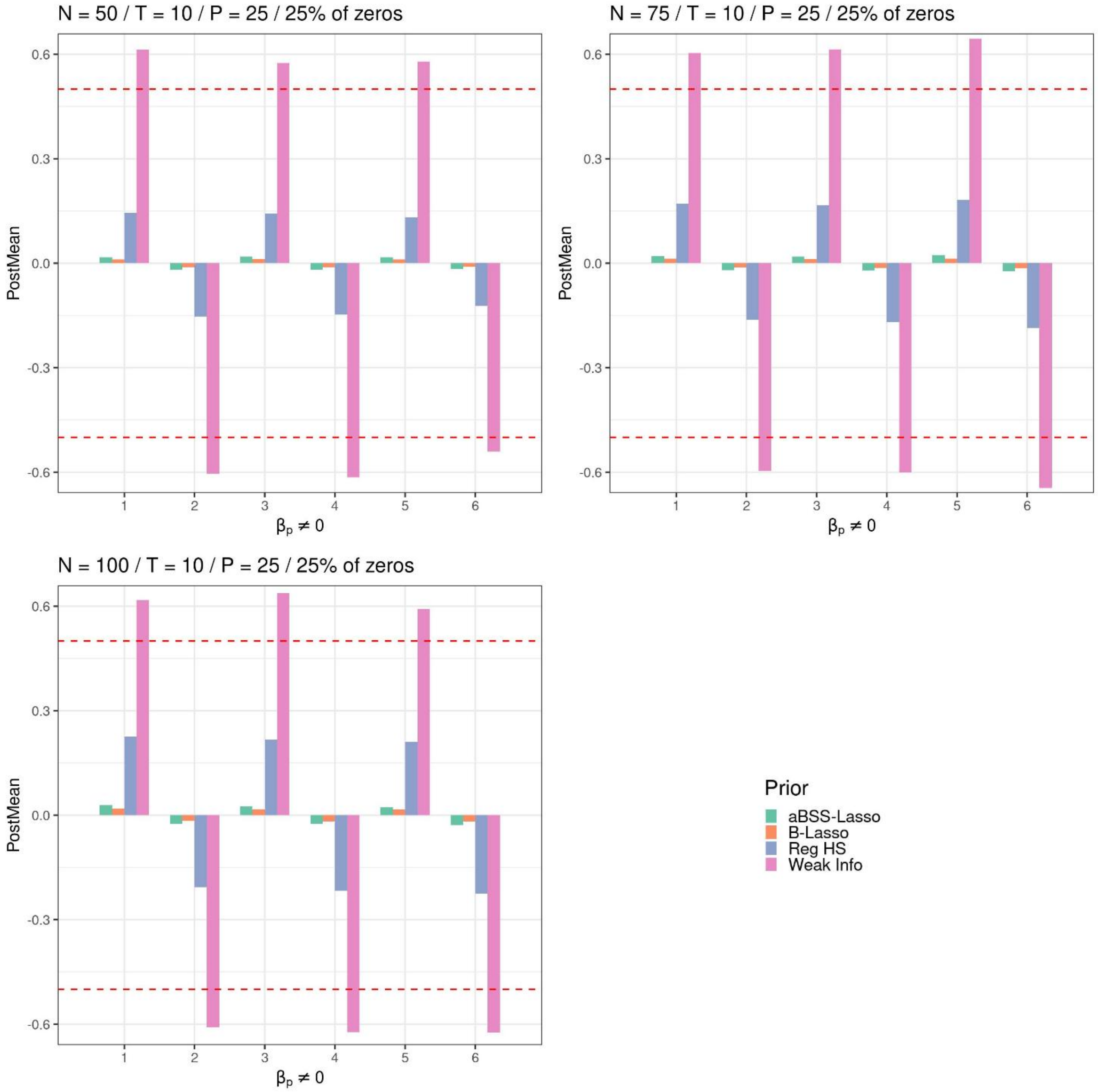

Figure 35: Average Posterior means (for the non- zero coeffcients) across N when $T = 10$, $P = 25$ and 75% of the elements of the $\boldsymbol{\beta}^*$ are zeros (simulation study 1).

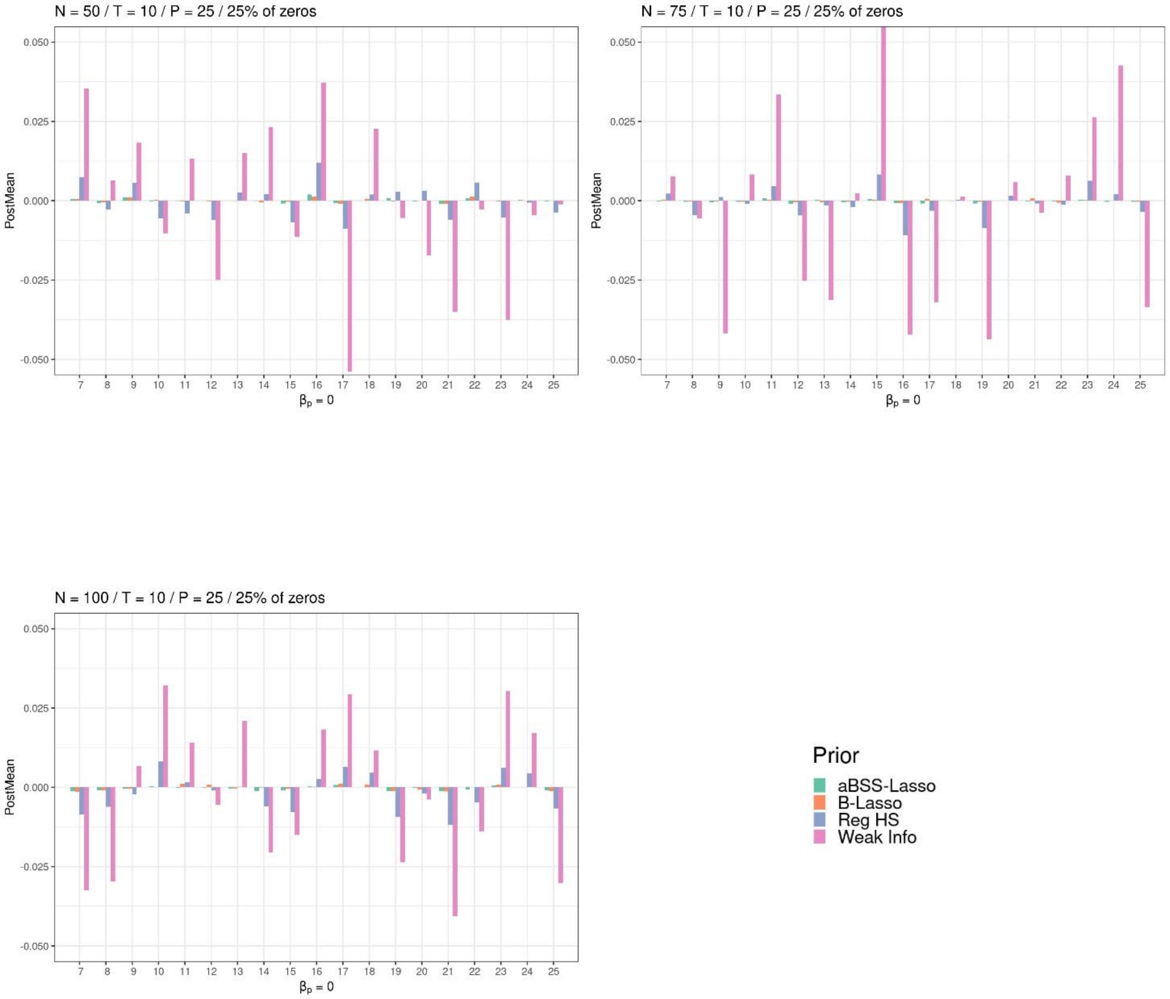

Figure 36: Average Posterior means (for the zero coeffcients) across N when $T = 10$, $P = 25$ and 75% of the elements of the $\boldsymbol{\beta}^*$ are zeros (simulation study 1).

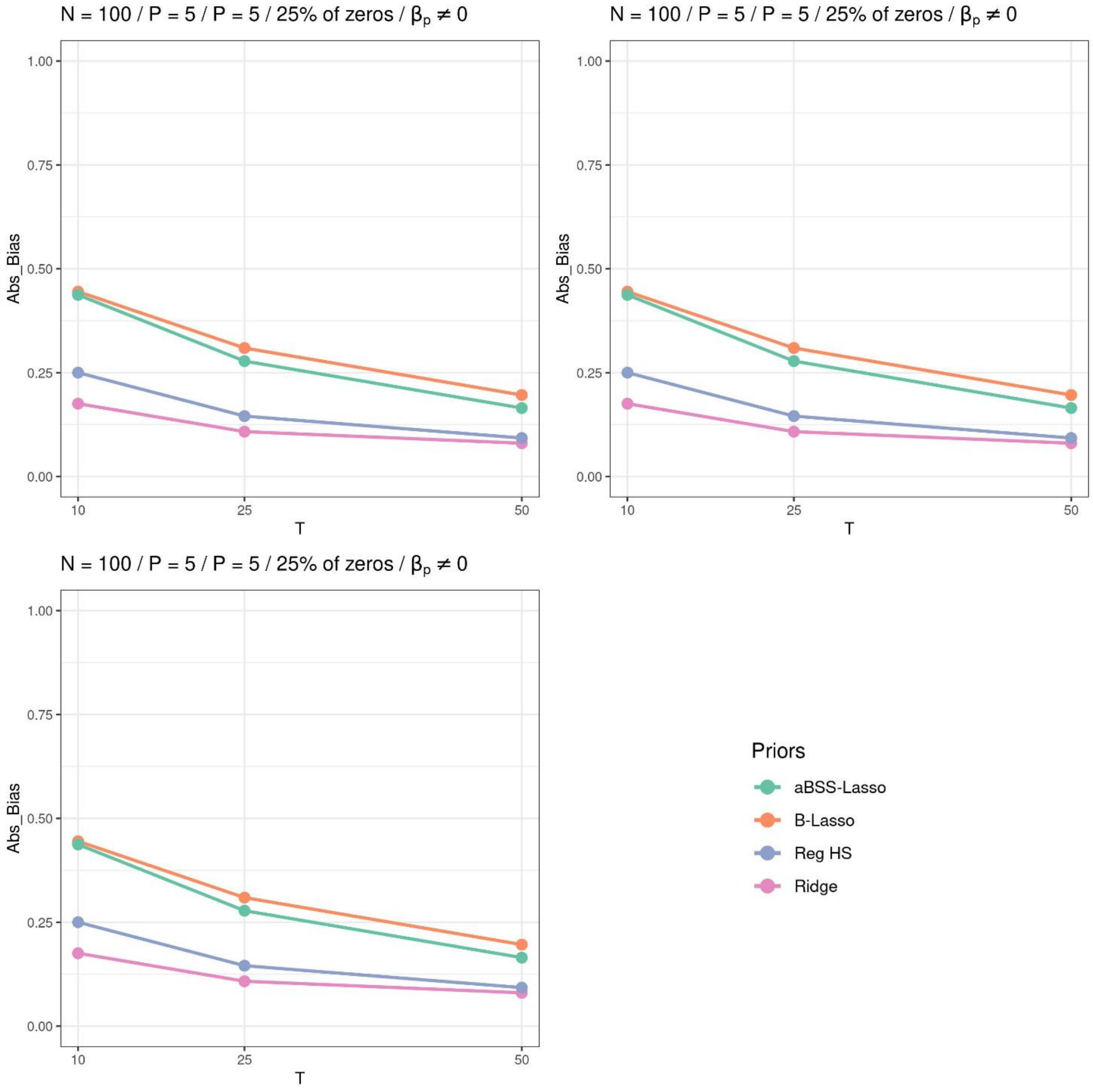

Figure 37: Absolute Bias (for non-zero coeffcients) across N and T when $P = 5$ and 75% of the elements of the $\boldsymbol{\beta}^*$ are zeros (simulation study 1).

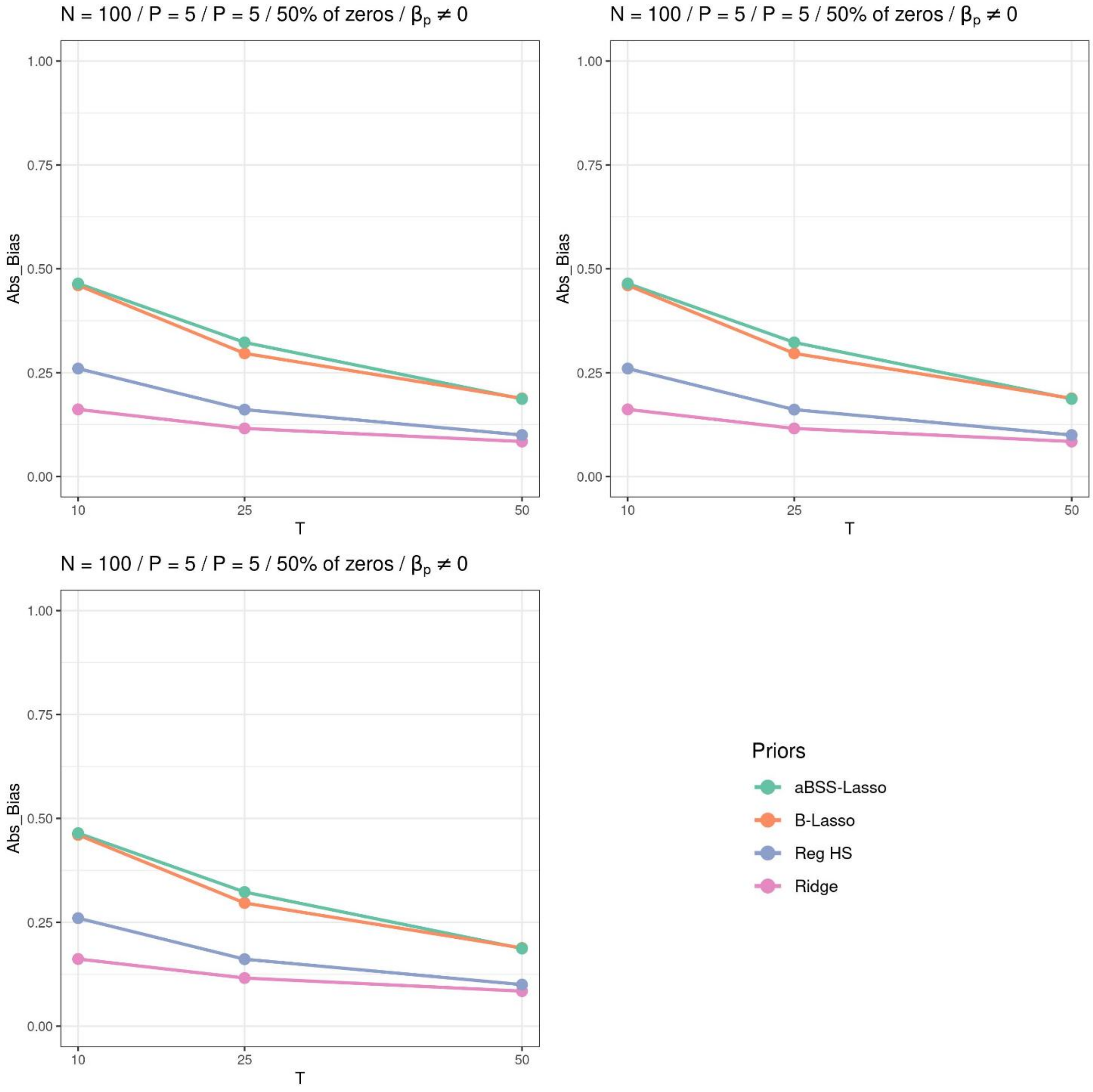

Figure 38: Absolute Bias (for non-zero coeffcients) across N and T when $P = 5$ and 50% of the elements of the $\boldsymbol{\beta}^*$ are zeros (simulation study 1).

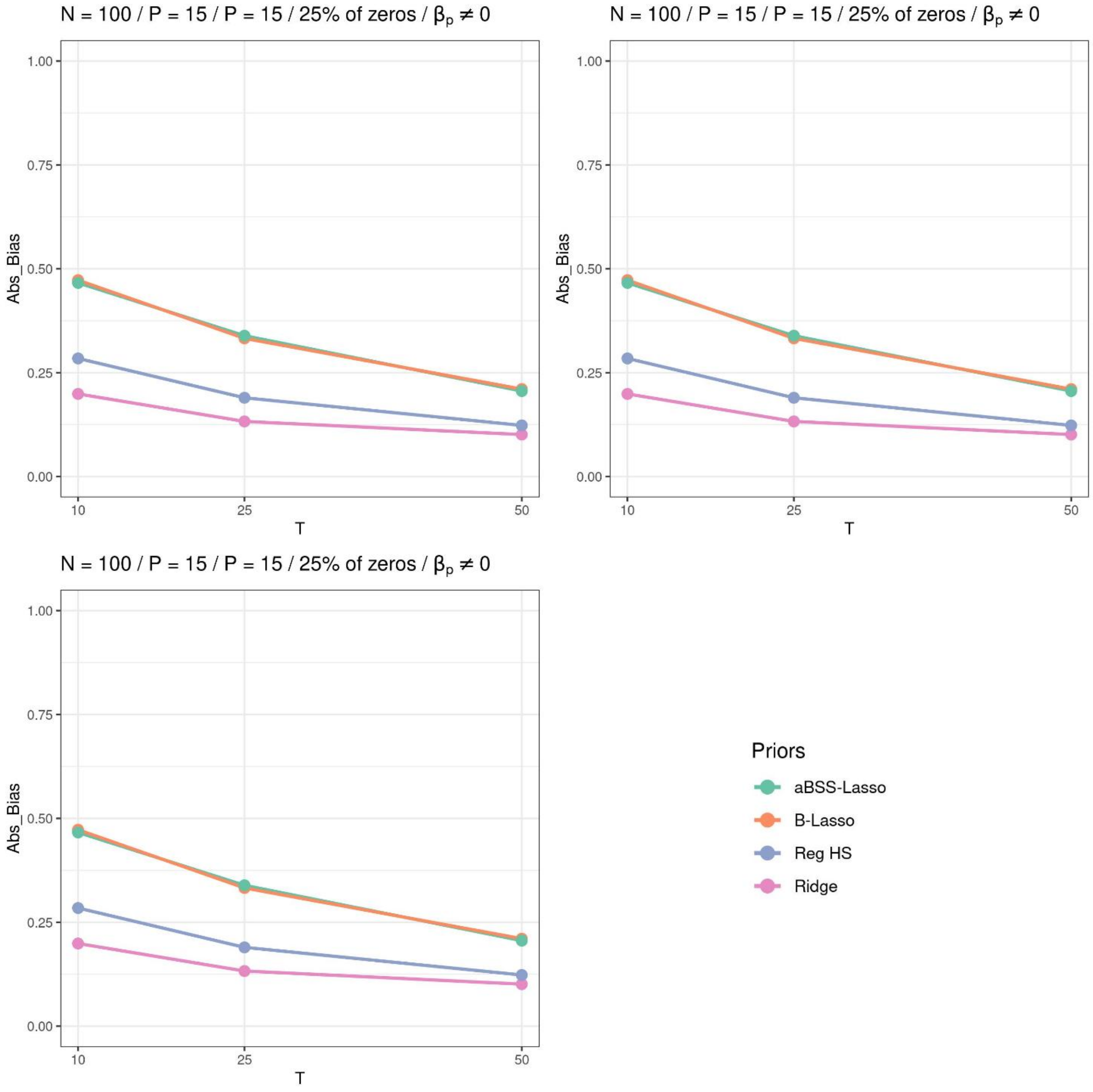

Figure 39: Absolute Bias (for non-zero coeffcients) across N and T when $P = 15$ and 75% of the elements of the $\boldsymbol{\beta}^*$ are zeros (simulation study 1).

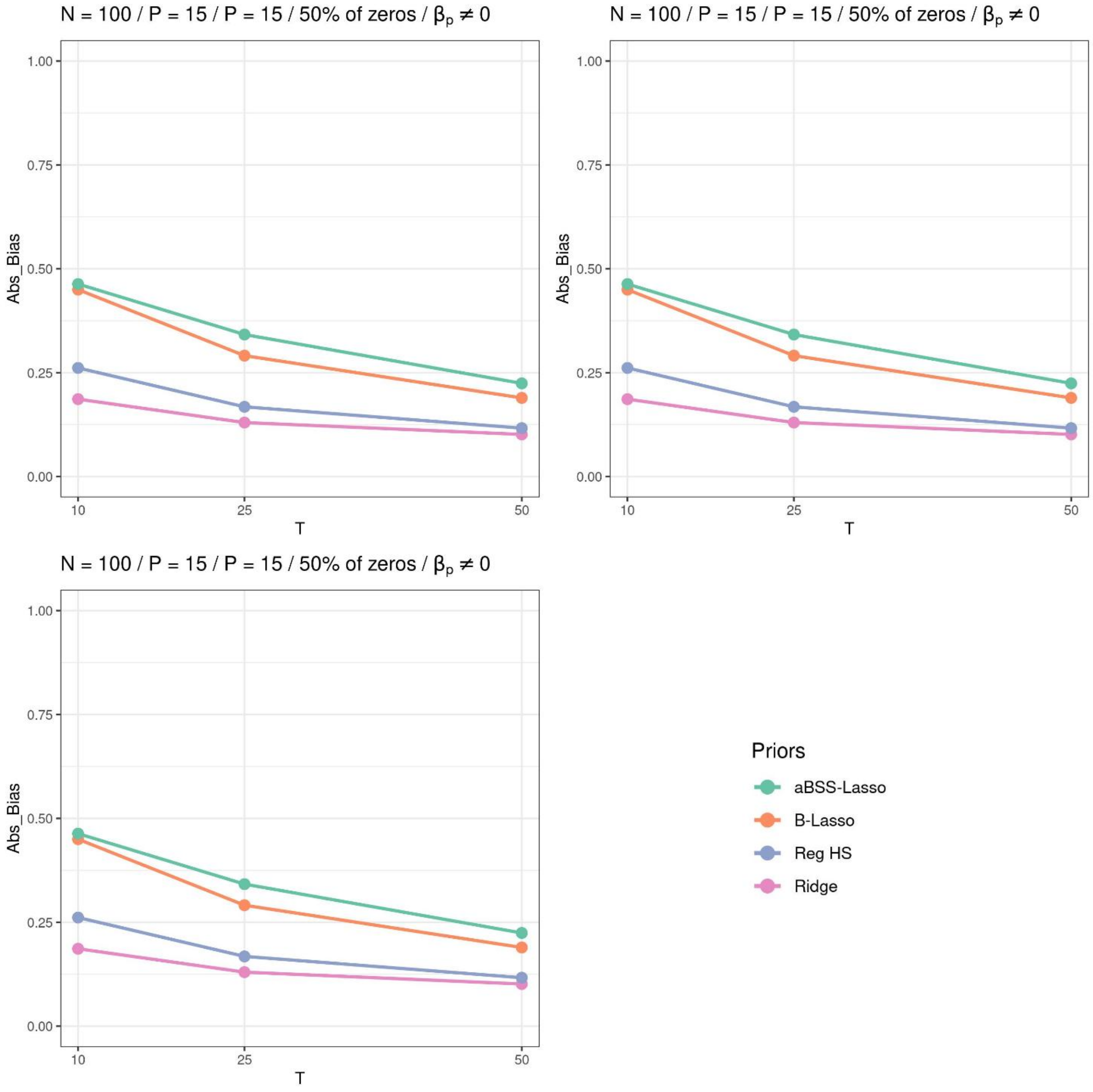

Figure 40: Absolute Bias (for non-zero coeffcients) across N and T when $P = 15$ and 50% of the elements of the $\boldsymbol{\beta}^*$ are zeros (simulation study 1).

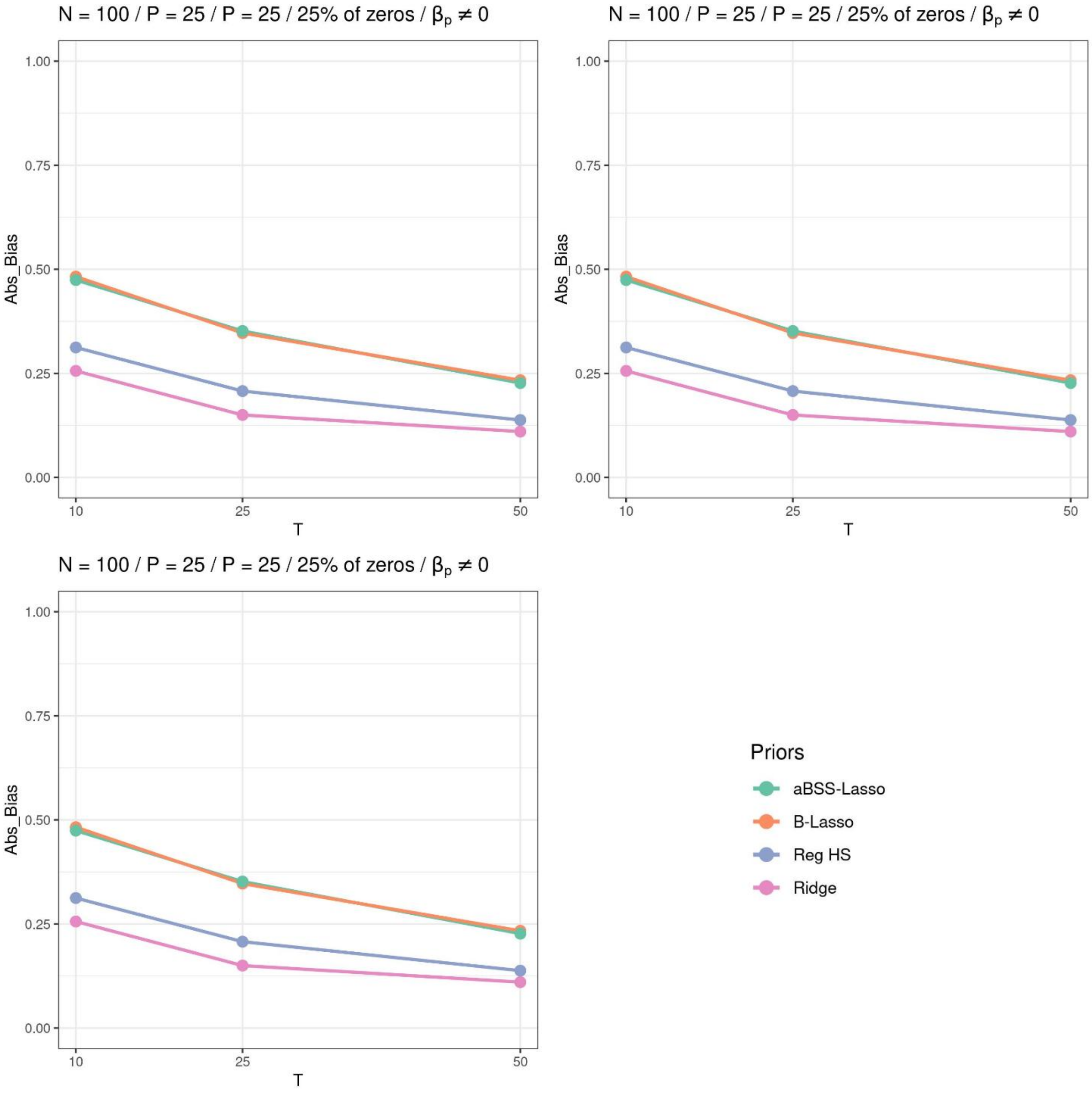

Figure 41: Absolute Bias (for non-zero coeffcients) across N and T when $P = 25$ and 75% of the elements of the $\boldsymbol{\beta}^*$ are zeros (simulation study 1).

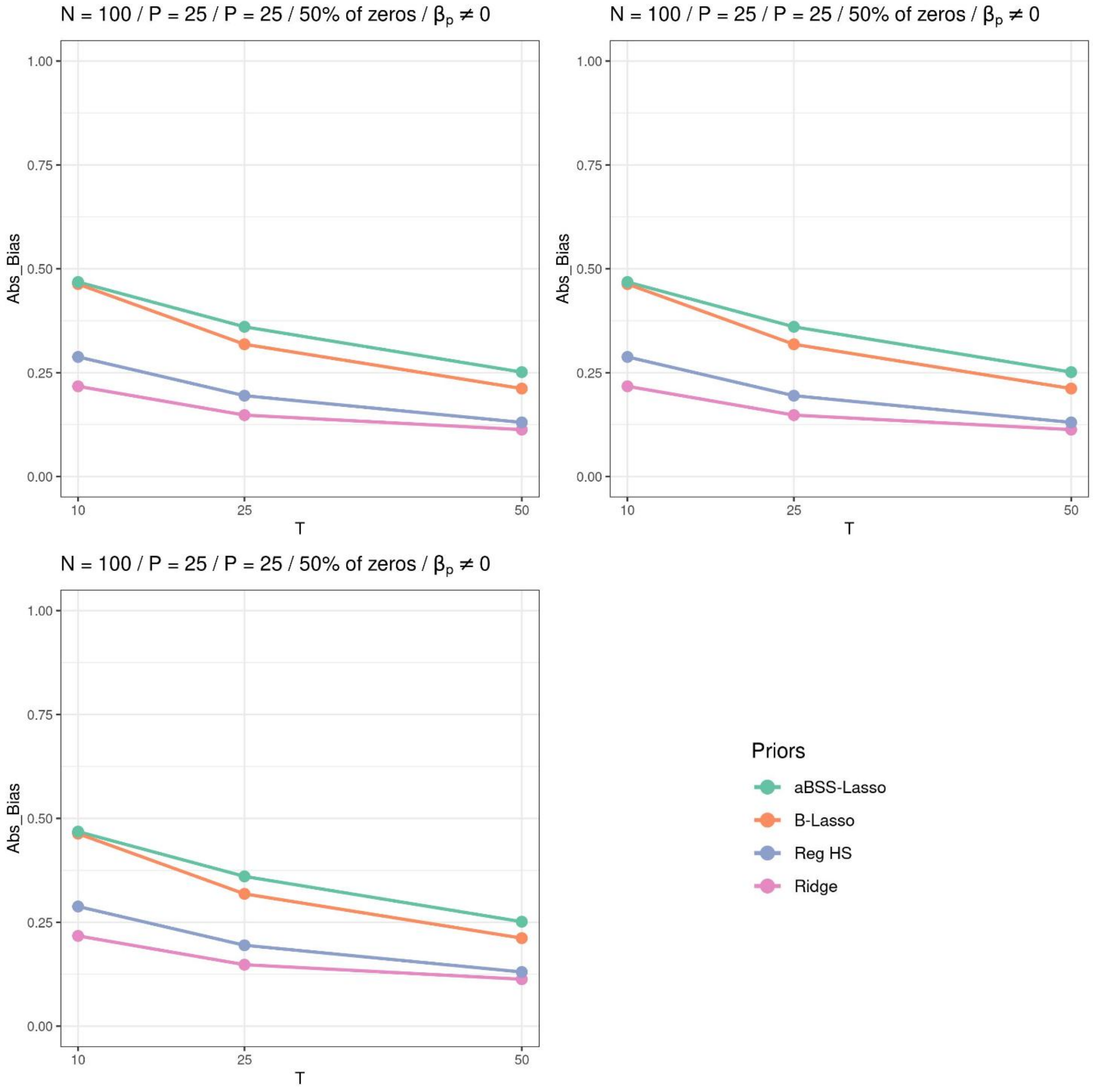

Figure 42: Absolute Bias (for non-zero coeffcients) across N and T when $P = 25$ and 50% of the elements of the $\boldsymbol{\beta}^*$ are zeros (simulation study 1).

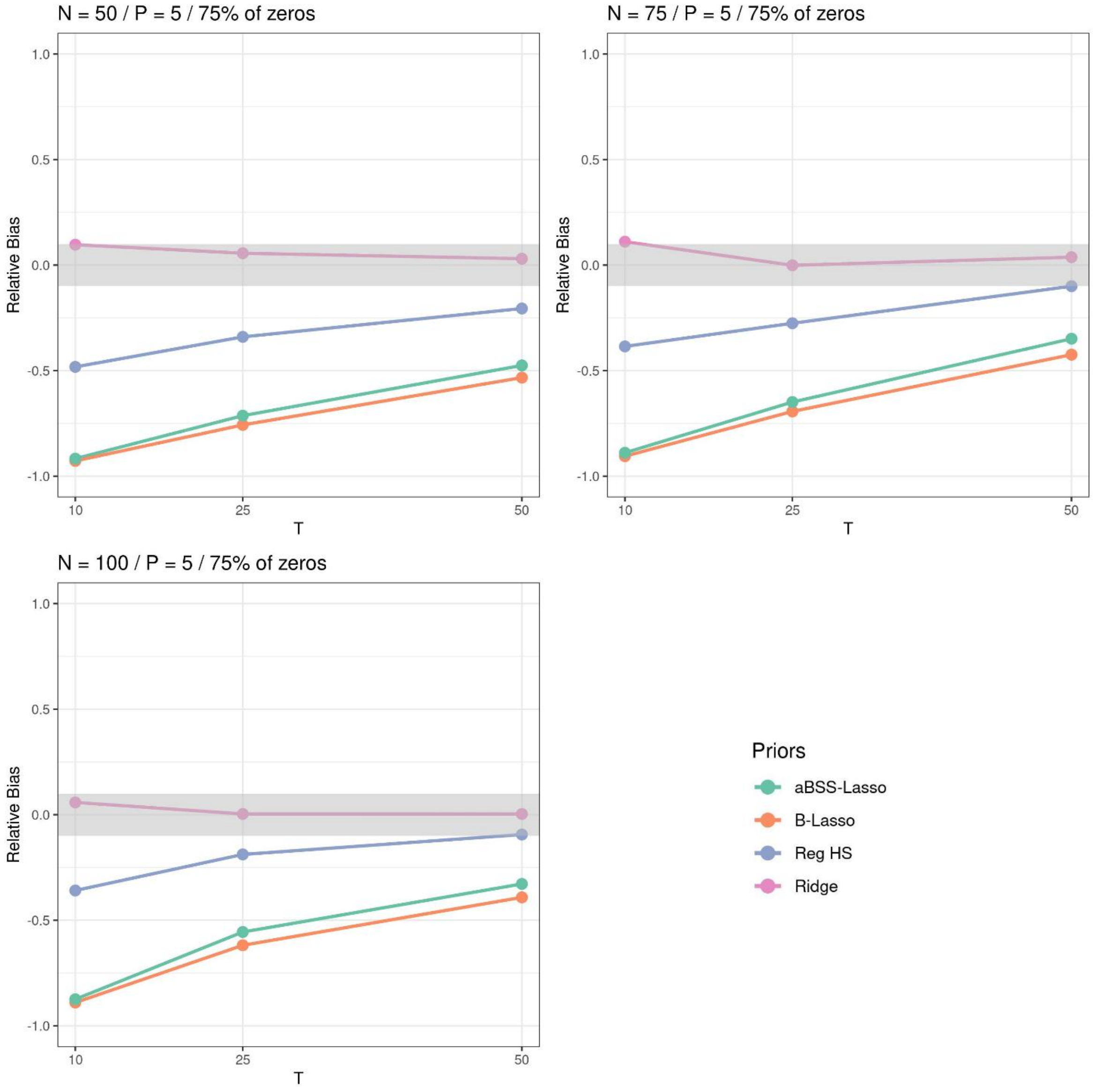

Figure 43: Relative Bias across N and T when $P = 5$ and 75% of the elements of the $\boldsymbol{\beta}^*$ are zeros (simulation study 1).

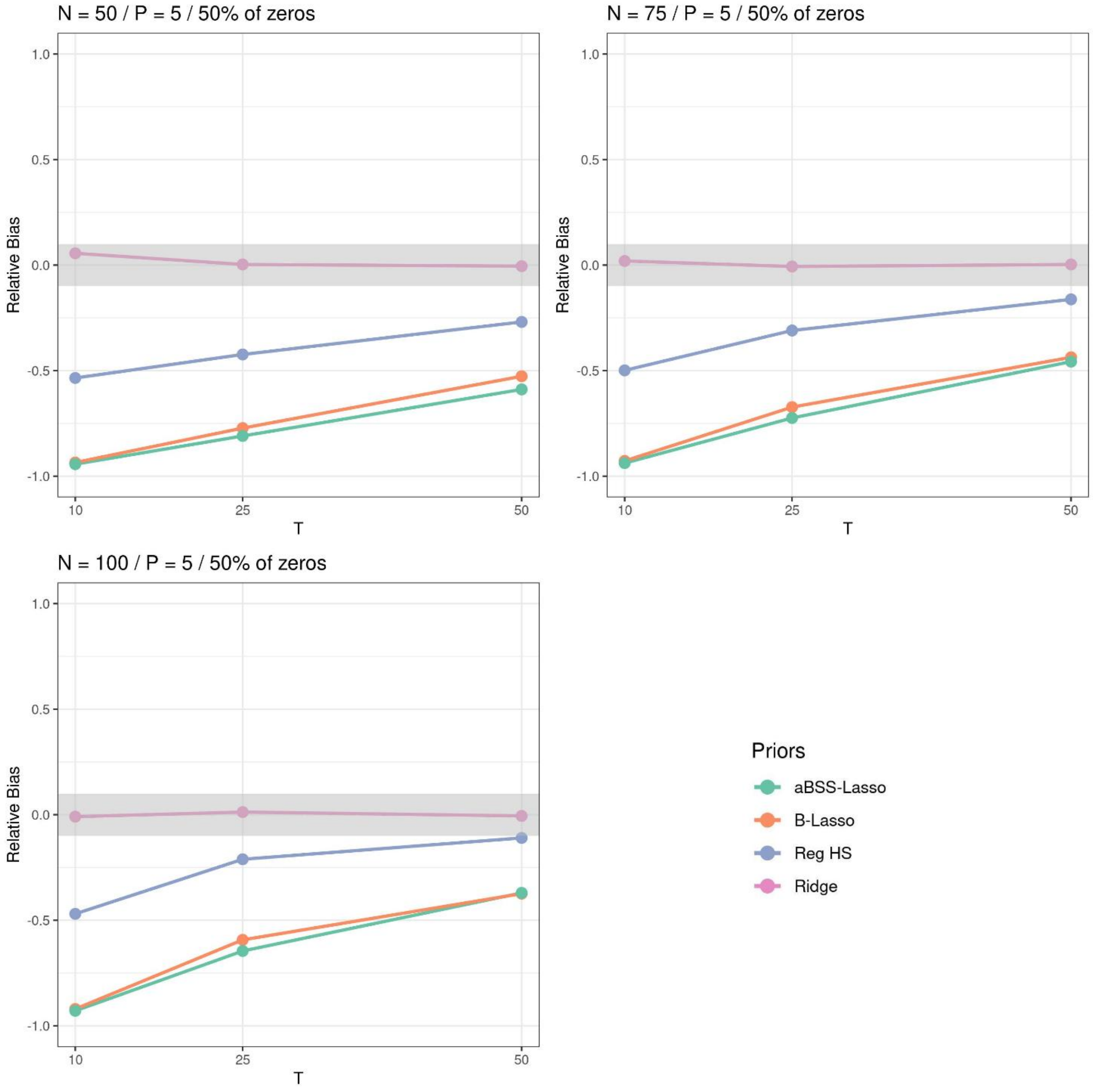

Figure 44: Relative Bias across N and T when $P = 5$ and 50% of the elements of the $\boldsymbol{\beta}^*$ are zeros (simulation study 1).

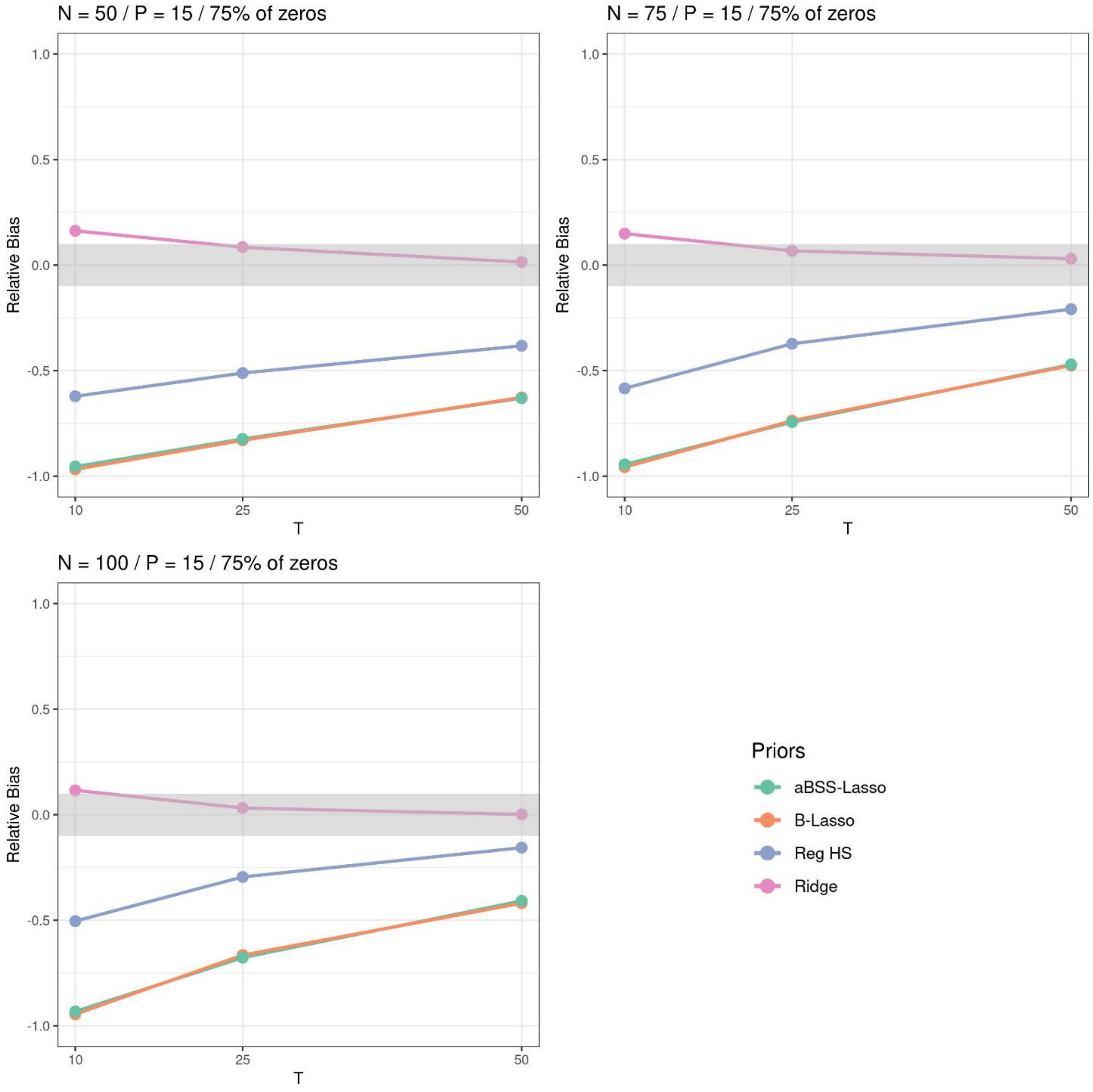

Figure 45: Relative Bias across N and T when $P = 15$ and 75% of the elements of the $\boldsymbol{\beta}^*$ are zeros (simulation study 1).

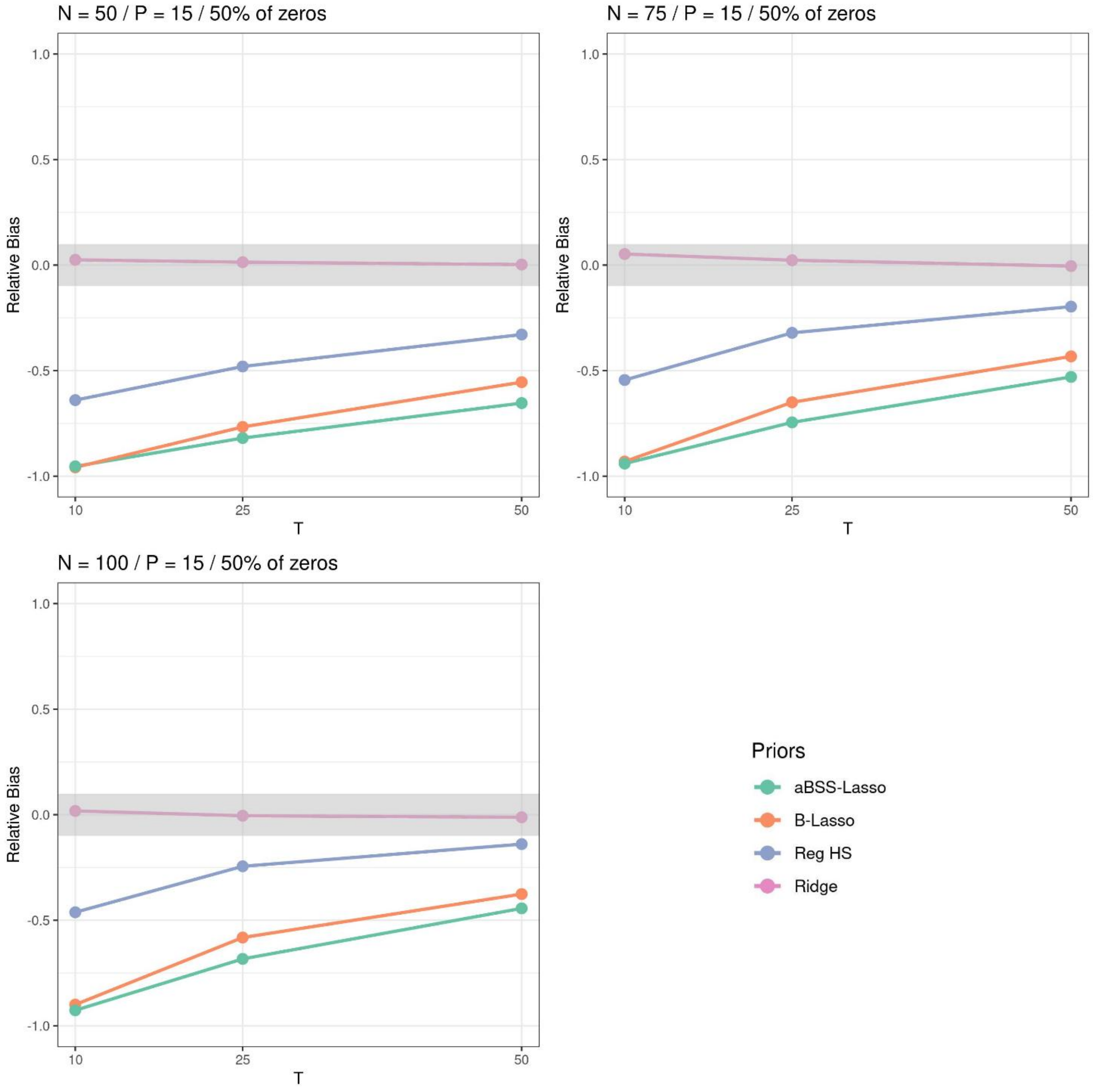

Figure 46: Relative Bias across N and T when $P = 15$ and 50% of the elements of the $\boldsymbol{\beta}^*$ are zeros (simulation study 1).

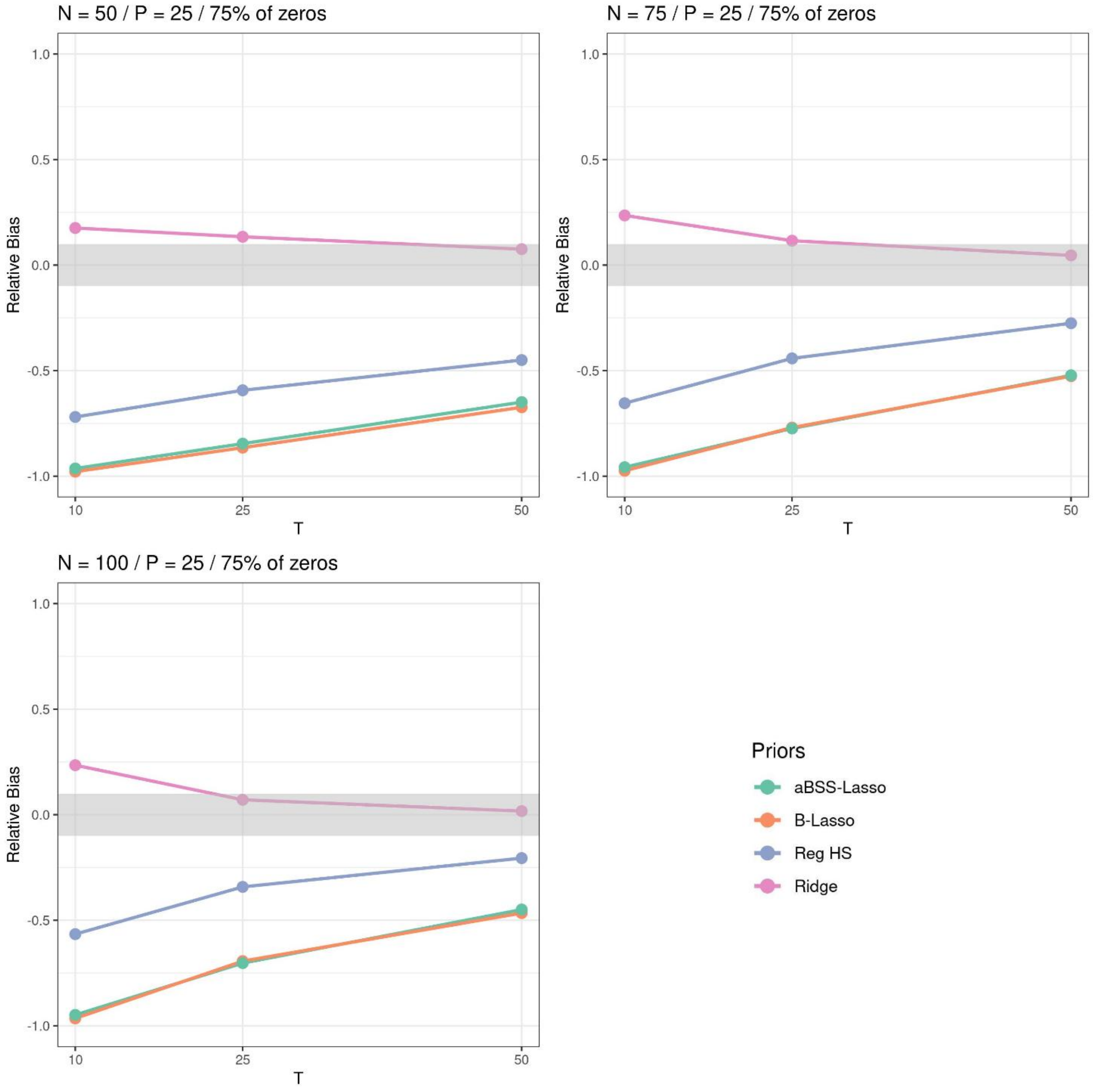

Figure 47: Relative Bias across N and T when $P = 25$ and 75% of the elements of the $\boldsymbol{\beta}^*$ are zeros (simulation study 1).

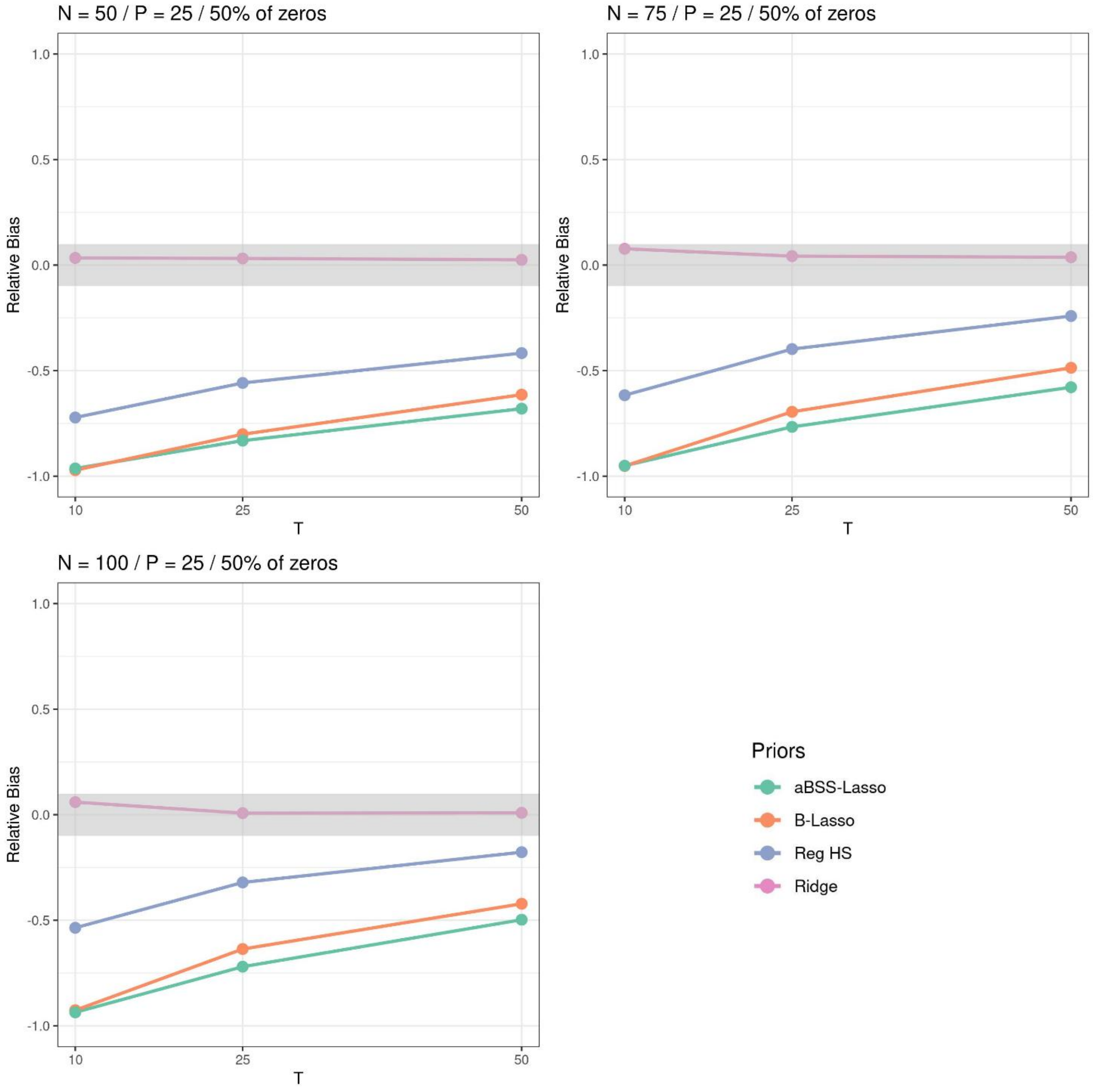

Figure 48: Relative Bias across N and T when $P = 25$ and 50% of the elements of the $\boldsymbol{\beta}^*$ are zeros (simulation study 1).

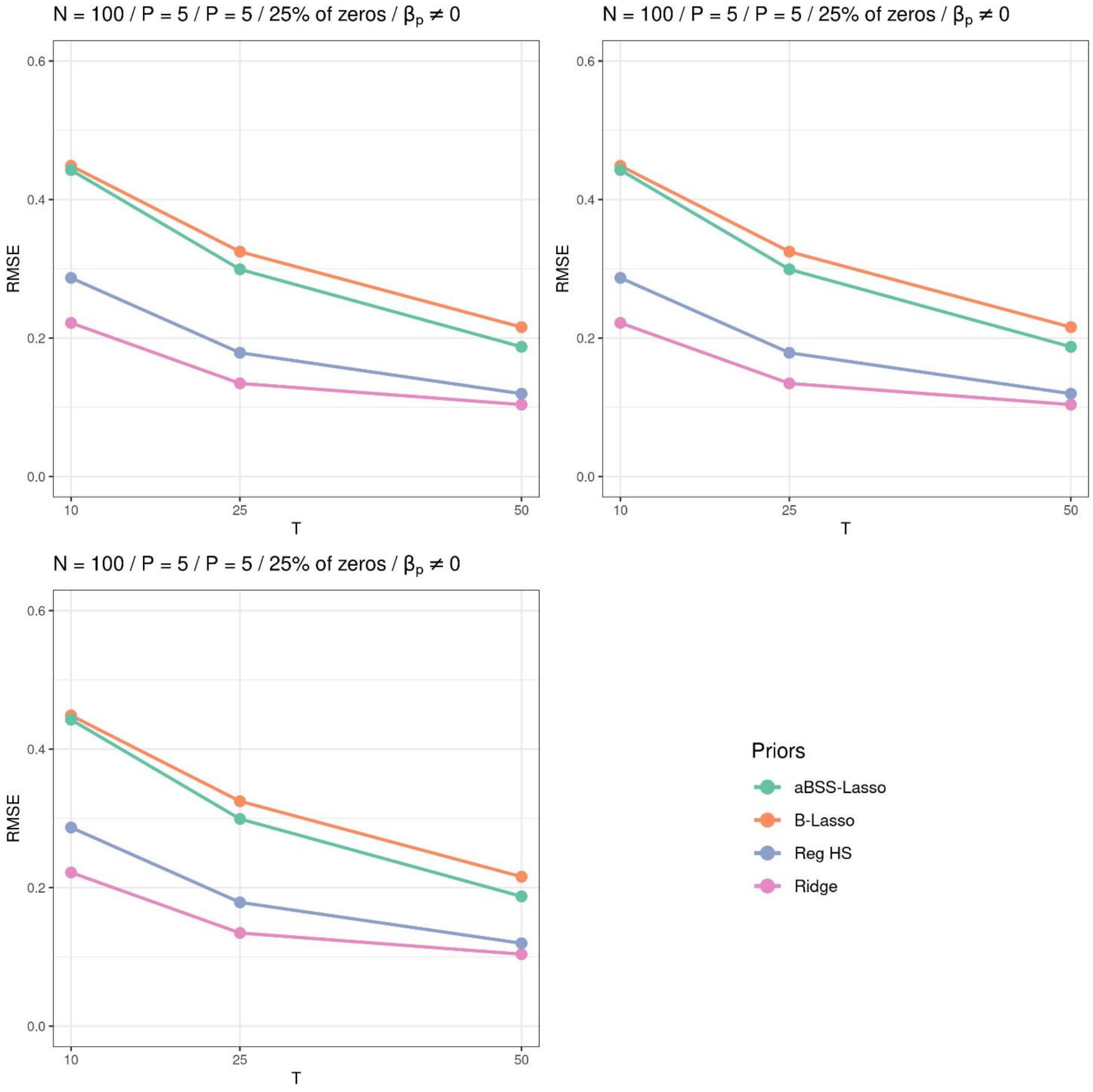

Figure 49: RMSE (for non-zero coeffcients) across N and T when $P = 5$ and 75% of the elements of the $\boldsymbol{\beta}^*$ are zeros (simulation study 1).

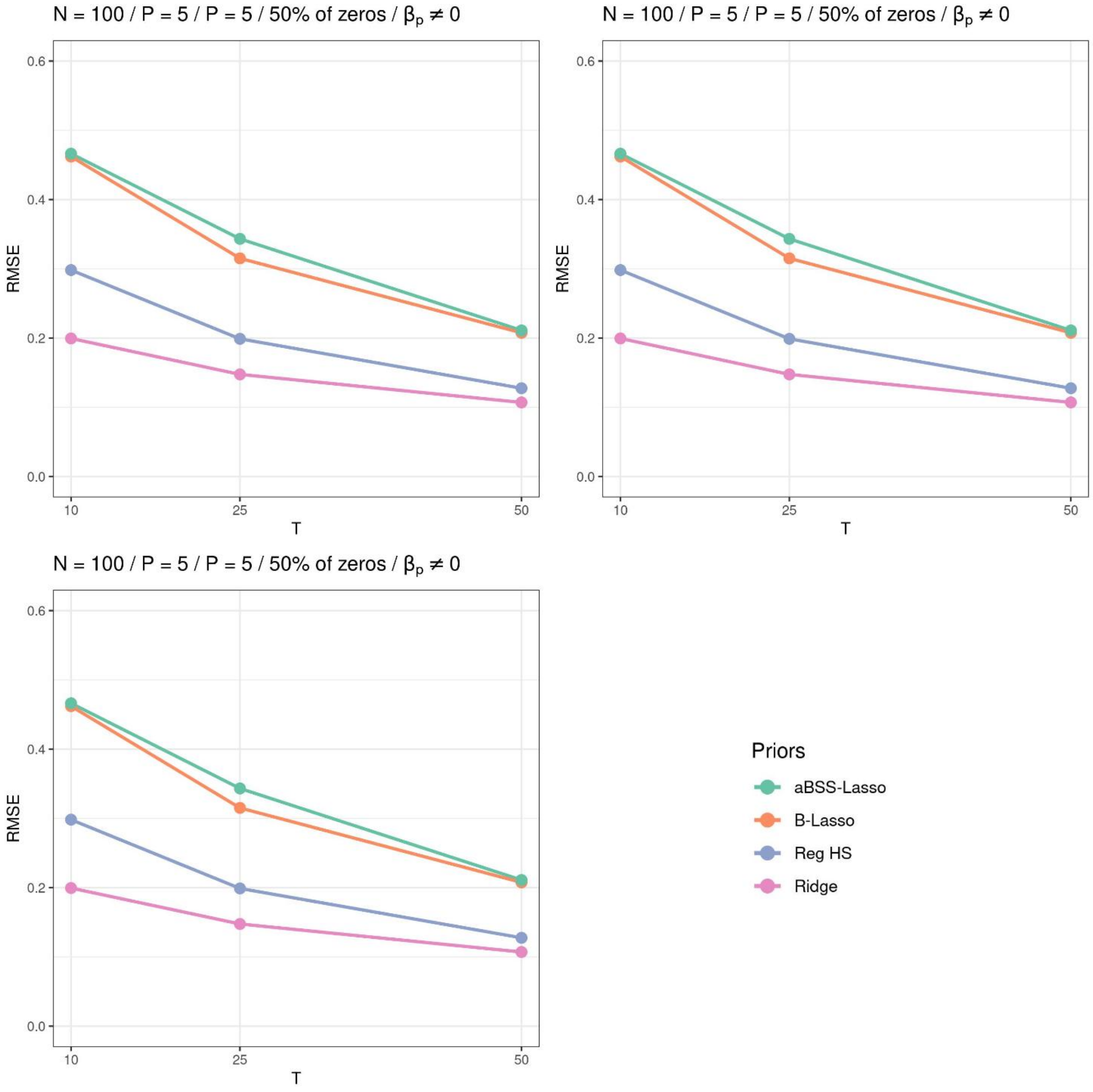

Figure 50: RMSE (for non-zero coeffcients) across N and T when $P = 5$ and 50% of the elements of the $\boldsymbol{\beta}^*$ are zeros (simulation study 1).

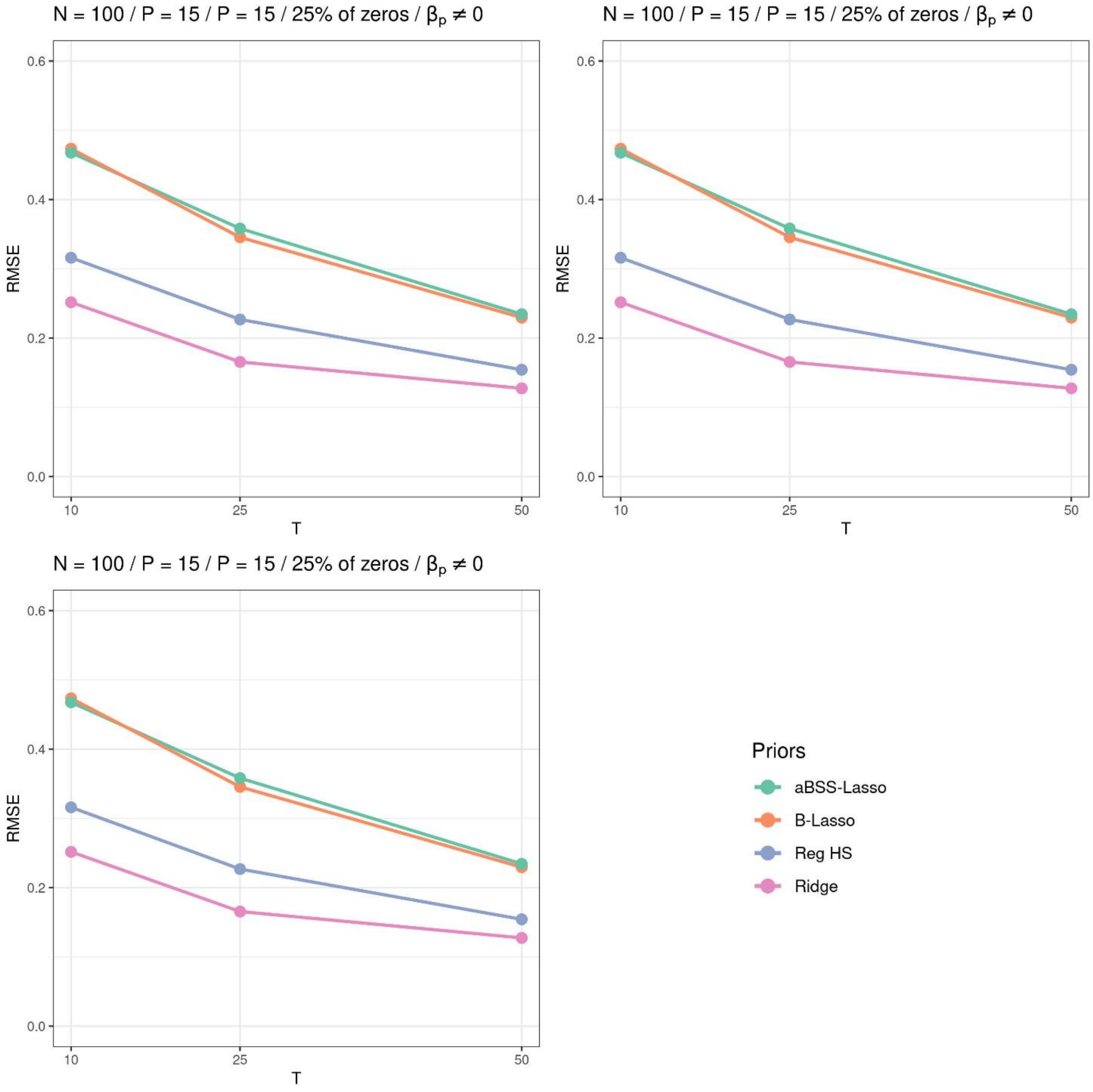

Figure 51: RMSE (for non-zero coeffcients) across N and T when $P = 15$ and 75% of the elements of the $\boldsymbol{\beta}^*$ are zeros (simulation study 1).

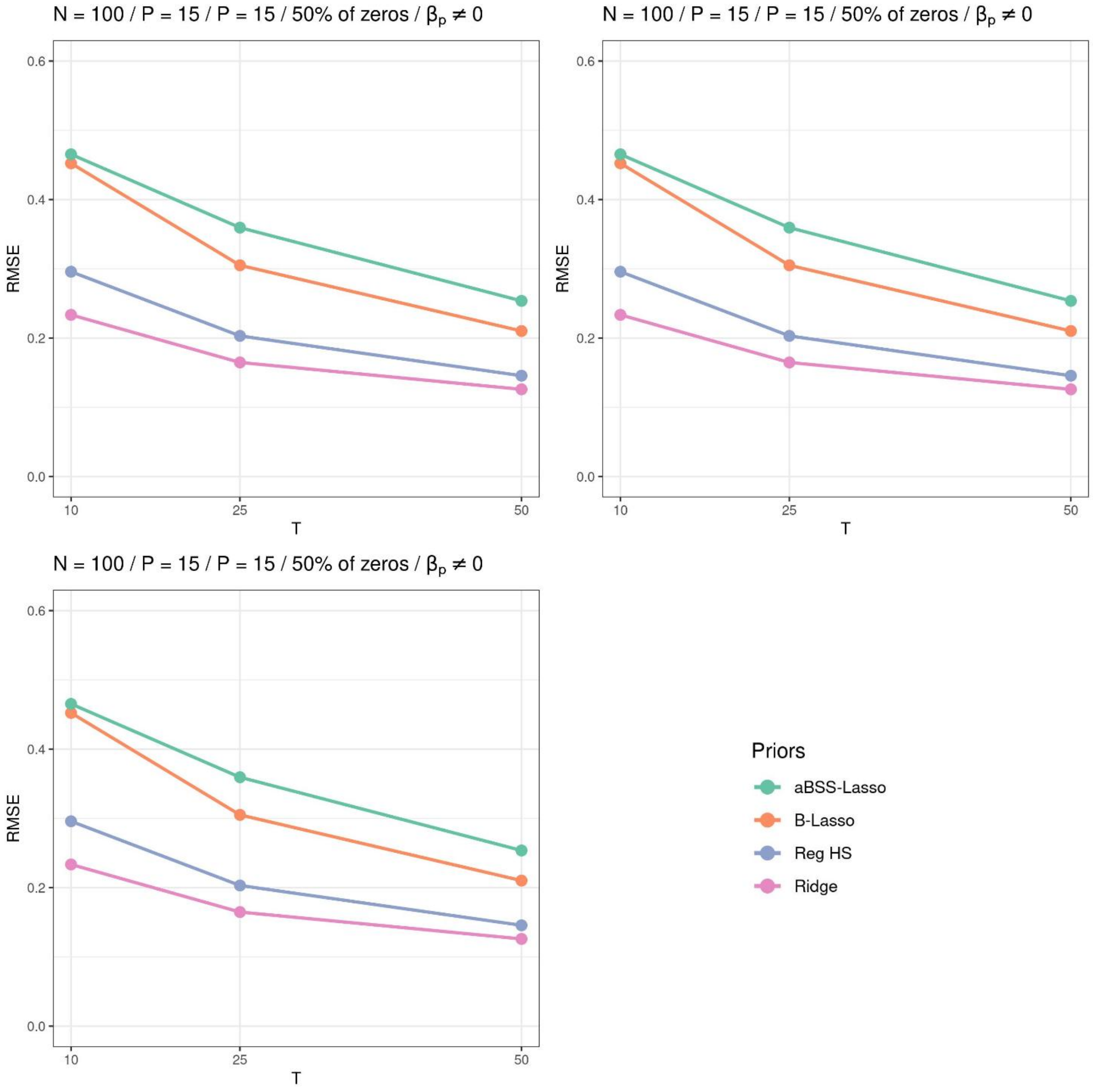

Figure 52: RMSE (for non-zero coeffcients) across N and T when $P = 15$ and 50% of the elements of the $\boldsymbol{\beta}^*$ are zeros (simulation study 1).

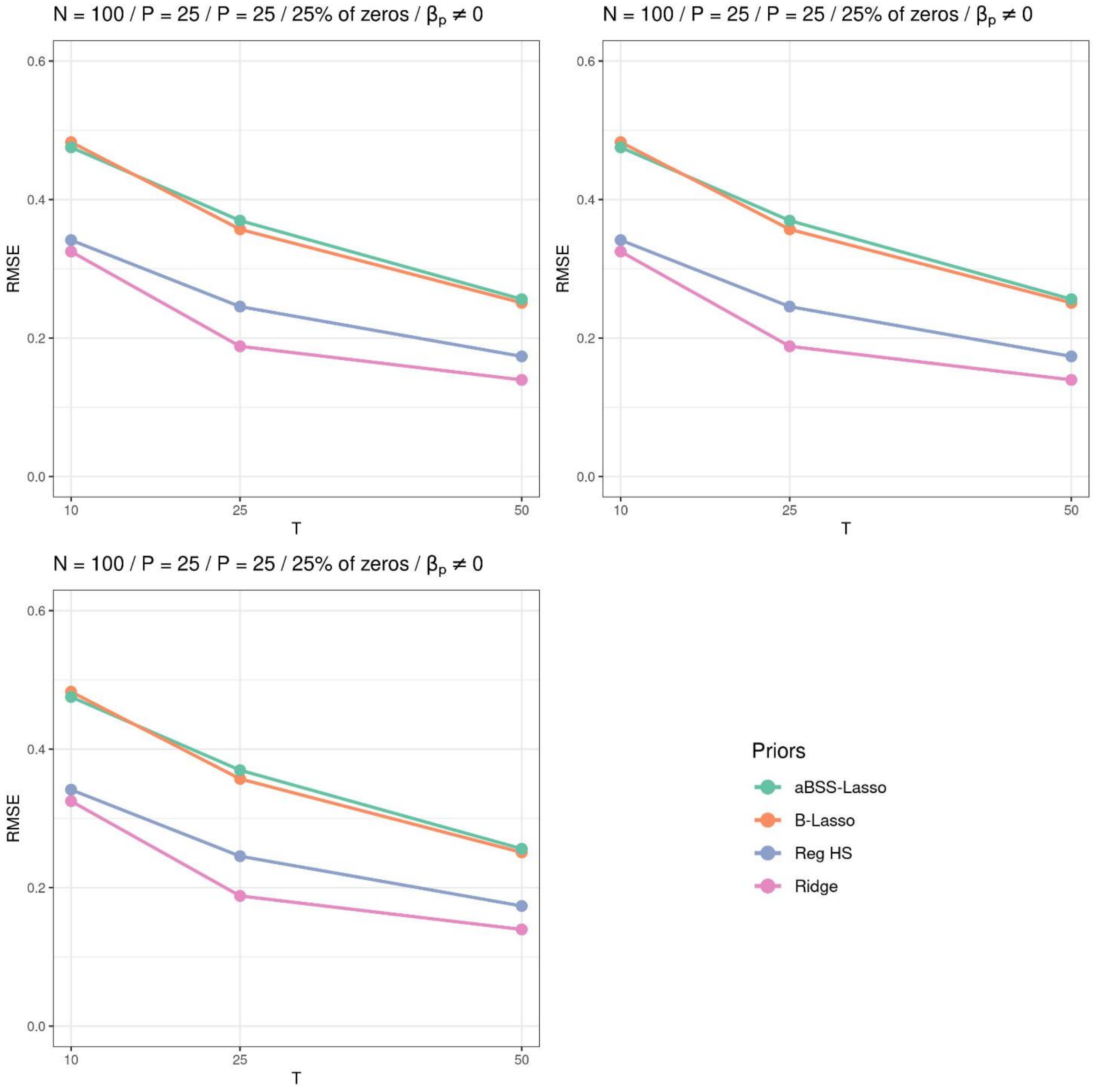

Figure 53: RMSE (for non-zero coeffcients) across N and T when $P = 25$ and 75% of the elements of the $\boldsymbol{\beta}^*$ are zeros (simulation study 1).

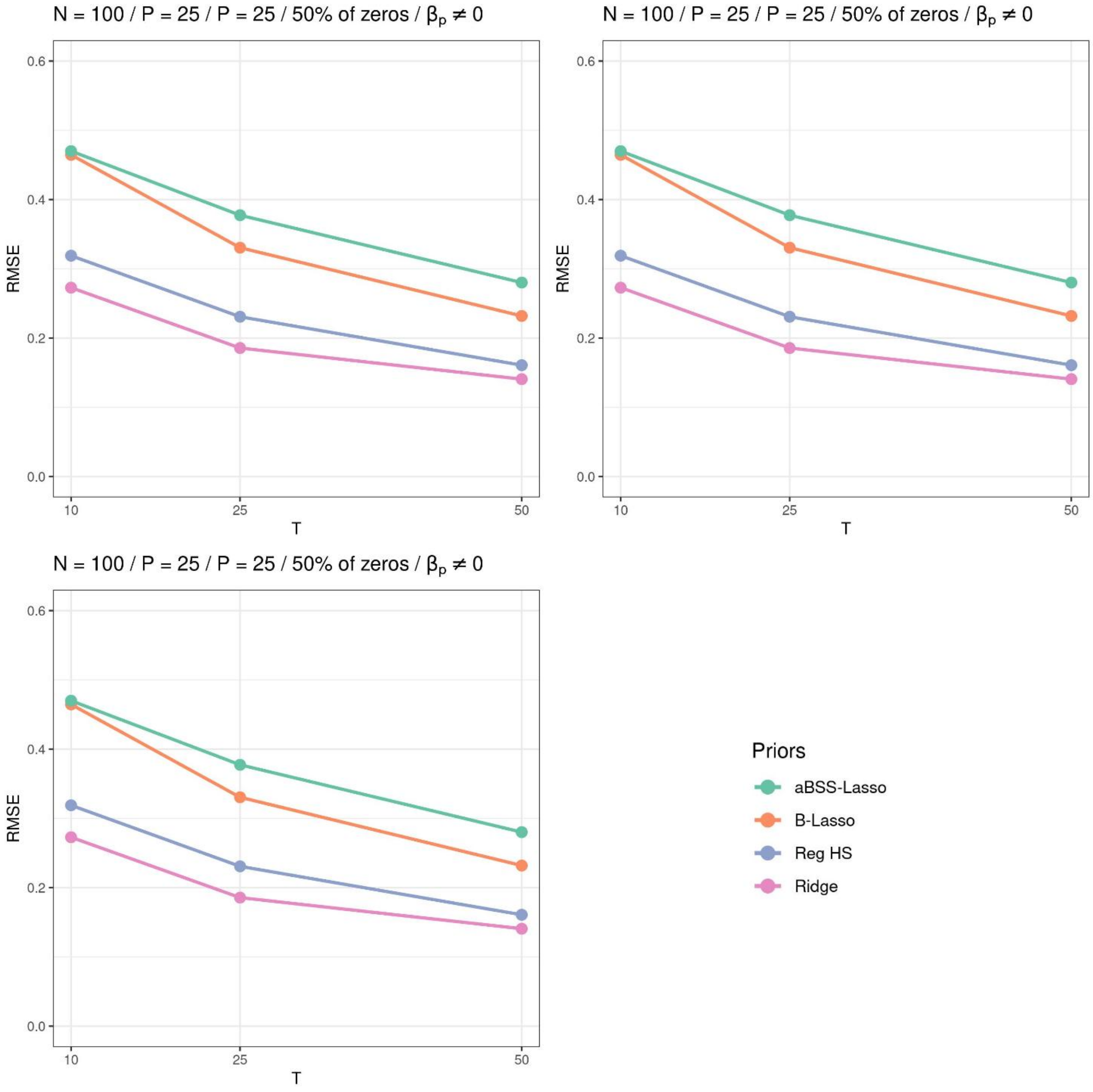

Figure 54: RMSE (for non-zero coeffcients) across N and T when $P = 25$ and 50% of the elements of the $\boldsymbol{\beta}^*$ are zeros (simulation study 1).

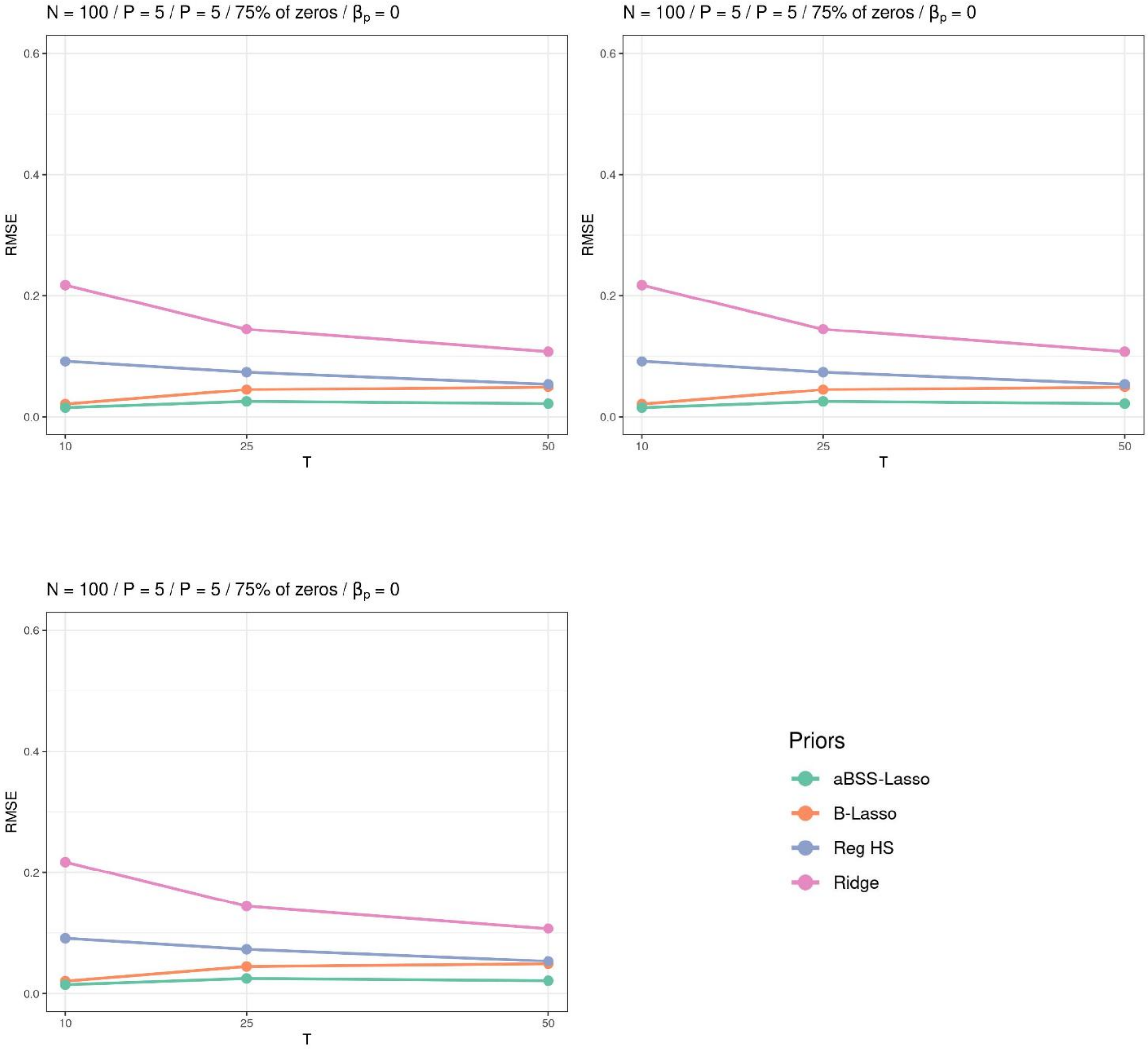

Figure 55: RMSE (zero coefficients) across N and T when $P = 5$ and 75% of the elements of the $\boldsymbol{\beta}^*$ are zeros (simulation study 1).

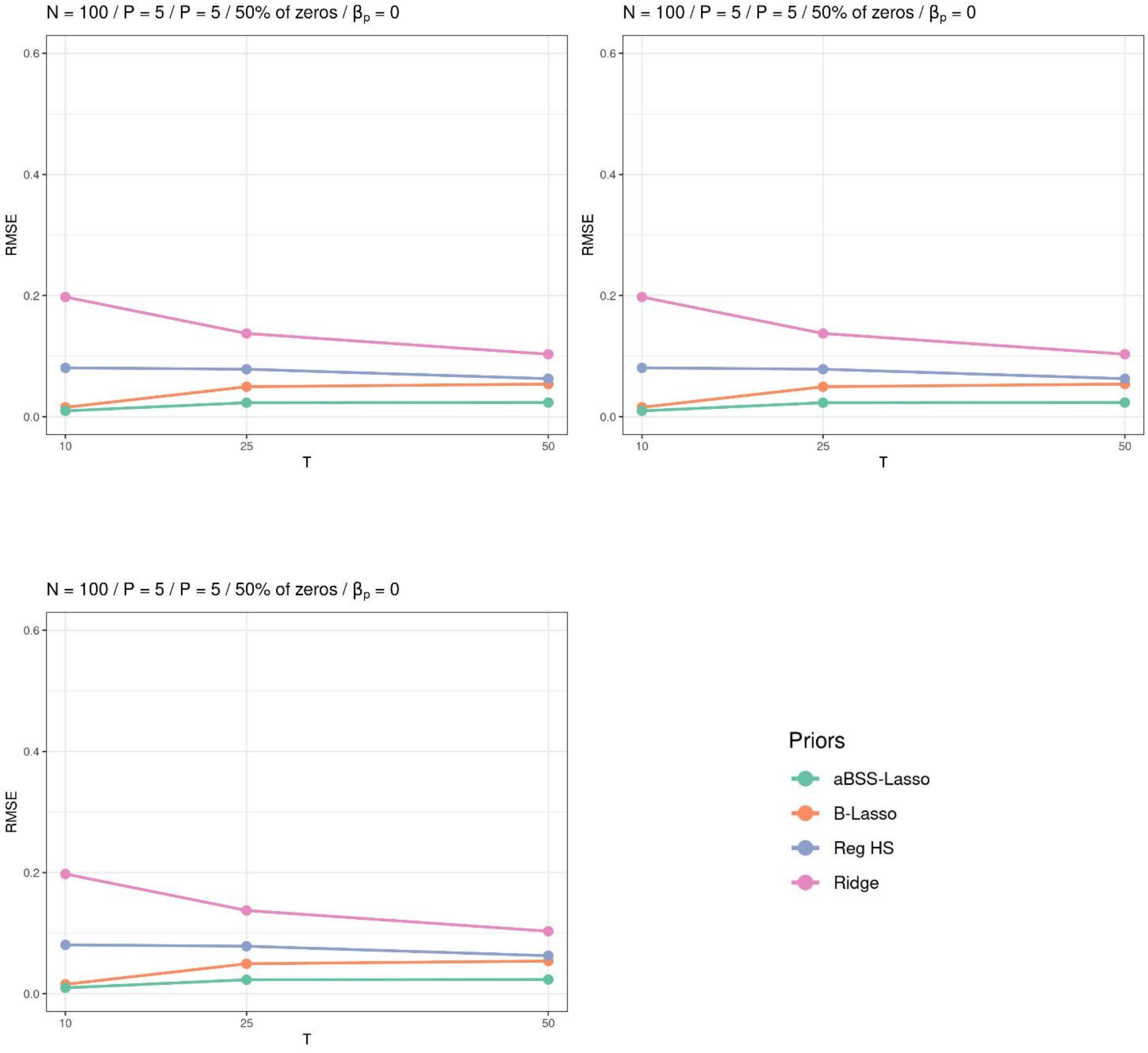

Figure 56: RMSE (zero coefficients) across N and T when $P = 5$ and 50% of the elements of the $\boldsymbol{\beta}^*$ are zeros (simulation study 1).

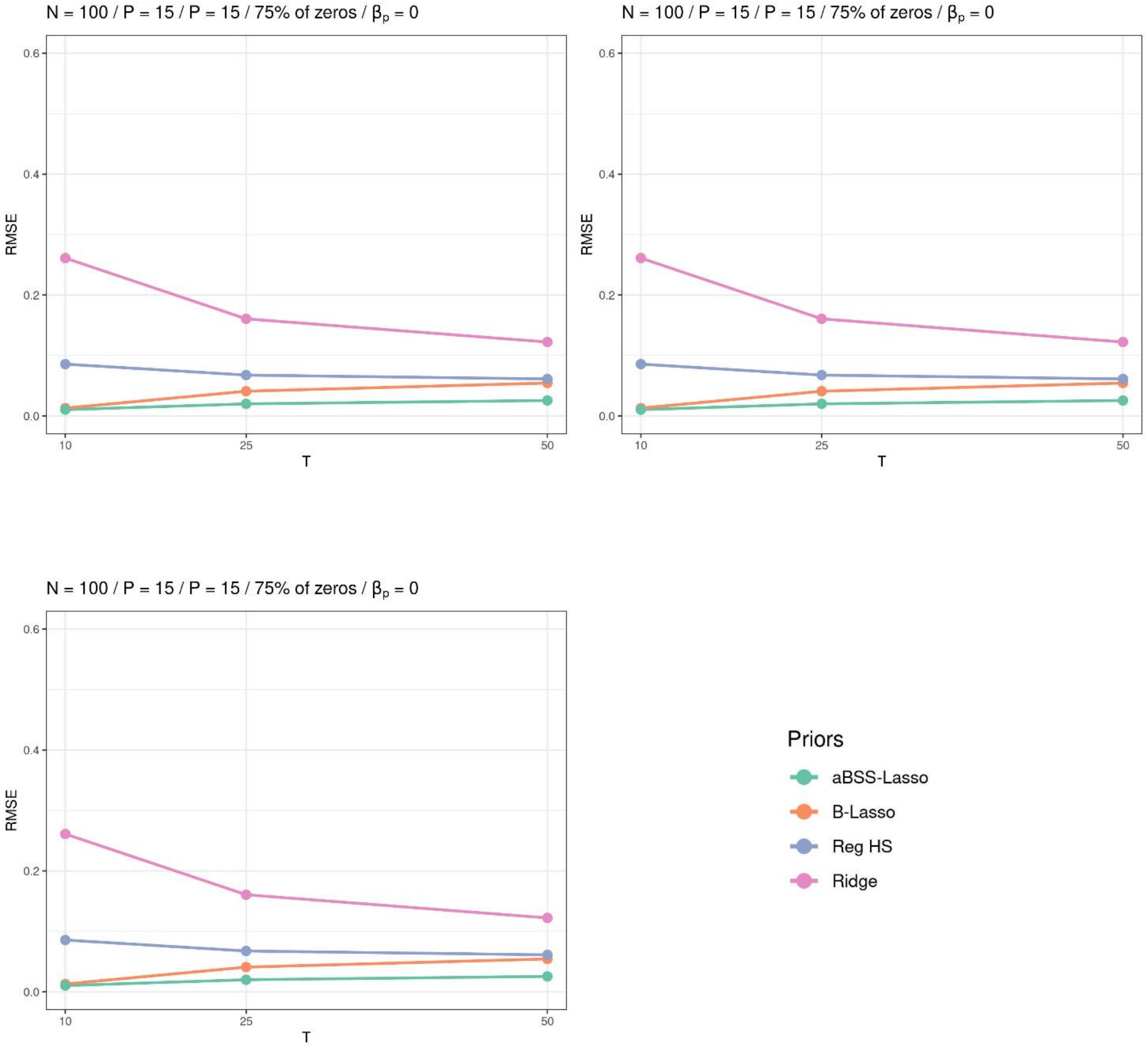

Figure 57: RMSE (zero coefficients) across N and T when $P = 15$ and 75% of the elements of the $\boldsymbol{\beta}^*$ are zeros (simulation study 1).

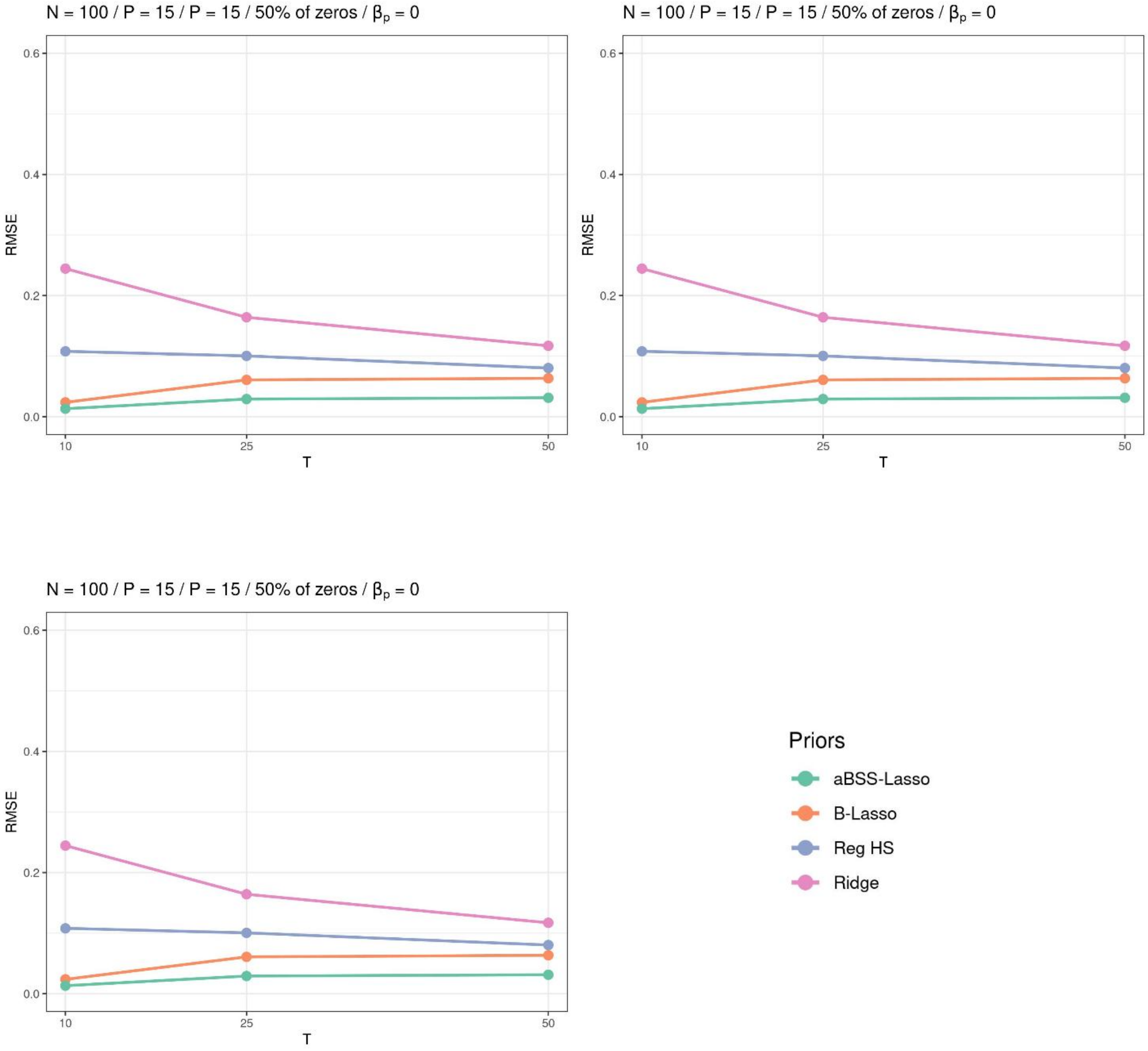

Figure 58: RMSE (zero coefficients) across N and T when $P = 15$ and 50% of the elements of the $\boldsymbol{\beta}^*$ are zeros (simulation study 1).

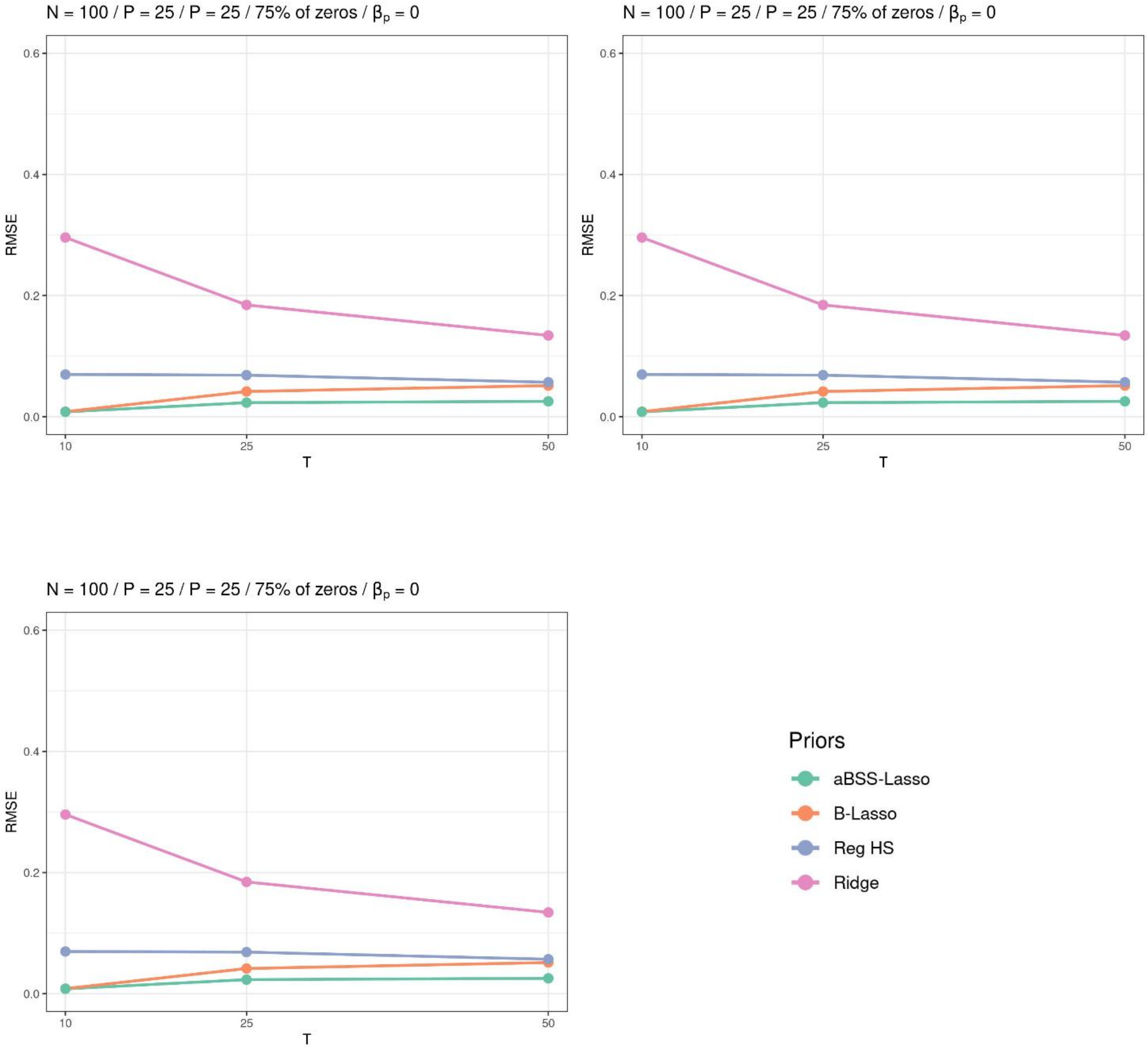

Figure 59: RMSE (zero coefficients) across N and T when $P = 25$ and 75% of the elements of the $\boldsymbol{\beta}^*$ are zeros (simulation study 1).

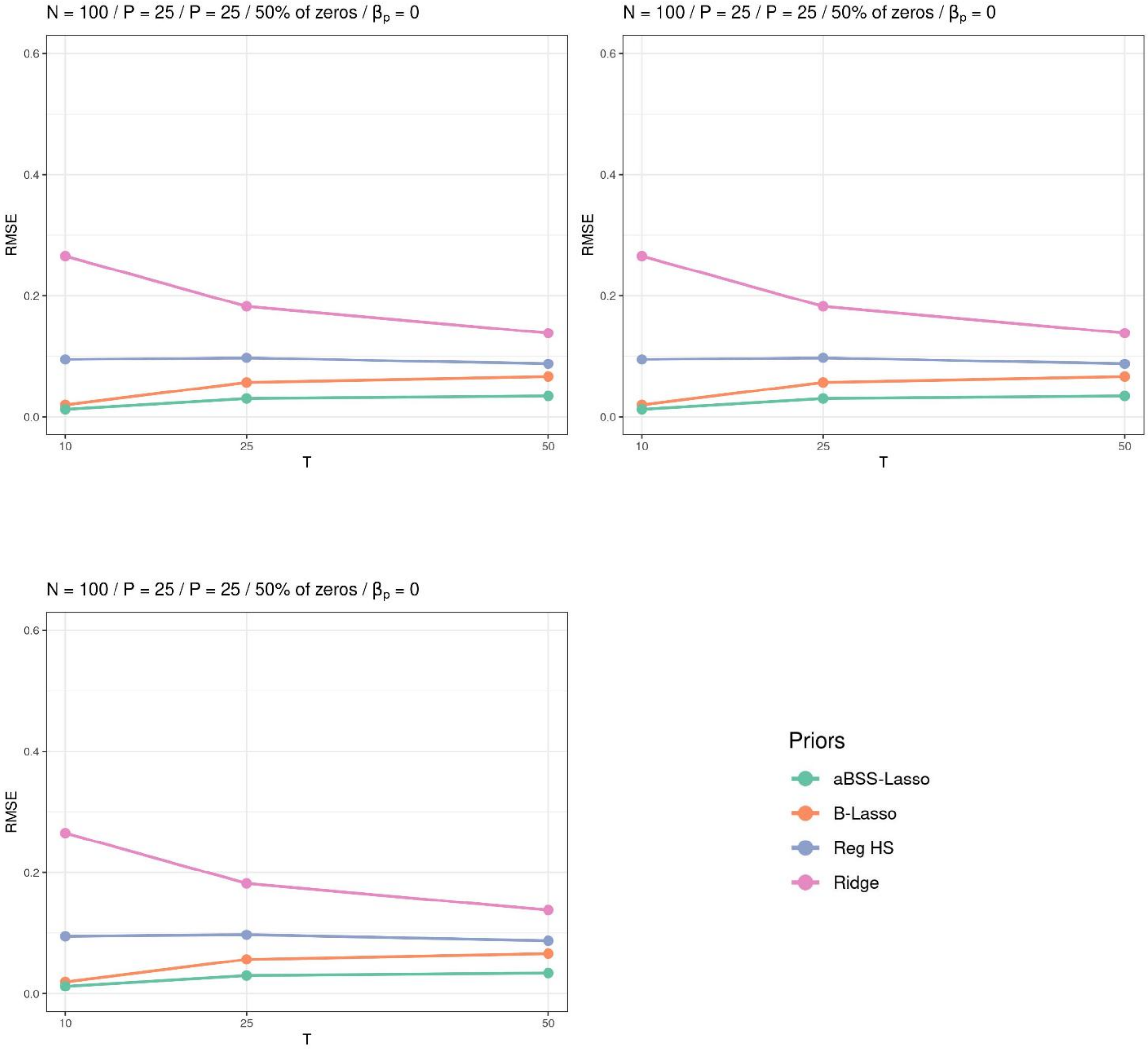

Figure 60: RMSE (zero coefficients) across N and T when $P = 25$ and 50% of the elements of the $\boldsymbol{\beta}^*$ are zeros (simulation study 1).

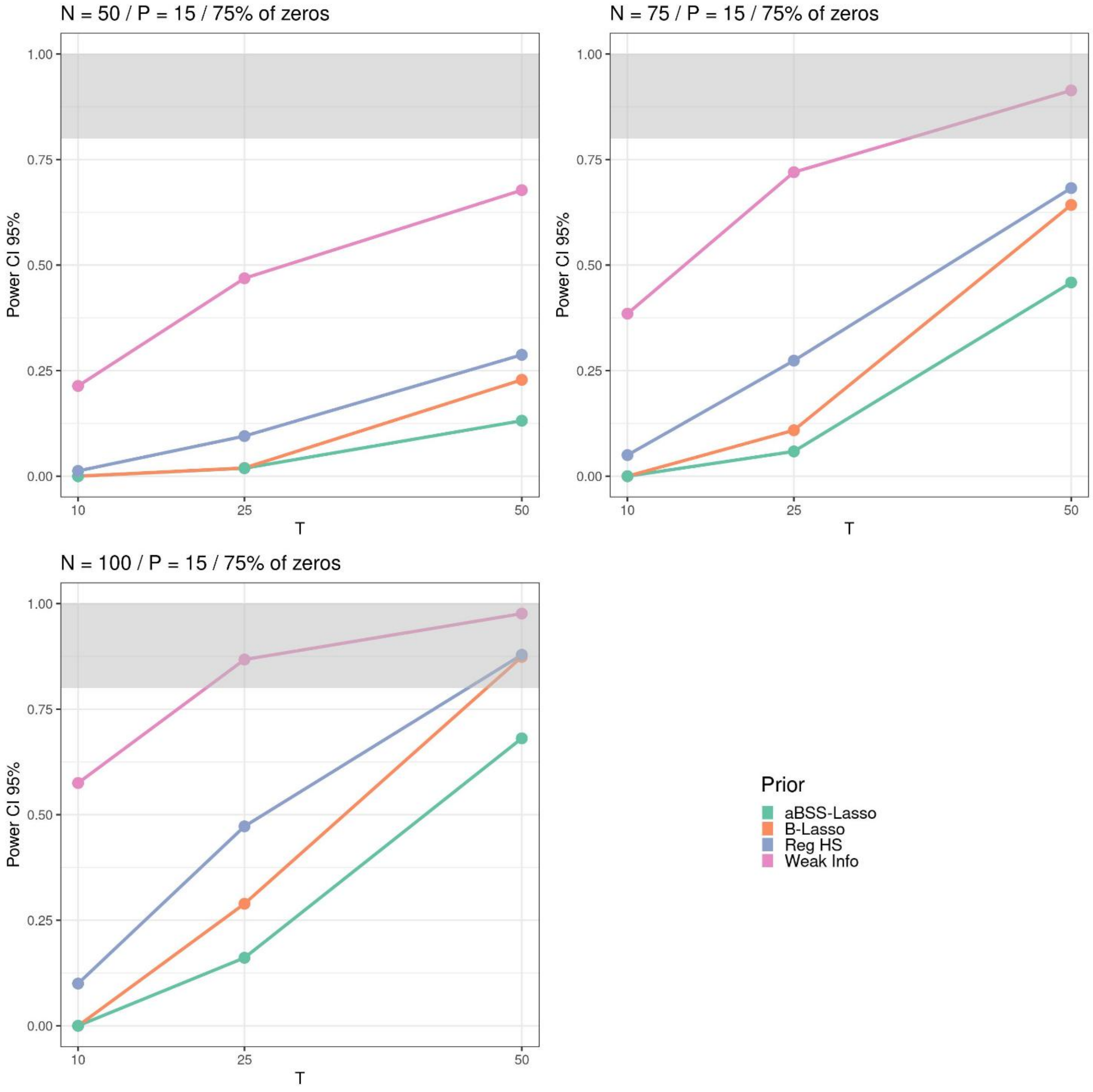

Figure 61: Power rate based on 95% CI across T and T when $P = 25$ and 75% of the elements of the $\boldsymbol{\beta}^*$ are zeros (simulation study 1).

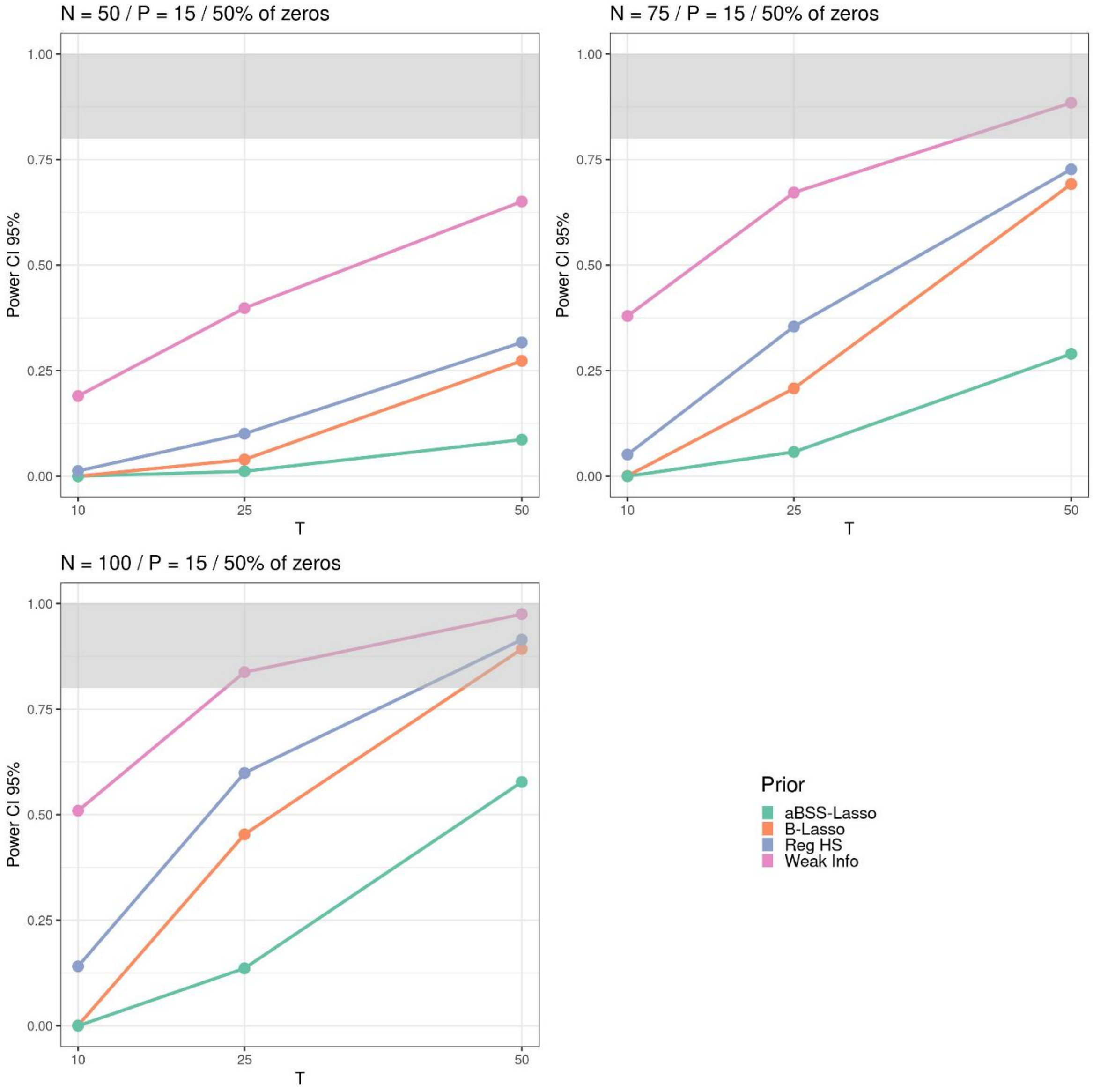

Figure 62: Power rate based on 95% CI across T and T when $P = 25$ and 50% of the elements of the $\boldsymbol{\beta}^*$ are zeros (simulation study 1).

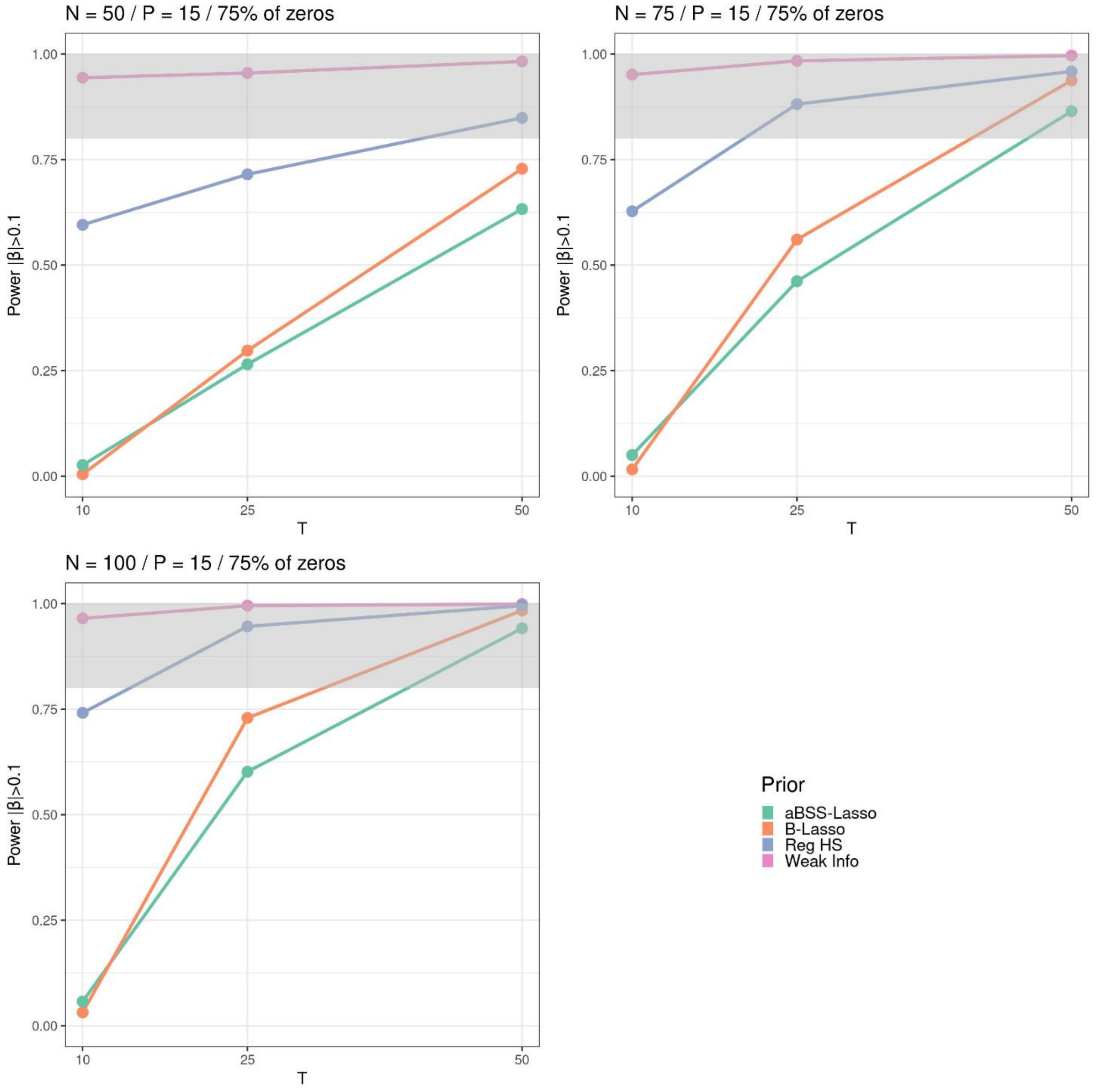

Figure 63: Power rate based on $|\beta_p| > 0.1$'s threshold across N and T when $P = 25$ and 75% of the elements of the $\boldsymbol{\beta}^*$ are zeros (simulation study 1).

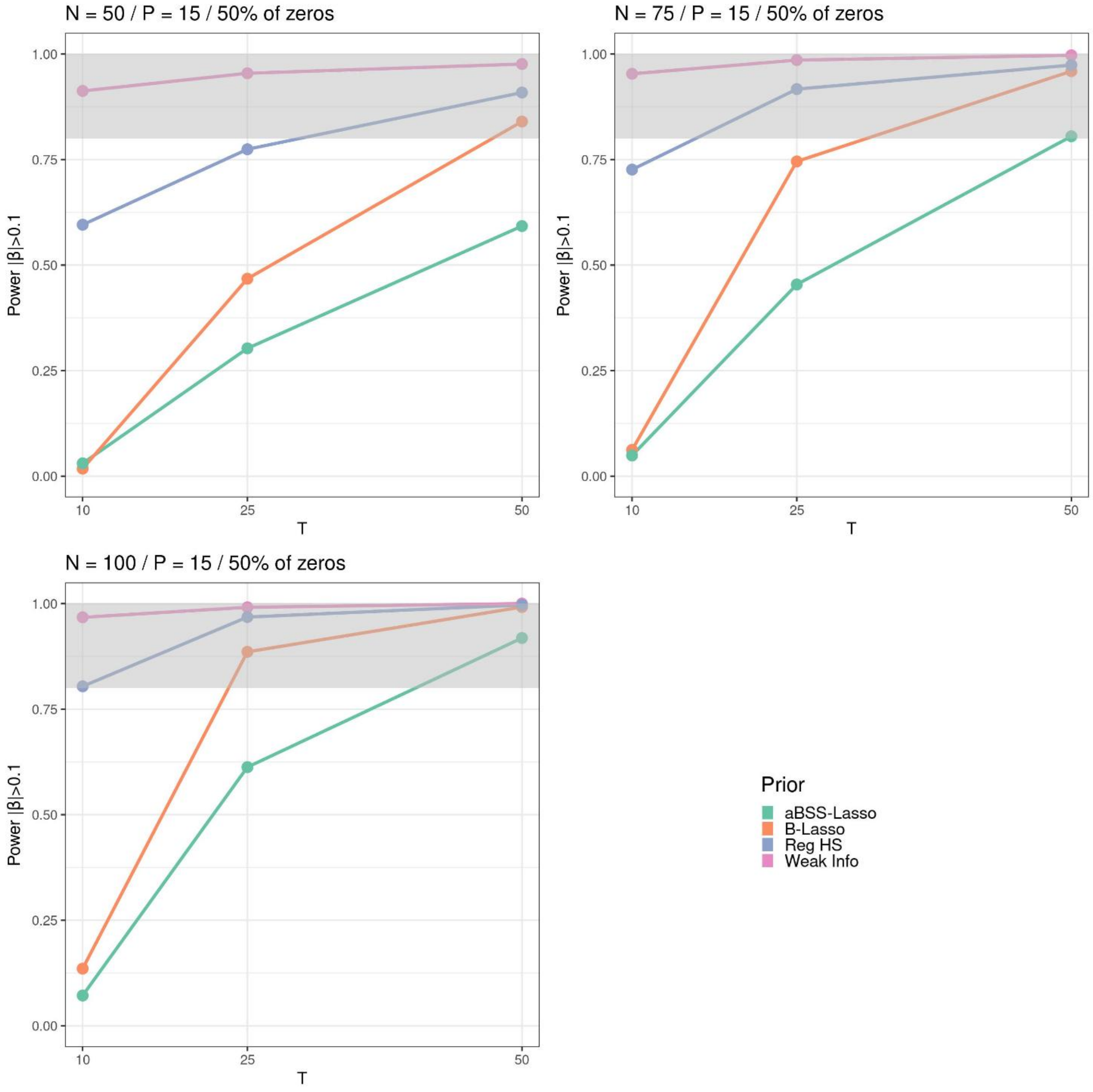

Figure 64: Power rate based on $|\beta_p| > 0.1$'s threshold across N and T when $P = 25$ and 50% of the elements of the $\boldsymbol{\beta}^*$ are zeros (simulation study 1).

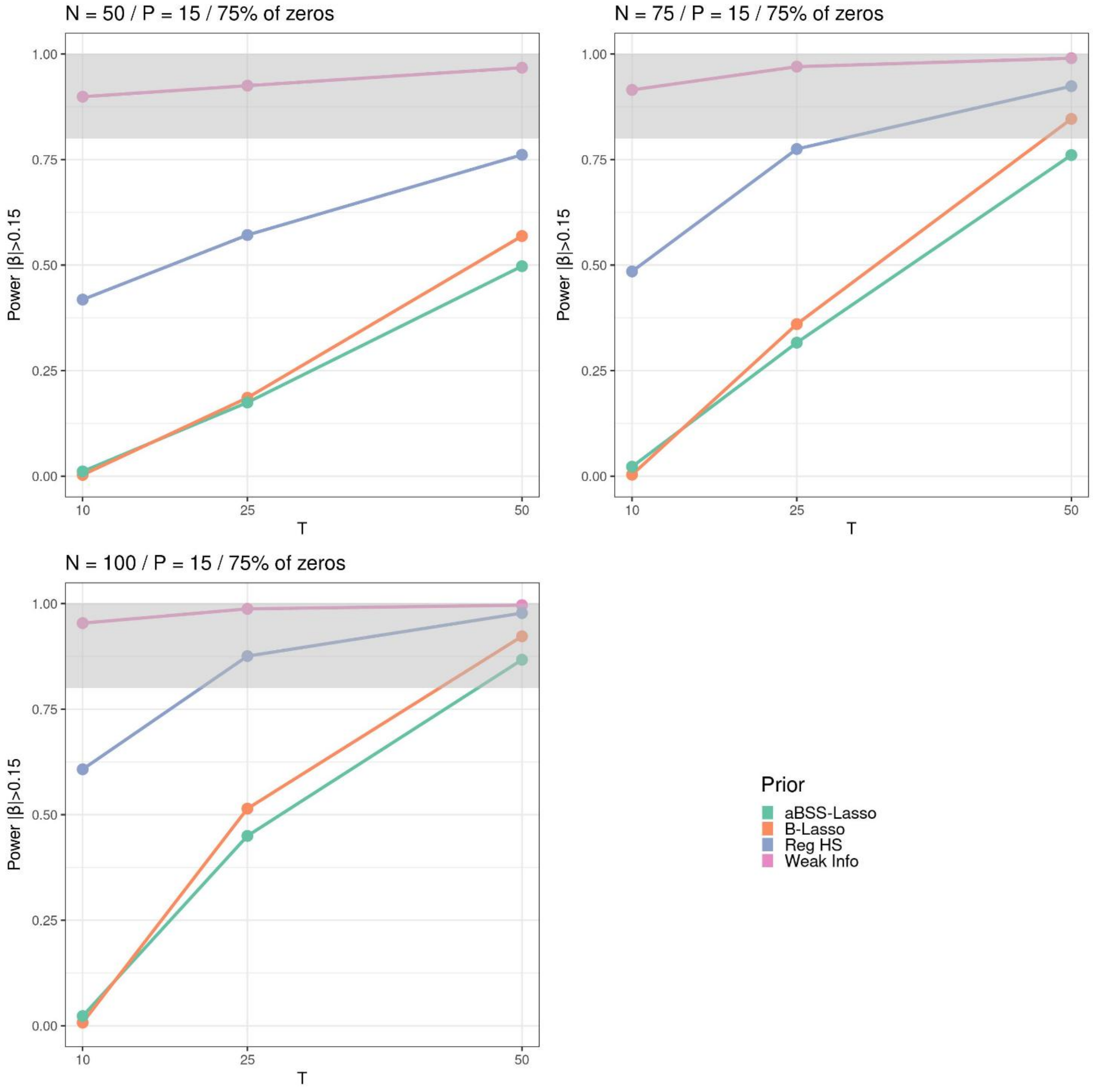

Figure 65: Power rate based on $|\beta_p| > 0.15$'s threshold across N and T when $P = 25$ and 75% of the elements of the $\boldsymbol{\beta}^*$ are zeros (simulation study 1).

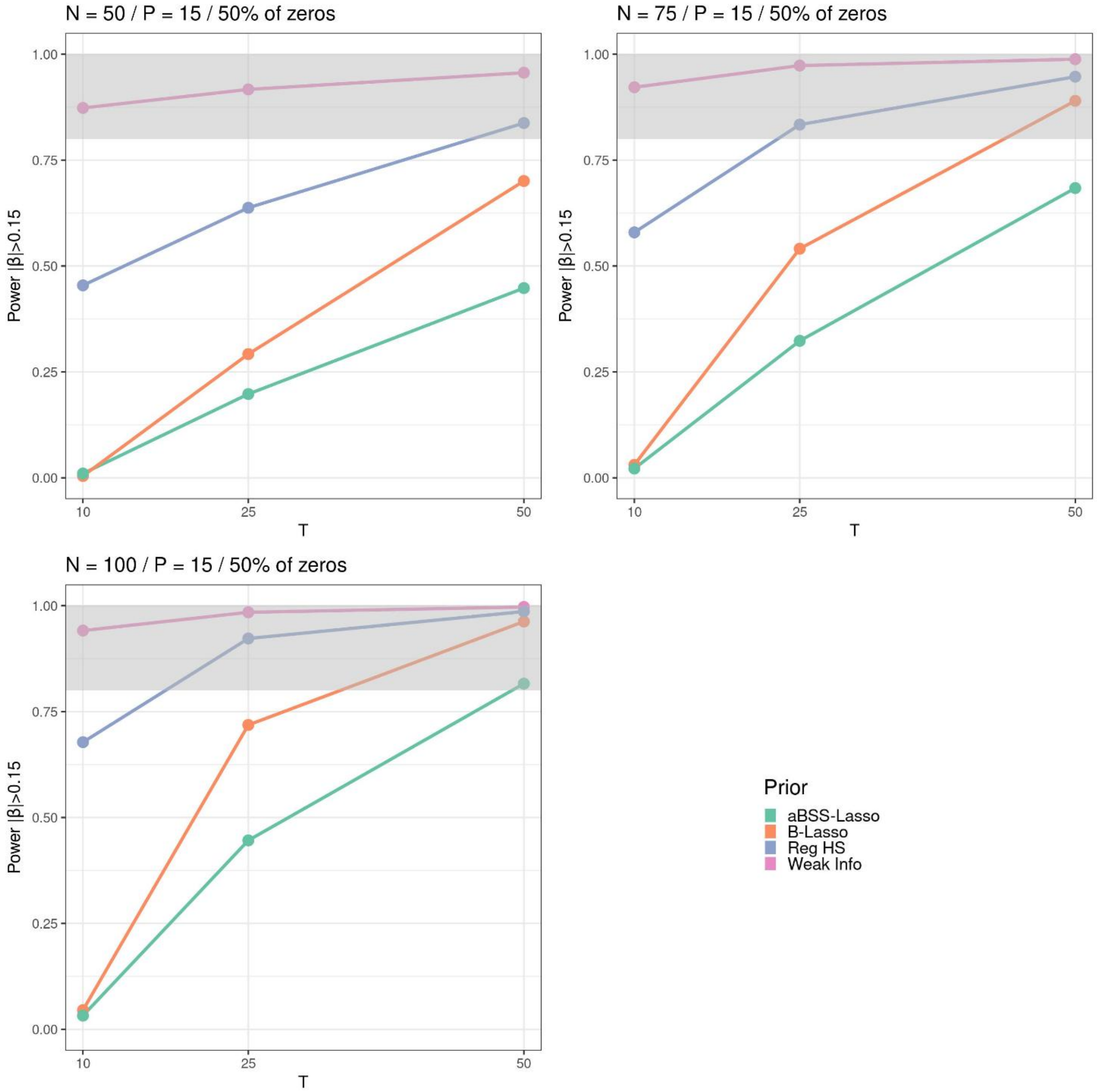

Figure 66: Power rate based on $|\beta_p| > 0.15$'s threshold across N and T when $P = 25$ and 50% of the elements of the $\boldsymbol{\beta}^*$ are zeros (simulation study 1).

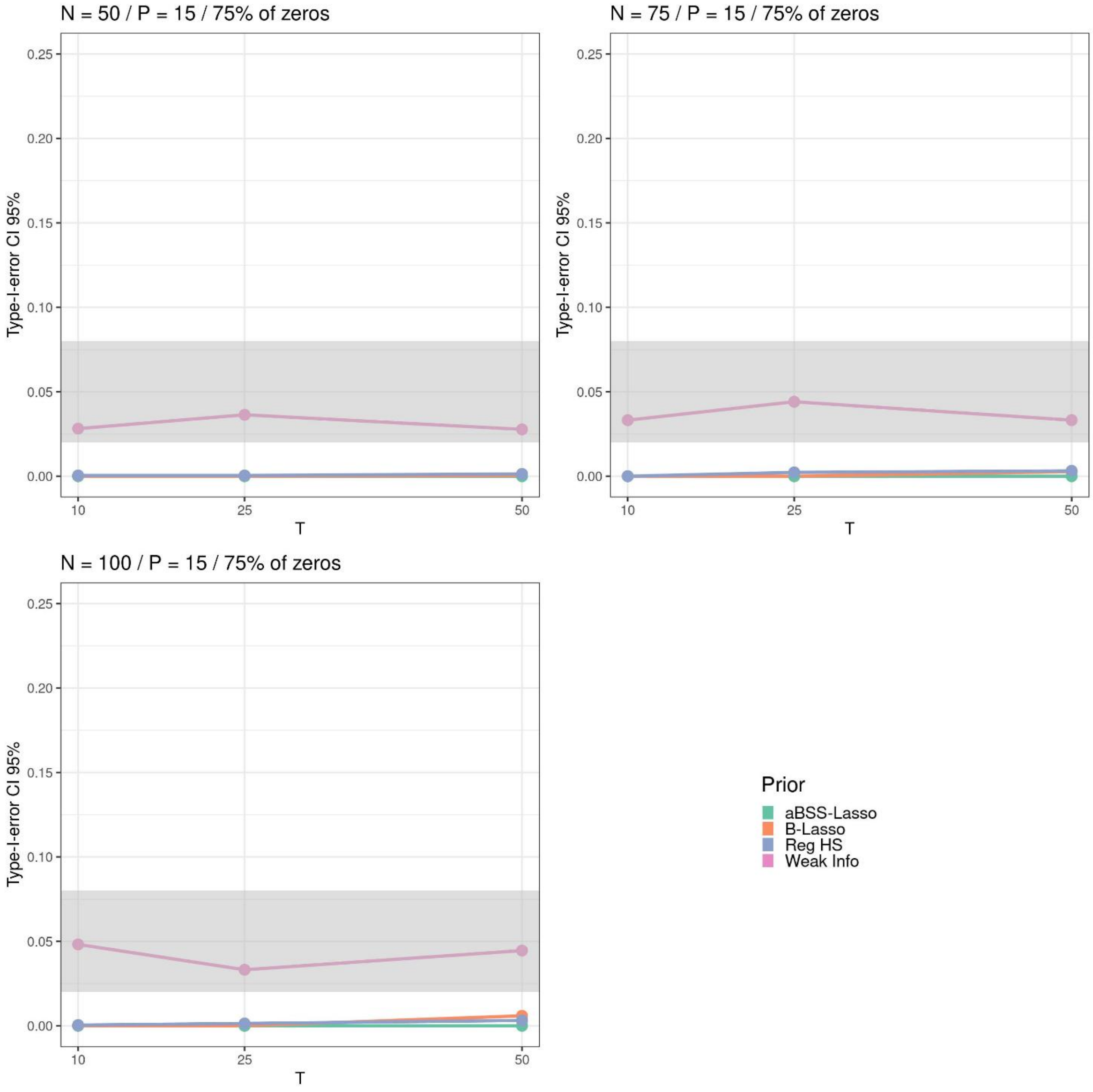

Figure 67: Type I error rate based on 95% CI across T and T when $P = 25$ and 75% of the elements of the $\boldsymbol{\beta}^*$ are zeros (simulation study 1).

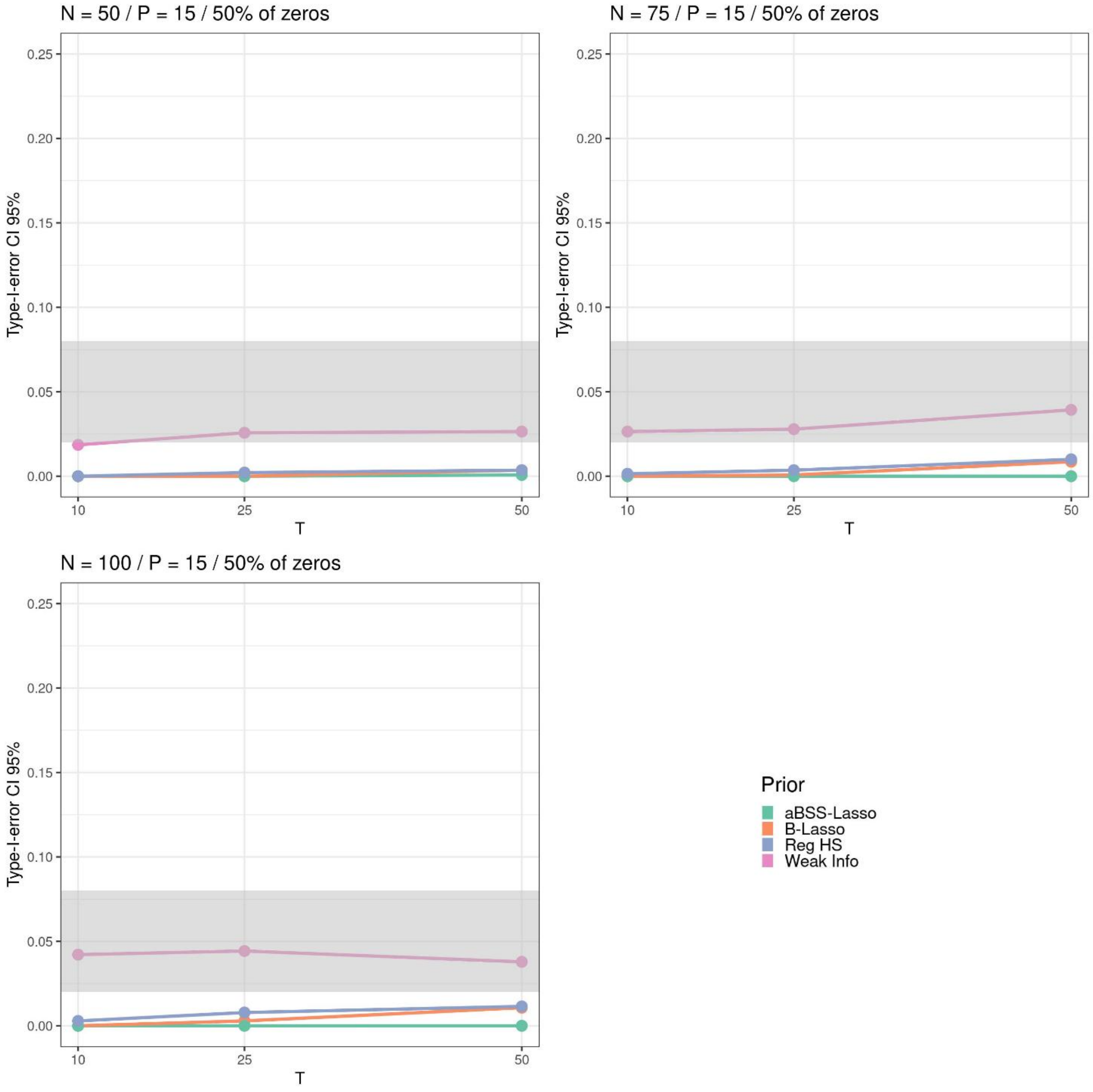

Figure 68: Type I error rate based on 95% CI across T and T when $P = 25$ and 50% of the elements of the $\boldsymbol{\beta}^*$ are zeros (simulation study 1).

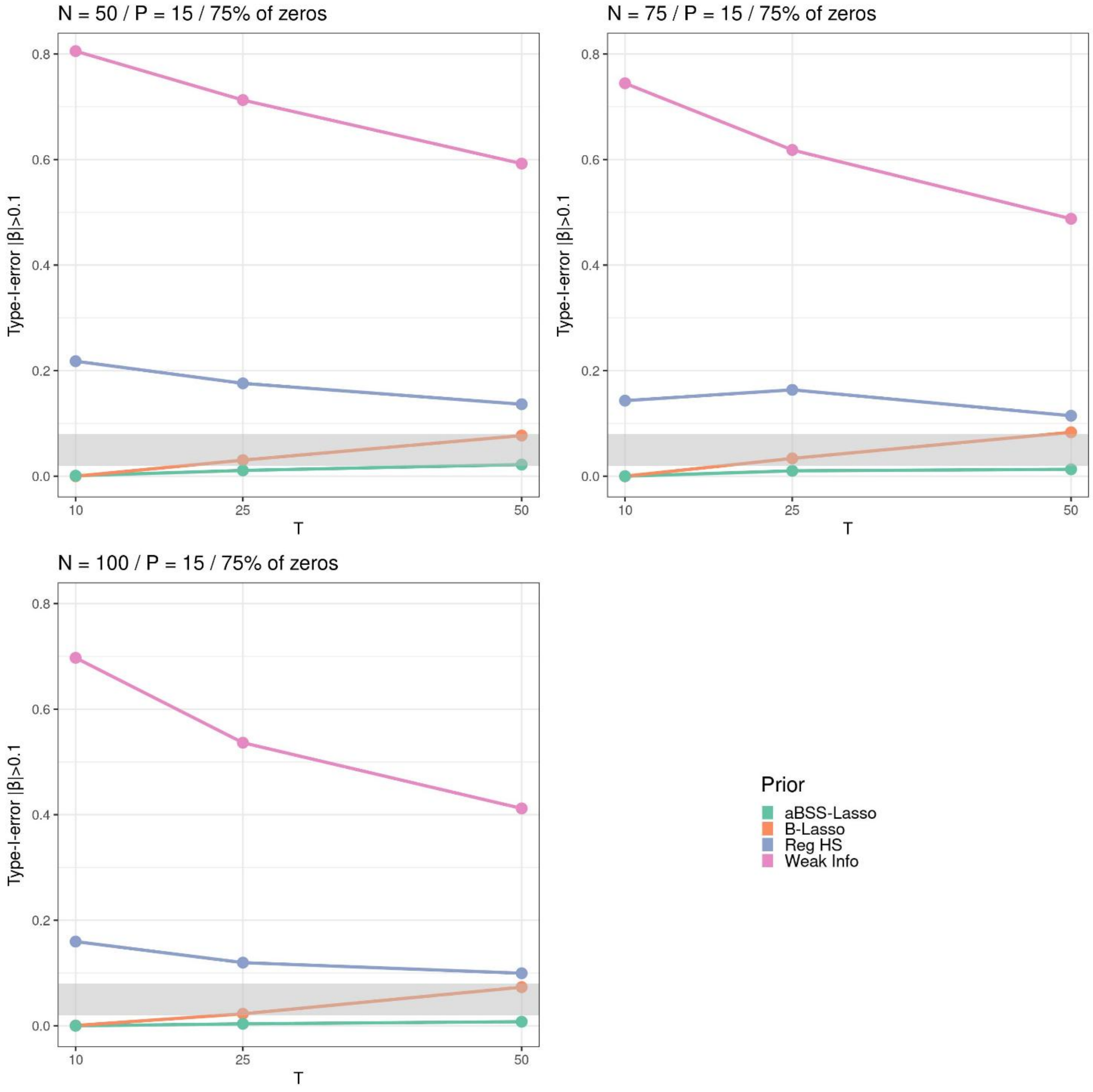

Figure 69: Type I error rate based on $|\beta_p| > 0.1$'s threshold across N and T when $P = 25$ and 75% of the elements of the $\boldsymbol{\beta}^*$ are zeros (simulation study 1).

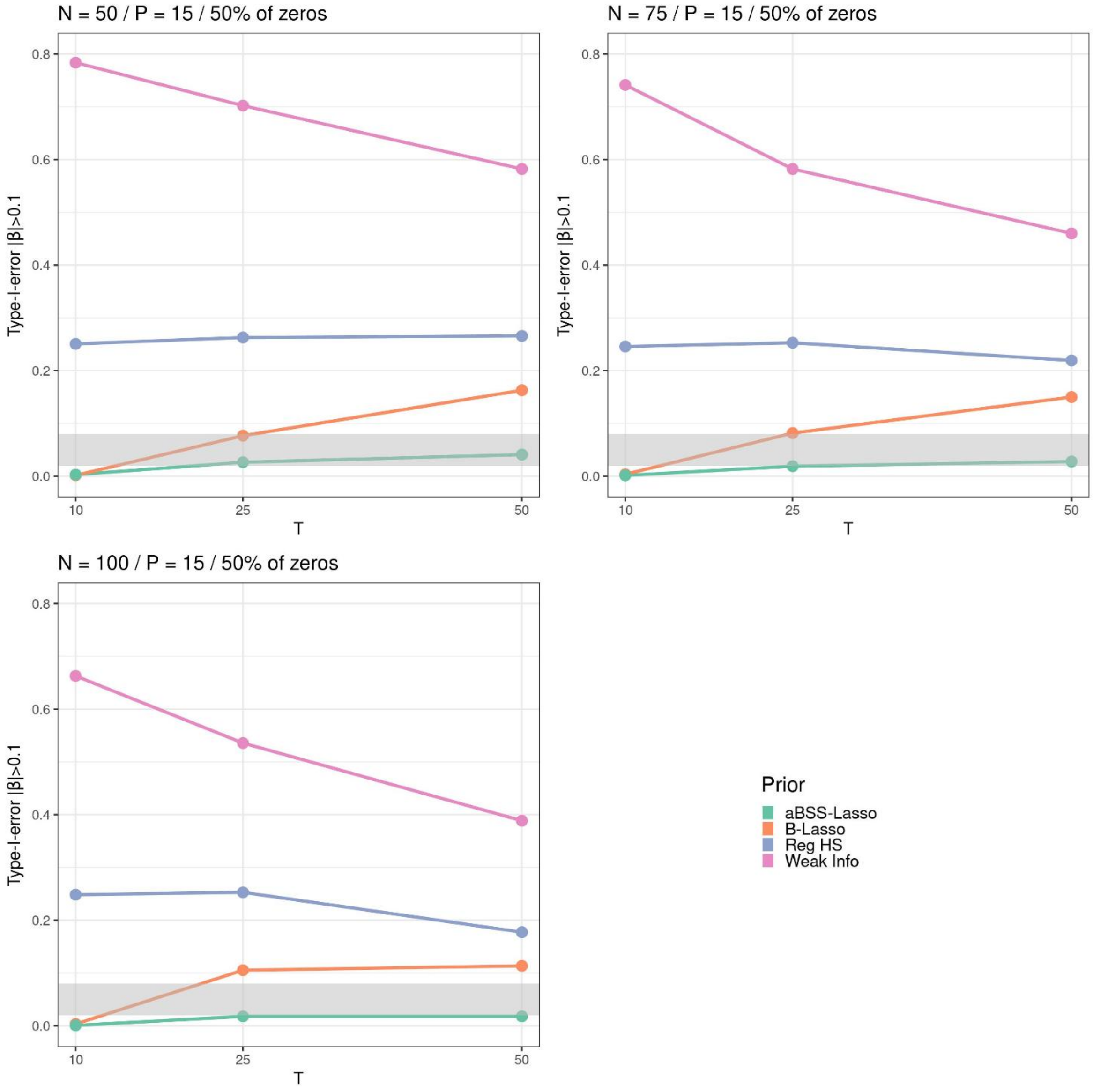

Figure 70: Type I error rate based on $|\beta_p| > 0.1$'s threshold across N and T when $P = 25$ and 50% of the elements of the $\boldsymbol{\beta}^*$ are zeros (simulation study 1).

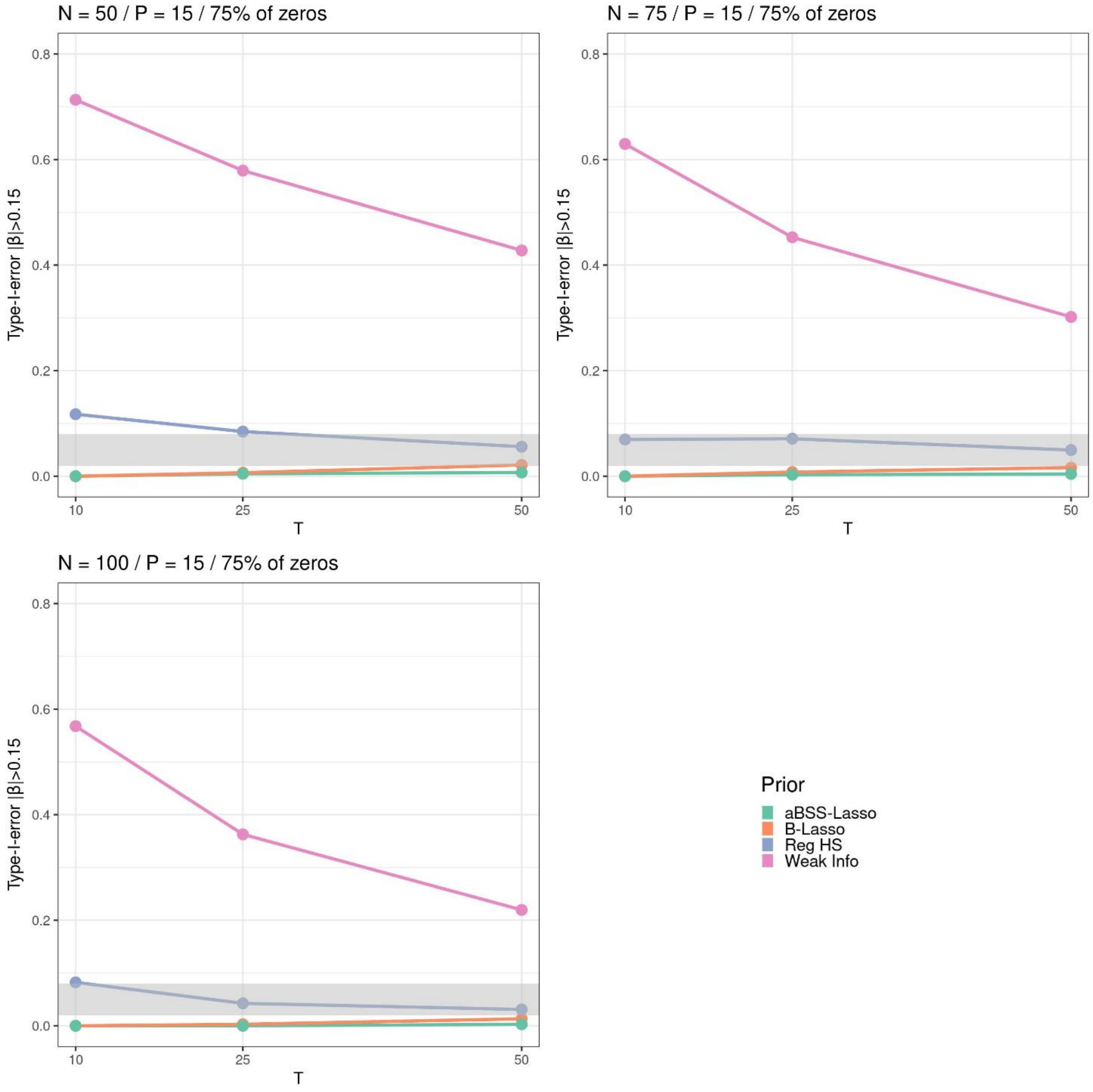

Figure 71: Type I error rate based on $|\beta_p| > 0.15$'s threshold across N and T when $P = 25$ and 75% of the elements of the $\boldsymbol{\beta}^*$ are zeros (simulation study 1).

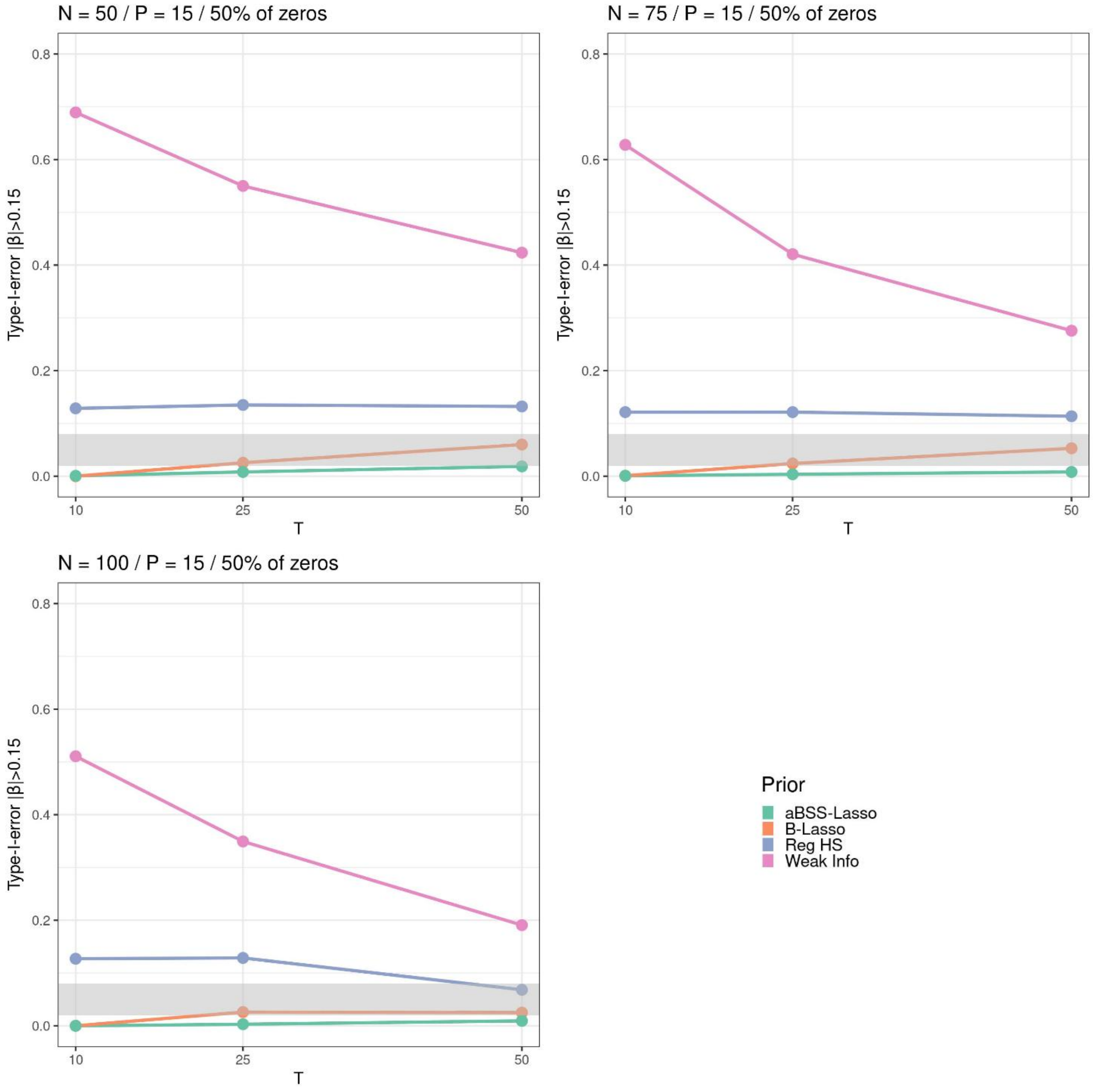

Figure 72: Type I error rate based on $|\beta_p| > 0.15$'s threshold across N and T when $P = 25$ and 50% of the elements of the $\boldsymbol{\beta}^*$ are zeros (simulation study 1).

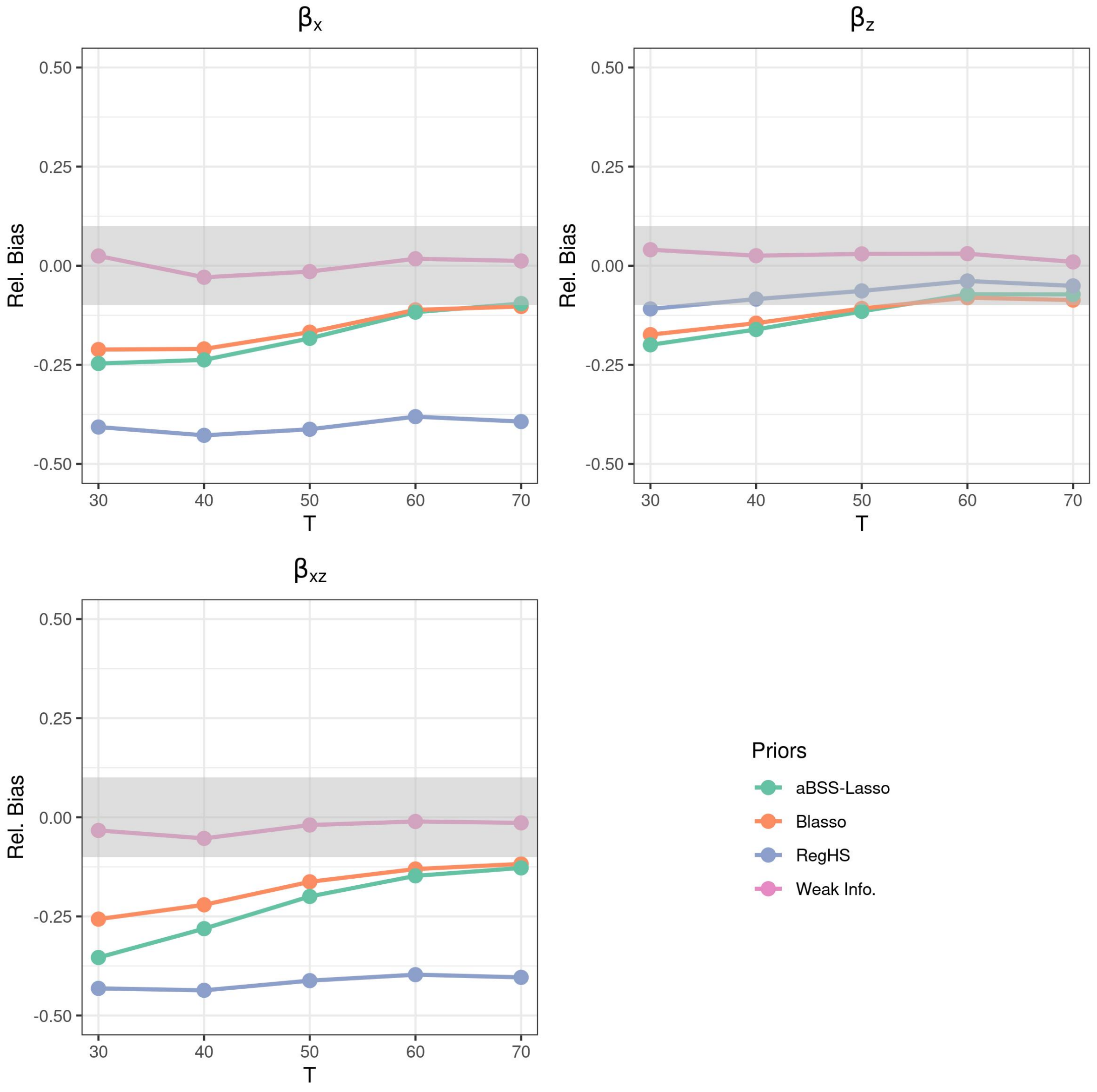

Figure 73: Relative bias for simulation study 2.

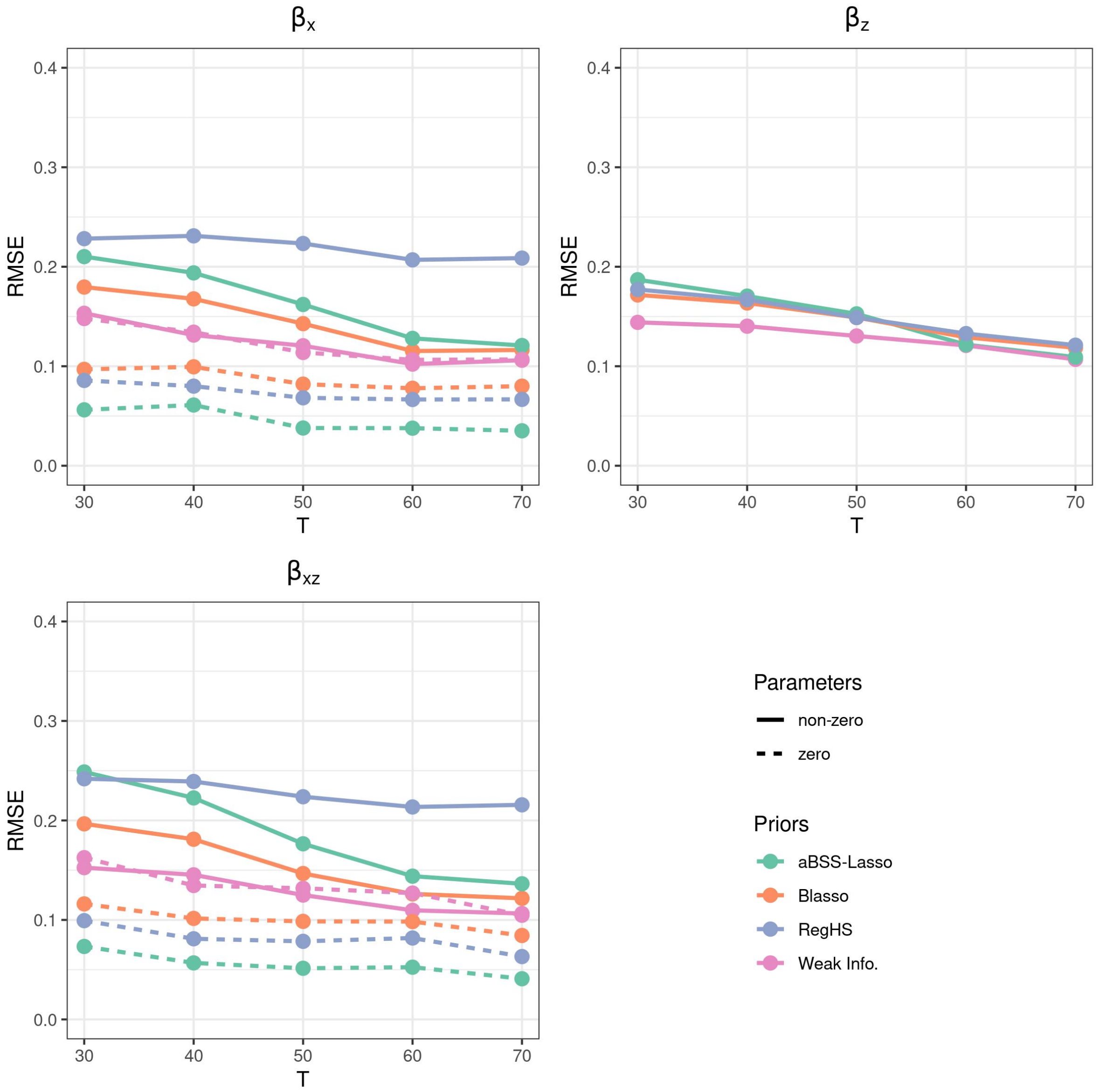

Figure 74: RMSE for simulation study 2.

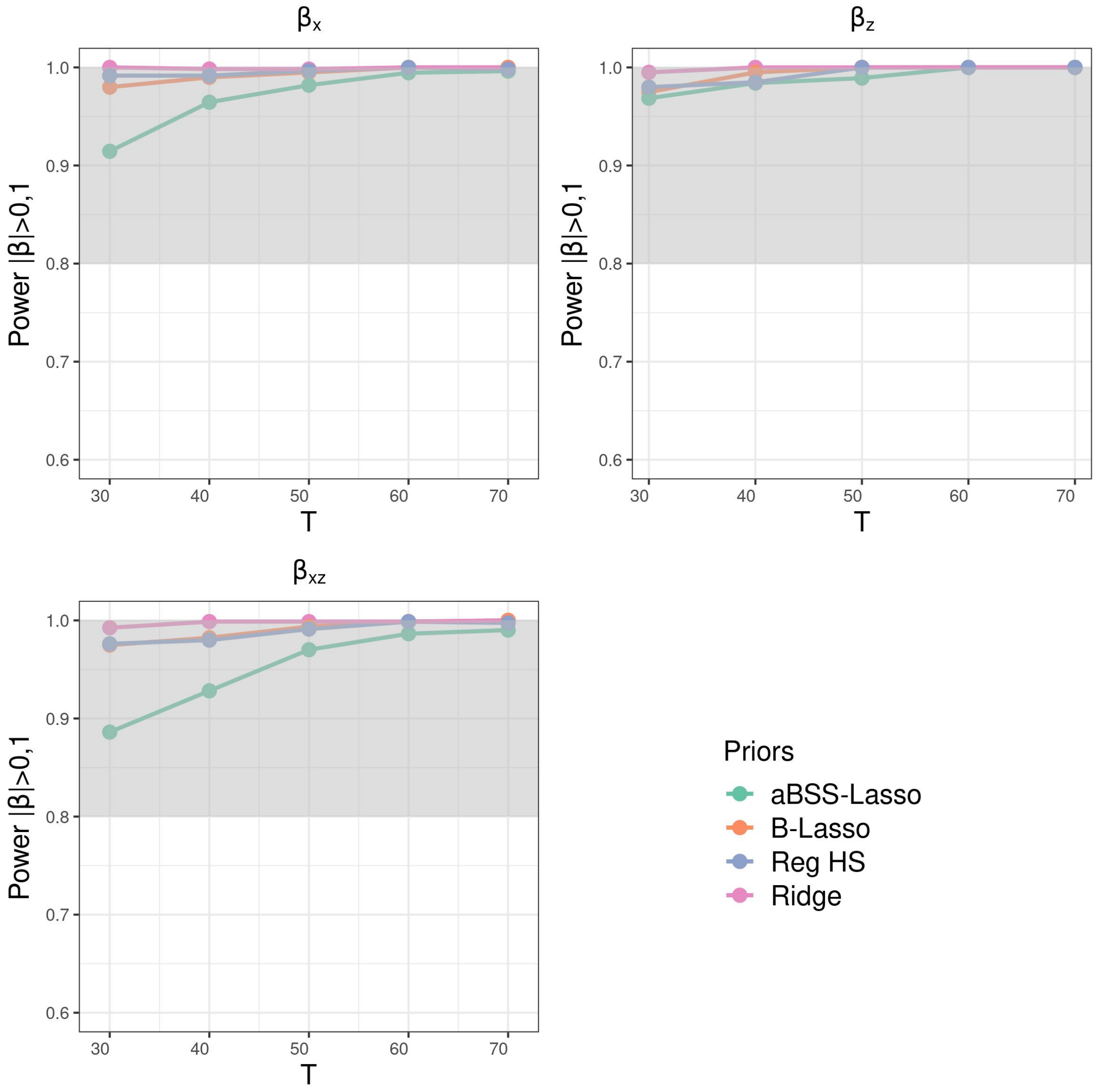

Figure 75: Power rate based on $|\beta_p| > 0.1$'s threshold for simulation study 2.

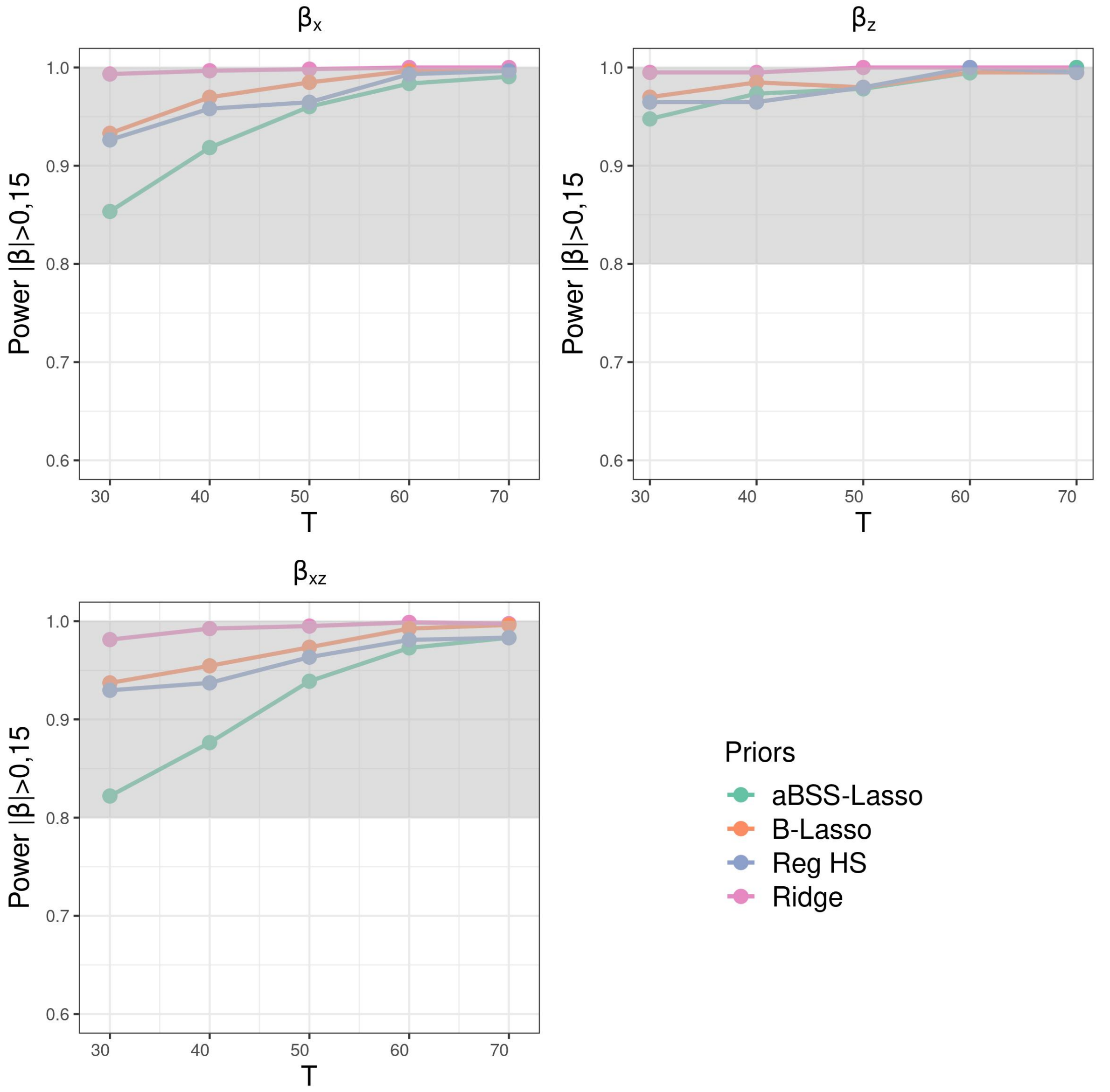

Figure 76: Power rate based on $|\beta_p| > 0.15$'s threshold for simulation study 2.

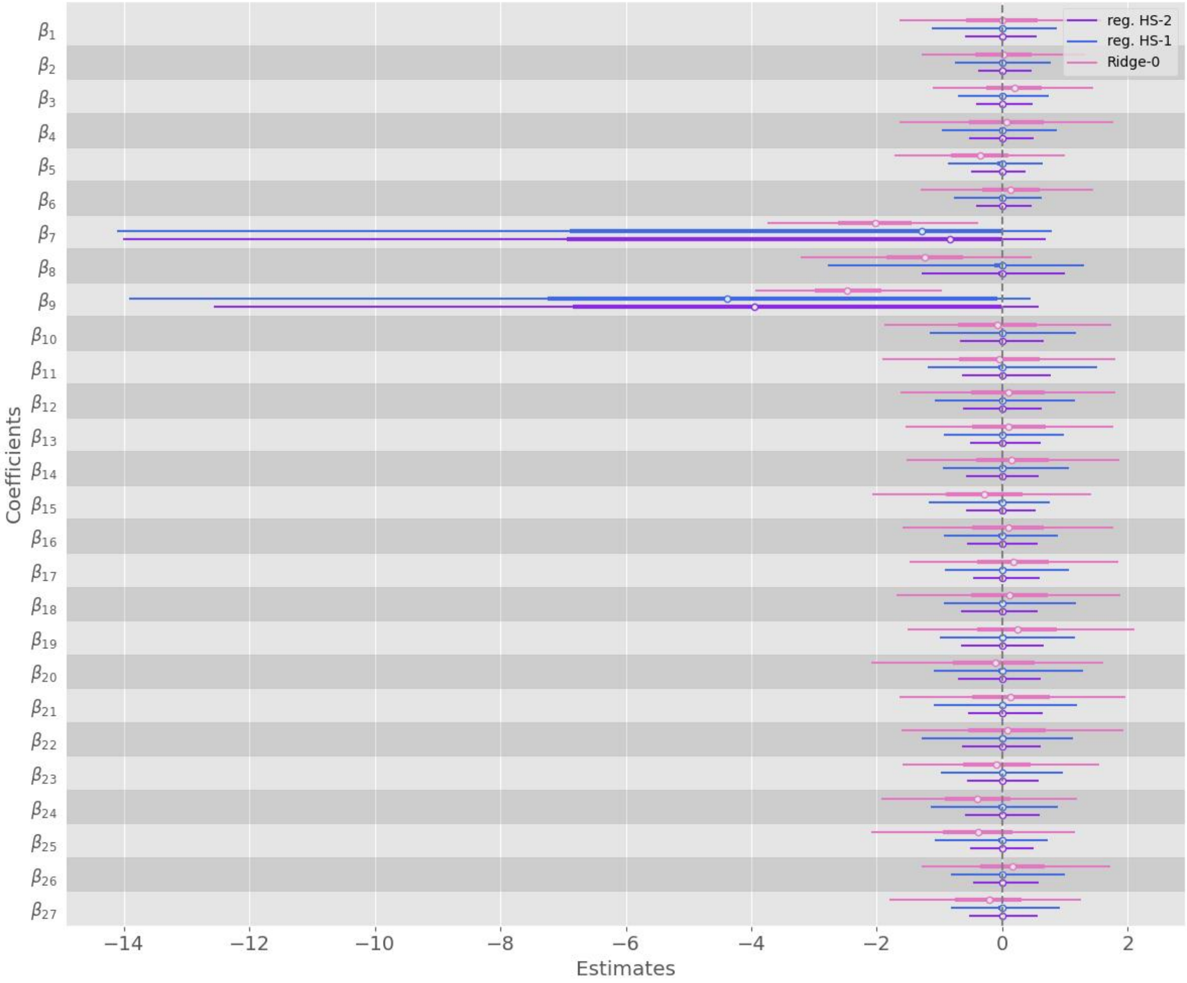

Figure 77: Comparison of the ridge priors and the reg. HS priors across all $\boldsymbol{\beta}^*$'s components. The thin lines correspond the 95% highest posterior density interval, the thick lines corrpesond to the quartiles and the white circle correspond to the posterior means.

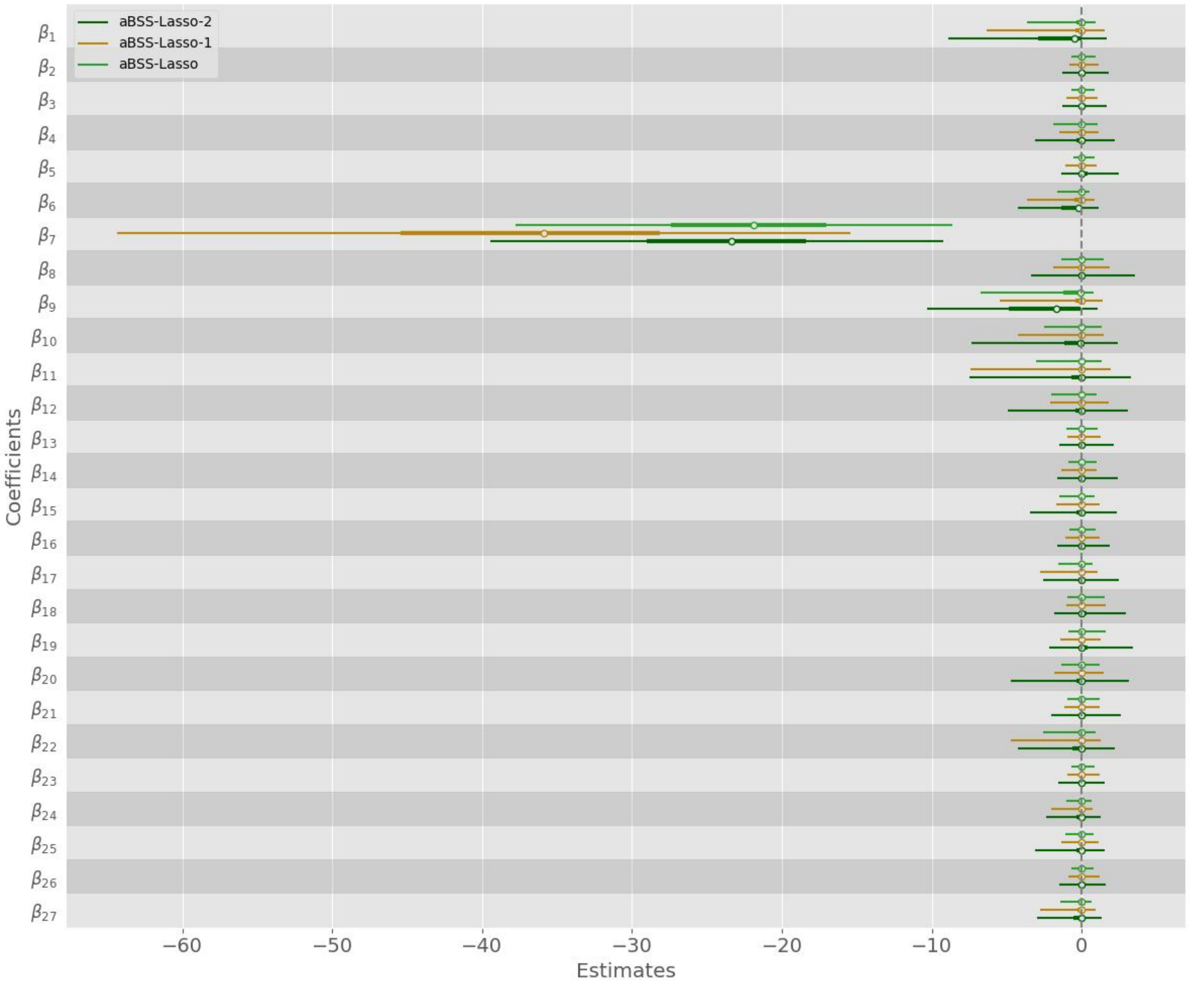

Figure 78: Comparison of the aBSS-Lasso priors with differents scaling across all $\boldsymbol{\beta}^*$'s components. The thin lines correspond the 95% highest posterior density interval, the thick lines corrpesond to the quartiles and the white circle correspond to the posterior means.

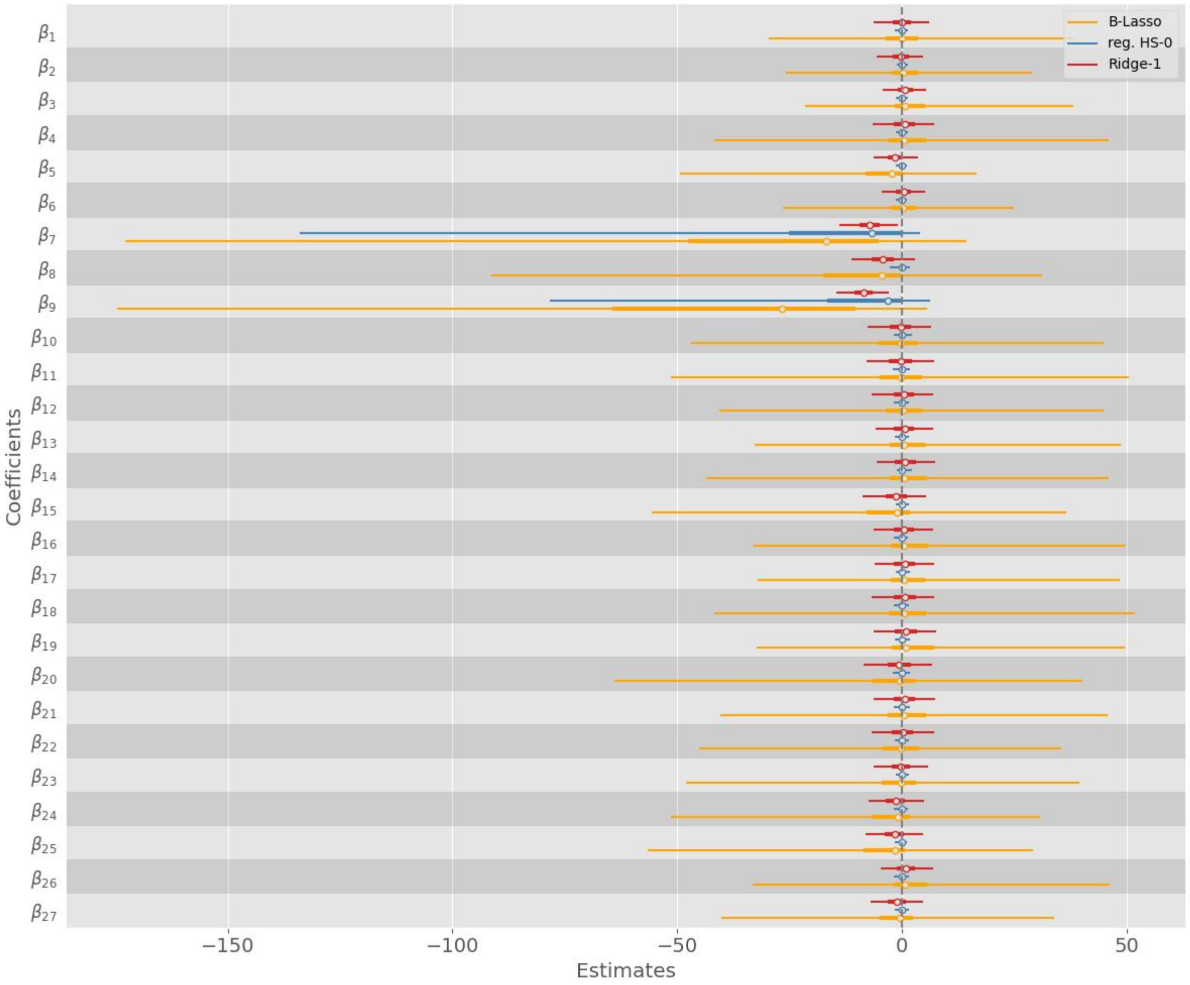

Figure 79: Comparison of the ridge prior (with $\sigma_\eta^2 = 4$), reg. HS prior (with Cauchy tails) and the B-Lassp prior across all $\boldsymbol{\beta}^*$'s components. The thin lines correspond the 95% highest posterior density interval, the thick lines corrpesond to the quartiles and the white circle correspond to the posterior means.

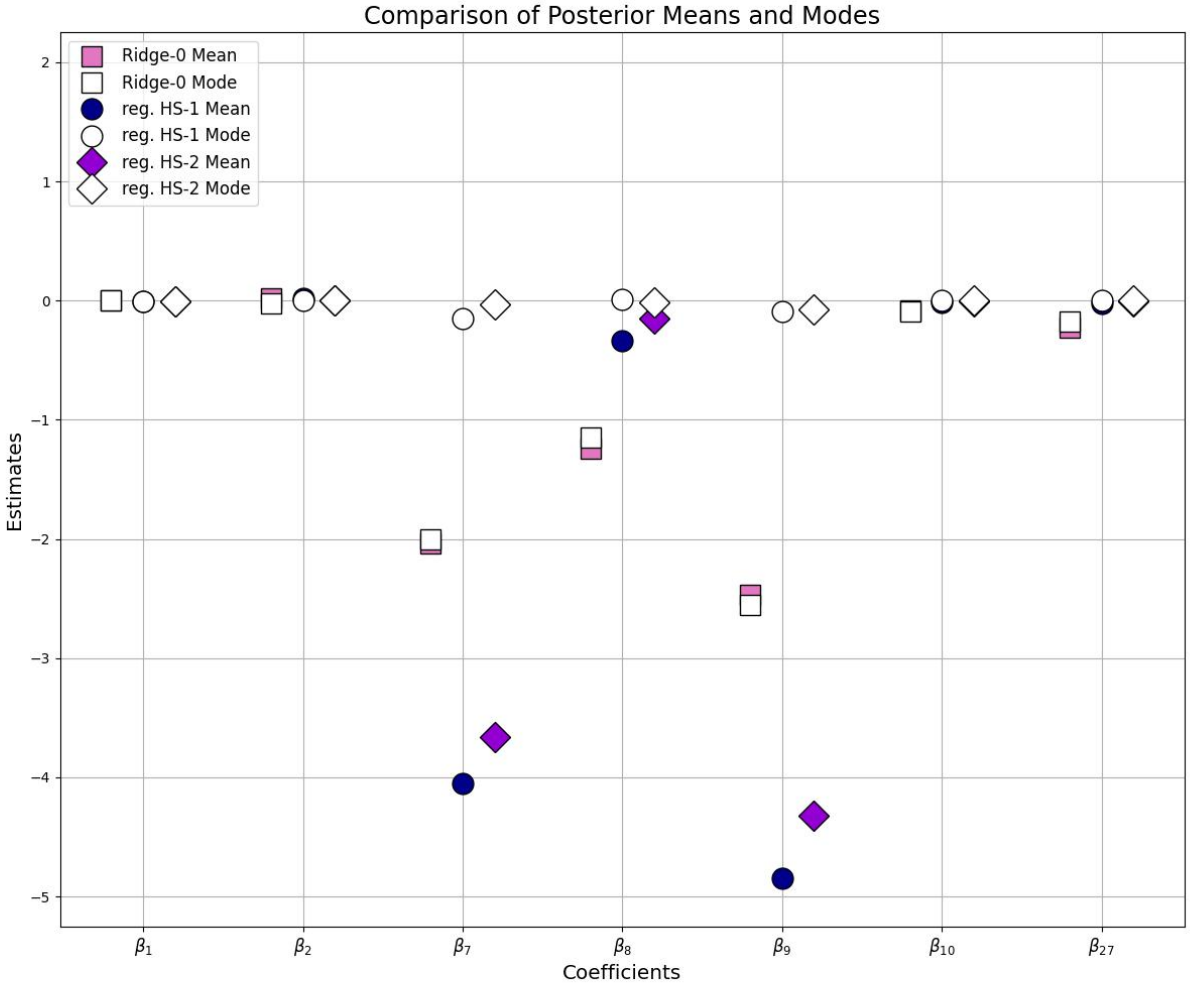

Figure 80: The posterior means (colored symbols) and the posterior modes (white symbols) of the ridge priors and the reg. HS priors for some selected coefficients β_p i.e. for $p = 1, 2, 7, 8, 9, 10, 27$. The rest of the β_ps were ignored as they were all close to zero (and were similar to $\beta_1, \beta_2.\beta_{10}$ and β_{27}).

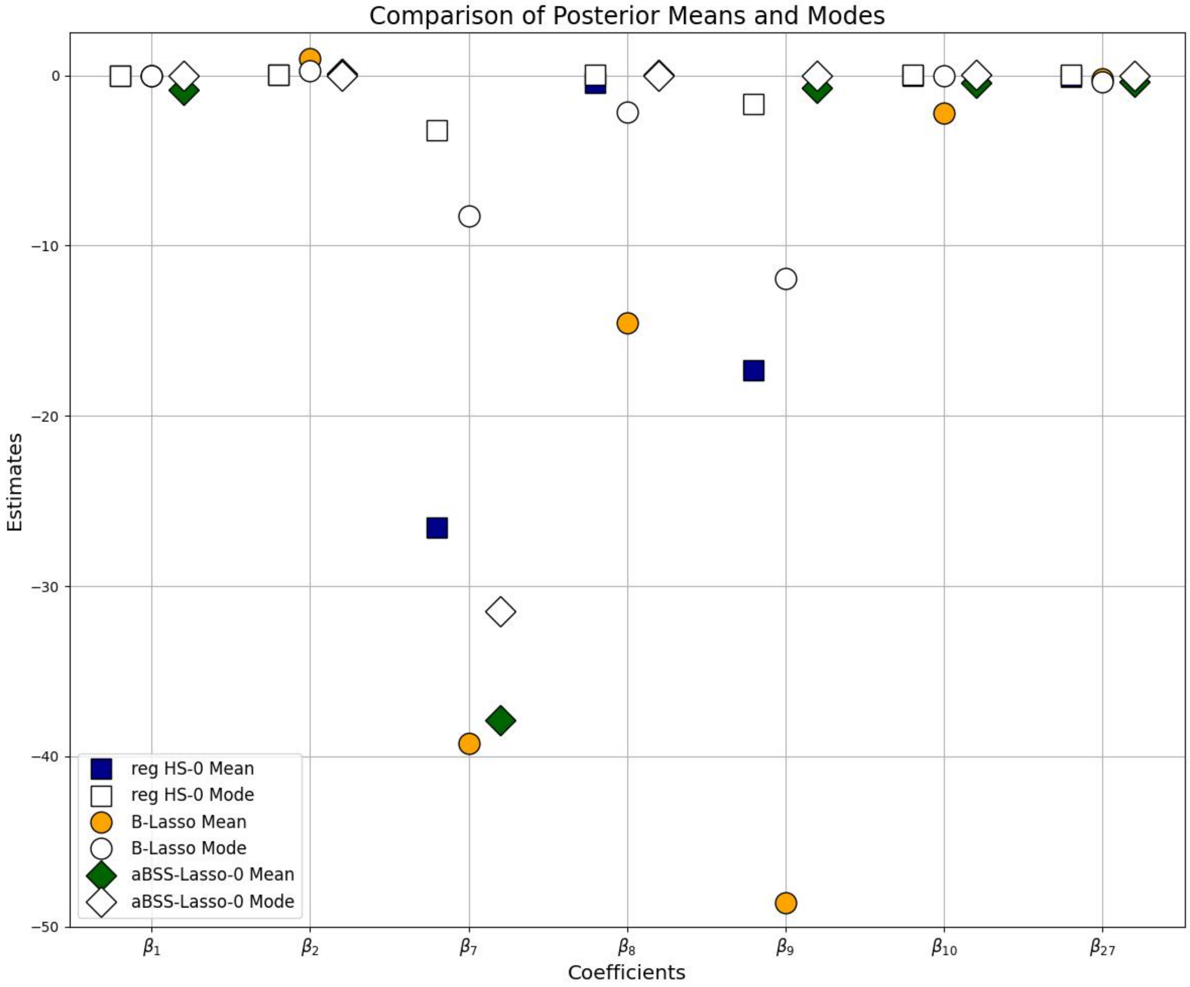

Figure 81: The posterior means (colored symbols) and the posterior modes (white symbols) of the ridge prior, the B-Lassp prior and the aBSS-Lasso prior for some selected coefficients β_p i.e. for $p = 1, 2, 7, 8, 9, 10, 27$. The rest of the β_ps were ignored as they were all close to zero (and were similar to $\beta_1, \beta_2.\beta_{10}$ and β_{27}).
